# Dynamic redox reactivation of platinum sites for long-term glycolate electrosynthesis from polyethylene terephthalate wastes

**DOI:** 10.1126/sciadv.aei5545

**Published:** 2026-11-04

**Authors:** Zehao Xiao, Hongyu Guo, Yu Gu, Changze Wei, Zheng Lin, Mingchuan Luo, Shaojun Guo

**Affiliations:** School of Materials Science and Engineering, Peking University, Beijing 100871, China.

## Abstract

Glycolates are indispensable in the health, chemical and pharmaceutical industries, whereas their mainstream production involve toxic chemicals and harsh operating condition. An alternative route for glycolate production is electrifying ethylene glycol (EG) that originates from polyethylene terephthalate (PET) but suffers from poor durability of active platinum site that tend to deactivate. Herein, we demonstrate a dynamic redox reactivation strategy, which alternates between working potential for oxidizing EG to glycolate and nonworking potential for reviving active platinum sites by the chemical reduction of adsorbed EG, realizing an unprecedented stability for over 3000 hours under average current density of ∼220 milliamperes per square centimeter. Upscaling this approach, the assembled electrolyzer drives a record current of 10 amperes at 1.18 volts for repeatable 40 batches of glycolates electrosynthesis, with each batch converting 48.0 grams of PET to 11.6 grams of glycolates (52.8% yield), 37.7 grams of *p*-phthalic acid (95.8% yield), 248.7 grams of NaHSO_4_ (95.2% yield) and ∼11.5 liters of hydrogen, showcasing a considerable revenue of ∼$1072 per ton glycolate.

## INTRODUCTION

Glycolic acid (GA) and glycolates with a global annual demand over 200,000 tons and a market value over $400 million are widely used in skin-caring, medicine, chemical washing, dyeing, bactericide, and food additives (*1*–*3*). The GA market is expected to reach $604.8 million by 2030 under a rapid annual growth rate of ∼6.1% (*4*). Although GA widely exists in natural plants, such as sugar cane, sugar beet, and grape, the direct extraction is impractical owing to its low content and great compatibility with other organic acids (*5*, *6*). Now, chloroacetic acid and formaldehyde carbonylation processes are mainstream production routes for GA and glycolates (Fig. 1A), necessitating the heavy use of toxic reactants and harsh reaction conditions, leading to environmental concerns (*7*, *8*). In this context, developing green synthesis route for GA and glycolates with economic feasibility is extremely desirable.

**Fig. 1.**
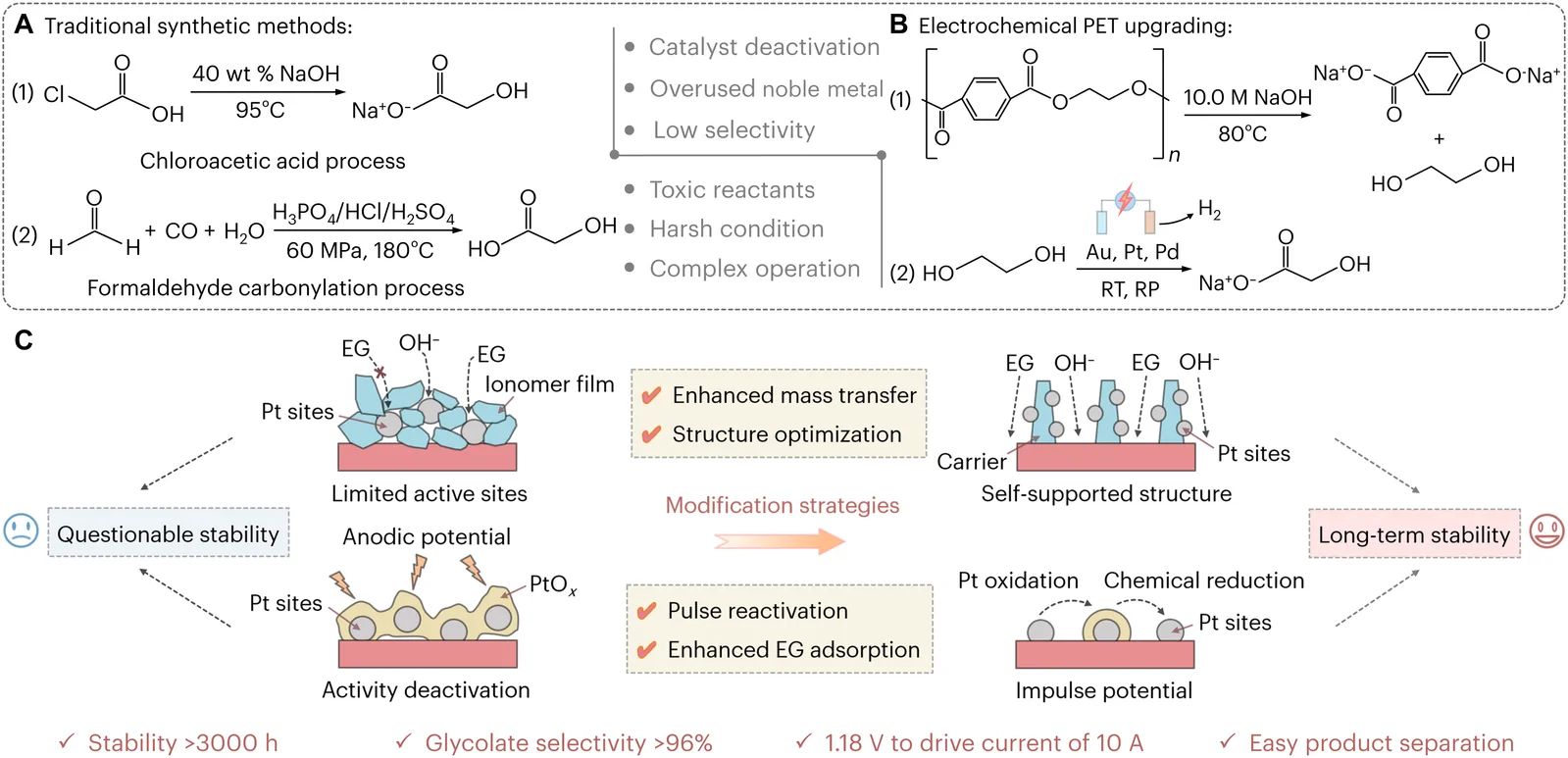
Schematics of GA/glycolate production routes. (**A**) Traditional industrial production routes of GA and glycolates. (**B**) Glycolate electrosynthesis route through electrochemical PET upgrading. RT, room temperature; RP, room pressure. (**C**) Strategies proposed in this work to promote the glycolate electrosynthesis through electrochemical PET upgrading. h, hours.

With the rapidly growing electricity from renewable energy (e.g., wind, solar, tidal wave, and nuclear), it becomes appealing to generate value-added products via electrochemical upgrading of wastes (*9*–*11*). Polyethylene terephthalate (PET) is a widely used plastic with an annual production over 70 million tons. The alkaline hydrolysis of PET generates terephthalates and ethylene glycol (EG) monomers, which potentially enables a cycling economic (Fig. 1B and fig. S1) (*12*–*14*). Although the former can be easily separated by precipitation to reproduce PET, however, it is challenging to separate EG from the hydrolysis solution due to high boiling point and water solubility (*15*, *16*). Alternatively, the EG contained alkaline solution can be fed as an electrolyte for electrochemical PET upgrading, which simultaneously enables value-added products from EG oxidation reaction (EGOR) and coproduction of green hydrogen fuels (figs. S2 and S3) (*17*, *18*). In this system, active and selective catalysts are necessary for economic feasibility. In particular, noble metal (e.g., Pt, Pd, and Au)–based catalysts steer EGOR to value-added glycolate or carbonate under low overpotential (*19*, *20*). Among them, Pt exhibits favorable adsorption energetics and efficient dehydrogenation kinetics toward EG, coupled with superior tolerance to carbonaceous intermediates, thus acting as a high-performance candidate for EGOR. Nevertheless, the catalytic durability and production rates of available Pt-based catalysts still remain far from satisfactory for the application in the large-scale glycolate electrosynthesis, primarily due to limited mass transport and rapid oxidative deactivation of active Pt sites (Fig. 1C) (*21*). Taking the commercial 20 wt % Pt/C catalyst for an example, its working current density attenuates by ∼80% after 5 hours of EGOR (fig. S4).

Herein, we report a class of Pt nanoparticles (NPs) decorated on hydroxide nanosheet arrays [Pt/NiCo(OH)*_x_*] with enhanced mass transport and a cyclic intermittent potential (IP) approach to address the oxidation-induced decay of Pt sites during EGOR. Such catalyst exhibits 300 mA cm^−2^ for EGOR at 0.65 V versus reversible hydrogen electrode (versus RHE). We demonstrate the dynamic redox reactivation protocol—working potential of 1.05 V versus RHE for 1200 s to produce glycolates with nonworking IP of 0.1 V versus RHE for 2 s to revive Pt sites by the strong chemical reduction ability of adsorbed EG in the electrolyte—that efficiently maximizes the catalytic performance of active Pt sites, achieving unprecedentedly durable EGOR electrocatalysis for over 3000 hours with an average current density of ∼220 mA cm^−2^ and a glycolate selectivity of >96%. Experimental and theoretical results reveal a synergistic effect between hydroxides and loading Pt NPs for promoting EG adsorption, *OH adsorption, and water dissociation. In addition, we extended scale-up glycolate electrosynthesis from waste PET, which could afford a record current of 10 A (current density > 400 mA cm^−2^) at a low voltage of 1.18 V for repeatable 40 batches. With this technical route, we demonstrated the production of 11.6 g of sodium glycolate (yield of 52.8%), along with 37.7 g of *p*-phthalic acid (PTA; yield of 95.8%), 248.7 g of NaHSO_4_ (yield of 95.2%), and ∼11.5 liters of hydrogen gas from 48.0 g of PET powders with a considerable revenue of ∼$1072 per ton glycolate.

## RESULTS

### Structural characterizations of the Pt/NiCo(OH)*_x_* catalyst

We designed and prepared the Pt/NiCo(OH)*_x_* catalyst on nickel foam (NF; fig. S5) via sequential hydrothermal, thermal annealing reduction, and electrodeposition (Fig. 2A and figs. S6 and S7; PtCl_6_^2−^ + 4e^−^ → Pt + 6Cl^−^). The inductively coupled plasma optical emission spectrometry (ICP-OES) analysis of Pt/NiCo(OH)*_x_* shows that the Pt mass loading is around 0.52 mg cm^−2^. Scanning electron microscopy (SEM) images of the NiCo(OH)_2_ nanosheets show that they are densely grown on NF, and their ingenious structure is still retained after annealing reduction (figs. S8 to S11). The as-made Pt/NiCo(OH)*_x_* catalyst consists of densely cross-connected thin nanosheets after electrodeposition under an alkaline electrolyte (Fig. 2B and fig. S12). Transmission electron microscopy (TEM) images of Pt/NiCo(OH)*_x_* show that plenty of highly dispersed particles with 10 nm in grain size are uniformly decorated on the interlaced nanosheets (Fig. 2C). The high-resolution TEM (HRTEM) image shows the typical (111) plane of Pt NPs, whereas the energy-dispersive spectroscopy (EDS) elemental mapping images of Pt/NiCo(OH)*_x_* further exhibit the distribution of loaded Pt NPs (fig. S13). The EDS line scan of the region that includes a single particle proves the element distribution of Pt (fig. S14). In contrast, direct electrodeposited Pt NPs on NF (Pt NPs/NF) are irregular with poor dispersion (fig. S15).

**Fig. 2.**
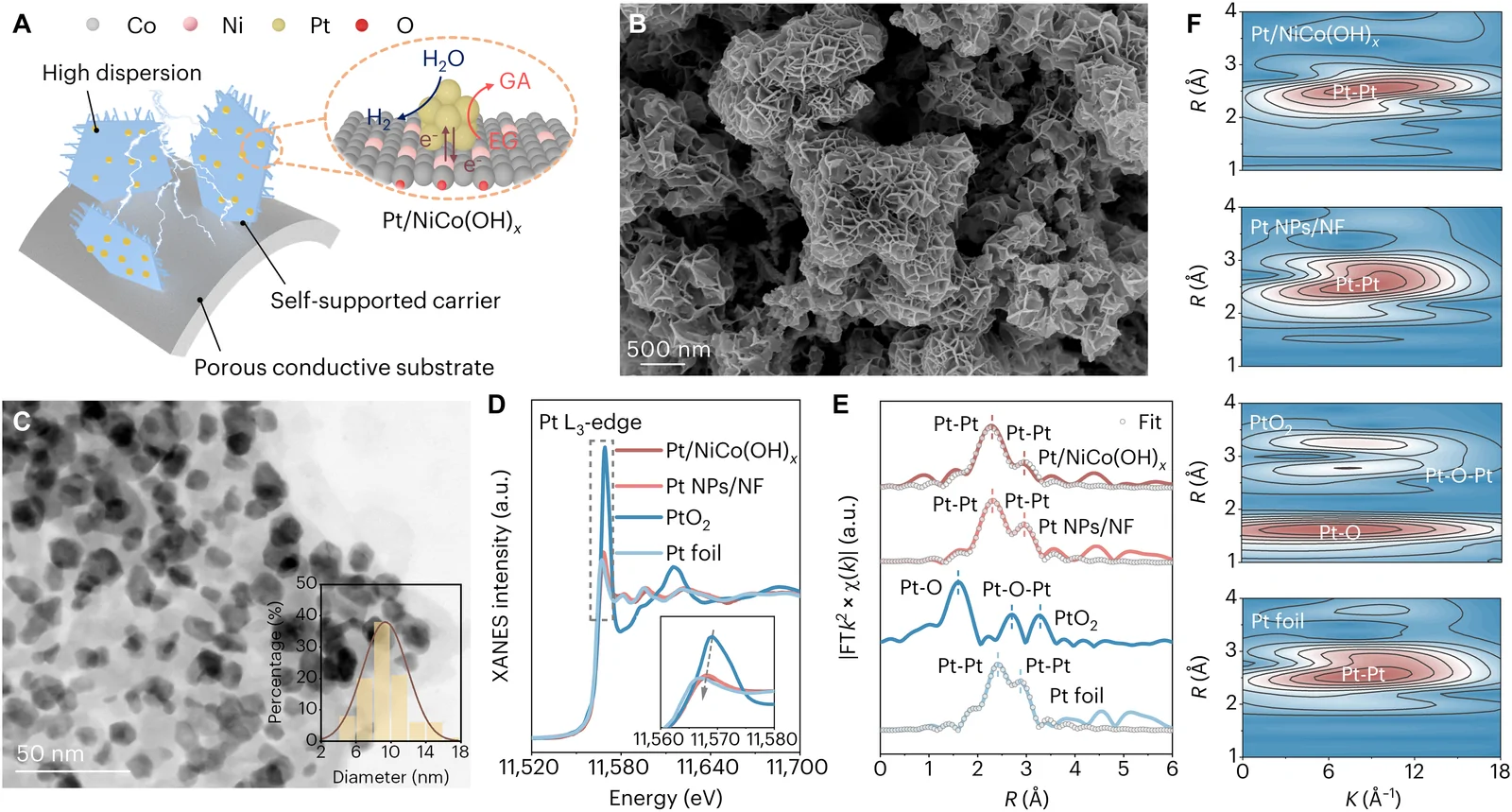
Structural characterizations. (**A**) Structural illustration of Pt/NiCo(OH)*_x_*. (**B**) SEM image and (**C**) TEM image of Pt/NiCo(OH)*_x_* with the corresponding loading Pt particle size statistical diagram. (**D**) Normalized Pt L_3_-edge XANES spectra. a.u., arbitrary units. (**E**) FT Pt L_3_-edge EXAFS spectra. (**F**) WT *k*^2^-weighted χ(*k*) function at the Pt L_3_-edge.

The x-ray diffraction (XRD) pattern of Pt/NiCo(OH)*_x_* catalyst shows the signals of face-centered cubic Pt, metallic cobalt and NF substrate (fig. S16). Fourier transform infrared (FTIR) and Raman spectra show the presence of metal hydroxides, possibly generated from the redox reaction [PtCl_6_^2−^ + 2Co + 4OH^−^ → 2Co(OH)_2_ + 6Cl^−^ + Pt] during the electrodeposition under alkaline media (fig. S17) (*22*, *23*). The high-resolution Pt 4f x-ray photoelectron spectroscopy (XPS) spectra of Pt/NiCo(OH)*_x_* and Pt NPs/NF show the coexistence of Pt^0^ and Pt^2+^ valence states (fig. S18A and table S1). The high-resolution Co 2p XPS spectra of Pt/NiCo(OH)*_x_* show the decreasing signal of the characteristic peak for the metallic Co^0^ state, further demonstrating the conversion of partial Co sites to Co(OH)_2_ states during the electrodeposition process (fig. S18B) (*24*, *25*).

The local coordination structure and chemical states are explored by extended x-ray absorption fine structure (EXAFS) tests. As shown in Fig. 2D, the nearly overlapping white-line peaks of Pt L_3_-edge x-ray absorption near-edge structure (XANES) spectra for Pt/NiCo(OH)*_x_* indicate a similar average valence state with the standard Pt foil. The Fourier transform (FT) *k*^2^-weighted Pt L_3_-edge EXAFS spectra (Fig. 2E) of Pt/NiCo(OH)*_x_*, Pt NPs/NF, and Pt foil show two peaks indicative of metallic Pt─Pt bonds instead of its oxidation features (table S2) (*26*). The wavelet transform (WT) Pt L_3_-edge EXAFS spectra in *K* and *R* spaces display the typical signal of the Pt─Pt bond at 8.9 Å^−1^ (Fig. 2F) (*27*). In the Co K-edge XANES spectra, the average valence of Co sites in Pt/NiCo(OH)*_x_* situates between standard Co foil and CoO*_x_* (fig. S19). In the FT *k*^3^-weighted Co K-edge EXAFS spectra of Co, the peak at 2.1 Å is attributed to Co─Co bonds and indicates the metallic bulk state (figs. S20 and S21) (*28*).

### Electrocatalytic alkaline EGOR performances

EGOR activity determines the production ability of EG to value-added chemicals. Electrocatalytic activities of synthesized catalysts were evaluated in the three-electrode system. Impressively, in the presence of EG, the Pt/NiCo(OH)*_x_* catalyst only requires potentials of 0.54 and 0.65 V versus RHE to drive current densities of 100 and 300 mA cm^−2^, whereas those potentials decrease for ∼1.0 V compared to oxygen evolution reaction (OER), representing the promising feasibility to simultaneously achieve energy-saving hydrogen production with efficient EG oxidation (figs. S22 and S23). Notably, the EGOR activity of Pt/NiCo(OH)*_x_* can be optimized by tuning the content of PtCl_6_^2−^ in electrolyte and deposition cycles during the electrodeposition (fig. S24). However, nickel-cobalt species have nearly no EGOR activity in such a low potential range. The higher EGOR activity for Pt/NiCo(OH)*_x_* than that of Pt NPs/NF demonstrates transition metal oxidized layers [e.g., CoO*_x_*, Co(OH)_2_, and CoOOH] are beneficial to promoting the electrochemical surface area (ECSA) of active Pt sites (fig. S25) and intrinsic activity (Fig. 3A and fig. S26) with higher turnover frequency (fig. S27).

**Fig. 3.**
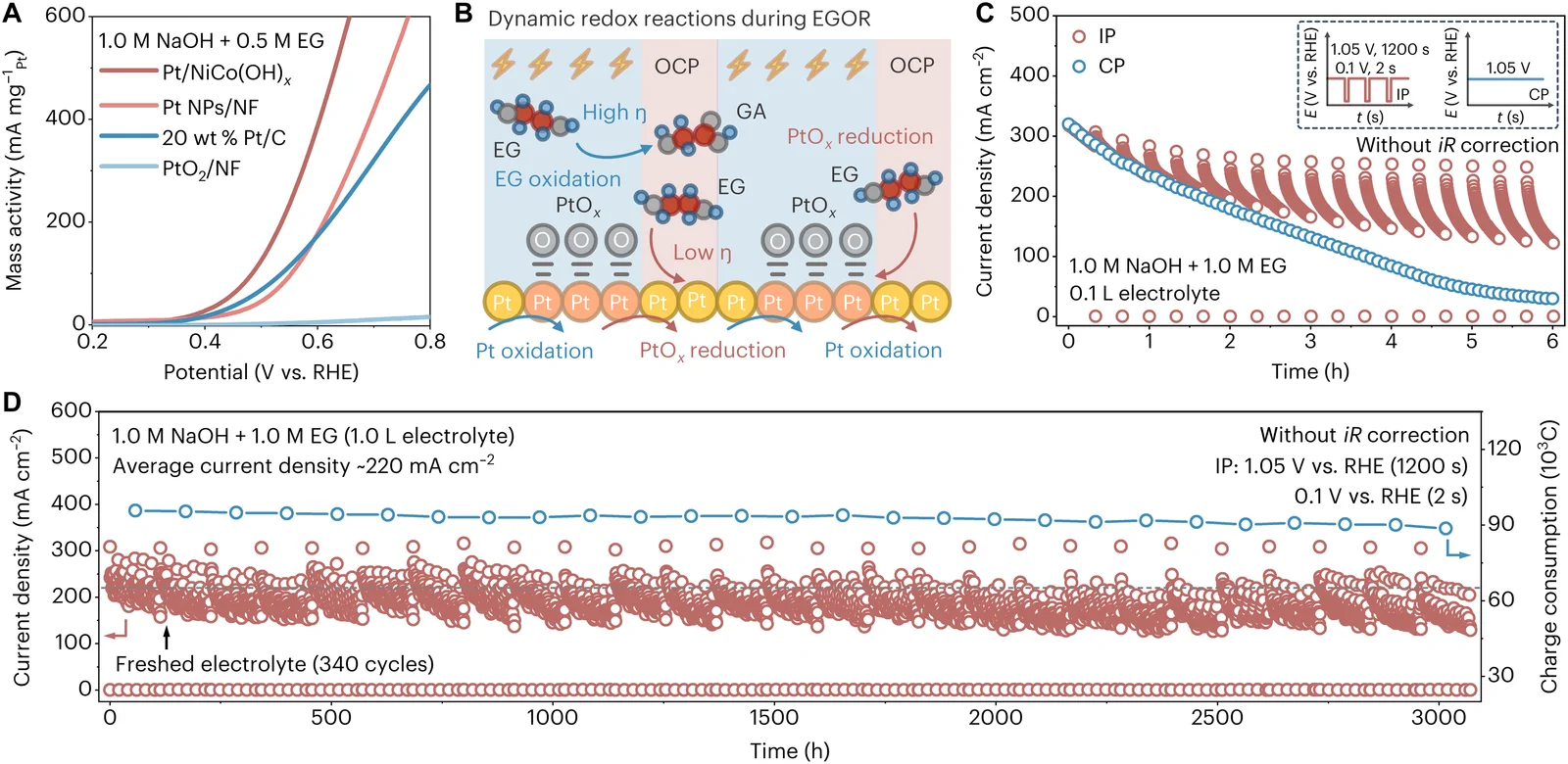
Electrocatalytic activities toward EGOR. (**A**) Pt mass loading–corrected EGOR LSV curves of Pt/NiCo(OH)*_x_* (0.52 mg_Pt_ cm^−2^), Pt NPs/NF (0.54 mg_Pt_ cm^−2^), 20 wt % Pt/C (0.48 mg_Pt_ cm^−2^), and PtO_2_/NF (0.65 mg_Pt_ cm^−2^). (**B**) Dynamic redox reaction illustration of the EGOR process. (**C**) EGOR stability test of Pt/NiCo(OH)*_x_* through CP (1.05 V versus RHE) and IP (1.05 V versus RHE for 1200 s; 0.1 V versus RHE for 2 s) modes. L, liters. (**D**) Long-term IP electrolysis and consumed charges for each batch of EGOR with the Pt/NiCo(OH)*_x_* electrode.

Although Pt-based catalysts generally require much lower operating potential than non-noble-metal catalysts in EGOR, their further application is severely limited by the inescapable oxidation of Pt to PtO*_x_* species, resulting in rapid activity deactivation (*29*). Of note, cyclic voltammetry (CV) curves of Pt/NiCo(OH)*_x_*, Pt NPs/NF, and 20 wt % Pt/C in 1.0 M NaOH exhibit two pairs of redox peaks for Pt sites (fig. S25), which are assigned to the hydrogen adsorption/desorption of Pt-H (∼0.26 V versus RHE) and the surface oxidation/reduction of PtO*_x_* (∼0.69 V versus RHE) (*22*). To mitigate the detrimental deactivation caused by Pt oxidation during EGOR, we propose that the reducing ability of adsorbed EG with multiple hydroxyl groups can effectively restore PtO*_x_* to active metallic Pt in the nonworking potential region of EGOR (fig. S28). As expected, upon the addition of 0.1 M EG, the open-circuit potential (OCP) decreased more substantially for Pt/NiCo(OH)*_x_* (0.71 V) than that for Pt NPs/NF (0.67 V) and 20 wt % Pt/C (0.61 V), indicating enhanced EG adsorption at the Helmholtz layer (fig. S29) (*30*).

With this in mind, we herein proposed a kind of IP test method for EGOR, which circularly alternated between the long-term EGOR under large current density in the high working potential region and chemical reduction of PtO*_x_* to catalytically active Pt sites in the low nonworking region (Fig. 3B). Notably, a short period of IP is ready to revive the EGOR activity (fig. S30). We then compared the EGOR stability between traditional constant potential (CP) with IP methods for Pt/NiCo(OH)*_x_* (Fig. 3C). The average current density of IP electrolysis is much higher than that of CP, and such a phenomenon also applies for the commercial 20 wt % Pt/C (fig. S31) and similar Pd/Au catalysts (figs. S32 and S33). Impressively, we also demonstrate the advance of IP electrolysis in other applications, including glycerol and glucose oxidation reactions (figs. S34 and S35). Furthermore, we used IP methods to evaluate long-term EGOR stability under large current density in the self-made flow electrolyzer with circulating electrolyte (fig. S36). Impressively, Pt/NiCo(OH)*_x_* keeps unprecedented stability with negligible dissolution of Pt and Co under average current density of ∼220 mA cm^−2^ for over 3000 hours (Fig. 3D and fig. S37). Such remarkable performance outdistances most of previously reported noble metal–based catalysts for EGOR (fig. S38 and table S3). Notably, the estimated charge consumption per batch indicates that EG and NaOH are consumed by ∼20%. Although the solution could still support continued IP electrolysis, we set 340 cycles per batch to maintain a high average working current density. The SEM image of Pt/NiCo(OH)*_x_* after the EGOR stability test shows the morphology transforms into wrapped arrays of clusters (fig. S39). XRD pattern and XPS spectra of Pt/NiCo(OH)*_x_* after the EGOR stability test confirmed the presence and higher valence content of the Pt state (figs. S40 and S41). TEM images after the EGOR stability test show that Pt sites are still evenly distributed in the clusters (fig. S42). The Pt/NiCo(OH)*_x_* catalyst still maintains a high ECSA after morphology reconstruction (fig. S43).

### Electrocatalytic alkaline HER performances

The increasing hydrogen evolution reaction (HER) activity of Pt during the electrodeposition process indicates the successful loading of Pt NPs on NiCo(OH)*_x_* (fig. S44). HER polarization curves in 1.0 M NaOH indicate that Pt/NiCo(OH)*_x_* requires overpotentials of 83 and 142 mV to drive current densities of 100 and 300 mA cm^−2^ (figs. S45 and S46). The Tafel slope (40.3 mV dec^−1^) of Pt/NiCo(OH)*_x_* shows the typical Volmer-Heyrovsky reaction pathway (fig. S47). The largest hydrogen turnover frequency indicates that Pt/NiCo(OH)*_x_* owns the optimal active sites for accelerating HER (figs. S48 and S49). Notably, the HER stability test of the Pt/NiCo(OH)*_x_* catalyst exhibits excellent durability for over 270 hours under current density of 200 mA cm^−2^ (fig. S50). The SEM image of Pt/NiCo(OH)*_x_* after the HER stability test shows that the morphology transforms into an interlaced cluster array (fig. S51), whereas the vanishing peak in XRD pattern after the HER stability test suggests that the reconstructed hydroxides are in amorphous with negligible changes of the Pt^0^ state (figs. S52 and S53). In addition, the oxygen content in the EDS mapping area increases due to the enhanced surface hydroxyl coverage after the HER stability test (fig. S54).

### Operando characterizations of EGOR and HER

In situ Bode plots are used to explore the interfacial electrochemical dynamics (*31*). As shown in fig. S55, the characteristic peaks at the low-frequency region (0 to 5 Hz) represent the OH^−^ adsorption in 1.0 M NaOH. As for EGOR, the peaks under the middle-frequency region (10 to 100 Hz) represent the direct oxidation of EG (fig. S56). Notably, Pt/NiCo(OH)*_x_* has lower charge transfer resistance to accelerate the oxidation rate of adsorbed EG than Pt NPs/NF (fig. S57) (*32*). In situ EXAFS measurements in a flow electrolyzer were used to probe the evolution of the valence state and coordination structure of Pt sites during EGOR (fig. S58), and a higher white-line peak intensity is indicative of a higher Pt valence state (Fig. 4A). Upon the addition of EG, the OCP decreases and the oxidation state of Pt sites is initially reduced; however, as EGOR proceeds, the valence state of Pt sites progressively increases. Once the voltage is cut off, the valence state accordingly decreases, owing to the reducing capability of EG. As expected, the evolution of the Pt-Pt coordination (∼2.7 Å) in the FT Pt L_3_-edge EXAFS spectra mirrors the trend observed for the white-line peak (fig. S59). In situ Raman spectra (fig. S60) indicate that the Pt sites spontaneously form Pt─O bonds (572 cm^−1^) at the OCP (0.93 V versus RHE) in 1.0 M NaOH, and the signal of Pt─O bonds disappears after adding EG, suggesting that the adsorbed EG has chemical reduction ability for the unfavorable PtO*_x_* state. Therefore, the PtO*_x_* states formed during EGOR can be reduced by EG after the potential cutoff, which indicates that adsorbed EG revives active Pt sites under the short time of nonworking potential in the IP strategy, thereby reactivating efficient EGOR. The comparative tests of CP and IP in the solutions with different concentrations of EG show that the oxidation rate of Pt to PtO*_x_* is in dynamic equilibrium with the reduction rate of adsorbed EG (Fig. 4, B and C). Although the short time of nonworking potential can revive EGOR activity under the low concentration of EG, the activity decay rate rapidly increases in the lowest concentration of EG, indicating that the reduction rate of EG on PtO*_x_* is far slower than the formation rate of PtO*_x_* under anodic potential.

**Fig. 4.**
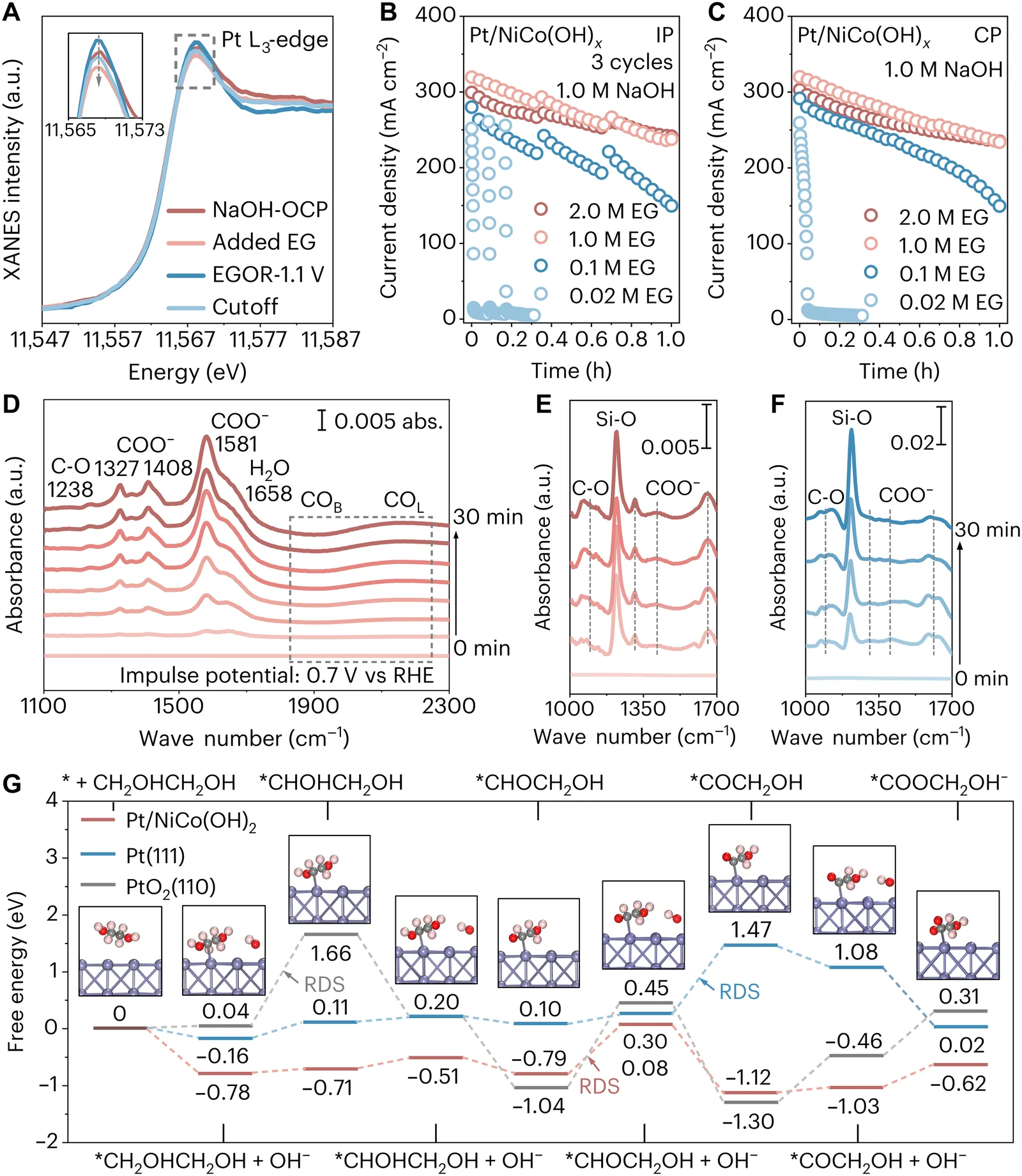
Catalytic mechanisms. (**A**) In situ normalized Pt L_3_-edge XANES spectra under different conditions. EGOR stability test of Pt/NiCo(OH)*_x_* through (**B**) IP (1.05 V versus RHE for 1200 s; 0.1 V versus RHE for 2 s; three cycles) and (**C**) CP (1.05 V versus RHE for 3600 s) modes under different concentrations of EG (0.1 liters). (**D**) In situ FTIR spectra (IRRAS model) of Pt/NiCo(OH)*_x_* for EGOR through IP methods in 1.0 M NaOH with 0.5 M EG. (**E**) In situ FTIR spectra (ATR-SEIRAS model) of Pt/NiCo(OH)*_x_* and (**F**) Pt for EGOR through IP methods in 1.0 M NaOH with 0.5 M EG. (**G**) Gibbs free energy of the EGOR stepwise pathway for Pt/NiCo(OH)_2_, Pt(111), and PtO_2_(110).

In situ FTIR spectra for EGOR (ATR-SEIRAS model for studying the surface adsorption of catalysts and IRRAS model for studying the intermediates of electrolyte; fig. S61) are used to probe critical intermediates. Apparently, the intermediates produced through IP and CP modes are extremely similar (Fig. 4D and fig. S62). The band at 1658 cm^−1^ is assigned to the bending vibration of H_2_O, and the band at 1238 cm^−1^ corresponded to the symmetric C─O stretching vibration of the formates, whereas the bands at 1327 and 1408 cm^−1^ are ascribed to the symmetric of COO^−^, and the band at 1581 cm^−1^ is attributed to the antisymmetric stretching vibrations of COO^−^ (*33*). Besides, no signal of bridge-bonded CO (CO_B_; ∼1850 cm^−1^) and linearly bonded CO (CO_L_; ∼2050 cm^−1^) means EGOR proceeds without carbonate intermediate (*34*). Pt/NiCo(OH)*_x_* has a stronger adsorption ability of the above intermediates than bare Pt (Fig. 4, E and F). In situ Raman spectra of Pt/NiCo(OH)*_x_* and Pt NPs/NF are investigated at the HER-relevant potential region (OCP to −0.12 V versus RHE) to reveal the HER mechanism (fig. S63). The peaks at 1608 and 2327 cm^−1^ are assigned to H─O─H bending mode (δ_H-O-H_) of interfacial H_2_O and Pt─H vibration mode (ν_Pt-H_), respectively (*35*, *36*). The much lower band of δ_H-O-H_ and higher band of ν_Pt-H_ indicate that nickel-cobalt hydroxides contribute to the adsorbed water dissociation and hydrogen adsorption.

Linear sweep voltammetry (LSV) curves under different concentrations of EG and NaOH demonstrate that enhanced EGOR activity relies on adequate concentrations of EG and OH^−^ (figs. S64 and S65), which is consistent with the fact that the alkaline alcohol oxidation generally demands sufficient OH^−^ to activate dehydrogenation for the formation of aldehyde (RCH_2_OH_ads_ + OH^−^ → RCHOH_ads_ + H_2_O) (*37*). Oxidation products of EGOR were qualitatively analyzed by ^1^H and ^13^C nuclear magnetic resonance (NMR) spectra (figs. S66 and S67); EG [3.5 parts per million (ppm)], the main product of sodium glycolate (3.8 ppm), and the by-product of sodium formate (8.3 ppm) were detected in the ^1^H NMR spectra, whereas sodium glycolate (61.3 ppm) and EG (62.8 ppm) were detected in the ^13^C NMR spectra (*38*, *39*). Among them, the by-product formation of formate is due to the further oxidation of glycolate. Concentrations of EG, glycolate and formate with faradaic efficiency and selectivity were quantificationally analyzed by high-performance liquid chromatography (HPLC; figs. S68 and S69). The highest faradaic efficiency (95.1%) with selectivity (96.7%) for glycolate was obtained at 1.05 V versus RHE (figs. S70 and S71). Notably, although the EGOR activity of Pt/NiCo(OH)*_x_* at the CP mode continuously decreases, the IP and CP modes still maintain similar faradaic efficiencies for glycolate, indicating that the formation of unfavorable PtO*_x_* species has nearly no negative effect on the selectivity of glycolate (fig. S72). In sum, the possible EGOR mechanism is shown in fig. S73. Initially, PtO*_x_* species are generated under applied potential. Subsequently, adsorbed EG molecules dehydrogenate to glycolaldehyde without the formation of *CO intermediate and reduce PtO*_x_* to active Pt sites. Last, glycolaldehyde is oxidized to glycolate under nucleophilic attack of vicinal hydroxyls and glycolate desorbs from Pt sites.

### Theoretical calculations

On the basis of the above analysis, Pt(111), PtO_2_(110), and an integrated model of Pt(111) with nickel-doped cobalt hydroxide [Pt/NiCo(OH)_2_] were constructed for density functional theory (DFT) calculations (fig. S74). The adsorption of all intermediates for HER and EGOR is assumed to occur at Pt sites. The Gibbs free energy of HER is probed along the pathway (* + H_2_O → *H + *OH → *H + OH^−^ → *H + H_2_O → * + H_2_ + OH^−^; fig. S75). The rate-determining step (RDS) for HER with the maximal energy barrier for Pt/NiCo(OH)_2_ is the H_2_ desorption step (0.57 eV), whereas the RDS for Pt(111) is the water dissociation (1.16 eV). As for EGOR, the charge density distributions and average Bader charges exhibit a notably enhanced charge density at regions between the adsorbed EG with Pt/NiCo(OH)_2_, suggesting EG-adsorbed Pt/NiCo(OH)_2_ has more free electrons to accelerate EG conversion (fig. S76). Furthermore, density of states (DOS) analysis demonstrates the total d-band center of Pt for Pt/NiCo(OH)_2_ (−2.20 eV) is closer to the Fermi level (0 eV) than that of Pt (−2.32 eV) and adsorbed PtO_2_ (−2.54 eV), which is beneficial for the efficient desorption of carbonyl intermediates (figs. S77 and S78) (*40*, *41*). The Gibbs free energy of EGOR is calculated through the pathway (* + CH_2_OHCH_2_OH → *CH_2_OHCH_2_OH + OH^−^ → *CHOHCH_2_OH → *CHOHCH_2_OH + OH^−^ → *CHOCH_2_OH → *CHOCH_2_OH + OH^−^ → *COCH_2_OH → *COCH_2_OH + OH^−^ → *COOCH_2_OH^−^; Fig. 4G). The RDS in EGOR with the maximal energy barrier for Pt/NiCo(OH)_2_ is the third dehydrogenation (0.87 eV), whereas the RDS for Pt is the forth dehydrogenation (1.17 eV) and the RDS for PtO_2_ is the first dehydrogenation (1.62 eV), indicating that Pt/NiCo(OH)_2_ owns the fastest reaction kinetics and the formation of PtO_2_ unfavorably delays EGOR. Furthermore, the above pathway involves EG and multiple hydroxyl adsorption, the higher EG adsorption energy and hydroxyl affinity of Pt/NiCo(OH)_2_ promote the intermediates conversion efficiency during EGOR (fig. S79). As expected, theoretical results reveal that PtO_2_ hampers EGOR and further confirm the importance of the above IP strategy for accelerating EGOR. Although nickel-cobalt species have negligible bifunctional activity at the working region of Pt sites, their derived hydroxides contribute to improving the adsorption of EG with hydroxyls and promoting water dissociation for accelerating reaction kinetics of both HER and EGOR.

### Electrosynthesis of glycolates

To establish the energy-saving system that benefits from the excellent bifunctional performance of Pt/NiCo(OH)*_x_*, we integrated the EGOR // HER two-electrode system that used Pt/NiCo(OH)*_x_* as both the cathode and the anode. Such an electrolyzer only requires voltages of 0.60 and 0.79 V to obtain current densities of 100 and 300 mA cm^−2^ (fig. S80). For comparison, the EGOR // HER system saves ∼63% energy consumption than the traditional OER // HER system without anodic oxygen generation and usage of expensive anion exchange membranes. As expected, the aforementioned cyclic IP strategy is also applicable to the two-electrode system (fig. S81).

To expand the practical application value, we used the hydrolysate of ground PET powders (1 μm in particle size, 100 mesh) from commercial water bottles as the electrolyte. Each gram of PET powder hydrolyzes into around 1.04 g of sodium terephthalates and 0.29 g of EG, whereas the conversion efficiency achieves nearly 100% and sodium terephthalates are almost precipitated in 10.0 M NaOH after 24 hours at 80°C (fig. S82). Impressively, the Pt/NiCo(OH)*_x_* catalyst still shows similar bifunctional activities in the PET hydrolysate (fig. S83). On the basis of the blocky character of a metal-foam catalyst, we used polymethyl methacrylate instead of expensive titanium alloy to design a mini-sized six-level slot-type flow electrolyzer (working area of 24 cm^2^) for driving an industrial-relevant current. Such a device has outstanding alkaline corrosion resistance and convenience to replace the catalyst (fig. S84). As shown in Fig. 5A, the PET hydrolysate electrolysis in the electrolyzer exhibits unprecedented low energy consumption, which only requires 1.18 V to drive an industrial-relevant current of 10 A (current density > 400 mA cm^−2^). Furthermore, such a flow electrolyzer even exhibits superior performance compared to a traditional membrane-free electrode assembly electrolyzer under the high-current-density working range (fig. S85). Notably, the large-batch PET hydrolysate electrolysis through the IP strategy durably processes for over 250 hours (Fig. 5B), which showcases the great promise for further large-scale application.

**Fig. 5.**
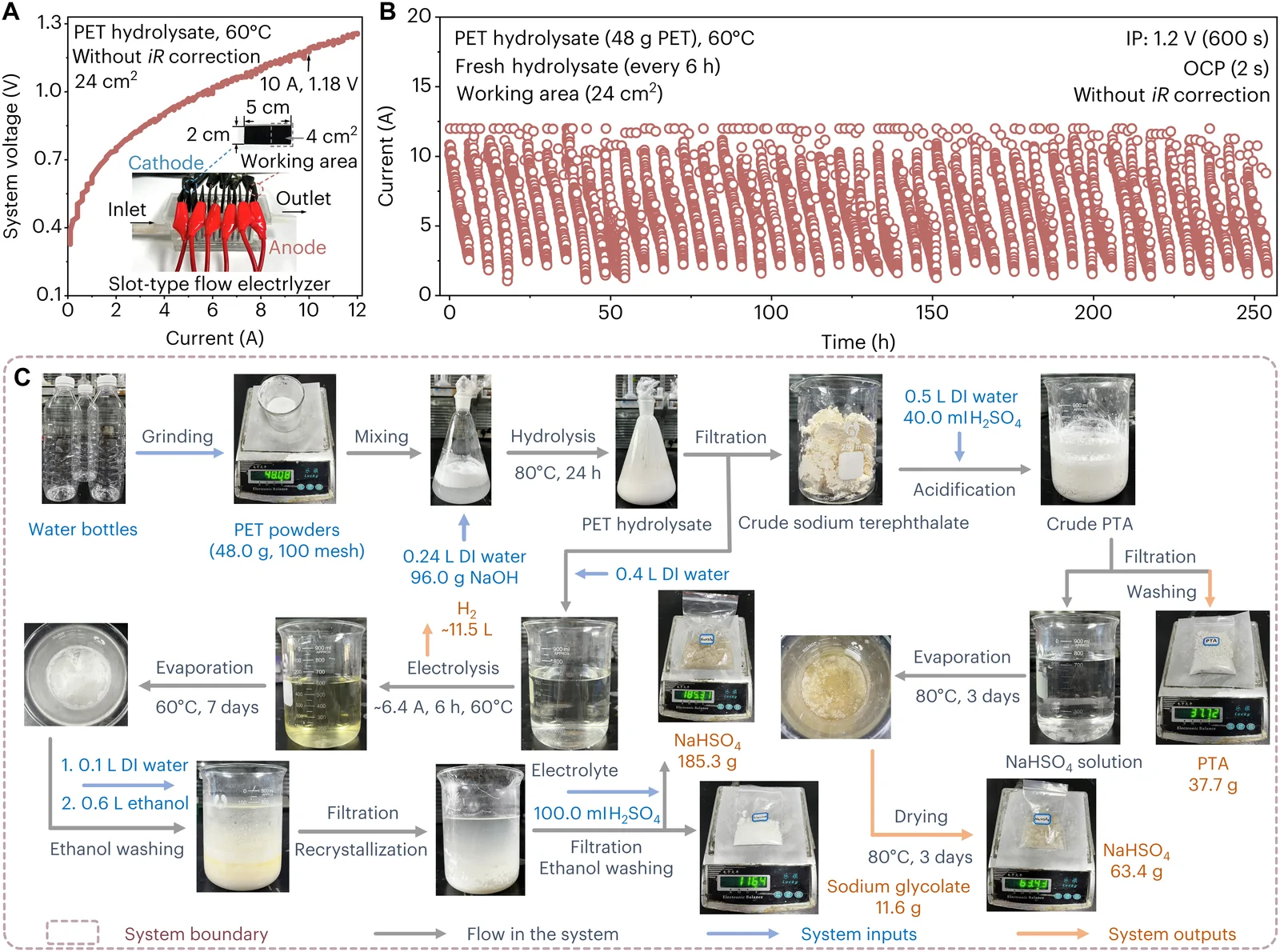
Device-level performance and technical route of PET electrochemical upgrading. (**A**) LSV curves for the PET hydrolysate electrolysis at a homemade flow electrolyzer with Pt/NiCo(OH)*_x_* electrodes. (**B**) Chronopotentiometry tests for the PET hydrolysate electrolysis at 1.2 V by IP method (replaced the hydrolysate with 48 g of PET powders every 6 hours). (**C**) Schematic illustration for the technical route of PET electrochemical upgrading.

### Product analysis of electrochemical PET upgrading

Designing the systematic technical route to achieve product separation is essential to maximize economical and engineering values. Glycolates can be inevitably oxidized to formates in the strongly basic environment under air; hence, recycling glycolates from an alkaline electrolyte in time is critical to reduce the product loss (fig. S86). Nevertheless, most existing researches ignore the further product separation from the electrolyte. As shown in Fig. 5C, we proposed the detailed technical route for the large-scale glycolate electrosynthesis through electrochemical PET upgrading. Sodium terephthalates of the PET hydrolysate under highly alkaline condition are initially precipitated after hydrolysis. Subsequent acidification with H_2_SO_4_ further transforms it into PTA precipitation, and NaHSO_4_ in the filtrate can be separated by the evaporative operation. EG of the PET hydrolysate is efficiently converted to glycolate with high selectivity after electrolysis under 1.2 V for 6 hours (fig. S87). Next, the evaporation of an electrolyte after electrolysis achieves the separation of unreacted EG from NaOH and glycolate. Last, taking advantages of the lower chemical polarity of ethanol than H_2_O and the lower solubility of glycolate than NaOH in ethanol, NaOH and glycolate are separated by multiple crystallization in the mixed solution of ethanol with H_2_O, whereas the remaining NaOH is also acidized by H_2_SO_4_ to obtain NaHSO_4_ or reused to PET hydrolysis. Impressively, we successfully produced glycolate (11.6 g, yield of 52.8%), along with PTA (37.7 g, yield of 95.8%), NaHSO_4_ (248.7 g, yield of 95.8%), and ∼11.5 liters of green hydrogen from 48.0 g of ground PET powders. XRD patterns and FTIR spectra of the products were compared with those of standard reagents to ensure desired purity (figs. S88 to S90). The PTA product can be used to reproduce PET and plasticizers, whereas the NaHSO_4_ product can be used to replace the highly corrosive acids for removing rust of devices and adjust the pH of wastewater. Therefore, such design owns great potential for the expansion from the laboratorial level to the industrial level (Fig. 6A). Compared with a recently reported alcohol oxidation–assisted system operating at an ampere-level current (Fig. 6B and table S4), the Pt/NiCo(OH)*_x_* assembled system occupies the leading position in the lowest operating voltage with optimal stability.

**Fig. 6.**
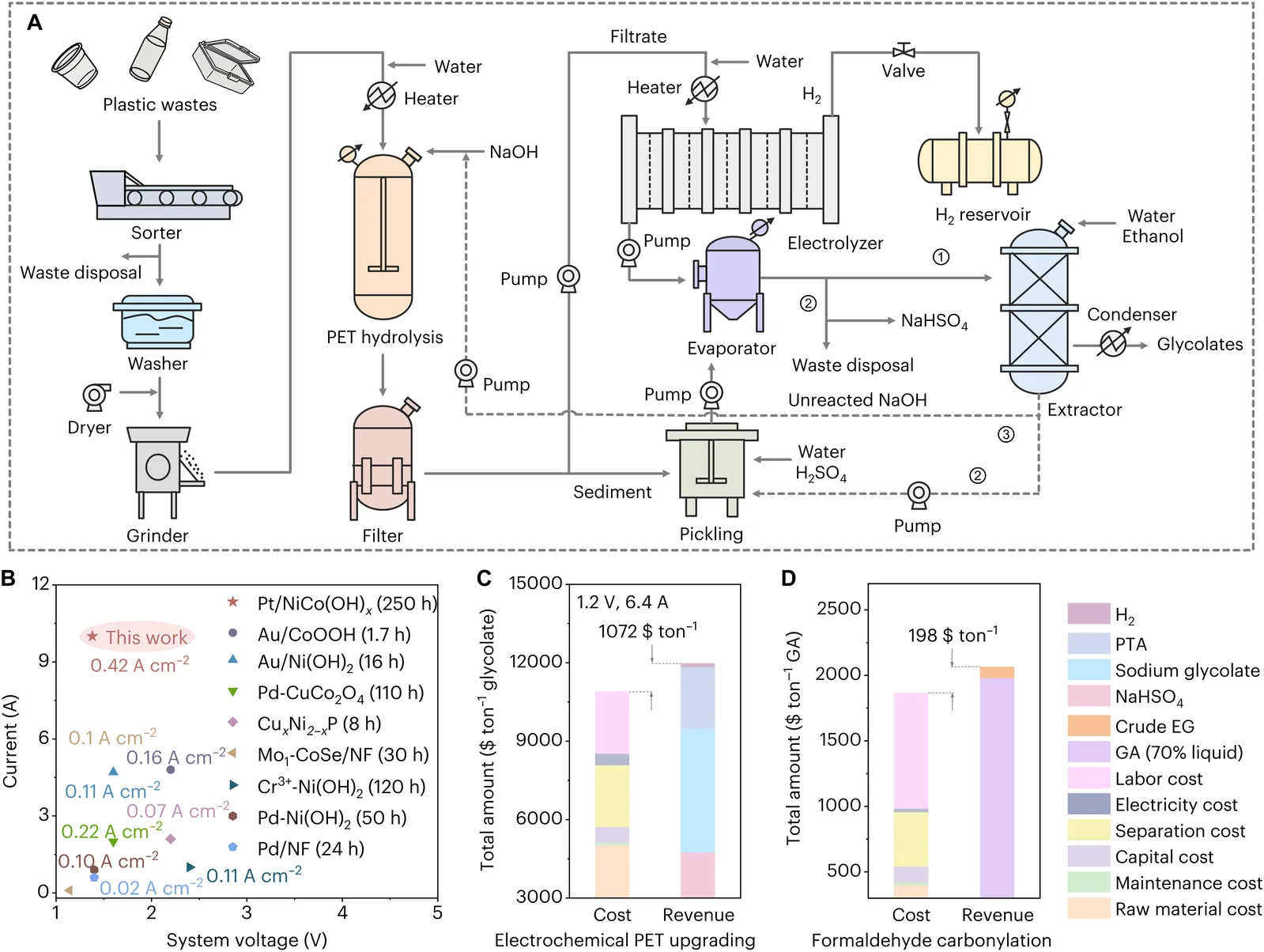
Analysis of practicability. (**A**) Schematic illustration of the technical route for the glycolate production through electrochemical PET upgrading. (**B**) Device-level performance comparison with recently reported alcohol oxidation–assisted systems. (**C**) Technoeconomic analysis of the glycolate production through electrochemical PET upgrading. (**D**) Technoeconomic analysis of the GA production through formaldehyde carbonylation.

The technoeconomic analysis indicates the highest spending of raw noble metal accounts of the total cost. Hence, the ideal total revenue is only obtained at the high ampere-level current region (fig. S91). Under the operating condition of this work (1.2 V with an average current of ∼6.4 A for each batch operation), the retained profit of ∼$1072 for producing per ton glycolate showcases the promising economic feasibility and precedes the current mainstream GA production route (Fig. 6, C and D, and table S5). Moreover, the Pt elements in the catalysts and the remaining electrolyte can be recycled through extra operations of acidification, extraction, and chemical reduction for further enhancing engineering values (fig. S92). These encouraging results highlight the pivotal role of the dynamic redox reactivation strategy for maximizing the stability of Pt-based catalysts and confirm the prospective application feasibility of the large-scale glycolate electrosynthesis from PET.

## DISCUSSION

The electrosynthesis of glycolates from waste PET enables the efficient cogeneration of diverse value-added chemicals while mitigating the global plastic crisis. However, its further deployment is severely hindered by the poor durability of available Pt-based catalysts under working potentials, which electrochemically oxidize active Pt sites into inert PtO*_x_* species. To address this, we have reported a dynamic redox reactivation strategy that alternates between the working potential for EGOR and the nonworking potential for reviving active Pt sites by the chemical reduction of adsorbed EG. With this approach, our self-supported Pt/NiCo(OH)_*x*_ catalyst achieves unprecedented durability for over 3000 hours at an average current density of ∼220 mA cm^−2^ while maintaining glycolate selectivity > 96%. An assembled electrolyzer delivered a record current of 10 A at 1.18 V, demonstrating impressive economic potential with an estimated revenue of ∼$1072 per ton of glycolate. This work paves the practical way for the large-scale electrosynthesis of glycolates from waste PET and holds great potential for decarbonizing the glycolate industry.

## MATERIALS AND METHODS

### Synthesis of NiCo(OH)_2_

A piece of NF (1.5 mm in thickness) was ultrasonically washed by 3 M HCl, ethanol, and deionized water for 20 min. Then, a mixture (30 ml) consisting of 0.04 M Co(NO_3_)_2_·6H_2_O, 0.12 M NH_4_F, and 0.33 M urea was stirred for 10 min in a Teflon-lined autoclave. Next, the cleaning NF was added into above solution and kept at 120°C for 3 hours. After naturally cooling down to room temperature, the obtained NiCo(OH)_2_ precursor was rinsed and dried in air.

### Synthesis of NiCoO*_x_*

The NiCo(OH)_2_ precursor was annealed at 400°C for 2 hours with a ramp rate of 5°C min^−1^ under the H_2_/Ar (10:90, 100 standard cubic centimeter per minute) atmosphere to synthesis NiCoO*_x_*. The NiCoO*_x_* after calcination should be stored in deionized water; otherwise, the green oxide layer will form after long-term exposure in air.

### Synthesis of Pt/NiCo(OH)*_x_*

The electrodeposition process was conducted under a three-electrode system, in which NiCoO*_x_* was used as the working electrode, the graphite rod was used as the counter electrode, and the Hg/HgO electrode was used as the reference electrode. The electrodeposition was carried out in 1.0 M KOH with 30 mg of 8 wt % H_2_PtCl_6_·6H_2_O solution (30 ml, 40°C) under stirring (300 rpm) by the CV method at a scan rate of 50 mV s^−1^ between 0 to −0.5 V versus RHE for 250 cycles (1-mV sample interval and 0.1 A V^−1^ sensitivity). Subsequently, the catalyst after electrodeposition was kept in the above electrolyte under stirring for 24 hours.

### Synthesis of Pt NPs/NF

The synthesis of Pt NPs/NF was similar to that of the Pt/NiCo(OH)*_x_* catalyst except for changing the substrate to cleaning NF for electrodeposition.

### Synthesis of 20 wt % Pt/C and PtO_2_ on NF

Typically, the mixture composed of 4.0 mg of commercial 20 wt % Pt/C powders or 1.5 mg of commercial PtO_2_ powders, 30 μl of Nafion, and 1.0 ml of isopropanol was ultrasonically treated at 20°C for 1 hour. Next, the above mixture was slowly dropped on the cleaning NF.

### Electrochemical measurement

Electrochemical measurements under a standard three-electrode system were conducted on the CHI 660E electrochemical workstation, in which the graphite rod was used as the counter electrode and the Hg/HgO electrode served as the reference electrode. The pH value of 1.0 M NaOH was measured to be 13.9. All potentials in this work were transferred to versus RHE according to the equation: *E*_RHE_ = *E*_Hg/HgO_ + 0.098 + 0.059 × pH. LSV curves were recorded under a scan rate of 2 mV s^−1^ with 85% *iR* correction to correct the voltage loss caused by the electrolyte solution, where the *R* was set to be 1.0 Ω. Electrochemical impedance spectra (EIS) were tested from 0.1 to 10,000 Hz with an amplitude of 5 mV. Long-term stability tests were conducted without *iR* correction, and ampere-level current tests were measured at the Neware BTS-5V12A power system.

### Flow electrolyzer fabrication

Stability tests for HER and EGOR under a three-electrode system were conducted in the self-made flow electrolyzer, and the electrolyte was circulated instead of outward between the three-electrode system with a 1.0-liter beaker at 32°C under stirring, and its flow rate was set to be 6.0 ml min^−1^ by the peristaltic pump. A 0.05-liter electrolyte was supplied to the 1-liter beaker per day according to the natural evaporation of the flow electrolyte. The slot-type six-unit flow electrolyzer with a height of 3.5 cm (the outlet is located at a height of 2.5 cm), a length of 6.0 cm, and a width of 3.1 cm was made of polymethyl methacrylate. Such an electrolyzer was connected to the power station through a pair of one-in and six-out copper clips. Scaled-up catalysts were directly inserted into slots with a spacing of 2.0 cm and a width of 3.0 cm. The flow rate of the PET hydrolysate was set to be 10.0 ml min^−1^ by the peristaltic pump at 60°C under stirring. In the nonworking state, 0.1 M EG aqueous solution is circulated in the electrolyzer to keep the stable state of catalysts.

### Characterizations

The morphology characterization was tested by a scanning electron microscope (Zeiss Gemini 300) and a transmission electron microscope (FEI Tecnai F20). Before the sample was used for TEM analysis, a piece of the sample was cut to an area of 0.2 cm^2^ and ultrasonically treated in 2.5 ml of ethanol for 30 s. Then, the suspension liquid was dropped to the ultrathin lacey carbon supported Cu grid for TEM testing. The crystal structure was tested by XRD (Bruker D8, Cu Kα x-ray source, 2° min^−1^). Valence states of elements were tested by XPS (ESCALAB 250, Al Kα x-ray source). FTIR spectra were tested in a Bruker Vertex 70, and Raman spectra (λ = 532 nm) were tested in Renishaw inVia. In situ FTIR and Raman characterizations were conducted in the in situ reaction device and controlled by the electrochemical workstation (CHI 660E). Before the test, the OCP was maintained under saturating of N_2_ for 1 hour to remove CO_2_ and O_2_ until a stable state and the background signal was subtracted. Specifically, the silicon crystal as the infrared window for in situ FTIR test (ATR-SEIRAS model) was first cleaned in aqua regia (20 min, *V*_HCl_:*V*_HNO3_ = 3:1), polished with Al_2_O_3_ powders (10 min, 50 nm), soaked in oxidation solution (20 min, *V*_H2SO4_:*V*_H2O2_ = 3:1), soaked in 40 wt % NH_4_F solution (5 min), and washed by deionized (DI) water. The gold coating operation on the silicon crystal required the preparation of a mixture of solution A (0.12 g of NaOH, 0.23 g of NaAuCl_4_·2H_2_O, and 3 ml of DI water) with solution B (0.13 g of NH_4_Cl, 0.95 g of Na_2_SO_3_, 0.62 g of NaS_2_O_3_·5H_2_O, and 50 ml of DI water). The cleaning silicon crystal was immersed into 15 ml of the above mixture at 55°C after adding 3.4 ml of 2 wt % HF solution. Last, the slurry of the catalyst was dropped on the gold film for testing. As for the in situ FTIR test (IRRAS model), the electrolyte layer between infrared windows of CaF_2_ with the catalyst on the gold electrode is thinner than 10 μm. The Pt mass loading was analyzed by ICP-OES, and leaching contents of Co and Pt were analyzed by inductively coupled plasma mass spectrometry (ICP-MS) in Agilent 7800. Products of EGOR were qualitatively analyzed by NMR (500 MHz) in Bruker Advance 400, in which 500 μl of the electrolyte was mixed with 500 μl of D_2_O and 30 μl of dimethyl sulfoxide. Products of EGOR were quantitatively analyzed by HPLC in Agilent 1260 through a chromatographic column (Xtimate Sugar-H) and a mobile phase of 25 mM H_2_SO_4_ (60°C, 0.6 ml min^−1^).

## Supplementary Materials

### This PDF file includes:

Supplementary Text Figs. S1 to S92 Tables S1 to S5 References
