# Supplementary material for: Dynamic redox reactivation of platinum sites for long-term glycolate electrosynthesis from polyethylene terephthalate wastes
Source: Sci Adv. 2026 Nov 4;12(45):eaei5545. doi: 10.1126/sciadv.aei5545 (PMC13733892; doi:10.1126/sciadv.aei5545)
Supplement: Supplementary file 1 [file sciadv.aei5545_sm.pdf]

Science Advances
AAAS

Supplementary Materials for

Dynamic redox reactivation of platinum sites for long-term glycolate electrosynthesis from polyethylene terephthalate wastes

Zehao Xiao *et al.*

Corresponding author: Mingchuan Luo, m.luo@pku.edu.cn; Shaojun Guo, guosj@pku.edu.cn

Sci. Adv. **12**, eaei5545 (2026)
DOI: 10.1126/sciadv.aei5545

This PDF file includes:

Supplementary Text
Figs. S1 to S92
Tables S1 to S5
References

Supplementary Text

Chemicals. All chemicals in this work were used without further purification. $Co(NO_3)_2 \cdot 6H_2O$, $H_2PtCl_6 \cdot 6H_2O$, hydrochloric acid (36.5 wt%), NaOH, NH_4F, urea ($CO(NH_2)_2$), ethylene glycol ($C_2H_6O_2$), formic acid (CH_2O_2, 88%), glycolic acid ($C_2H_4O_3$) and sodium glycolate were purchased from Sinopharm Chemical Reagent Co., Ltd. in China. Commercial 20 wt% Pt/C and PtO_2 catalysts were purchased from Alab (Shanghai) Chemical Technology Co., Ltd. in China. Nickel foams (NF, 1.5 mm in thick, 350 $g \cdot cm^{-2}$ in areal density) were purchased from Kunshan Lvchuang Technology Co., Ltd. in China. De-ionized (DI, 18.2 $\Omega \cdot cm^{-1}$) water was used in all experiments. Commercial mineral-water bottles were ground to powders of 100 mesh.

Product analysis. The Faradaic efficiency (FE) of product is calculated from the corresponding electron transfer at per reaction through the following equation:

$$FE = 100\% \times \frac{n_{product} \times n_e}{Q} \times F \qquad (S1)$$

Where n_e is the number of transferred electron, $n_{product}$ is the amount of product, F is the Faraday constant of 96,485 $C \cdot mol^{-1}$ and Q is the amount of electric charge.

The selectivity of product is calculated through the following equation:

$$Selectivity = 100\% \times \frac{n_{product}}{n_{total}} \qquad (S2)$$

Where n_{total} is the amount of all products in per reaction.

Electrochemical active surface area (ECSA) calculation. The ECSA of Pt sites for Pt-based catalysts are calculated from the following equation:

$$ECSA_{Pt,cat}(cm^2 \cdot mg_{Pt}^{-1}) = \frac{Q_{H-adsorption}}{210\ \mu C \times cm_{Pt}^{-2} \times L_{Pt}(mg_{Pt} \cdot cm^{-2}) \times S} \qquad (S3)$$

Where $Q_{H\text{-}adsorption}$ is the integral charge of the oxidation peak at the potential region from 0.05 to 0.4 V *vs.* RHE, L_{Pt} is the mass loading of Pt and S is the geometric area of the electrode.*(42)*

Turnover frequency (TOF) calculation. TOF relates to the reactants of per reaction at *per* active site *per* time. TOF at per active site for per reaction is calculated through the following equation:

$$\text{TOF} = \frac{|j| \times N_A \times S \times (1\ C \cdot s^{-1})}{n_e \times n \times F} \qquad (S4)$$

Where N_A is the Avogadro constant of 6.023×10^{23} ($molecules \cdot mol^{-1}$), n_e is the amount of charge transfer for each reaction, $|j|$ is the absolute value of the current density ($A \cdot cm^{-2}$) at a given potential, F is the Faraday constant of 96,485 $C \cdot mol^{-1}$ and n is the amount of active sites.

The amount of active sites for per reaction is calculated through the following equation:

$$n = S \times ECSA \times n_s \quad (S5)$$

Where S is the geometric area of the electrode and n_s is a surface site density close to 2 × 10^{15} sites·cm^{-2} of most materials.*(43, 44)*

DFT calculation details. All spin-polarized density functional theory (DFT) methods were conducted through the Vienna ab-initio simulation package (VASP) code by the projector augmented wave (PAW).*(45, 46)* The generalized gradient approximation (GGA) with Perdew-Burke-Ernzerhof (PBE) functional formulations were used to analyse the exchange-correlation term.*(47)* The PAW pseudo-potentials were used to analyse ionic cores. The cut-off energy for the plane-wave basis was 450 eV. The Van der Waals (VDW) interactions were analysed through the empirical correction in Grimme's scheme (DFT-D3).*(48)* The convergence tolerances for energy and force were 10^{-5} eV and 0.05 eV·$Å^{-1}$.

The adsorption energy (E_{ads}) is calculated from the following equation:

$$E_{ads} = E_{ad/sub} - E_{ad} - E_{sub} \quad (S6)$$

Where $E_{ad/sub}$ is the total energy of the optimized adsorbate/substrate system, E_{ad} is the total energy of the adsorbate in the structure and E_{sub} is the energy of the substrate.

The Gibbs free energy (ΔG) is calculated from the following equation:

$$\Delta G = \Delta E_{DFT} + \Delta E_{ZPE} - T \times \Delta S \quad (S7)$$

Where ΔE_{DFT} is the electronic energy change from DFT calculations, ΔE_{ZPE} is the zero-point energy and ΔS is the entropy change from frequency calculations at 298.15 K.

Extended X-ray absorption fine structure (EXAFS) analysis. All EXAFS data (transmission mode) were conducted at the BL11B beamline of the Shanghai synchrotron radiation facility. All EXAFS data were processed in the Athena software and simulations of EXAFS data were fitted in the Artemis software. The variables of data simulations are defined as follows:

CN: coordination number; R: distance between the absorber and backscatter atoms; σ^2: Debye-Waller factor; ΔE_0: inner potential correction. R-factor: indicator of goodness of fit.

Error bounds (accuracies) of the structural parameters for data simulations are estimated as follows:

N: ±20%; R: ±5%; σ^2: ±20%; and ΔE_0: ±20%.

Products separation. The hydrolysis of ground PET powders was conducted at 80 °C for 24 h in the conical flask containing 48.0 grams of PET powders, 2.4 mol NaOH and 0.24 L DI water with stirring. Subsequently, sodium terephthalate precipitations with a small quantity of NaOH were filtrated and acidized by 40 mL H_2SO_4 to adjust pH to 1.0 with stirring. After the temperature was cooled down, PTA precipitations were filtrated and washed by DI water for several times, while the filtrate was concentrated under 80 °C with stirring for 5 days until crystals precipitated in the small amount of remaining solution (unreacted H_2SO_4). Afterwards, the separated crystals were dried at 80 °C for 3 days under vacuum to separate $NaHSO_4$. Electrolyte containing EG after

electrolysis was evaporated under vacuum to dryness at 60 °C for 7 days. After the remaining solid was completely dissolved in 0.1 L DI water under ultrasonically treatment for 1 h, 0.6 L ethanol was added and the solution was crystallized at 10 °C for 12 h, such crystallization operation was repeated for 3 times. Then, the separated filtrate was acidized by adding 100 mL H_2SO_4 to recycle unreacted NaOH.

Economic model of techno-economic analysis for glycolate production through electrochemical PET upgrading. The assumptive total amount of disposing waste plastics is set to be 3,000 tons (350 working days·year^{-1}). The current on the each six-level slot-type flow electrolyzer is *I* A. According to the estimated Faradaic efficiency (95%) of EGOR and overall energy efficiency (60%), total charge and required reaction area are calculated as following:

$$Total\ charge = \frac{3{,}000{,}000\ kg \times 29\%}{62.068 \times 10^{-3}\ kg \cdot mol^{-1}} \times 4\ e^{-} \times \frac{96{,}485\ C}{mol} \times \frac{1}{0.95} = 5.69 \times 10^{12}\ C$$

$$Total\ current = \frac{5.69 \times 10^{12}\ C}{60\% \times 350 \times 24 \times 3600\ s} = 313602.30\ \text{A}$$

Reaction area is calculated as following:

$$Required\ reactor\ aomount = \frac{313602.30}{I}$$

$$Reaction\ area = \frac{313602.30}{I} \times 24 \times 10^{-4}\ m^2 = \frac{752.65}{I}\ m^2$$

Raw material cost. Assuming that anodic/cathodic catalysts are replaced every 25 days. Market sale prices of $Co(NO_3)_2 \cdot 6H_2O$, NH_4F, urea, $H_2PtCl_6 \cdot 6H_2O$, H_2/Ar (10 : 90), NaOH and H_2SO_4 are 7.26 *USD·kg*$^{-1}$, 0.96 *USD·kg*$^{-1}$, 0.32 *USD·kg*$^{-1}$, 15.69 *USD·g*$^{-1}$, 1.05 *USD·kg*$^{-1}$, 0.15 *USD·kg*$^{-1}$ and 0.04 *USD·kg*$^{-1}$, respectively. Assuming that the quality of recycled PET is reduced, the sale price of recycled PET is set to be 80% of the market price (0.31 *USD·kg*$^{-1}$). Raw material cost is calculated as following:

$$Co(NO_3)_2\ cost = 2 \times \frac{752.65}{I}\ m^2 \times \frac{7.26\ USD}{1000\ g} \times \frac{0.349\ g}{0.0004\ m^2} \times 14 = \frac{133491.36}{I}\ USD$$

$$NH_4F\ cost = 2 \times \frac{752.65}{I}\ m^2 \times \frac{0.96\ USD}{1000\ g} \times \frac{0.133\ g}{0.0004\ m^2} \times 14 = \frac{6726.89}{I}\ USD$$

$$Urea\ cost = 2 \times \frac{752.65}{I}\ m^2 \times \frac{0.32\ USD}{1000\ g} \times \frac{0.6\ g}{0.0004\ m^2} \times 14 = \frac{10115.62}{I}\ USD$$

$$H_2\text{Pt}Cl_6 \cdot 6H_2\text{O}\ cost = 2 \times \frac{752.65}{I}\ m^2 \times \frac{15.69\ USD}{1\ g} \times \frac{0.0024\ g}{0.0001\ m^2} \times 14 = \frac{7935700.76}{I}\ USD$$

$$H_2/Ar\ cost = \frac{752.65}{I} m^2 \times 14 \times \frac{1800\ L}{0.5\ m^2 \times 26.5\ L \cdot mol^{-1}} \times \frac{33.4\ g}{1\ mol} \times \frac{1.05\ USD}{1000\ g} = \frac{50201.14}{I}\ USD$$

$$NaOH\ cost = 3{,}000{,}000\ kg \times \frac{96\ g}{48\ g} \times \frac{0.15\ USD}{1000\ g} = 9.00 \times 10^5\ USD$$

$$H_2SO_4\ cost = 140\ mL \times \frac{3{,}000{,}000\ kg}{48\ g} \times \frac{1.84\ g}{1\ mL} \times \frac{0.04\ USD}{1000\ g} = 6.44 \times 10^5\ USD$$

$$Waste\ PET\ cost = 3{,}000{,}000\ kg \times 80\% \times \frac{0.31\ USD}{1000\ g} = 7.44 \times 10^5\ USD$$

$$Raw\ material\ cost = (2.30 \times 10^6 + \frac{8136235.77}{I})\ USD$$

Total revenue. Assuming that the Faradaic efficiency of HER is 98%, yield rate of PTA is 95%, yield rate of $NaHSO_4$ is 95% and yield rate of sodium glycolate is 52%. Market sale prices of $NaHSO_4$, sodium glycolate, PTA and H_2 are 0.22 $USD \cdot kg^{-1}$, 4.77 $USD \cdot kg^{-1}$, 0.71 $USD \cdot kg^{-1}$ and 1.90 $USD \cdot kg^{-1}$. Total product revenue is calculated as following:

$$NaHSO_4\ revenue = 3{,}000{,}000\ kg \times 90\% \times \frac{96\ g}{48\ g} \times \frac{120.06\ g \cdot mol^{-1}}{40.00\ g \cdot mol^{-1}} \times \frac{0.22\ USD}{1000\ g} \times 95\% = 3.38 \times 10^6\ USD$$

$$Sodium\ glycolate\ revenue = 3{,}000{,}000\ kg \times 29\% \times 52\% \times \frac{98.03\ g \cdot mol^{-1}}{62.07\ g \cdot mol^{-1}} \times \frac{4.77\ USD}{1000\ g} = 3.41 \times 10^6\ USD$$

$$PTA\ revenue = 3{,}000{,}000\ kg \times 82\% \times 95\% \times \frac{0.71\ USD}{1000\ g} = 1.66 \times 10^6\ USD$$

$$H_2\ revenue = \frac{5.69 \times 10^{12}\ C}{2 \times 96{,}485\ C \cdot mol^{-1}} \times 2\ g \cdot mol^{-1} \times \frac{1.9\ USD}{1000\ g} \times 0.98 = 1.09 \times 10^5\ USD$$

$$Total\ revenue = 8.55 \times 10^6\ USD$$

Capital cost. In this work, we used polymethyl methacrylate as the raw material for designing the electrolyzer and its stack cost is set to be 20% of titanium alloy (460 *USD/kW*). The balance of plant (BoP) cost is assumed to be 35% of the electrolyzer cost, and these values are calculated from the H_2A model.*(49)* The voltage on each six-level slot-type flow electrolyzer is *U* V.

$$Electrolyzer\ cost = \frac{92\ USD}{1000\ W} \times 313602.30\ A \times U\ V = (28851.42 \times U)\ USD$$

$$BoP\ cost = 28851.42 \times U\ USD \times \frac{0.35}{0.65} = (15535.38 \times U)\ USD$$

PSA cost relates to the hydrogen gas flow rate, which is calculated as following:

$$Volume\ of\ H_2 = \frac{5.69 \times 10^{12}\ C}{2 \times 96{,}485\ C \cdot mol^{-1}} \times 26.5\ L \cdot mol^{-1} \times 0.98 = 7.66 \times 10^5\ m^3$$

$$PSA\ cost = 1{,}989{,}043\ USD \times (\frac{7.66 \times 10^5\ m^3}{350 \times 24\ h \times 10^3\ m^3 \cdot h^{-1}})^{0.7} = 3.73 \times 10^5\ USD$$

$$Captial\ cost = (3.73 \times 10^5 + 44386.80 \times U)\ \mathrm{USD}$$

Operating cost. Assuming that the cost of industrial electricity (Beijing, China) is 0.05 *USD·(kW·h)*$^{-1}$ and the total electricity consumption of the whole technology is twice the electrolyzer consumption. Electricity cost is calculated as following:

$$Electricity\ cost = 2 \times 313602.30\ A \times U\ V \times 350 \times 24\ h \times \frac{0.05\ USD}{1000\ W \cdot h}$$
$$= (263425.94 \times U)\ USD$$

Assuming that the working and maintenance cost is 20% of the capital cost.

$$Working\ and\ maintenance\ cost = (3.73 \times 10^5 + 44386.80 \times U) \times 20\%$$
$$= (7.46 \times 10^4 + 8877.36 \times U)\ USD$$

Assuming that hydrogen as the only product of the cathode without separation operation, solid products need to be separated with 20% cost of revenues for solid products.

$$Separation\ cost = 8.45 \times 10^6 \times 20\% = 1.69 \times 10^6\ USD$$

$$Operating\ cost = (1.77 \times 10^6 + 272303.30 \times U)\ USD$$

Other cost. Other cost includes pretreatment costs, water costs, labor costs, transportation costs, management costs, and sales costs and so on. The value of other cost is set to be 20% of the total revenue.

$$Other\ cost = 8.55 \times 10^6 \times 20\% = 1.71 \times 10^6\ USD$$

Retained profit. Retained profit for glycolate production through electrochemical PET upgrading is calculated as following:

$$Total\ cost = 2.30 \times 10^6 + \frac{8136235.77}{I} + 3.73 \times 10^5 + 44386.80 \times U + 1.77 \times 10^6$$
$$+ 272303.30 \times U + 1.71 \times 10^6$$
$$= \left(6.16 \times 10^6 + 316690.10 \times U + \frac{8136235.77}{I}\right) USD$$

$$Retained\ profit\ (glycolate) = \frac{Total\ revenue - Total\ cost}{3{,}000\ tons \times 29\% \times 52\% \times \frac{98.03\ g \cdot mol^{-1}}{62.07\ g \cdot mol^{-1}}}$$
$$= \left(3345.01 - 443.24 \times U - \frac{11387.38}{I}\right)\ USD \cdot ton^{-1}$$

Economic model of techno-economic analysis for GA production through formaldehyde carbonylation process. Such technology uses formaldehyde from syngas, carbon monoxide and water as raw materials to synthesize GA under high temperature, high pressure and acid catalyst conditions, which is the mainstream technology in the world.*(7)* According to the current market situation, the production of GA from syngas possesses greater cost advantage and better economic benefit. Most of the economic analysis in this section refers to the cost estimate of GA production given by the Huajing Economic Research Institute (www.huaon.com).

Raw material cost. Per ton of GA production require CO (791.3 $Nm^3 \cdot ton^{-1}$), H_2 (976.7 $Nm^3 \cdot ton^{-1}$), O_2 (197.8 $Nm^3 \cdot ton^{-1}$), methanol (70 $kg \cdot ton^{-1}$), 68 wt% nitric acid (*7* $kg \cdot ton^{-1}$) and catalyst (30 $USD \cdot ton^{-1}$). Raw material cost is calculated as following:

$$\begin{aligned} &Raw\ material\ cost \\ &= 791.3\ Nm^3 \times \frac{0.22\ USD}{Nm^3} + 976.7\ Nm^3 \times \frac{0.16\ USD}{Nm^3} + 197.8\ Nm^3 \\ &\times \frac{0.07\ USD}{Nm^3} + 70\ kg \times \frac{0.31\ USD}{1\ kg} + 7\ kg \times \frac{0.18\ USD}{1\ kg} + 30\ USD \\ &= 397.16\ USD \end{aligned}$$

Total revenue. The main products include GA (70% liquid) and crude ethylene glycol (EG). For the production of *per* ton GA (70% liquid), 180 *kg* crude EG are obtained as by-product. Market sale prices of GA (70% liquid) and crude EG are 1.98 $USD \cdot kg^{-1}$ and 0.48 $USD \cdot kg^{-1}$. Total product revenue is calculated as following:

$$GA\ (70\%\ liquid)\ revenue = 1000\ kg \times \frac{1.98\ USD}{1\ kg} = 1980\ USD$$

$$Crude\ EG\ revenue = 180\ kg \times \frac{0.48\ USD}{1\ kg} = 86.4\ USD$$

$$Total\ revenue = 2066.4\ USD$$

Capital cost. Such technology requires high pressure and strong acid condition, which lead to serious corrosion and high requirements for equipment. Capital cost includes administration cost and depreciation cost. Capital cost is calculated as following:

$$\begin{aligned} Captial\ cost &= Administration\ cost + Depreciation\ cost = 13.66\ USD + 109.28\ USD \\ &= 122.94\ USD \end{aligned}$$

Operating cost. Operating cost includes separation cost, electricity cost, steam heating cost, working and maintenance cost. Operating cost is calculated as following:

$$
\begin{aligned}
&Operating\ cost \\
&\quad = Separation\ cost + Electricity\ cost + Steam\ heating\ cost \\
&\quad + Working\ and\ maintenance\ cost \\
&\quad = 2066.4\ \times 20\%\ USD + 800\ kW \cdot h \times \frac{0.05\ USD}{1000\ W \cdot h} + 5000\ kg\ \times \frac{0.02\ USD}{1000\ g} \\
&\quad + 21.86\ USD = 935.14\ USD
\end{aligned}
$$

Other cost. Other cost includes pretreatment costs, water costs, labor costs, transportation costs, management costs, and sales costs and so on. The value of other cost is set to be 20% of the total revenue.

$$Other\ cost = 2066.4\ \times 20\%\ = 413.28\ USD$$

Retained profit. Retained profit for GA production through formaldehyde carbonylation process is calculated as following:

$$Retained\ profit = Total\ revenue - Total\ cost = 197.88\ USD \cdot ton^{-1}$$

Supplementary Figures

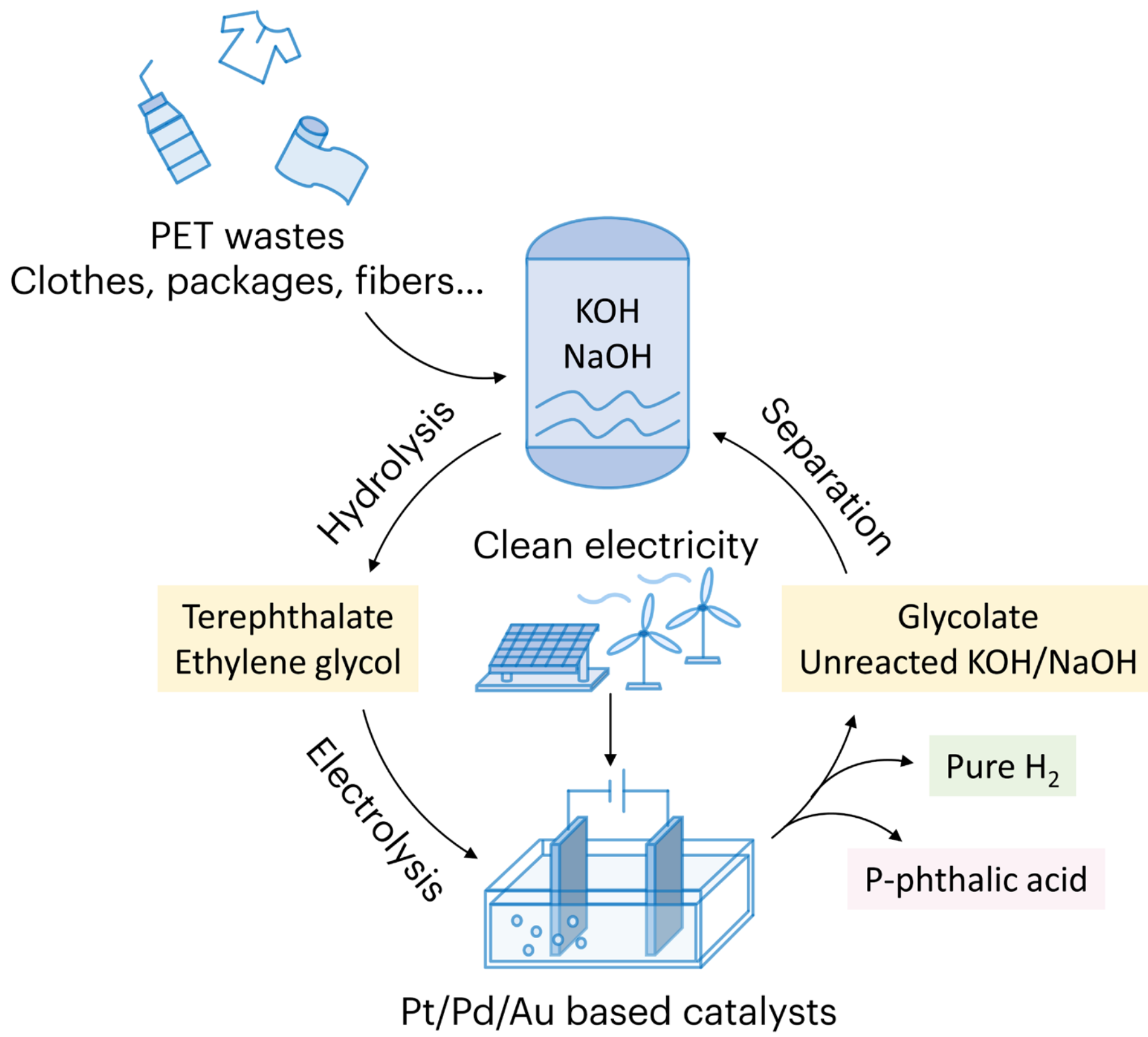

Fig. S1. Technical schematic diagram. Schematic diagram for the synthesis of glycolates through electrochemical PET upgrading strategy.

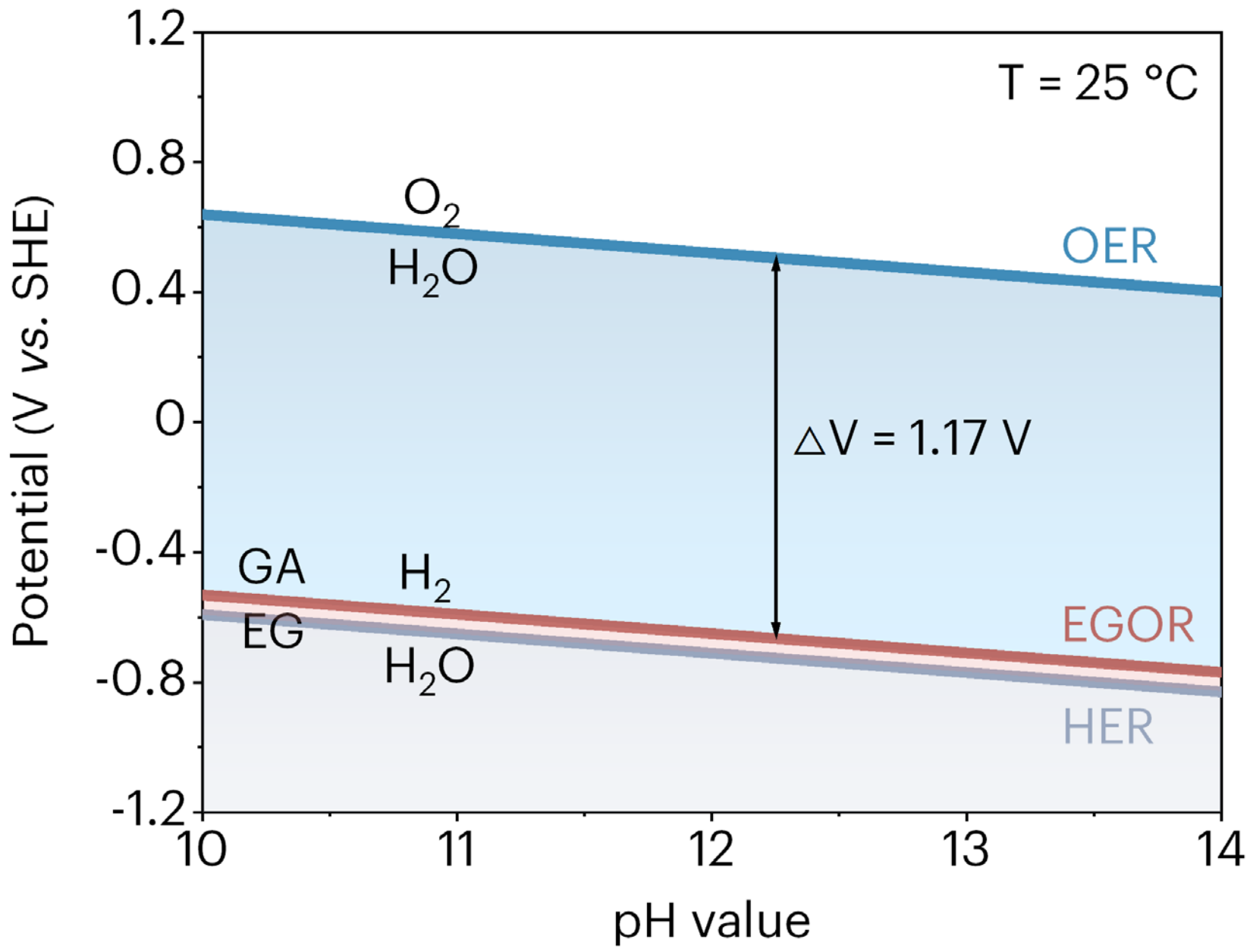

Fig. S2. Theoretical analysis. Pourbaix *E*-pH diagram of OER, EGOR (EG to GA) and HER under different pH values.*(50)*

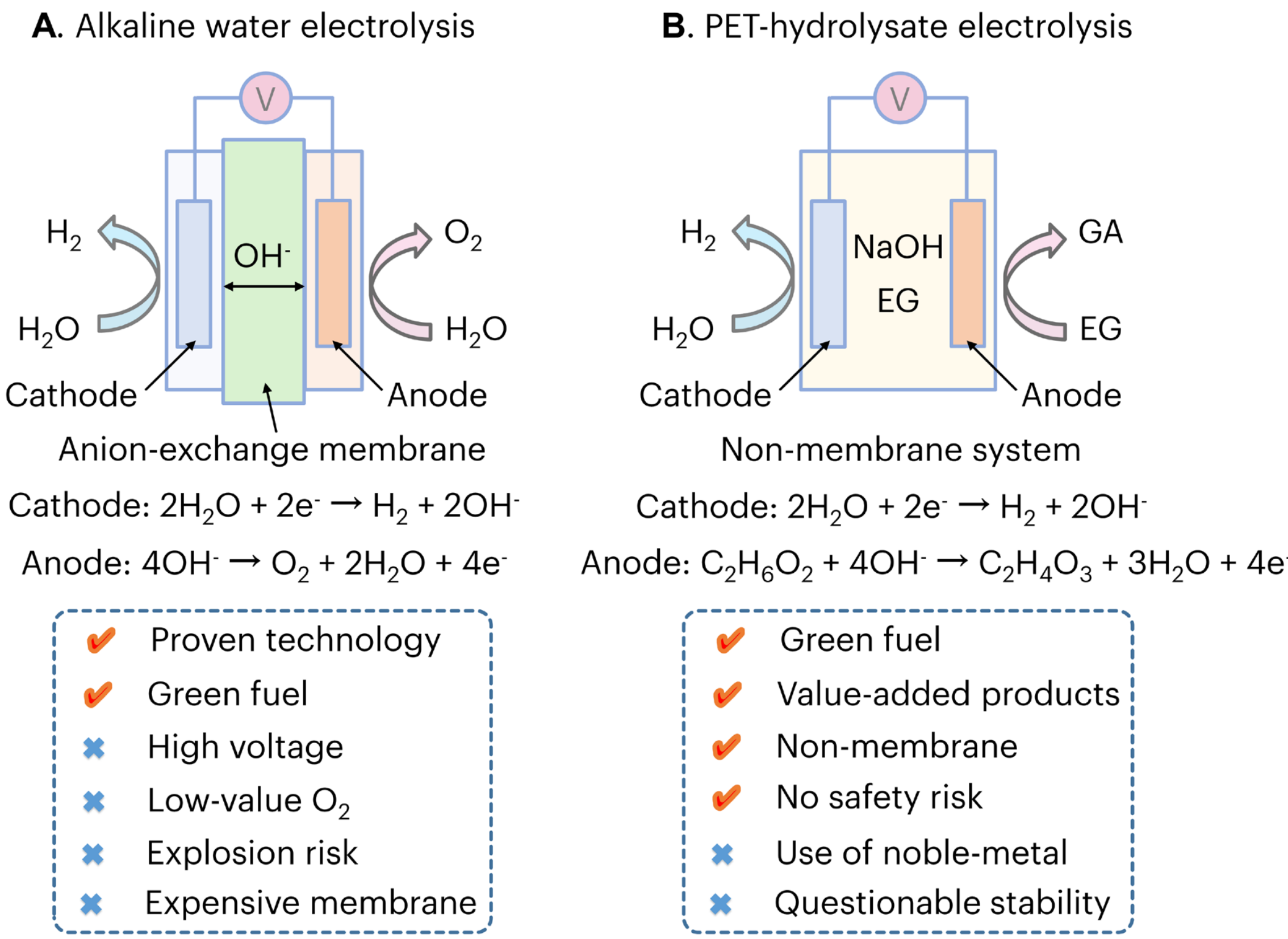

Fig. S3. Technical schematic diagram. Schematic diagrams of (**A**) traditional alkaline water electrolysis system and (**B**) hybrid PET-hydrolysate electrolysis system.

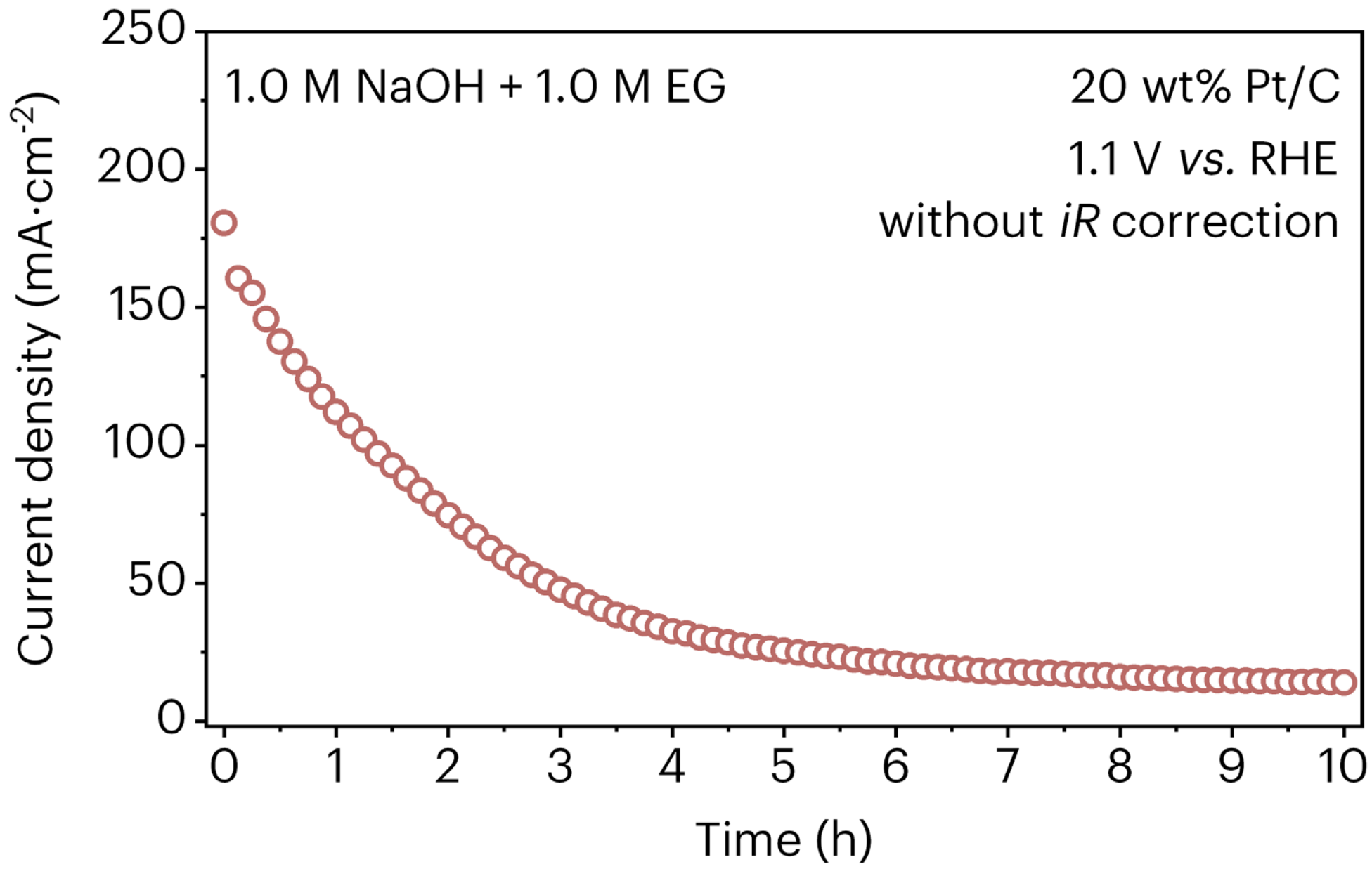

Fig. S4. Electrochemical performance. EGOR stability test of 20 wt% Pt/C at 1.1 V *versus* the reversible hydrogen electrode (*vs.* RHE) in the electrolyte of 1.0 M NaOH + 1.0 M EG.

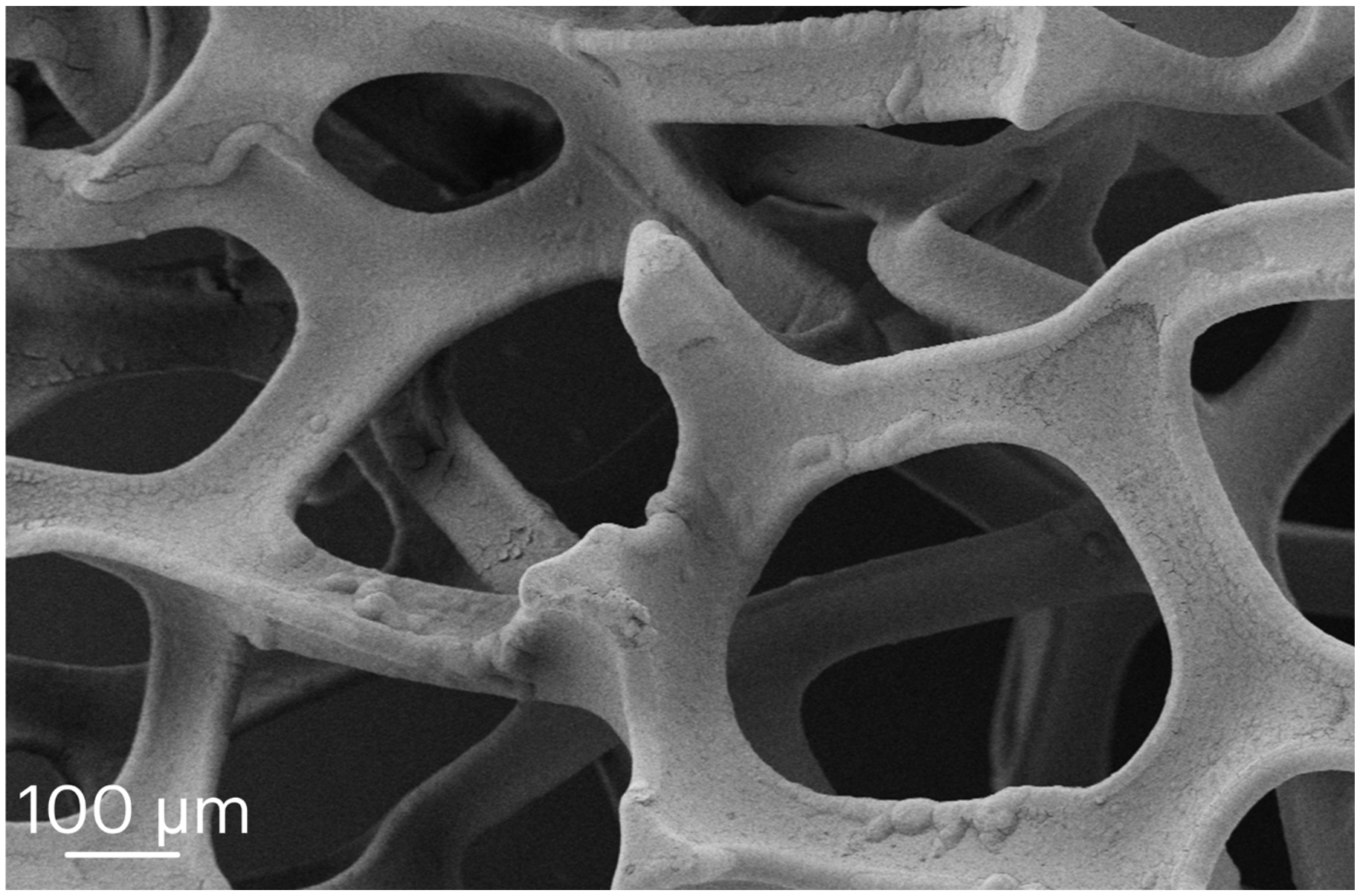

Fig. S5. Structural analysis. SEM image of the bare NF.

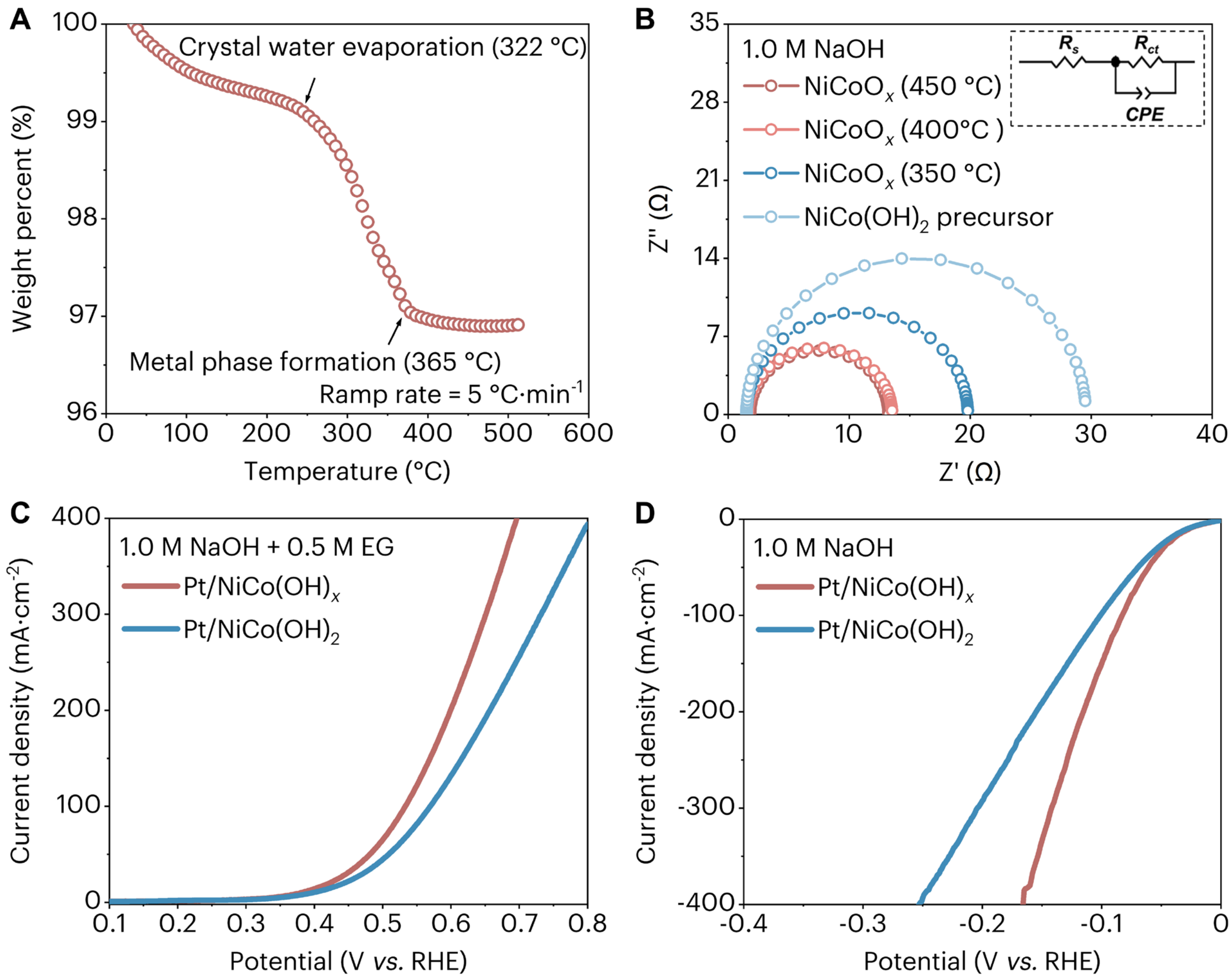

Fig. S6. Structural and impedance analysis. (**A**) Thermal gravimetric analysis curve for the annealing reduction process of NiCo(OH)$_2$. (**B**) Electrochemical impedance spectroscopy (EIS) plots of NiCo(OH)$_2$ and NiCoO$_x$ under different annealing temperatures at -0.05 V *vs.* RHE. *Inset* shows the fitting circuit diagram. (**C**) EGOR linear sweep voltammetry (LSV) curves of Pt/NiCo(OH)$_x$ and Pt/NiCo(OH)$_2$. (**D**) HER LSV curves of Pt/NiCo(OH)$_x$ and Pt/NiCo(OH)$_2$.

Note: The Pt/NiCo(OH)$_2$ catalyst was synthesized by the same electro-deposition of Pt nanoparticles (NPs) as Pt/NiCo(OH)$_x$ on the NiCo(OH)$_2$ precursor.

.

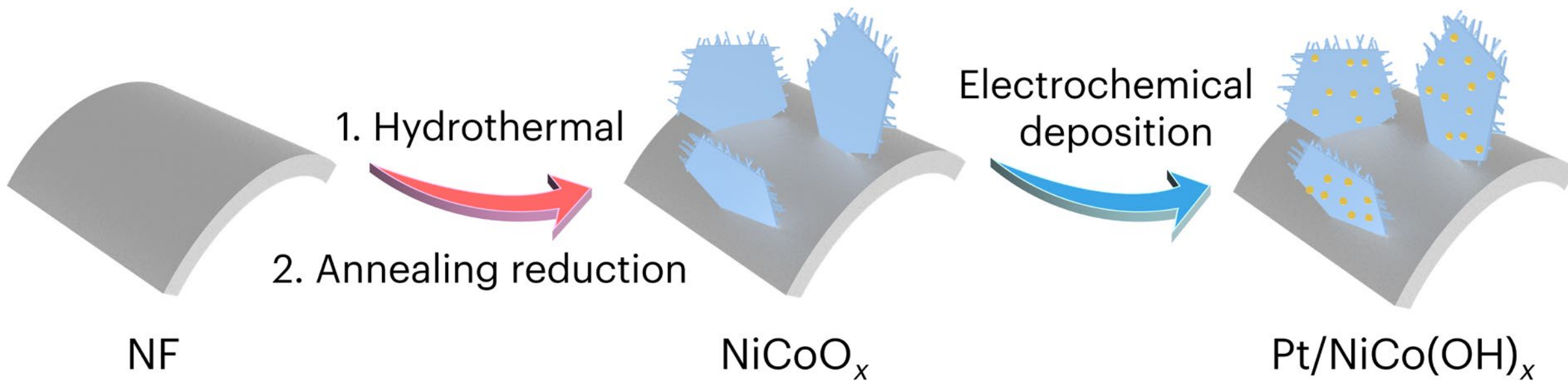

Fig. S7. Synthesis scheme. Schematic illustration of the synthetic process for the Pt/NiCo(OH)$_x$ catalyst.

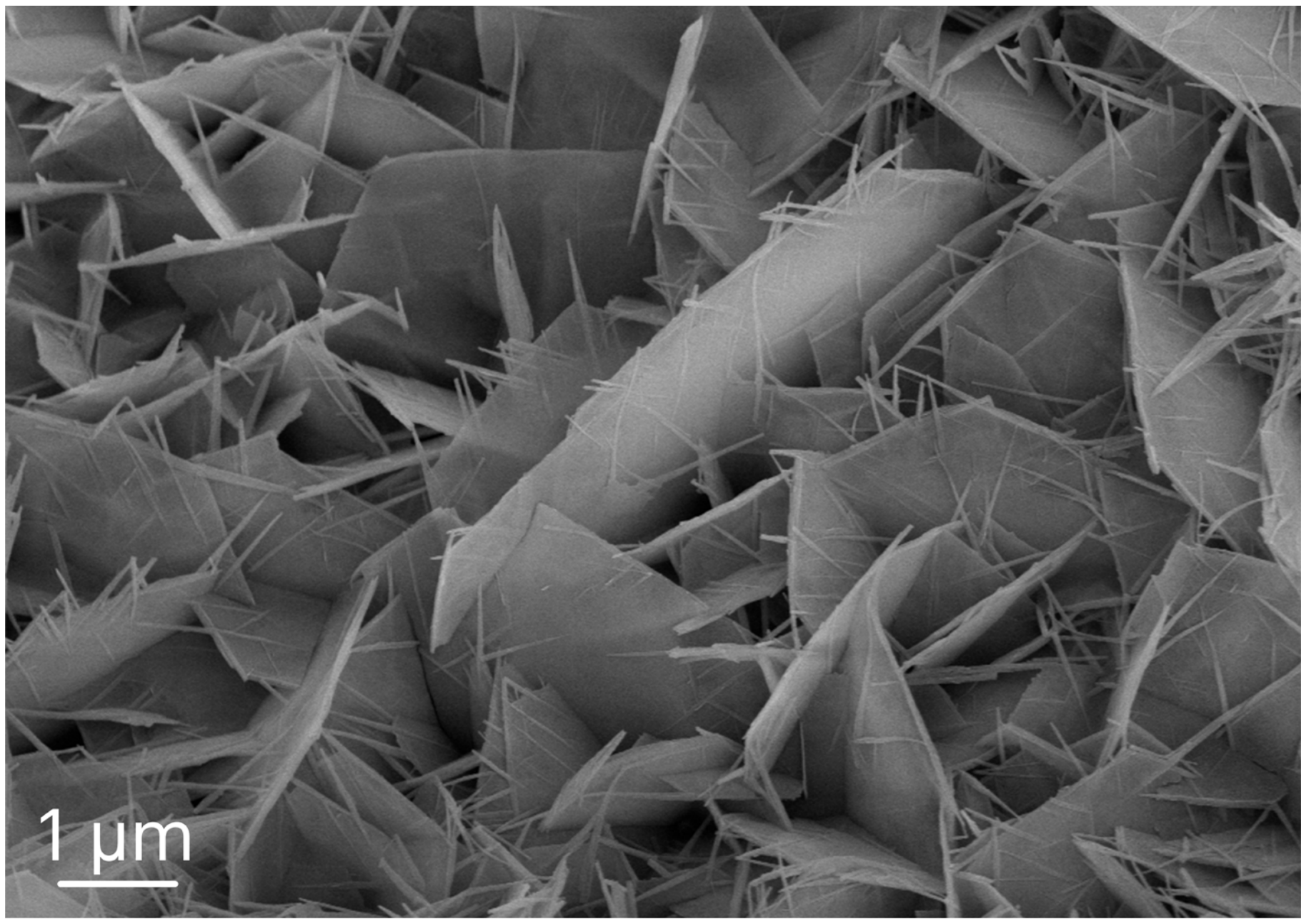

Fig. S8. Structural analysis. SEM image of $NiCo(OH)_2$ nanosheets.

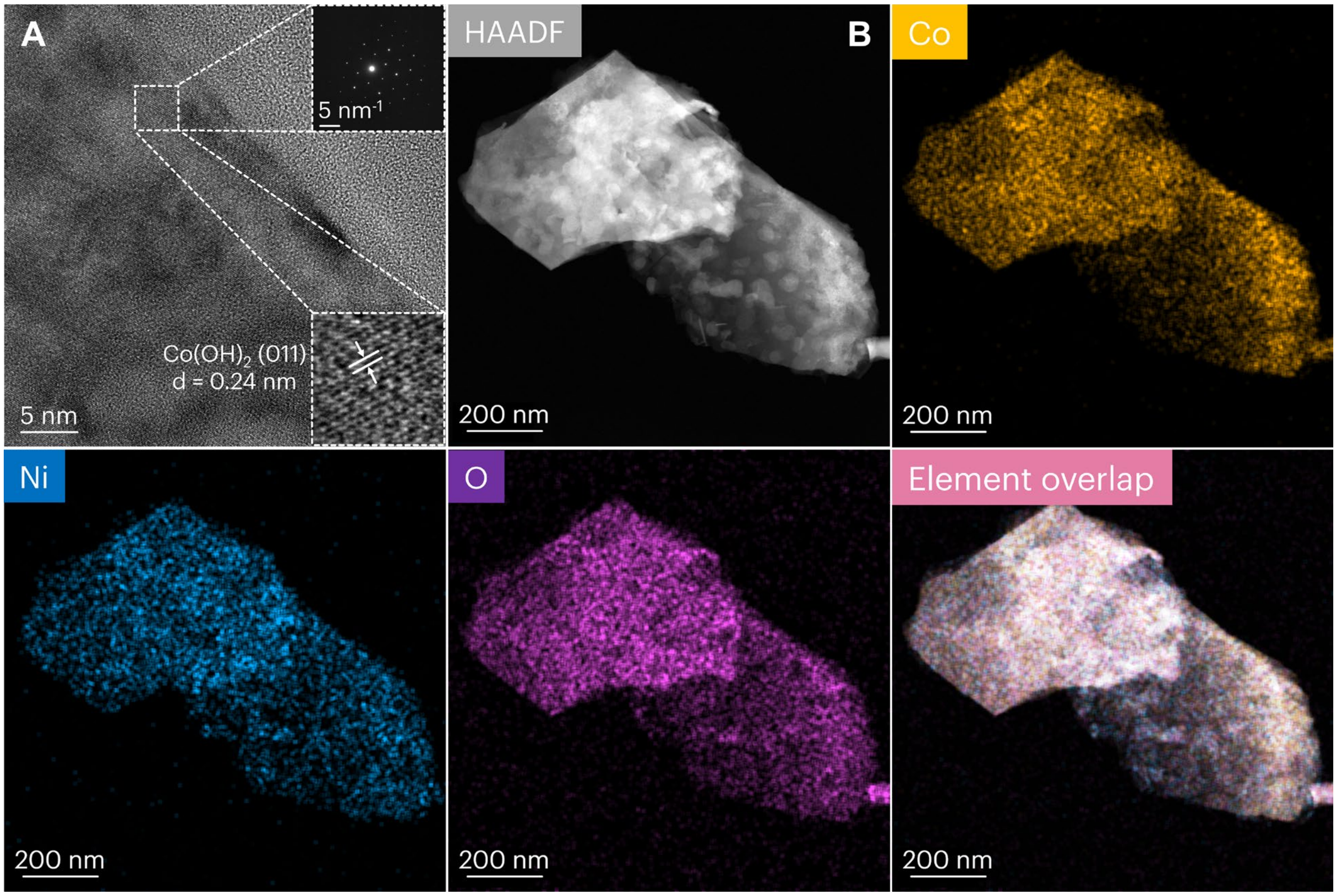

Fig. S9. Structural analysis. (**A**) Transmission electron microscope (TEM) image and (**B**) Energy dispersive spectroscopy (EDS) elemental mapping images for Co, Ni and O of the $NiCo(OH)_2$ precursor.

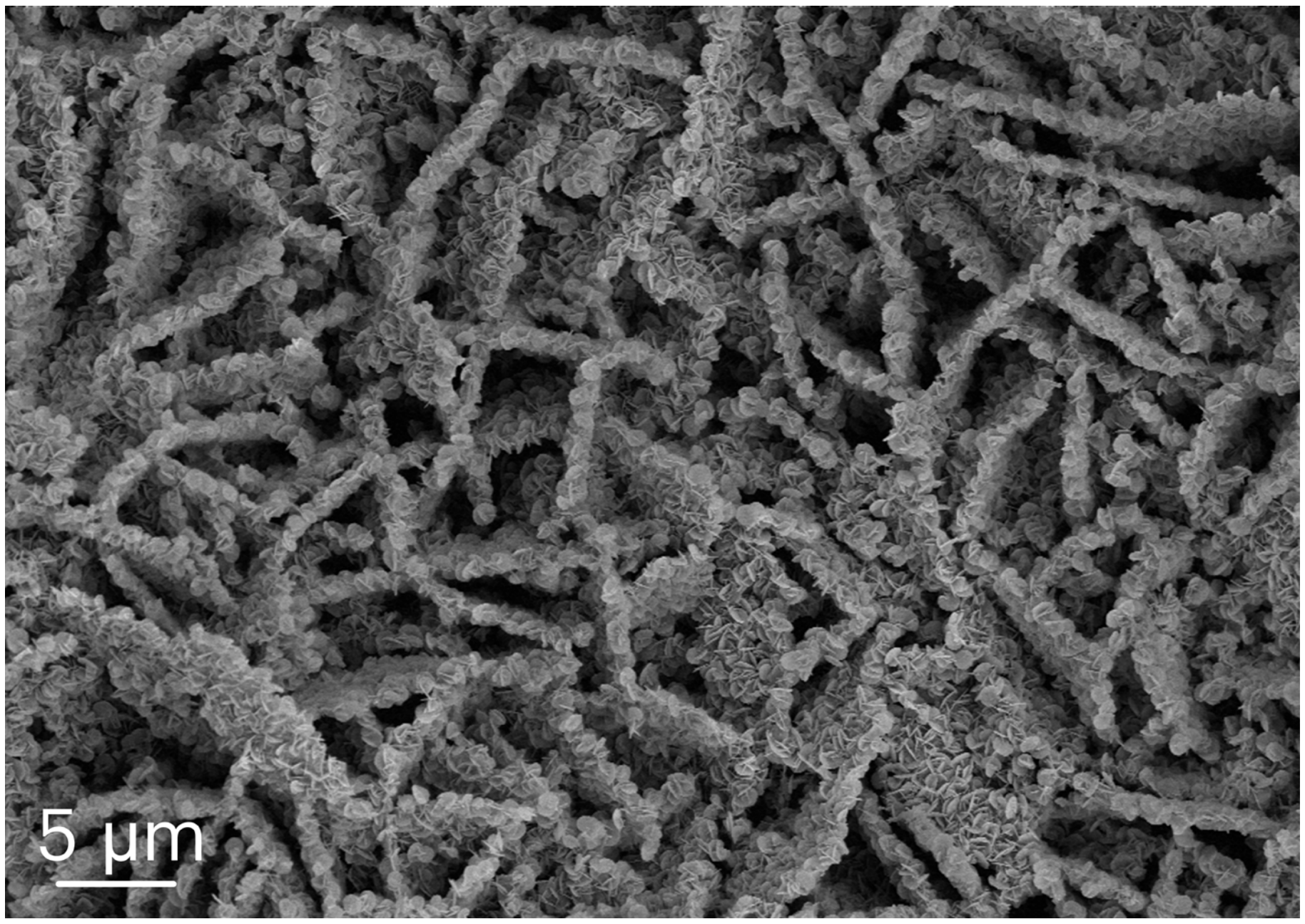

Fig. S10. Structural analysis. SEM image of $NiCoO_x$.

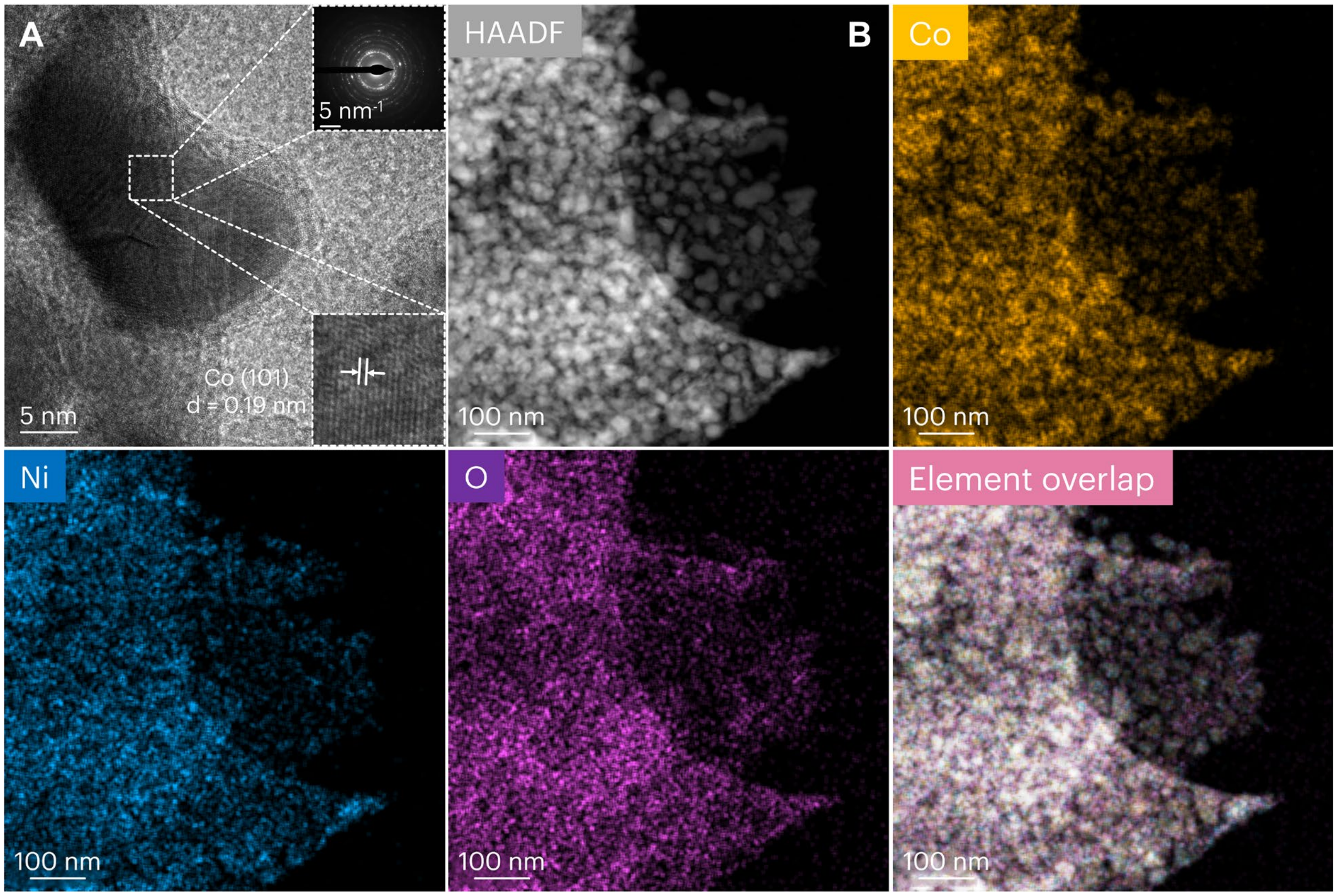

Fig. S11. Structural analysis. (**A**) High-resolution TEM image of $NiCoO_x$. (**B**) EDS elemental mapping images for Co, Ni and O of $NiCoO_x$.

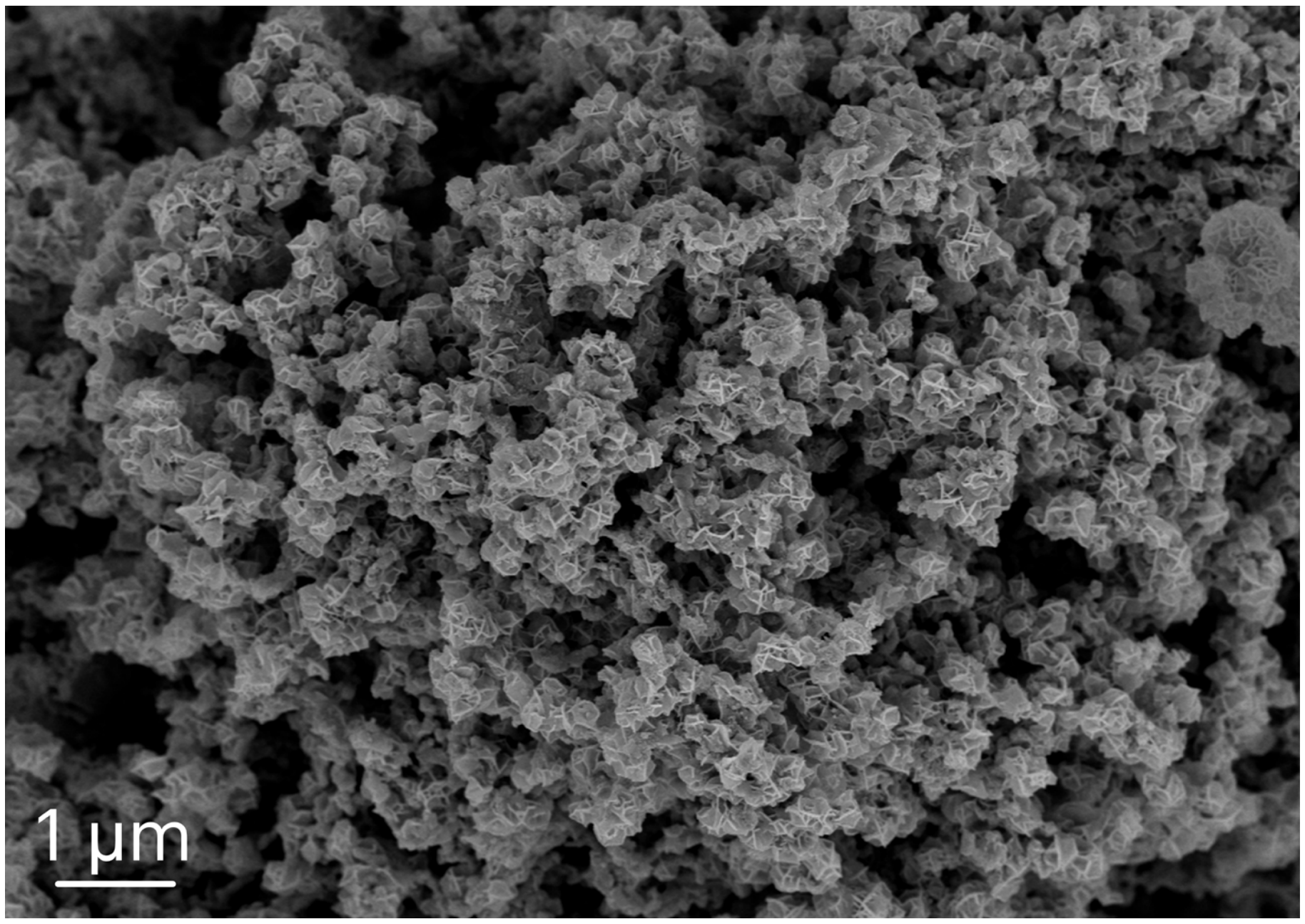

Fig. S12. Structural analysis. SEM image of Pt/NiCo(OH)$_x$.

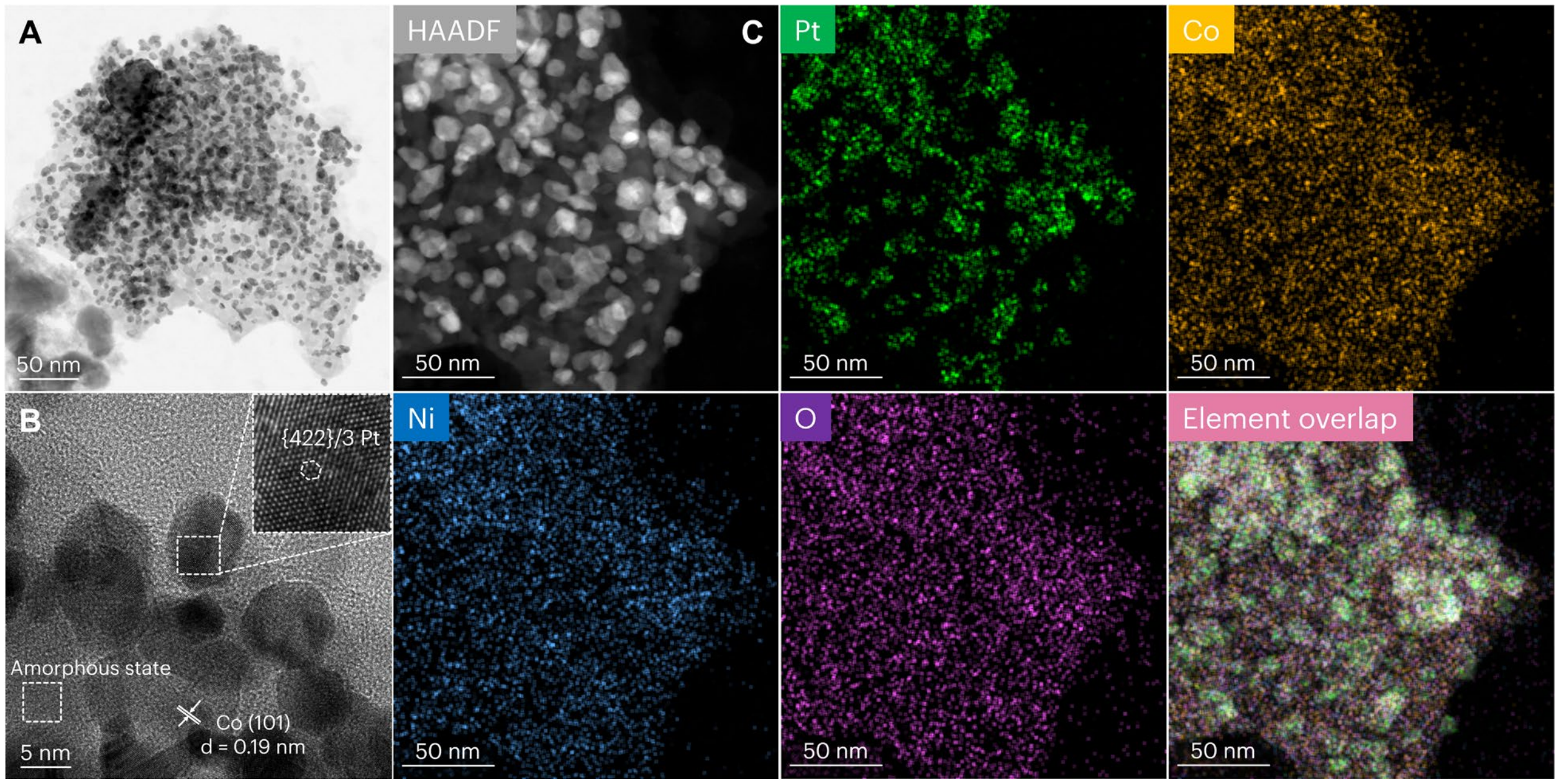

Fig. S13. Structural analysis. (**A**) TEM image and (**B**) high-resolution TEM image of Pt/NiCo(OH)$_x$. (**C**) EDS elemental mapping images for Pt, Co, Ni and O of Pt/NiCo(OH)$_x$.

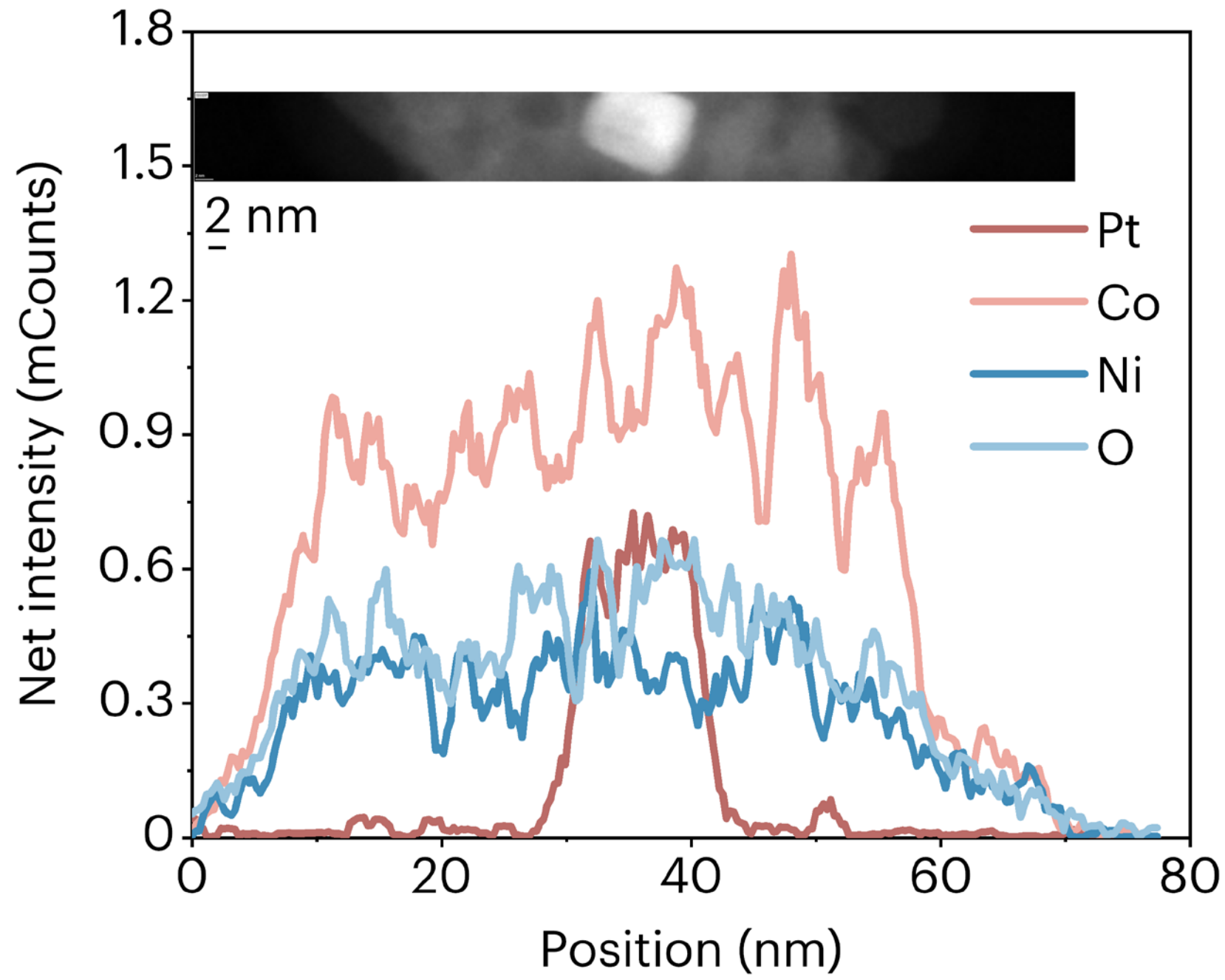

Fig. S14. Structural analysis. Energy-dispersive X-ray spectroscopy line scan of Pt/NiCo(OH)$_x$.

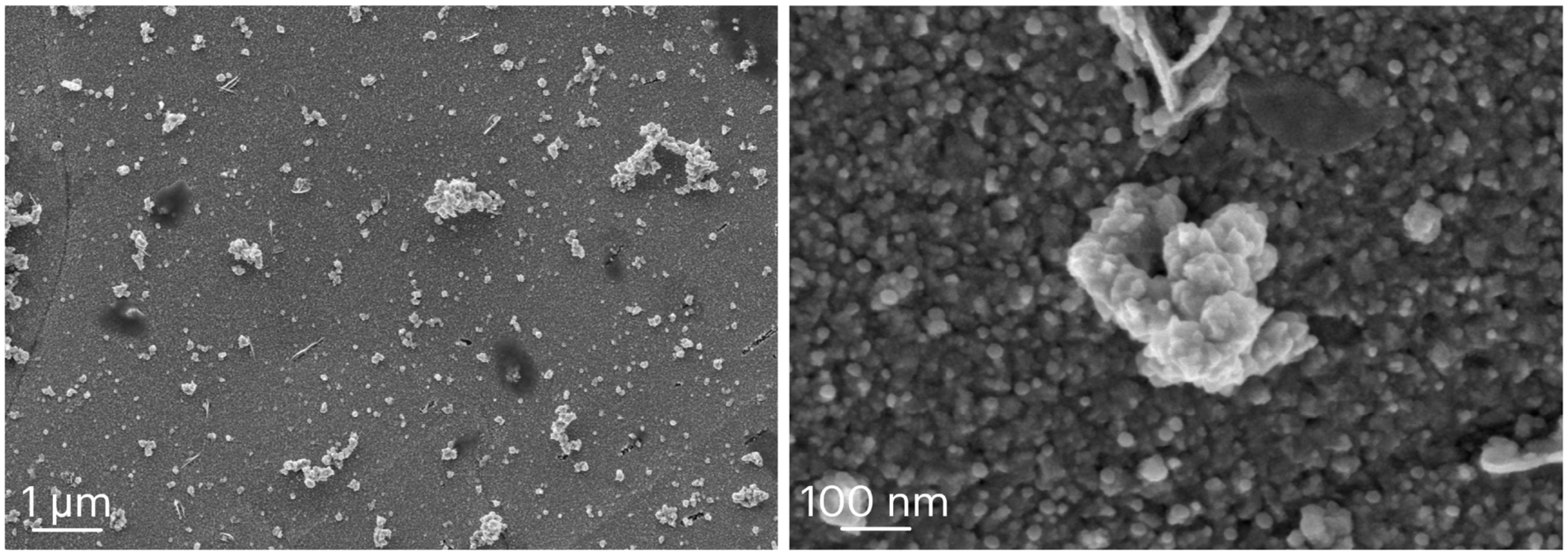

Fig. S15. Structural analysis. SEM images of electro-deposited Pt NPs on NF (Pt NPs/NF).

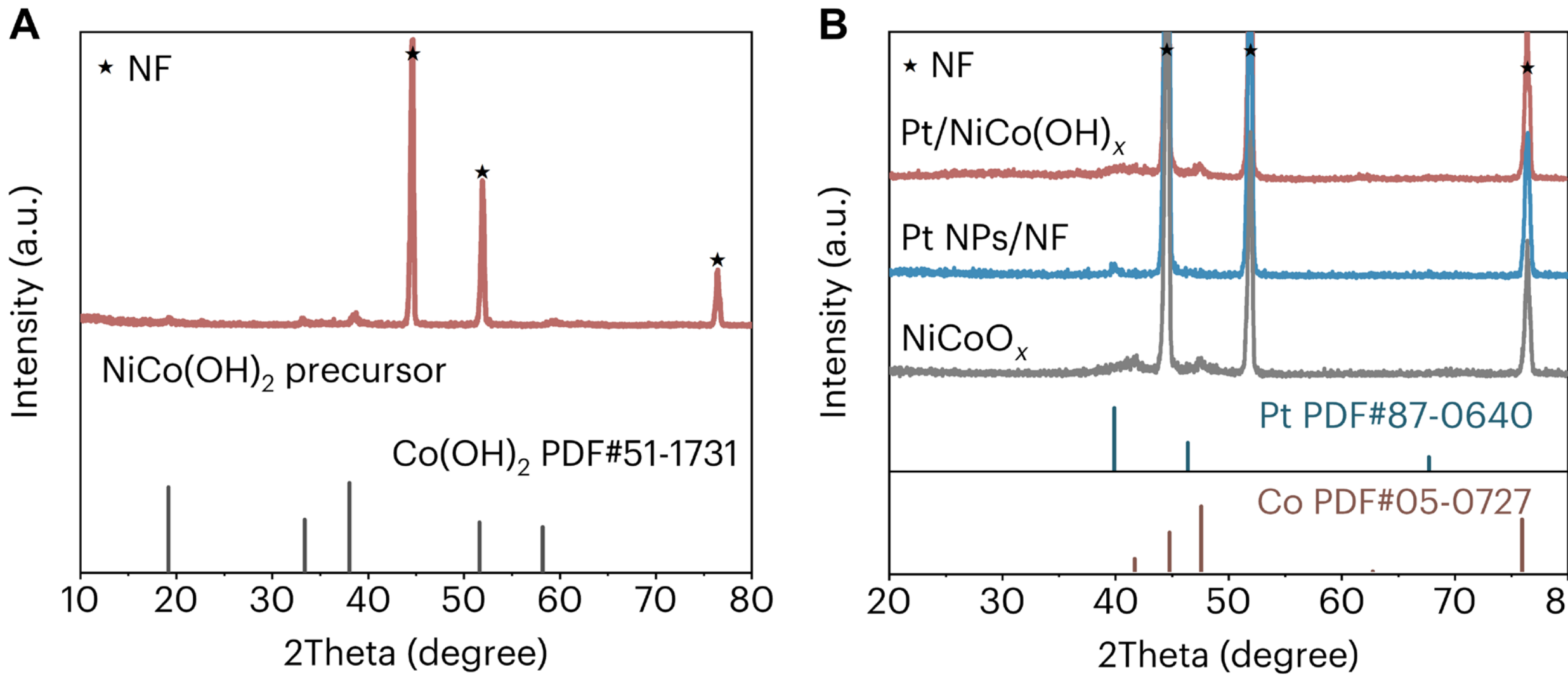

Fig. S16. Structural analysis. (**A**) X-ray diffraction (XRD) pattern of the $NiCo(OH)_2$ precursor. (**B**) XRD patterns of Pt/$NiCo(OH)_x$, Pt NPs/NF and $NiCoO_x$.

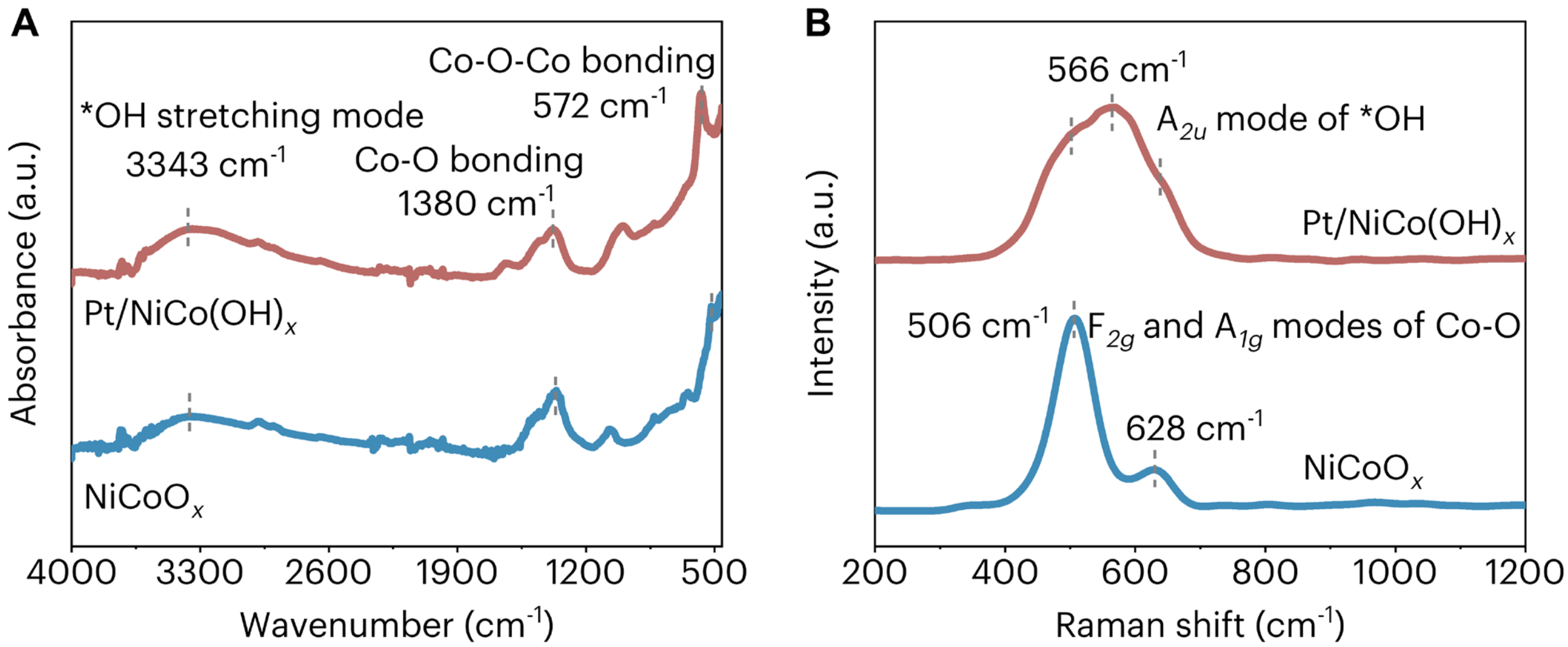

Fig. S17. Structural analysis. (**A**) Fourier transform infrared spectra (FTIR) and (**B**) Raman spectra of Pt/NiCo(OH)$_x$ and NiCoO$_x$.

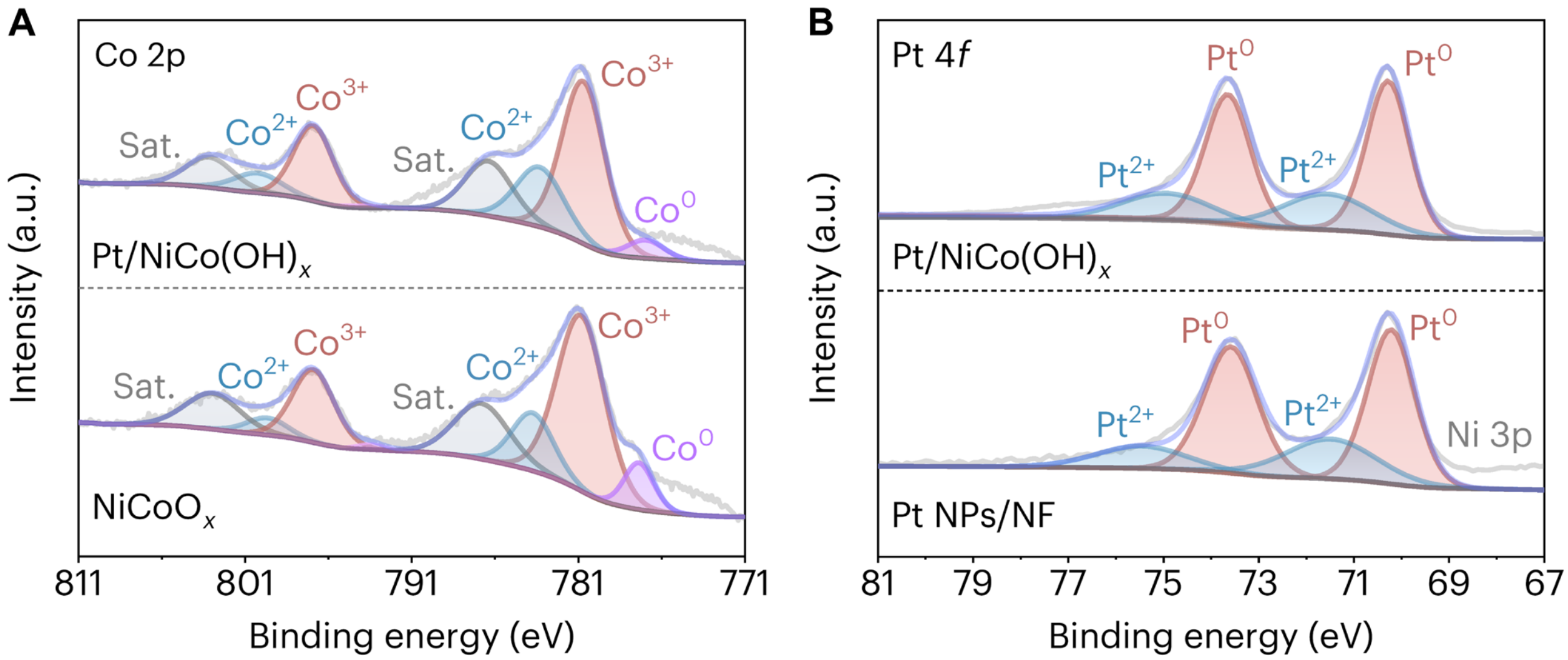

Fig. S18. Valence analysis. High-resolution (**A**) Co 2p X-ray photoelectron spectroscopy (XPS) spectra of $NiCoO_x$ and $Pt/NiCo(OH)_x$, and (**B**) Pt 4*f* XPS spectra of Pt NPs/NF and $Pt/NiCo(OH)_x$.

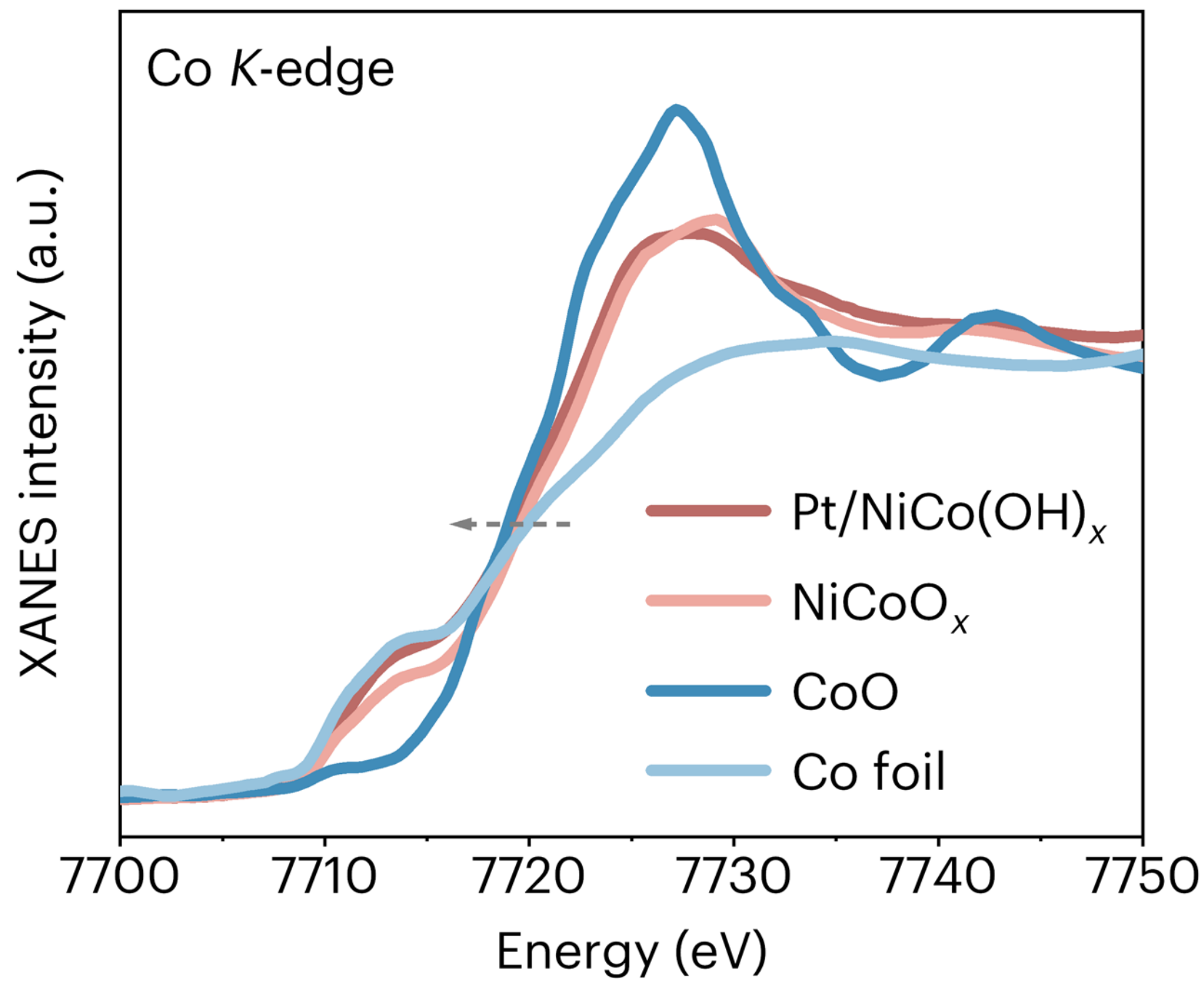

Fig. S19. Valence analysis. Normalized X-ray absorption near-edge structure spectra at the Co *K*-edge for Pt/NiCo(OH)$_x$, NiCoO$_x$, CoO and Co foil.

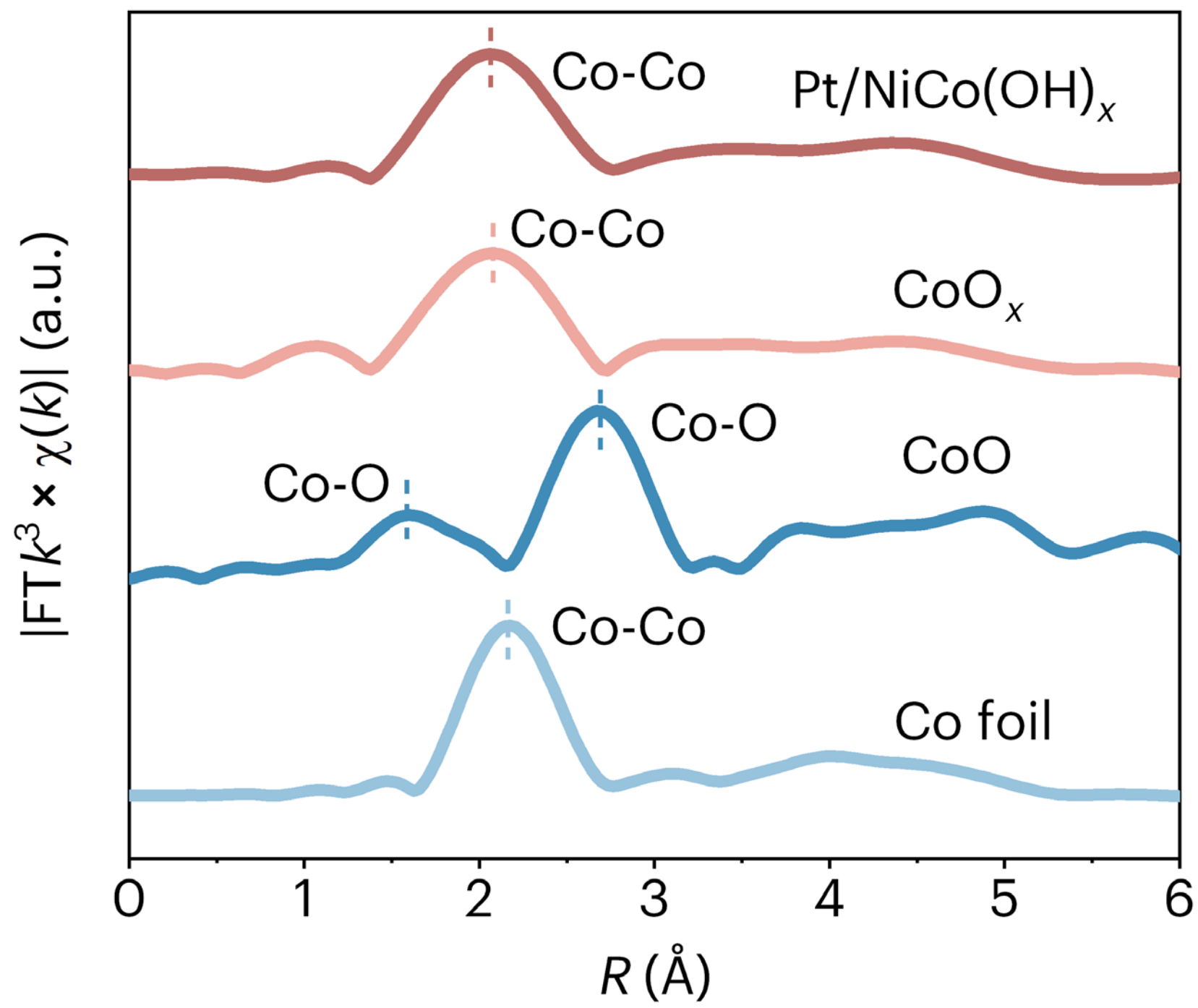

Fig. S20. Coordination analysis. Fourier-transform k^3-weighted $\chi(k)$-function at the Co K-edge for Pt/NiCo(OH)$_x$, NiCoO$_x$, CoO and Co foil.

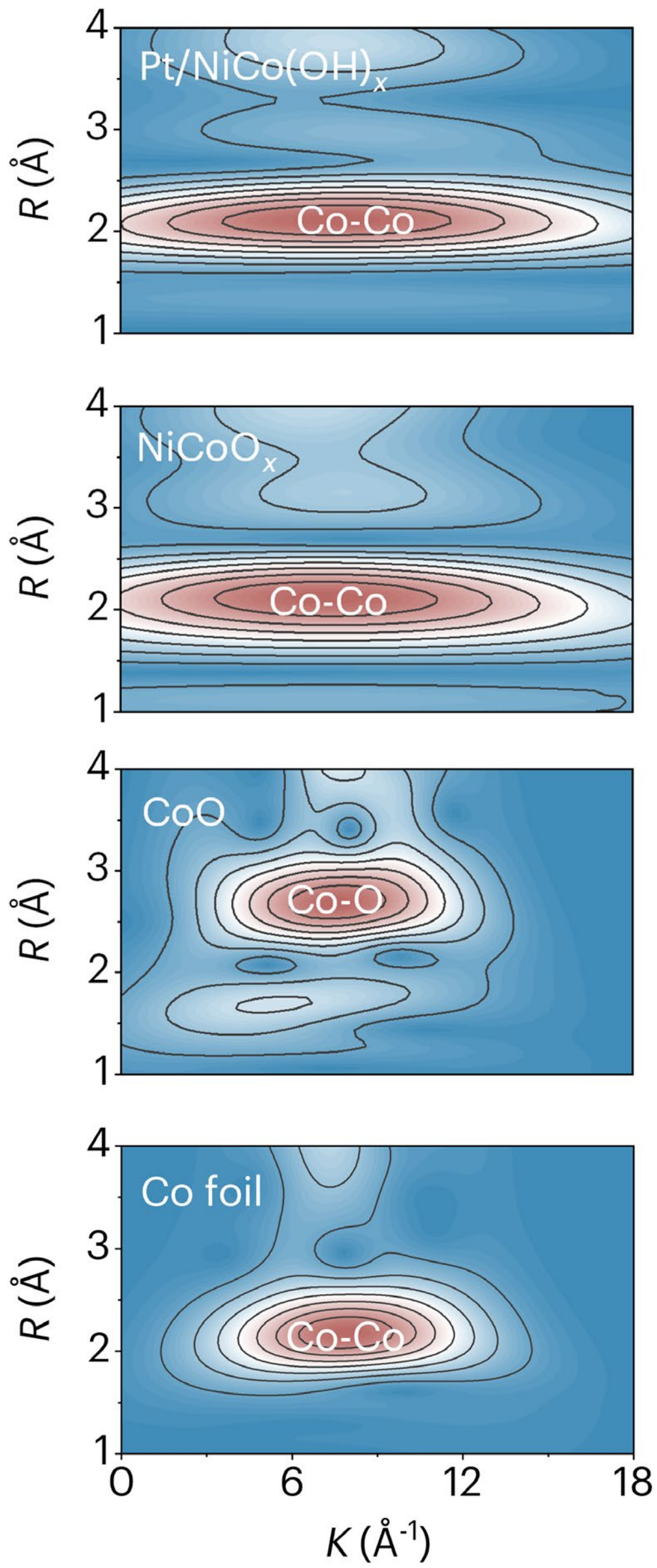

Fig. S21. Coordination analysis. Wavelet transformed k^3-weighted χ(k)-function at the Co K-edge for Pt/NiCo(OH)$_x$, NiCoO$_x$, CoO and Co foil.

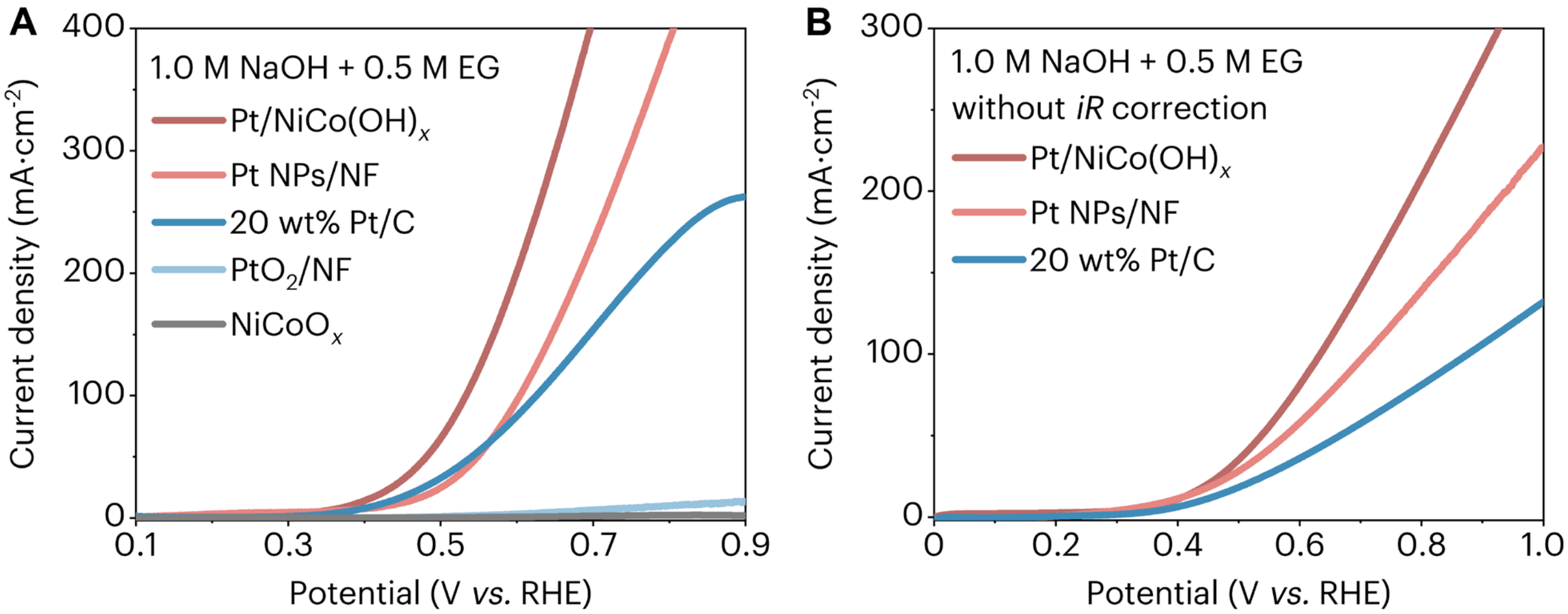

Fig. S22. Electrochemical performance. (**A**) EGOR LSV curves of Pt/NiCo(OH)$_x$, Pt NPs/NF, 20 wt% Pt/C, PtO_2/NF and NiCoO$_x$ with *iR* correction in 1.0 M NaOH with 0.5 M EG (normalized to geometric area). (**B**) EGOR LSV curves of Pt/NiCo(OH)$_x$, Pt NPs/NF and 20 wt% Pt/C without *iR* correction in 1.0 M NaOH with 0.5 M EG.

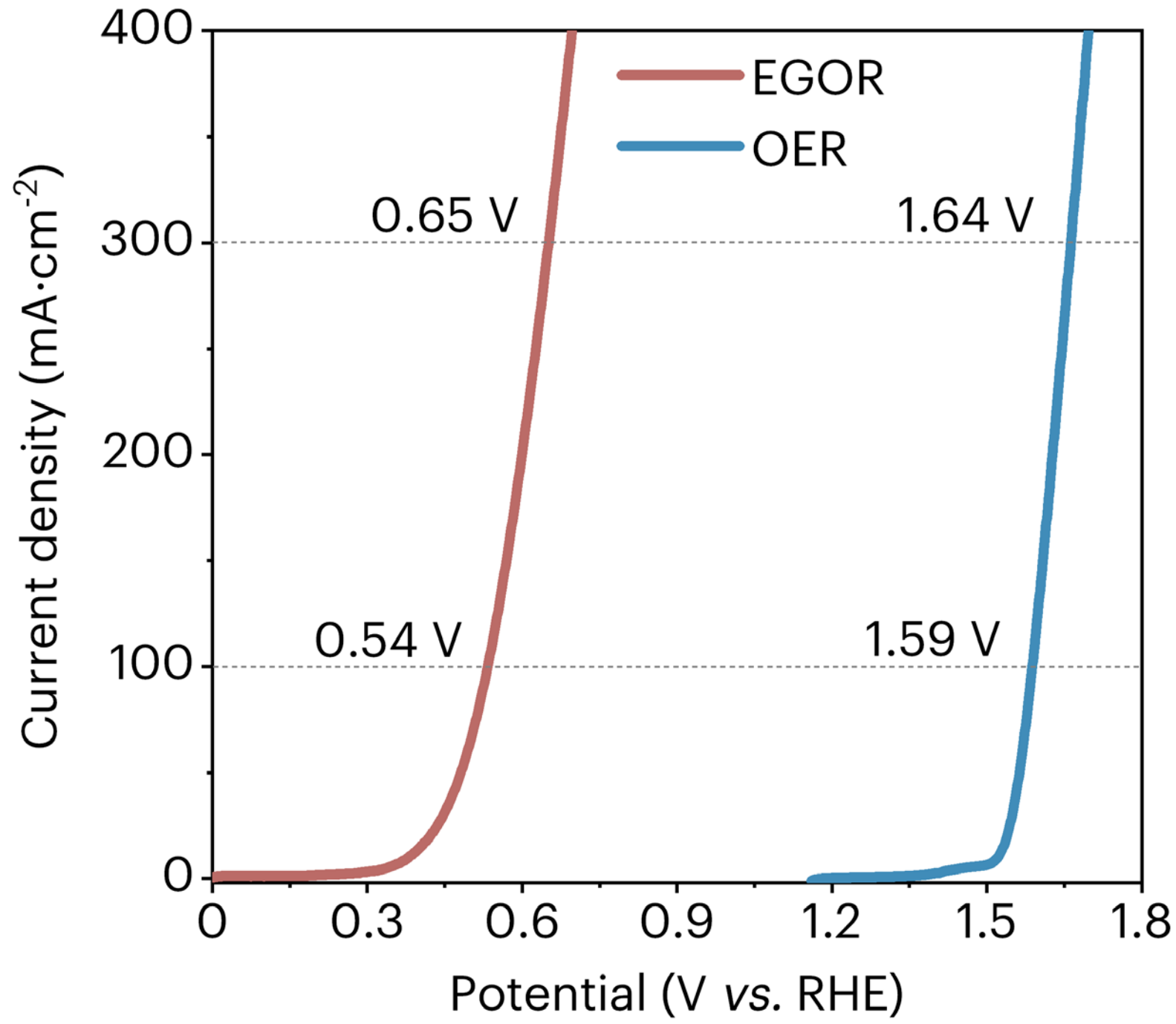

Fig. S23. Electrochemical performance. EGOR and OER LSV curves for Pt/NiCo(OH)*x*.

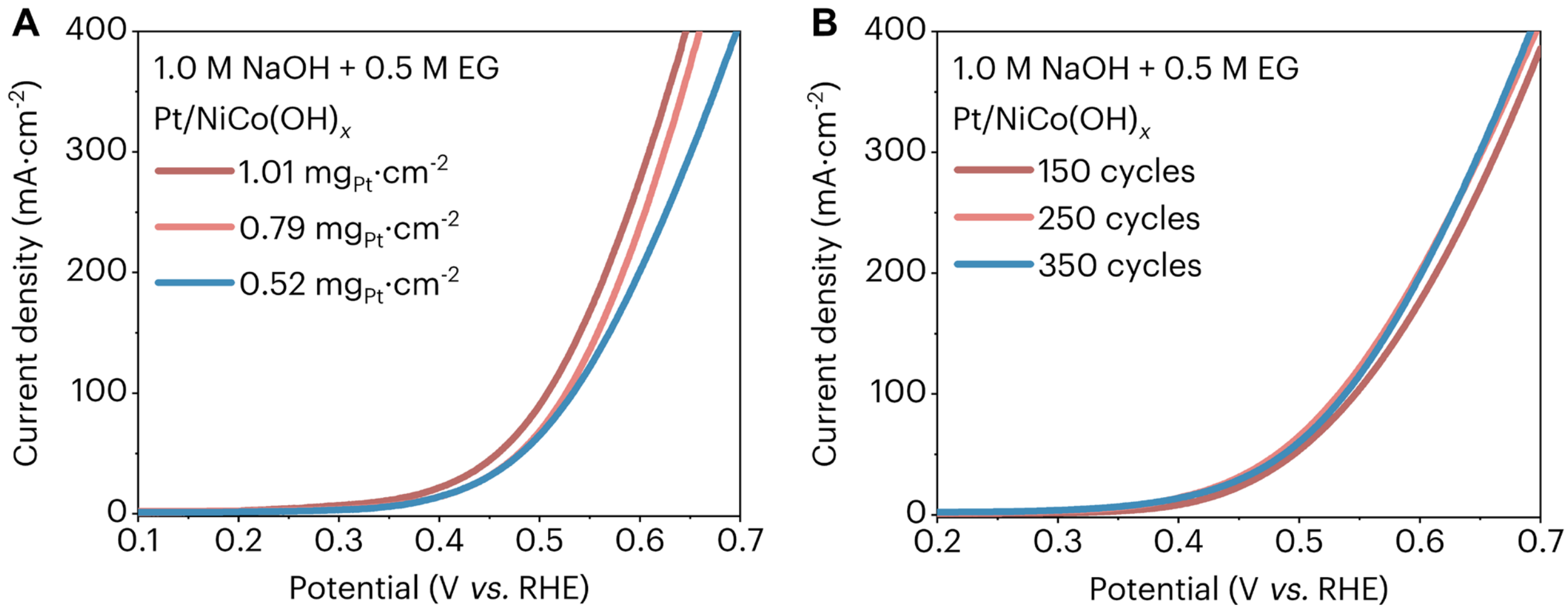

Fig. S24. Electrochemical performance. (**A**) EGOR LSV curves of Pt/NiCo(OH)$_x$ with different Pt mass loadings. (**B**) EGOR LSV curves of Pt/NiCo(OH)$_x$ after different electrodeposition cycles.

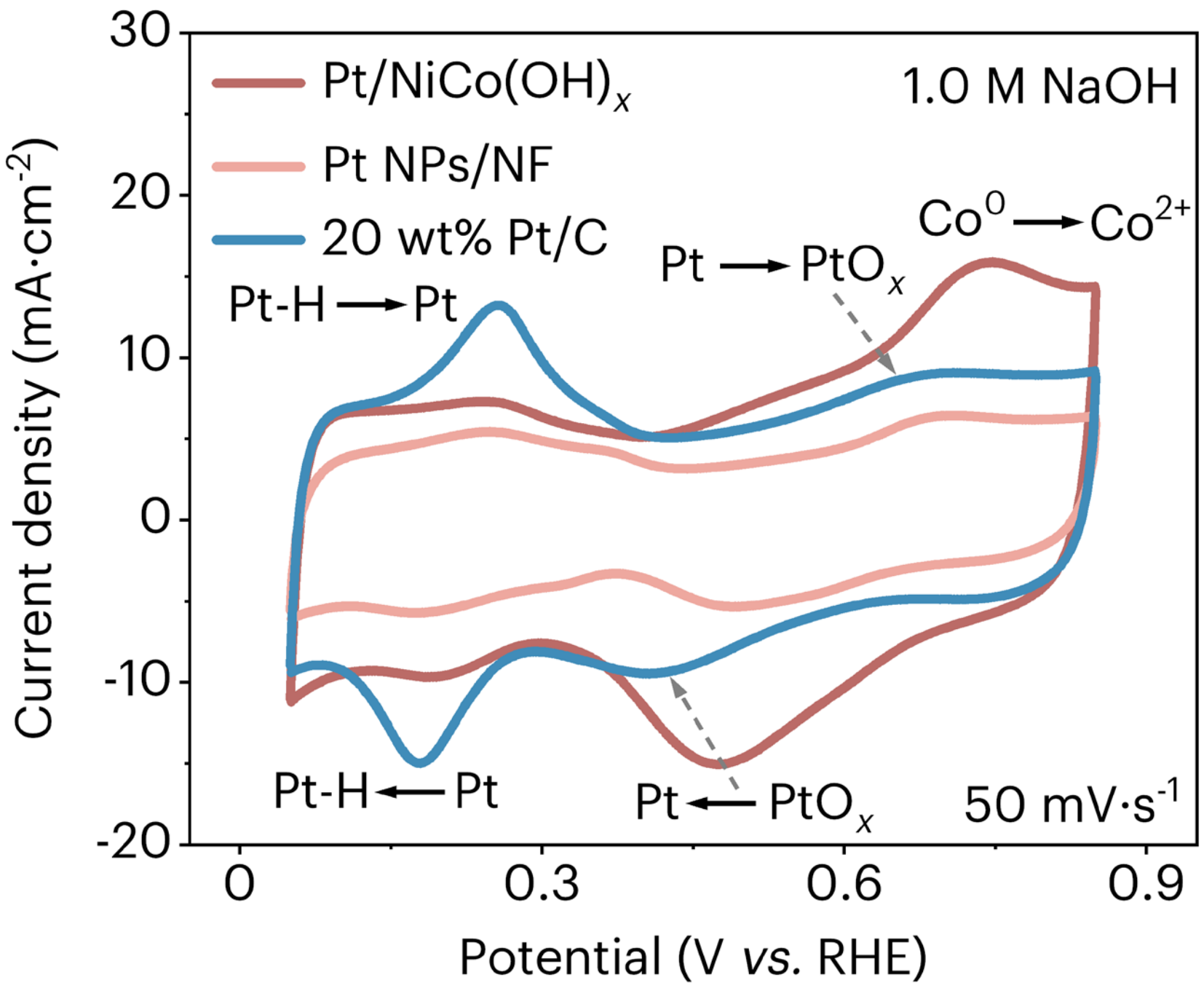

Fig. S25. Electrochemical active site analysis. Cyclic voltammetry (CV) curves of Pt/NiCo(OH)$_x$, Pt NPs/NF and 20 wt% Pt/C in 1.0 M NaOH.

Note: The values of ECSA$_{Pt}$ for Pt/NiCo(OH)$_x$, Pt NPs/NF and 20 wt% Pt/C are 39.9, 36.4 and 57.1 $cm^2 \cdot mg_{Pt}^{-1}$. Hence, the values of ECSA for Pt/NiCo(OH)$_x$, Pt NPs/NF and 20 wt% Pt/C are 20.8, 19.7 and 27.4 cm^2.

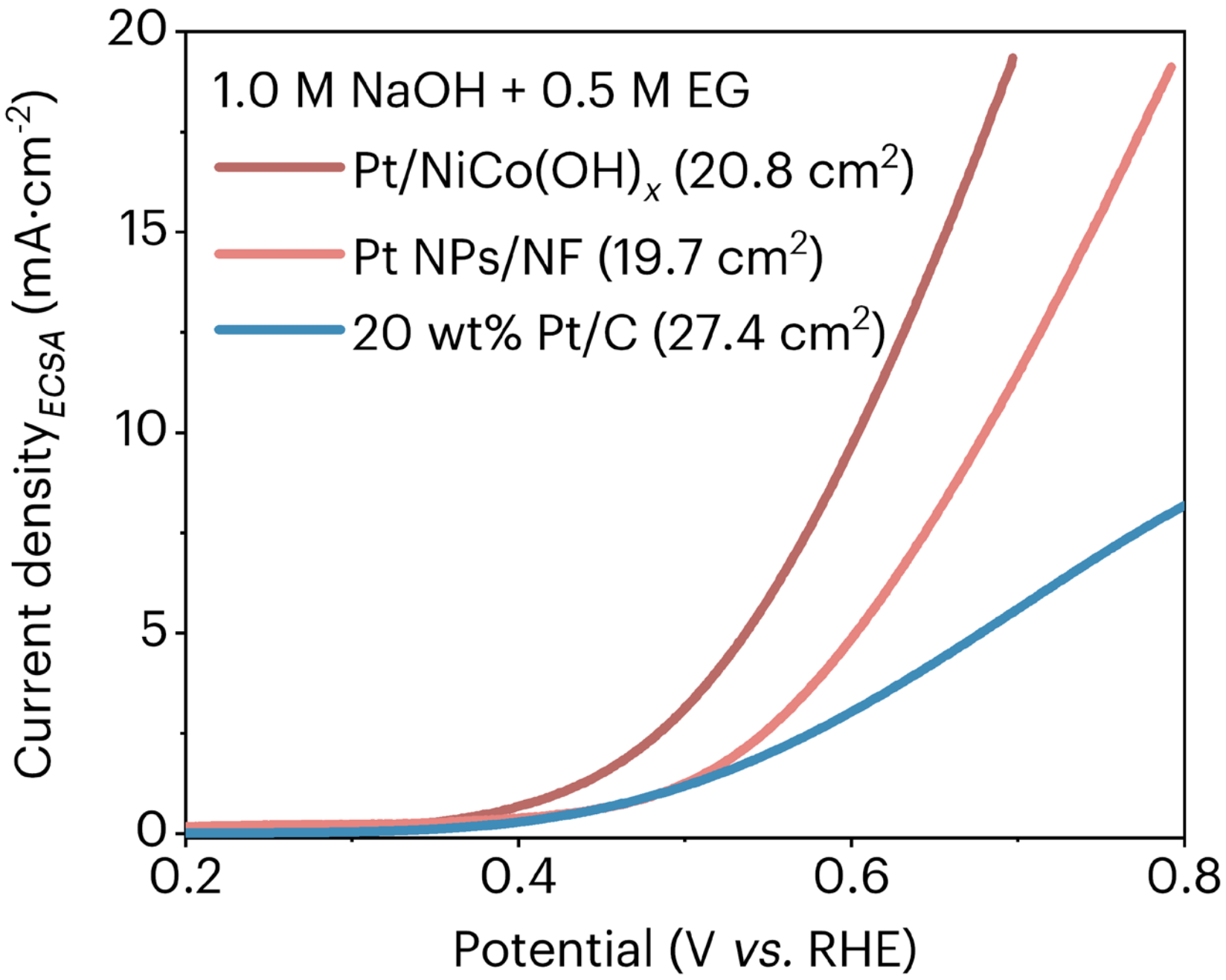

Fig. S26. Electrochemical active site analysis. ECSA corrected EGOR LSV curves of Pt/NiCo(OH)$_x$, Pt NPs/NF and 20 wt% Pt/C.

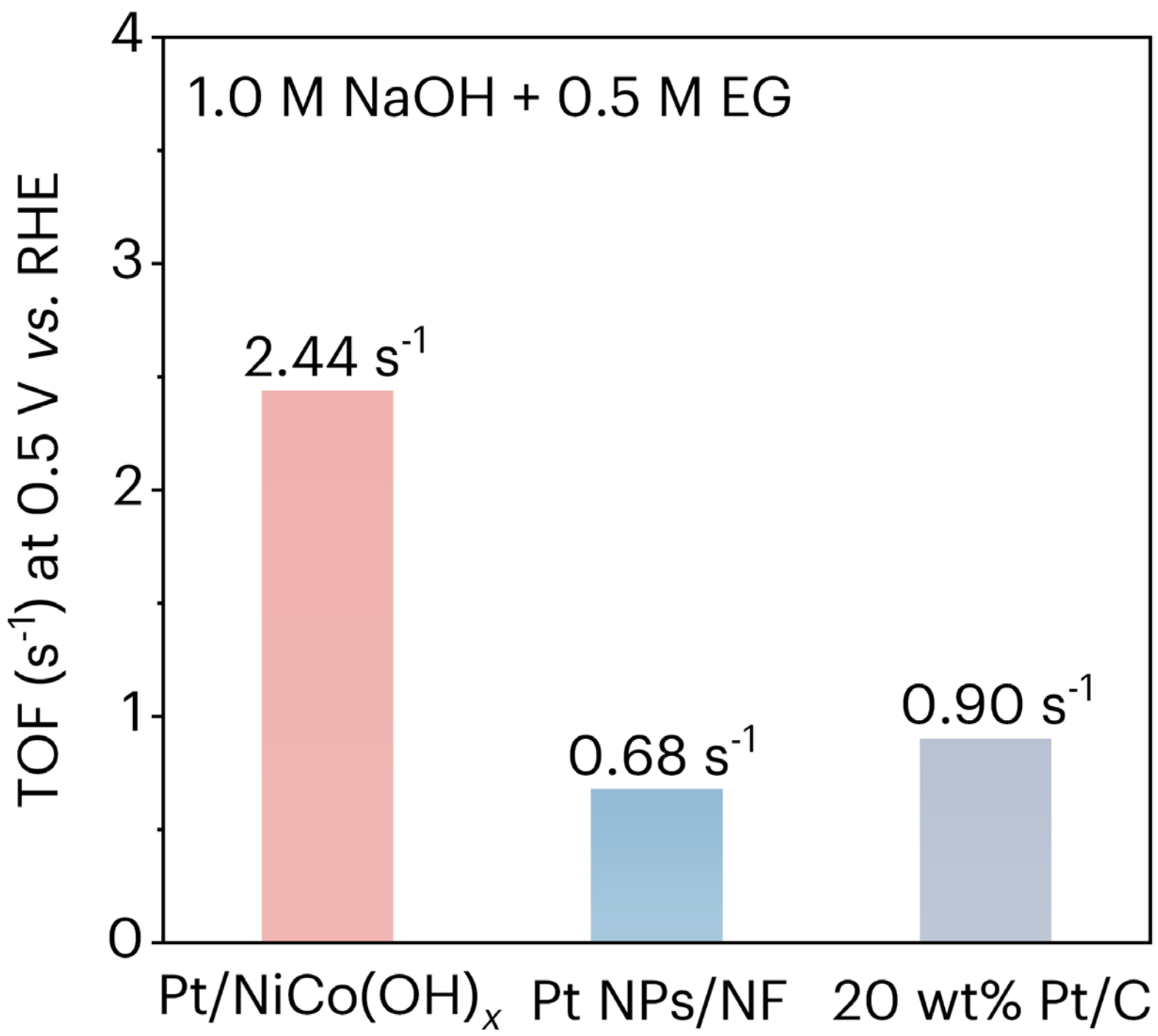

Fig. S27. Electrochemical active site analysis. EGOR TOF values of Pt sites for Pt/NiCo(OH)$_x$, Pt NPs/NF and 20 wt% Pt/C at 0.5 V *vs.* RHE.

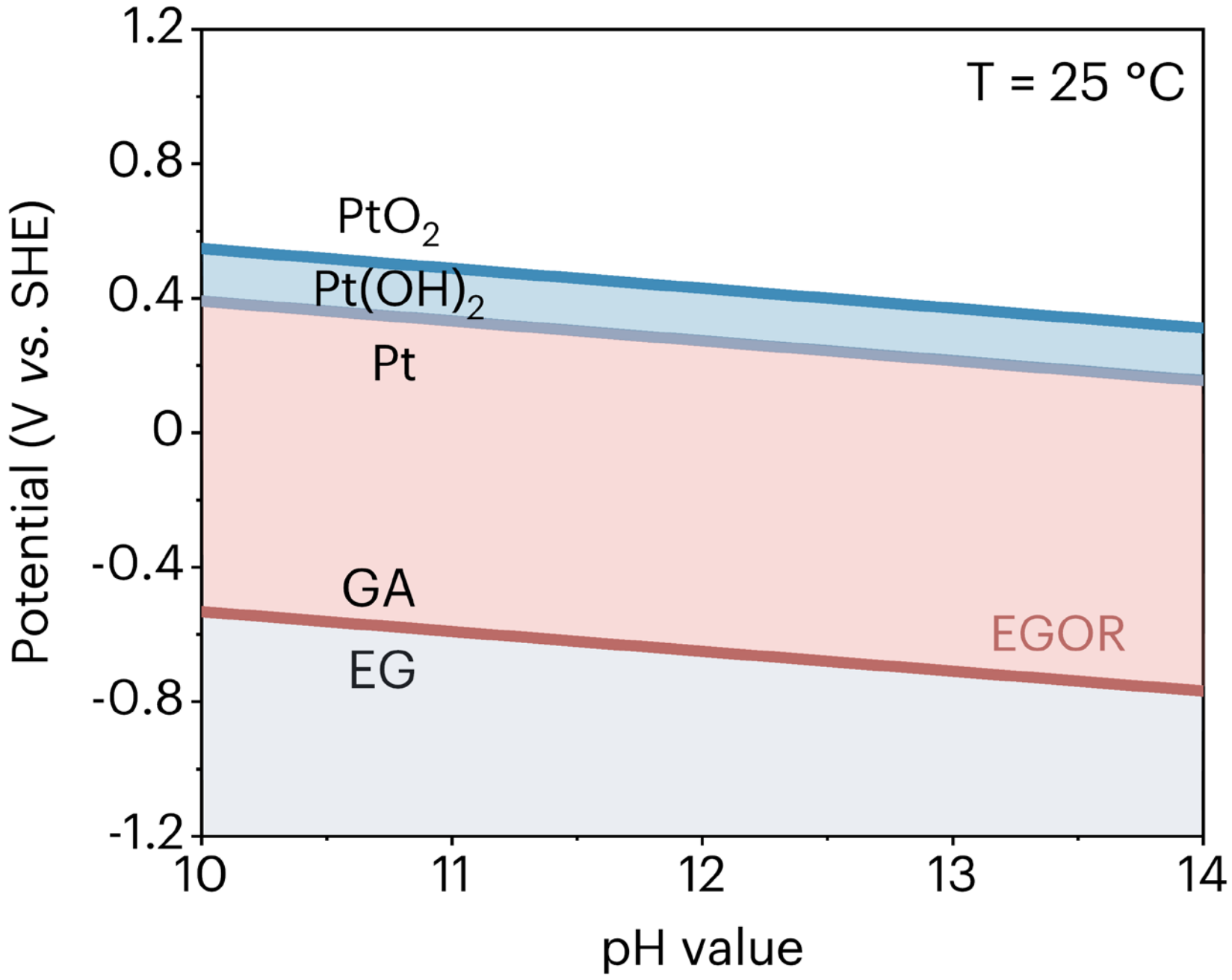

Fig. S28. Theoretical analysis. Pourbaix *E*-pH diagram of Pt oxidation (Pt to $Pt(OH)_2$, $Pt(OH)_2$ to PtO_2) and EGOR under different pH values.

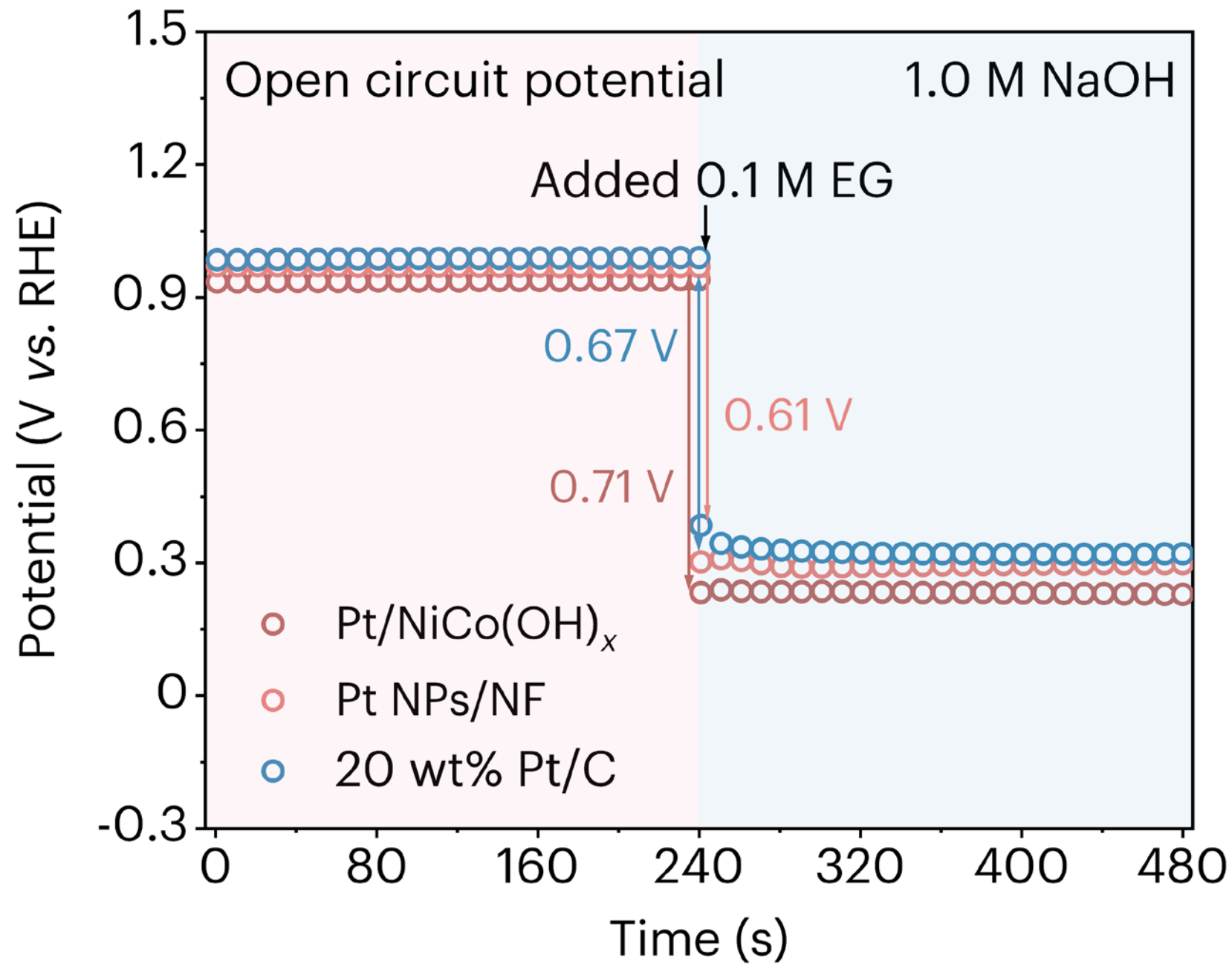

Fig. S29. EGOR mechanism analysis. Open circuit potential (OCP) measurements of Pt/NiCo(OH)$_x$, Pt NPs/NF and 20 wt% Pt/C under different conditions.

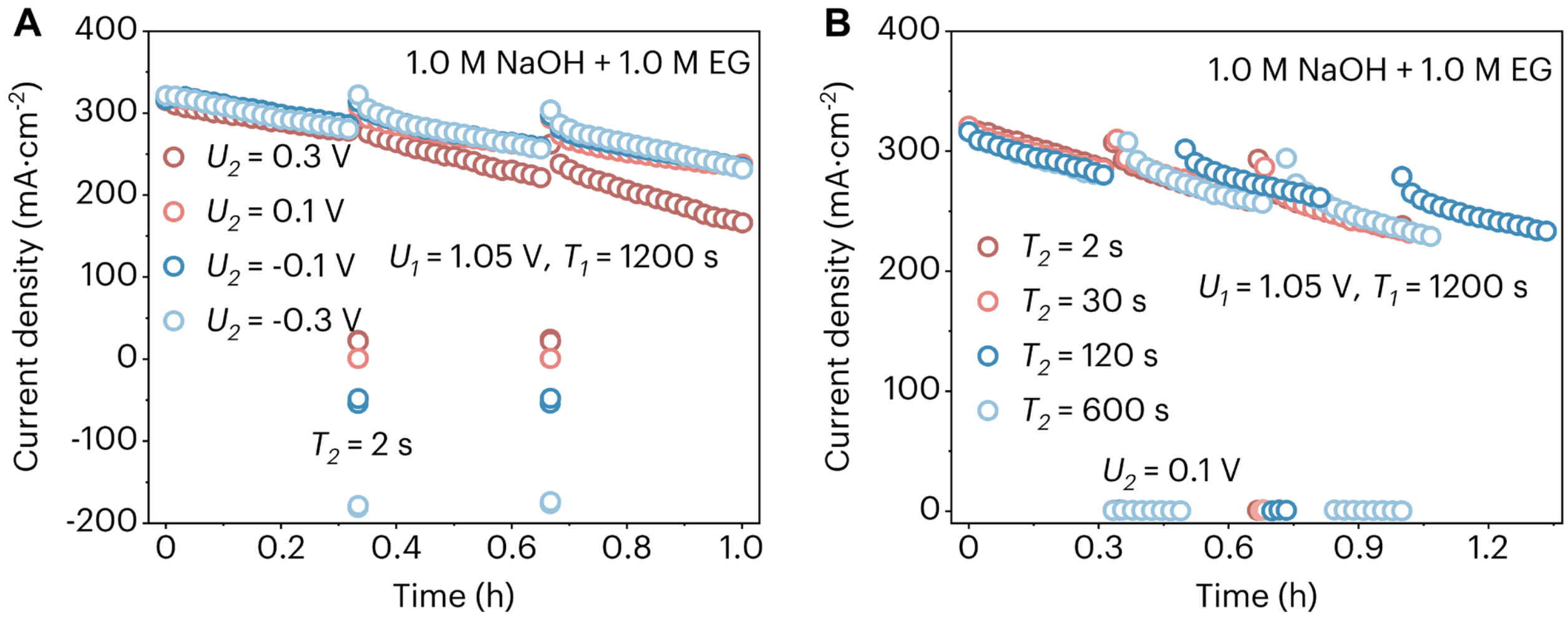

Fig. S30. Electrochemical performance. EGOR stability tests through intermittent potential (IP) method with different (**A**) intermittent potentials and (**B**) intermittent times for Pt/NiCo(OH)$_x$.

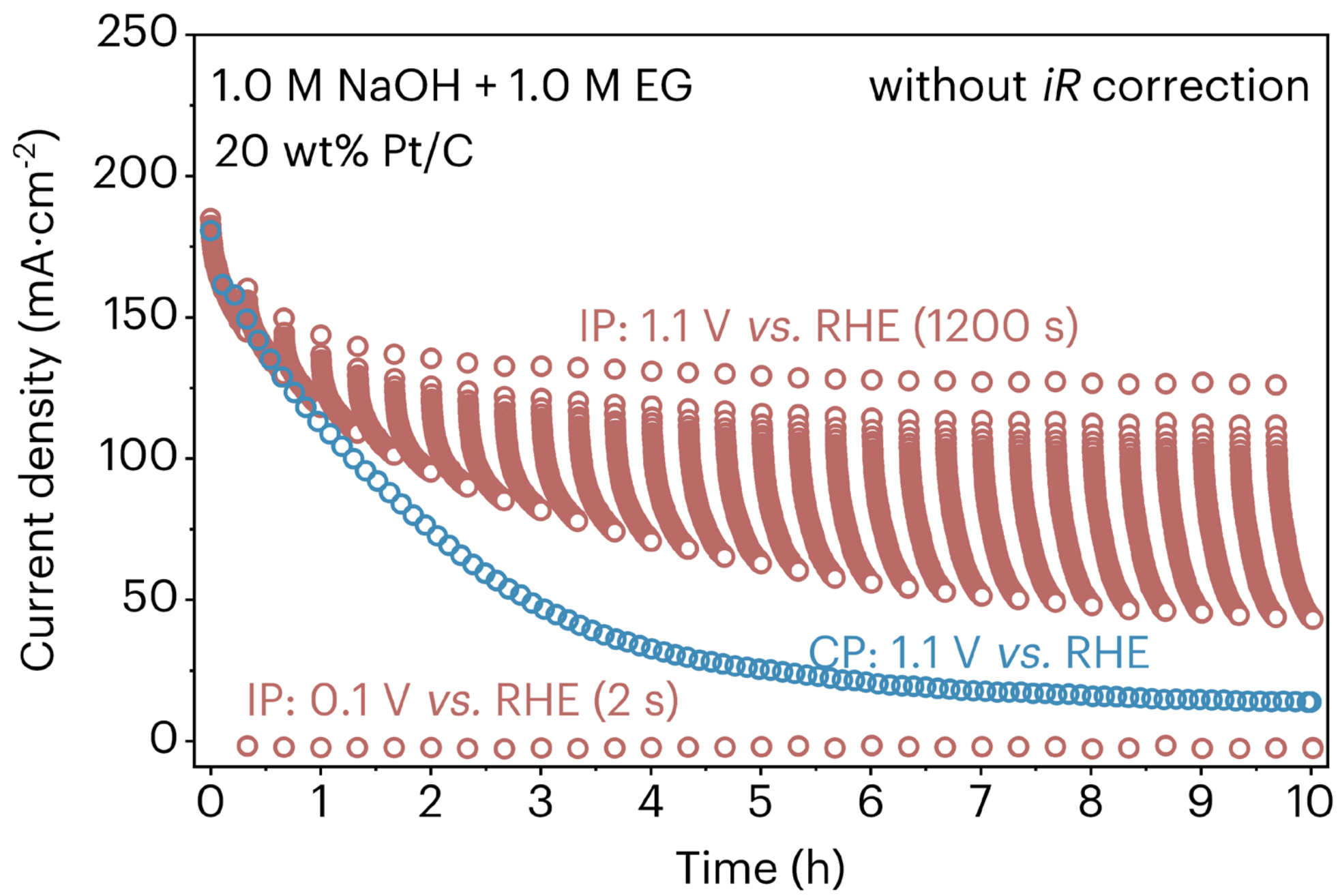

Fig. S31. Electrochemical performance. EGOR stability tests through constant potential (CP, 1.1 V *vs.* RHE) and intermittent potential (IP, 1.1 V *vs.* RHE for 1200 s, 0.1 V *vs.* RHE for 2 s) methods for commercial 20 wt% Pt/C.

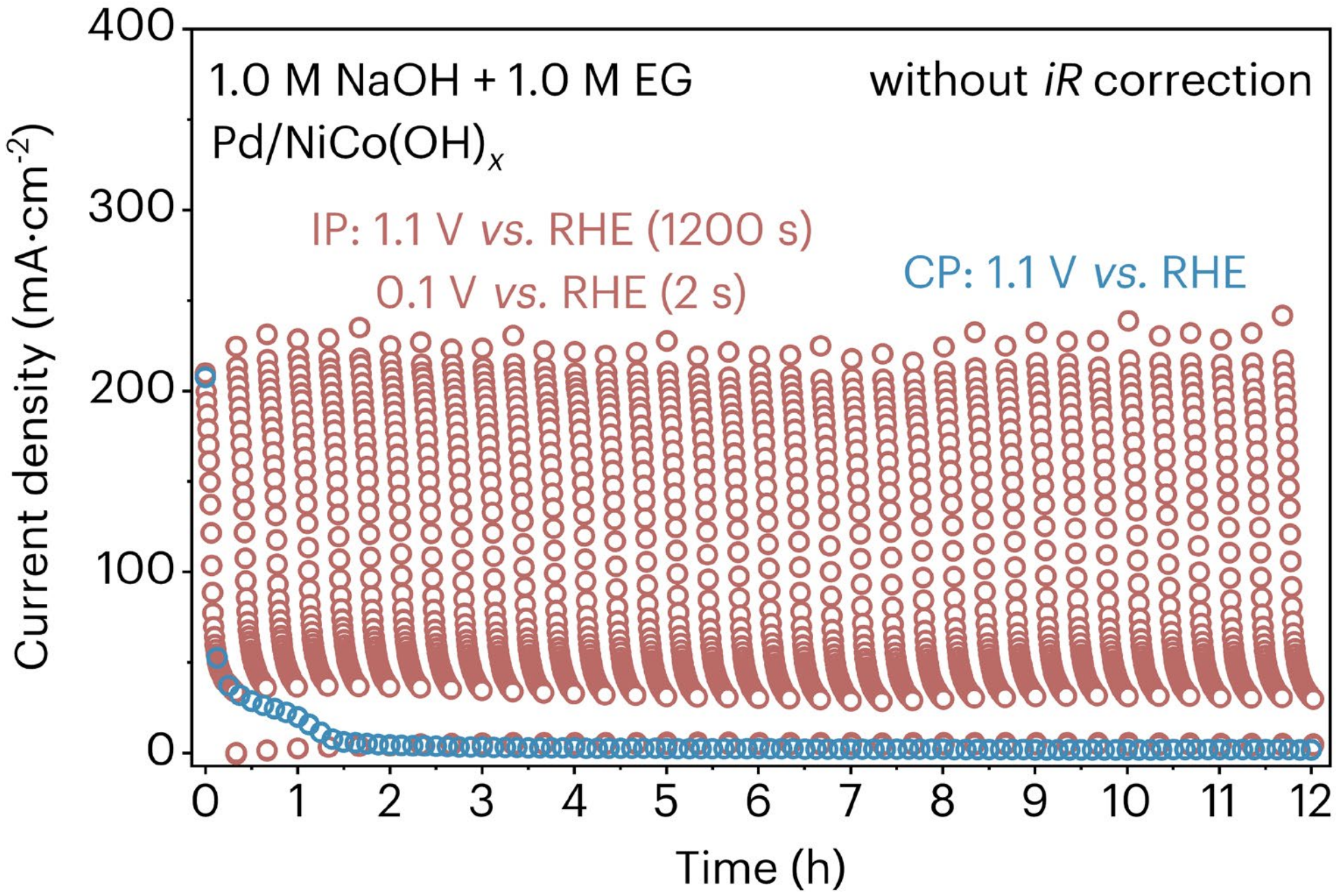

Fig. S32. Electrochemical performance. EGOR stability tests through constant potential (CP, 1.1 V *vs.* RHE) and intermittent potential (IP, 1.1 V *vs.* RHE for 1200 s, 0.1 V *vs.* RHE for 2 s) methods for Pd/NiCo(OH)$_x$.

Note: The preparation scheme for the Pd/NiCo(OH)$_x$ catalyst is to add a piece of NiCoO$_x$ (1.0 cm^2) into the 5 mL aqueous solution containing 3 mg K_2PdCl_4, 10 μL 3 M HCl and 20 μL ethanol under 80 °C for 6 hours.

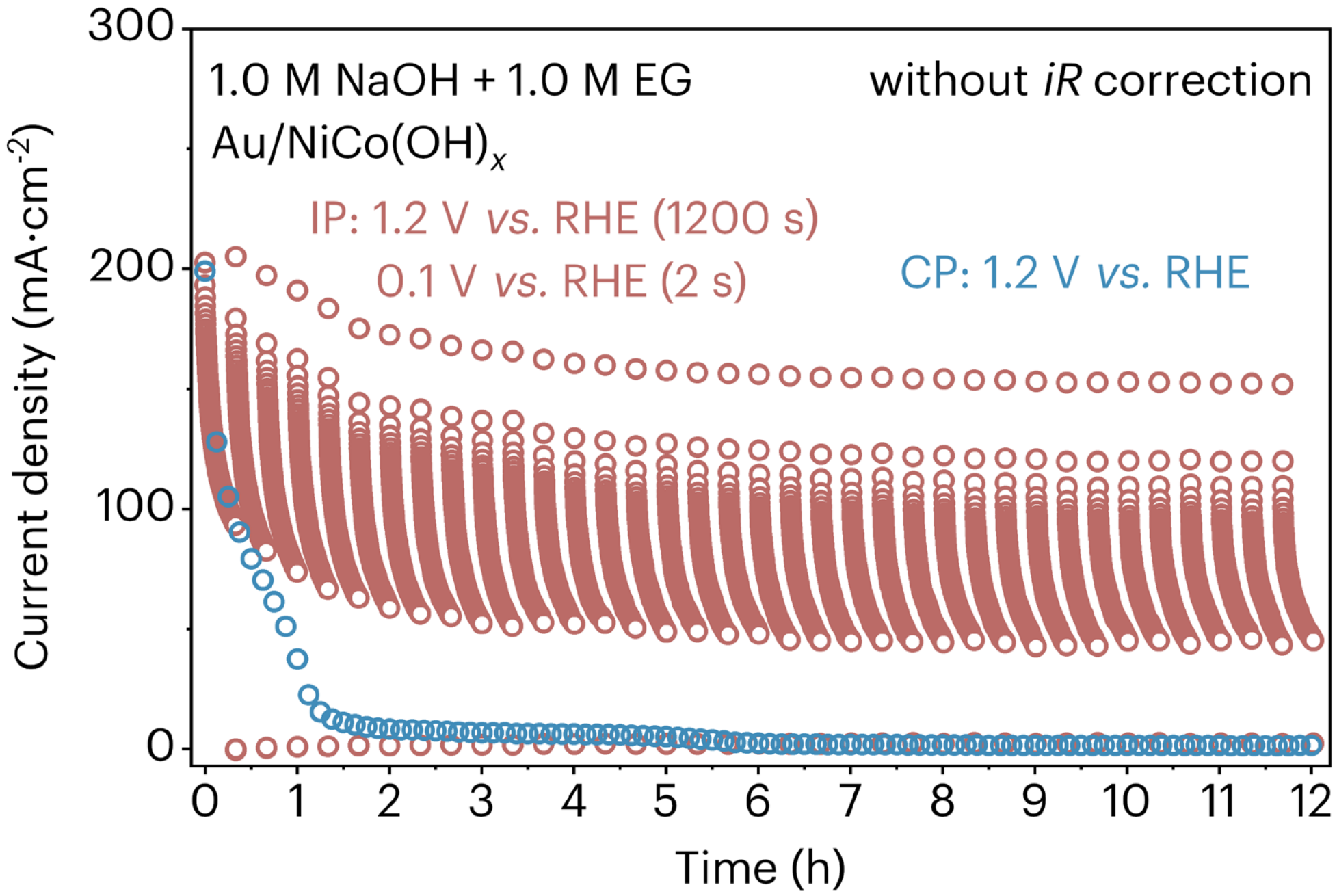

Fig. S33. Electrochemical performance. EGOR stability tests through constant potential (CP, 1.2 V *vs.* RHE) and intermittent potential (IP, 1.2 V *vs.* RHE for 1200 s, 0.1 V *vs.* RHE for 2 s) methods for Au/NiCo(OH)$_x$.

Note: The preparation scheme for the Au/NiCo(OH)$_x$ catalyst is to add a piece of NiCoO$_x$ (1.0 cm^2) into the 5 mL aqueous solution containing 2 mg $AuCl_3 \cdot 3H_2O$ under 80 °C for 6 hours.

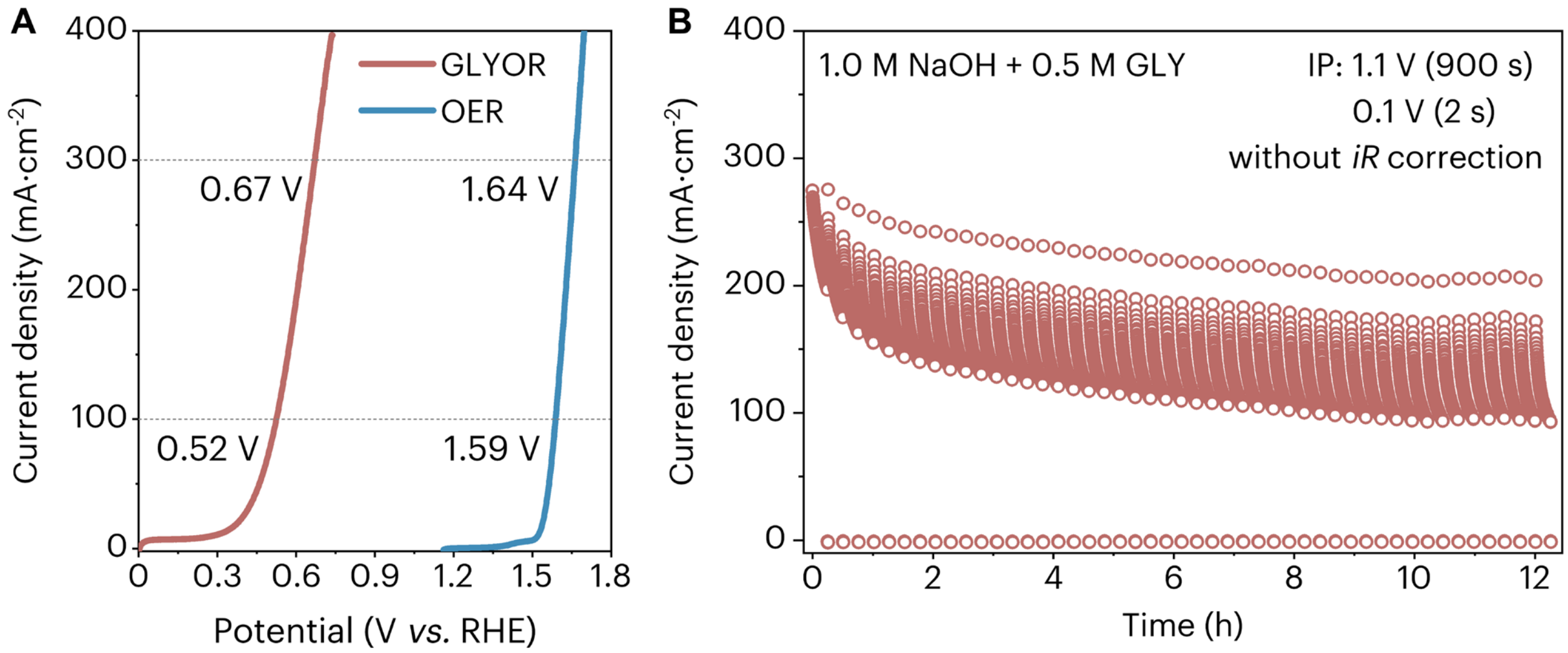

Fig. S34. Electrochemical performance. (**A**) Glycerol oxidation (GLYOR) and OER LSV curves for Pt/NiCo(OH)$_x$. (**B**) GLYOR stability test through IP (1.1 V *vs.* RHE for 900 s, 0.1 V *vs.* RHE for 2 s) method for Pt/NiCo(OH)$_x$.

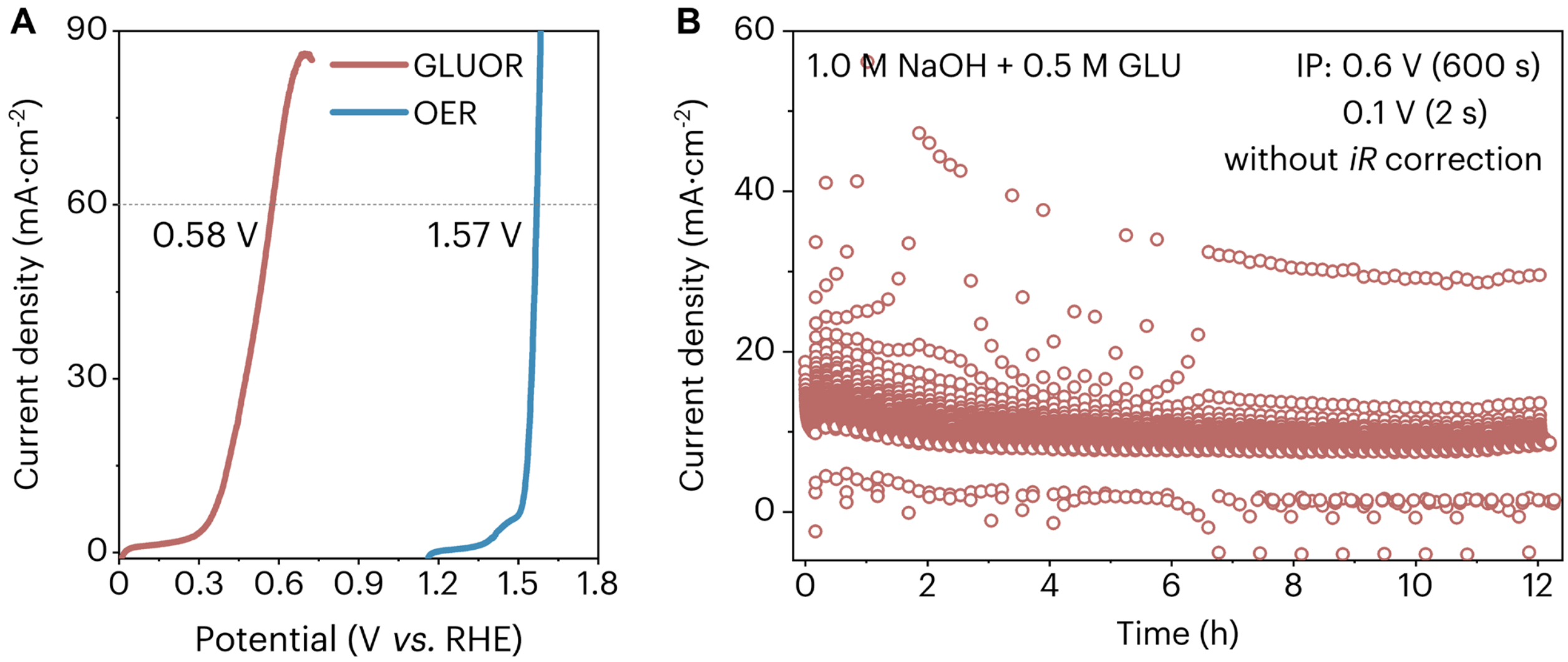

Fig. S35. Electrochemical performance. (**A**) Glucose oxidation (GLUOR) and OER LSV curves for Pt/NiCo(OH)$_x$. (**B**) GLUOR stability test through IP (0.6 V *vs.* RHE for 600 s, 0.1 V *vs.* RHE for 2 s) method for Pt/NiCo(OH)$_x$.

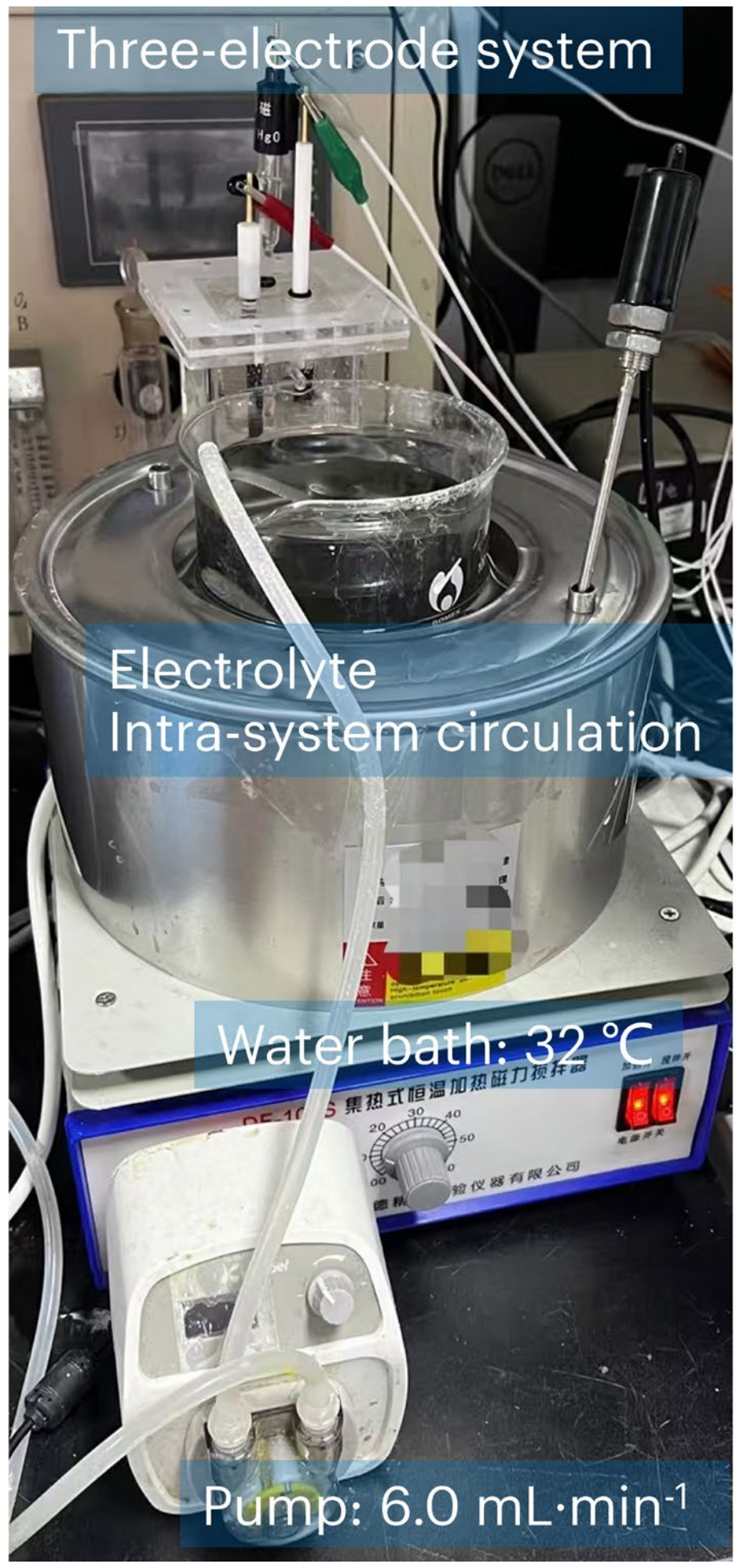

Fig. S36. Reaction device diagram. The digital image of the self-made flow electrolyzer for long-term EGOR/HER stability tests.

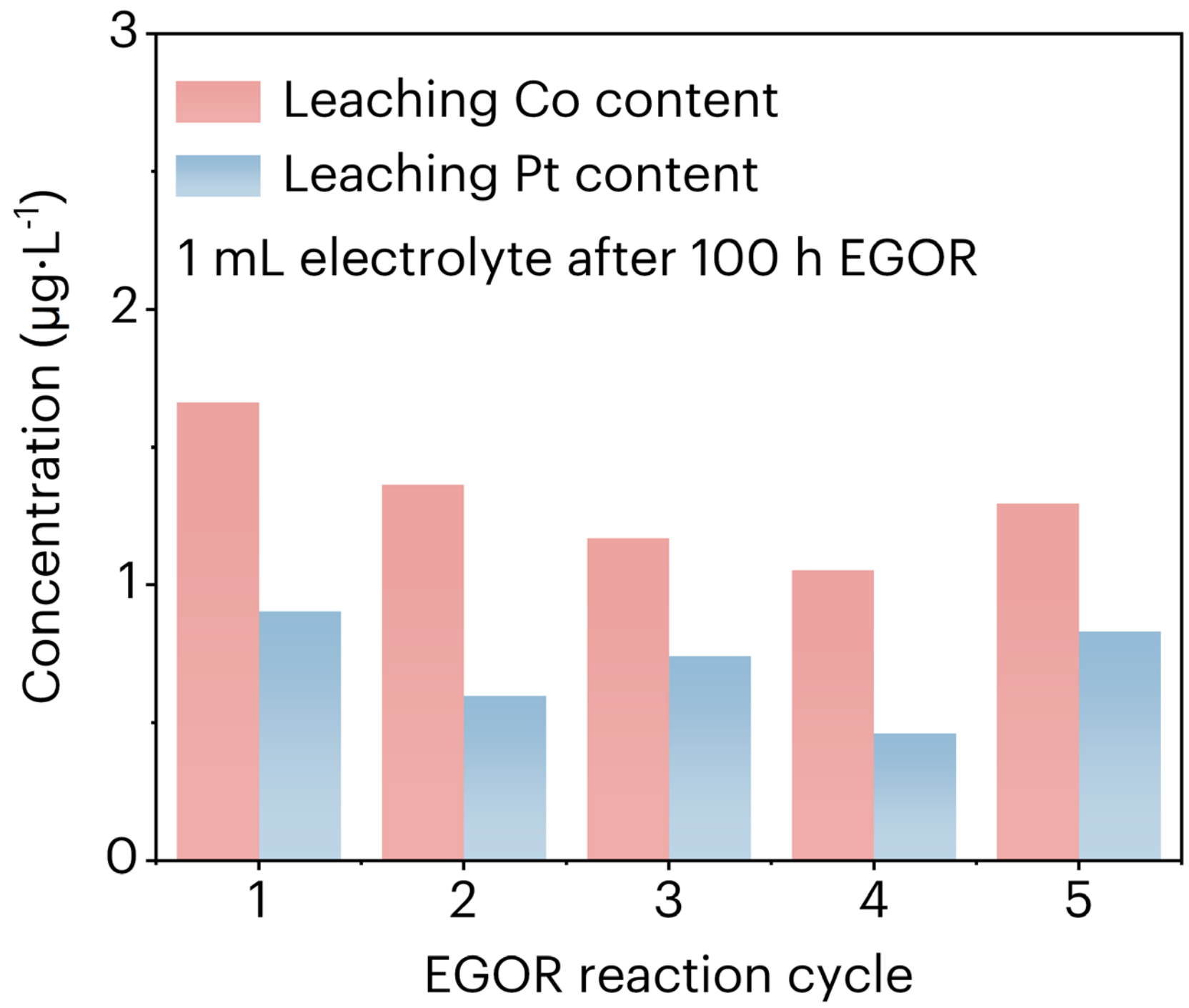

Fig. S37. Leaching contents of Co and Pt for Pt/NiCo(OH)$_x$ (The stable state after sufficient activation) through inductively coupled plasma-mass spectrometry (ICP-MS) test in random 5 batches of 100 h EGOR stability test.

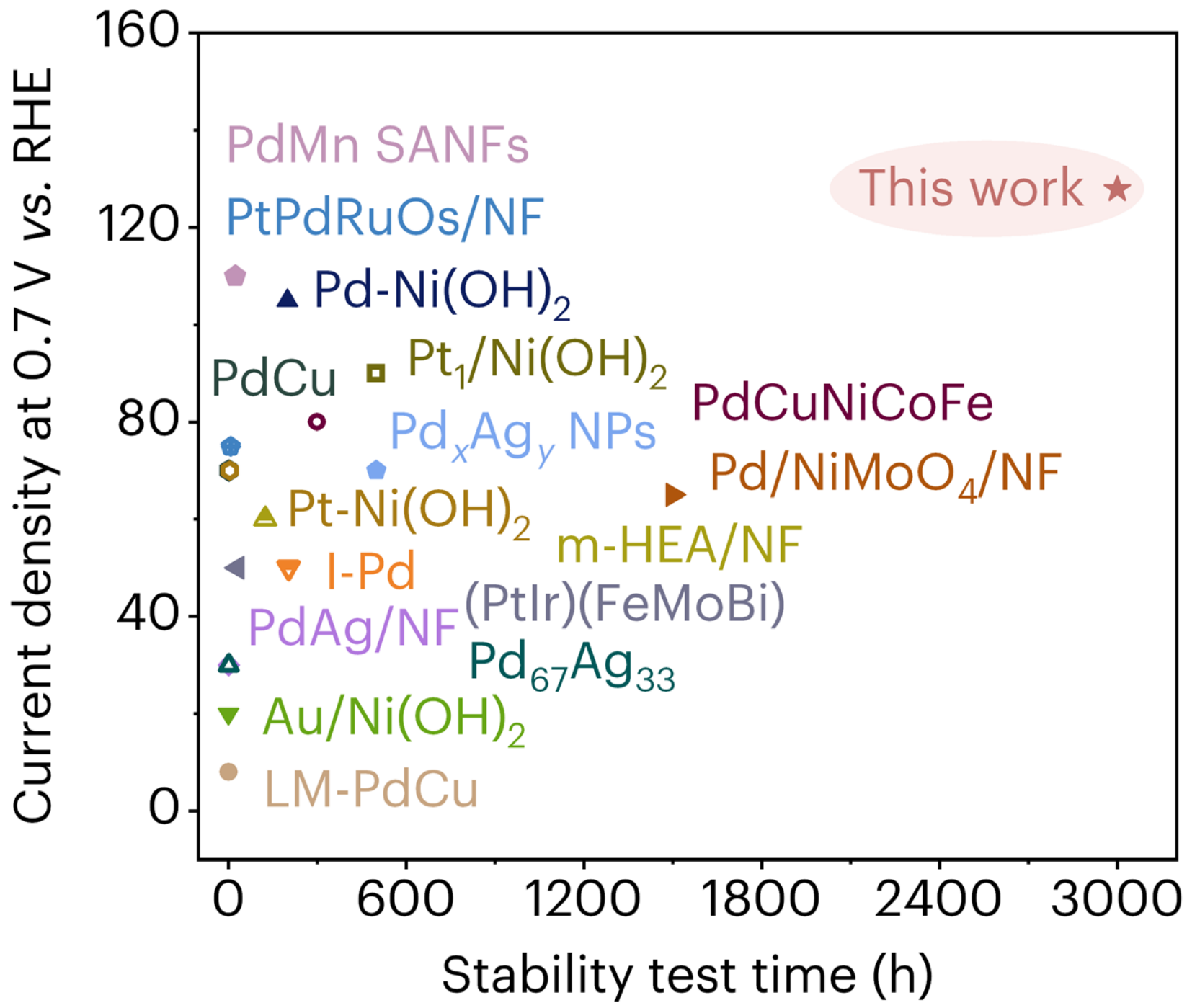

Fig. S38. EGOR activity comparison. Comparison of EGOR activity (without *iR* correction) for Pt/NiCo(OH)$_x$ with recently reported efficient noble-metal-based catalysts.

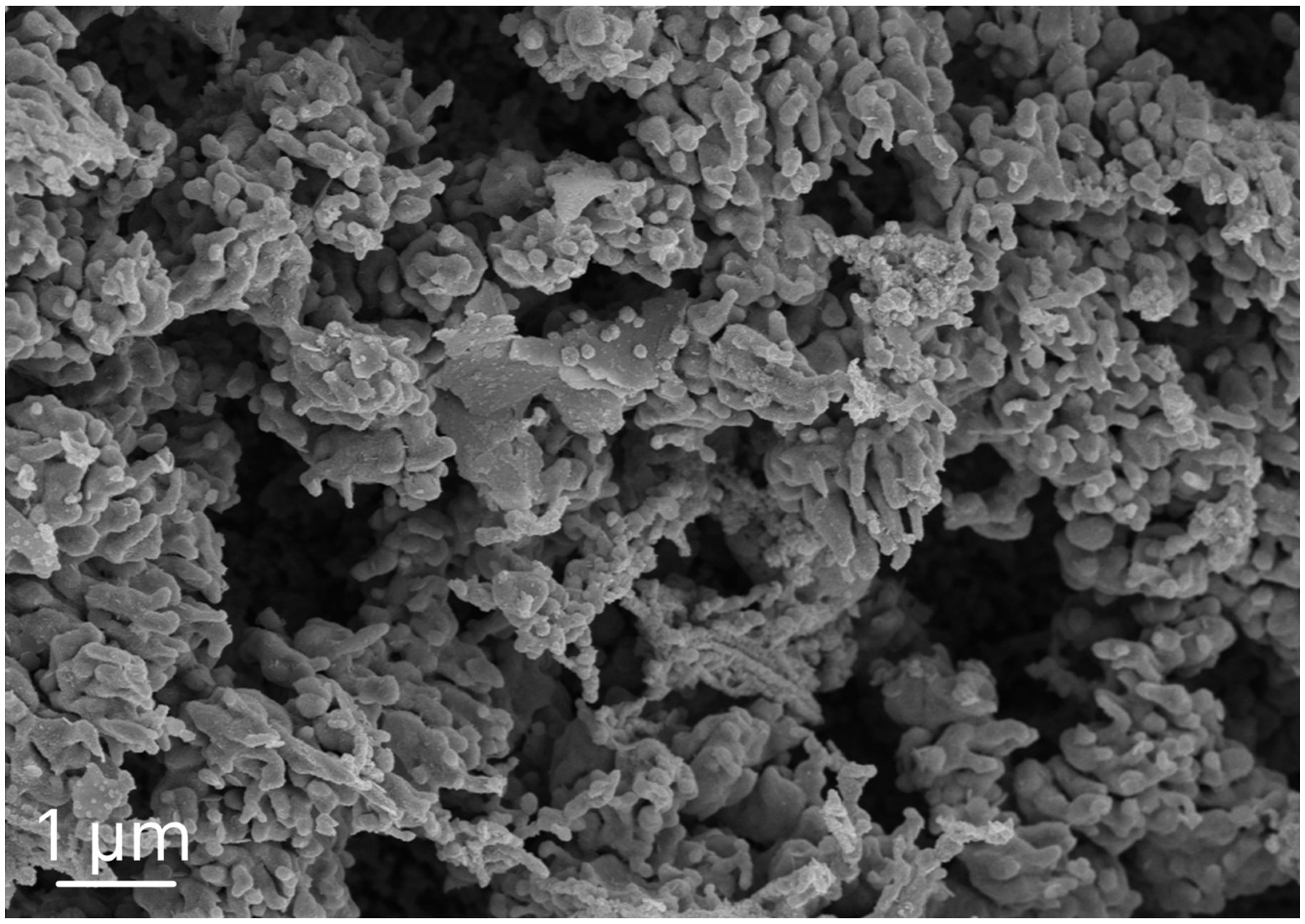

Fig. S39. Structural analysis. SEM image of Pt/NiCo(OH)$_x$ after EGOR stability test.

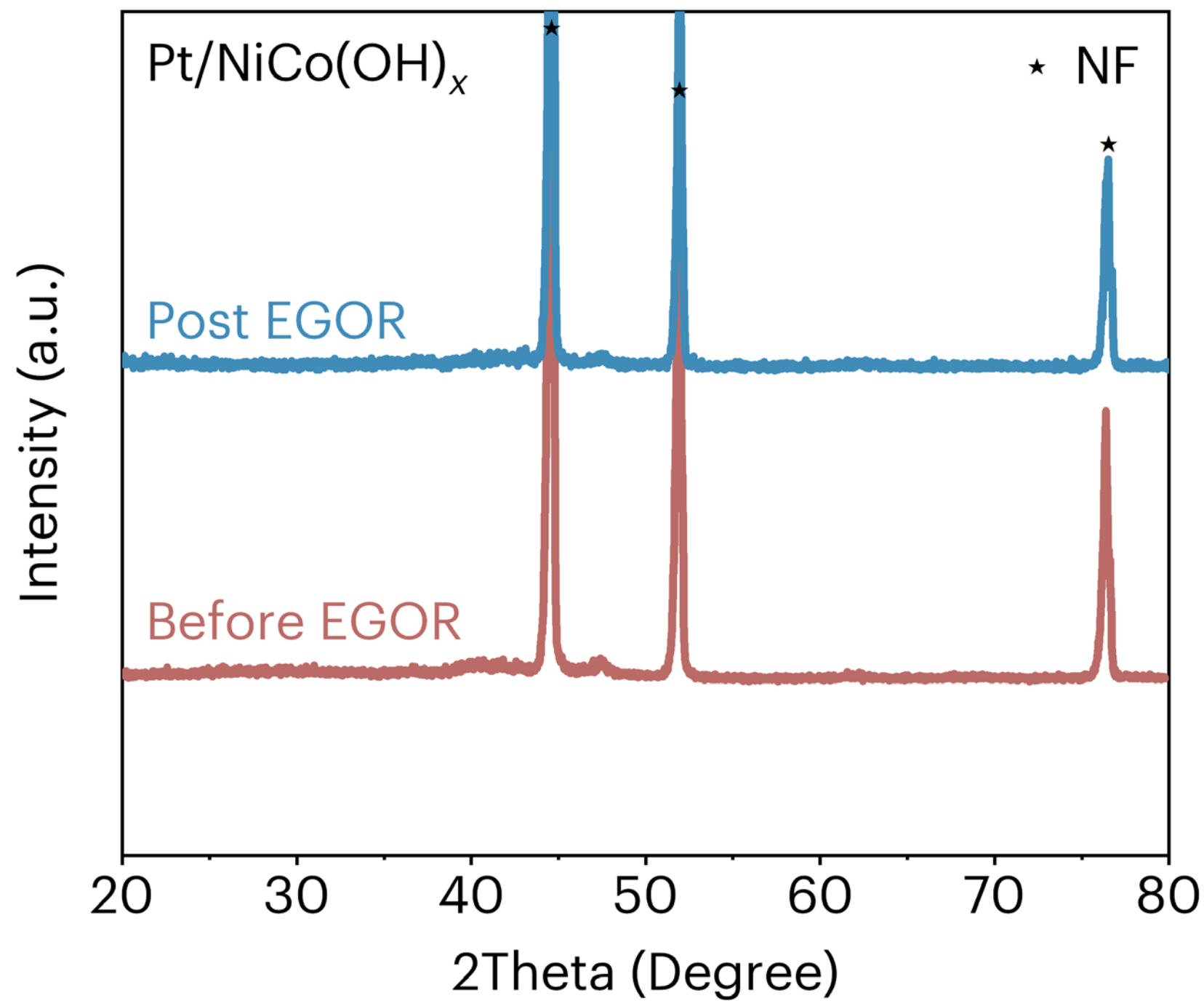

Fig. S40. Structural analysis. XRD patterns of Pt/NiCo(OH)$_x$ before and after EGOR stability test.

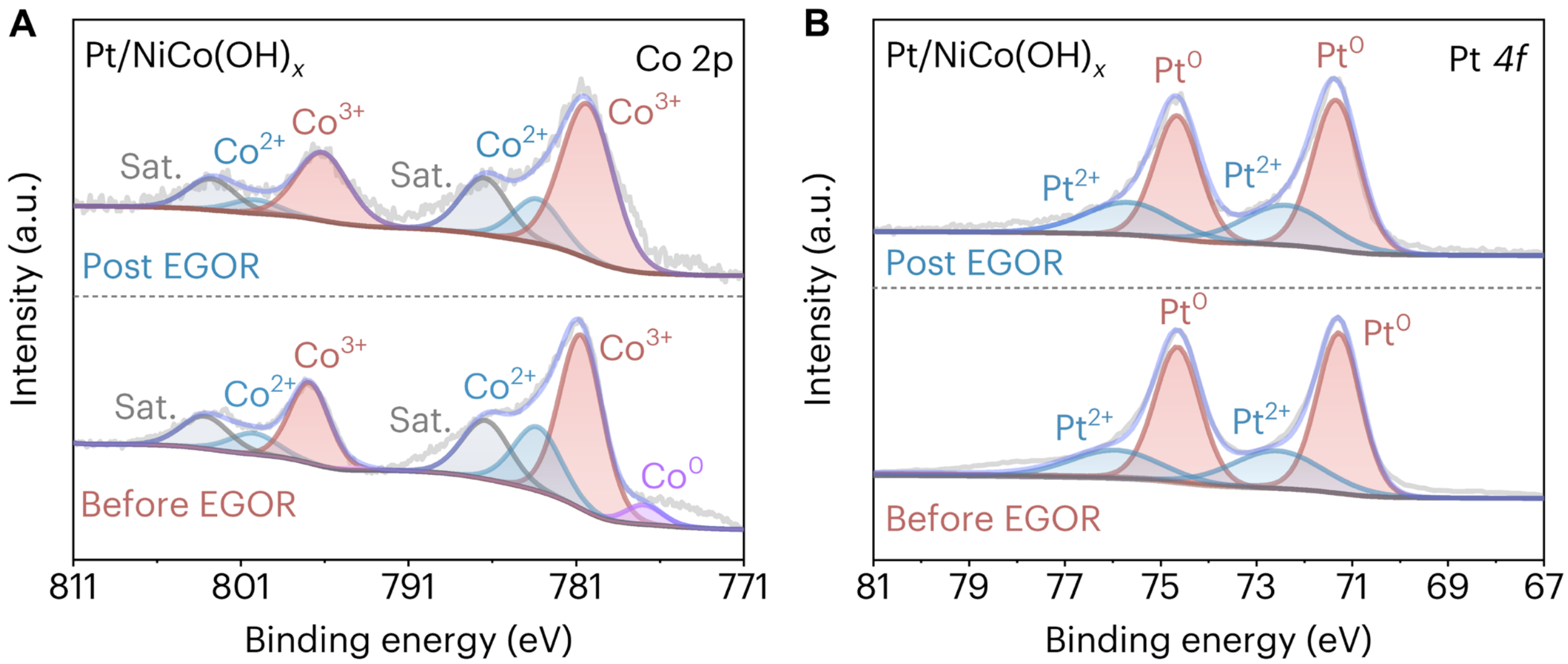

Fig. S41. Valence analysis. High-resolution (**A**) Co 2p and (**B**) Pt 4*f* XPS spectra of Pt/NiCo$(OH)_x$ before and after EGOR stability test.

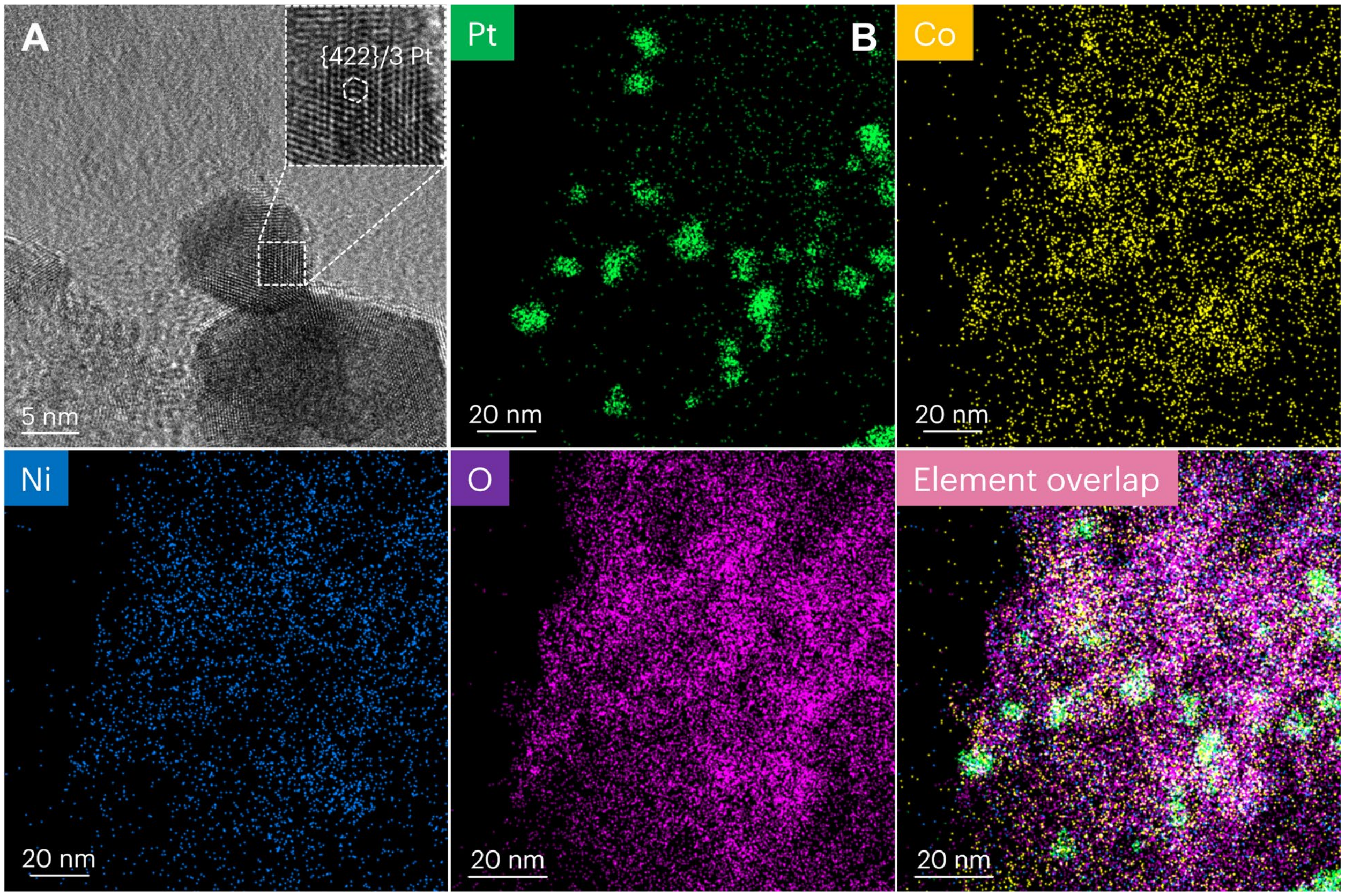

Fig. S42. Structural analysis. (**A**) High-resolution TEM image and (**B**) EDS elemental mapping images for Pt, Co, Ni and O of Pt/NiCo(OH)$_x$ after EGOR stability test.

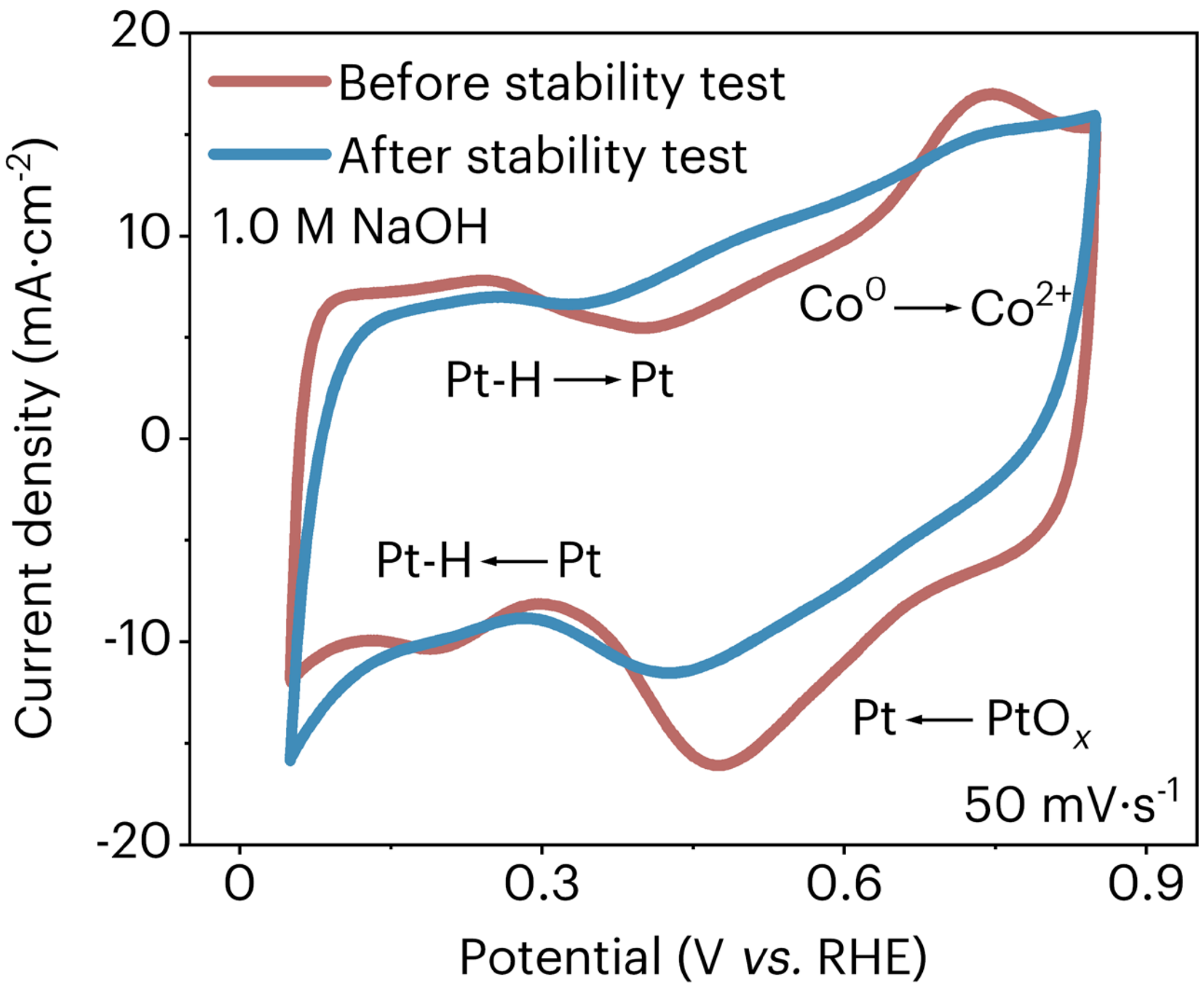

Fig. S43. Electrochemical active site analysis. CV curves of Pt/NiCo(OH)$_x$ in 1.0 M NaOH before and after EGOR stability test.

Note: ICP-OES test indicates that the Pt mass loading of Pt/NiCo(OH)$_x$ after EGOR stability test is 0.46 $mg_{Pt}\cdot cm^{-2}$. The values of $ECSA_{Pt}$ for Pt/NiCo(OH)$_x$ before and after EGOR stability test are 39.9 and 31.2 $cm^2\cdot mg_{Pt}^{-1}$. Hence, the values of ECSA for Pt/NiCo(OH)$_x$ before and after EGOR stability test are 20.8 and 14.4 cm^2. Due to the long-term operation under alkaline oxidation conditions, the oxidation peak of Co^0 to Co^{2+} is difficult to observe, which is consistent with the above XPS results.

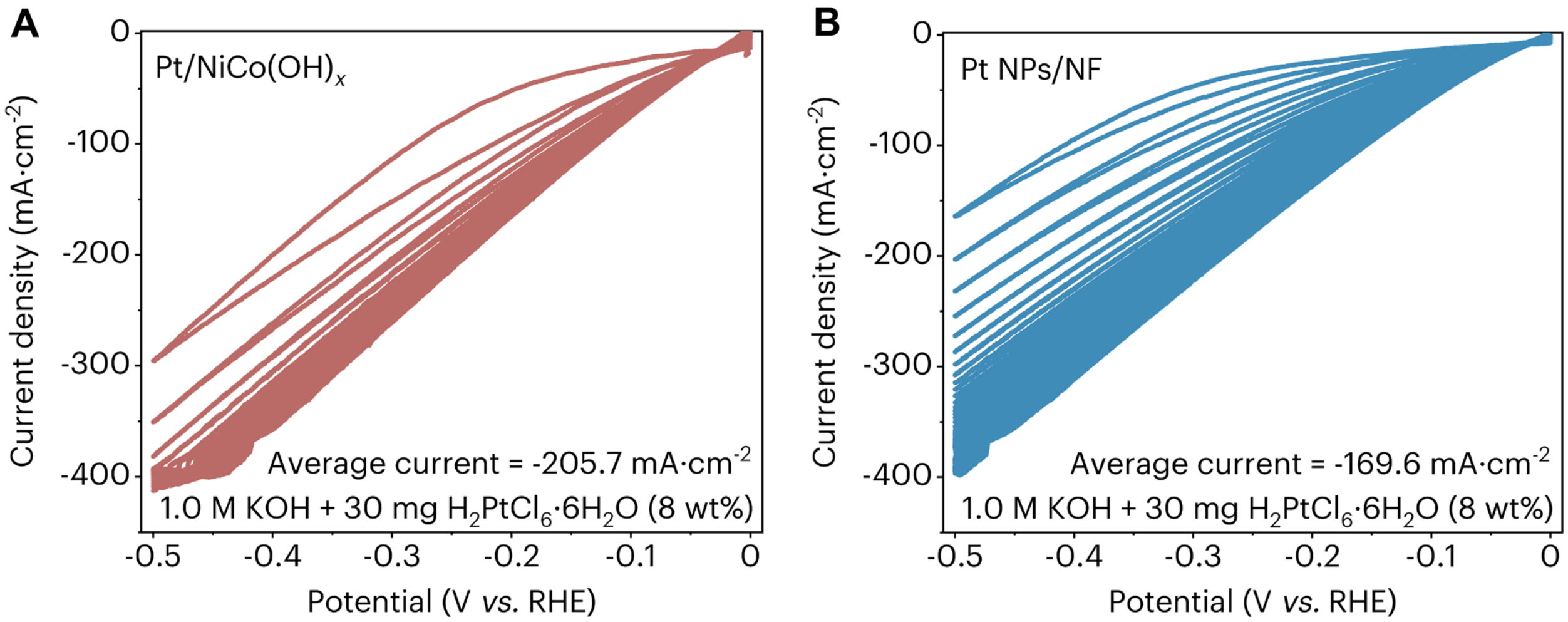

Fig. S44. Electrochemical performance. CV curves of the electro-deposition process for (**A**) Pt/NiCo(OH)$_x$ and (**B**) Pt NPs/NF.

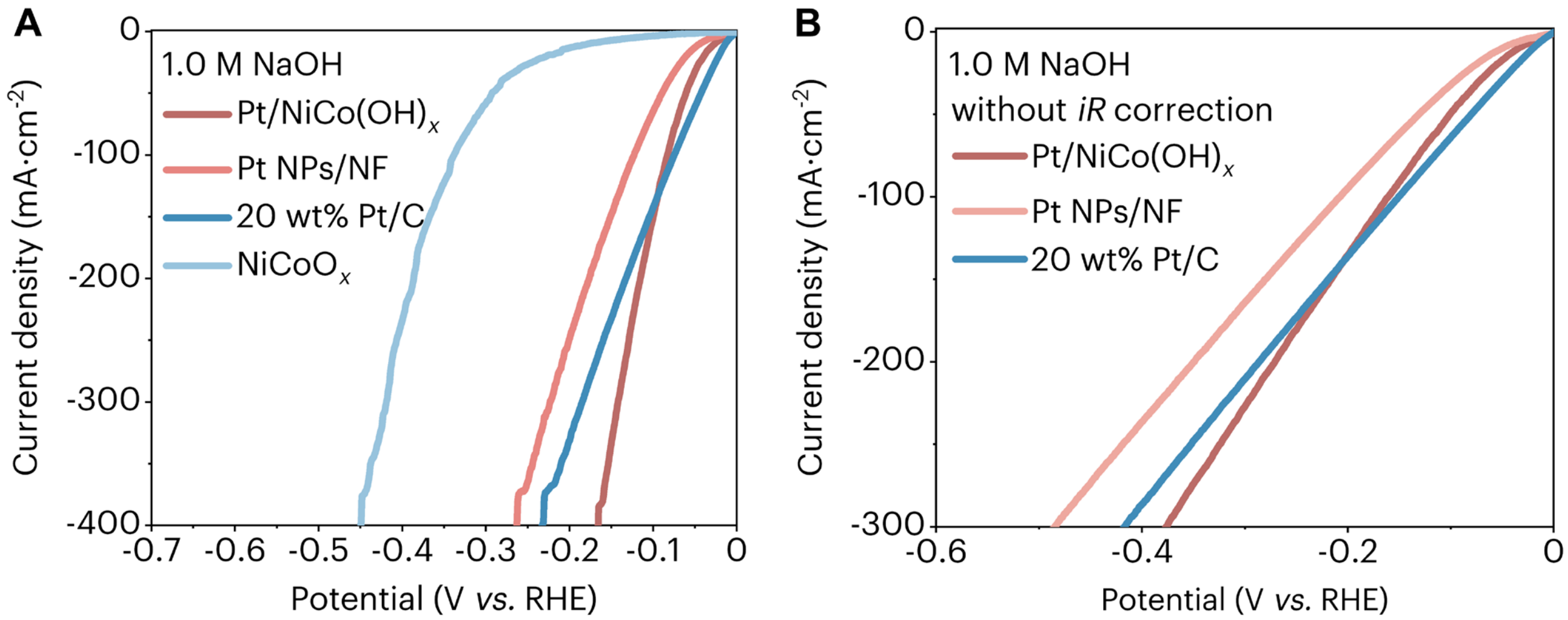

Fig. S45. Electrochemical performance. (**A**) HER LSV curves of Pt/NiCo(OH)$_x$, Pt NPs/NF, 20 wt% Pt/C and NiCoO$_x$ in 1.0 M NaOH (normalized to geometric area). (**B**) HER LSV curves of Pt/NiCo(OH)$_x$, Pt NPs/NF and 20 wt% Pt/C without *iR* correction in 1.0 M NaOH.

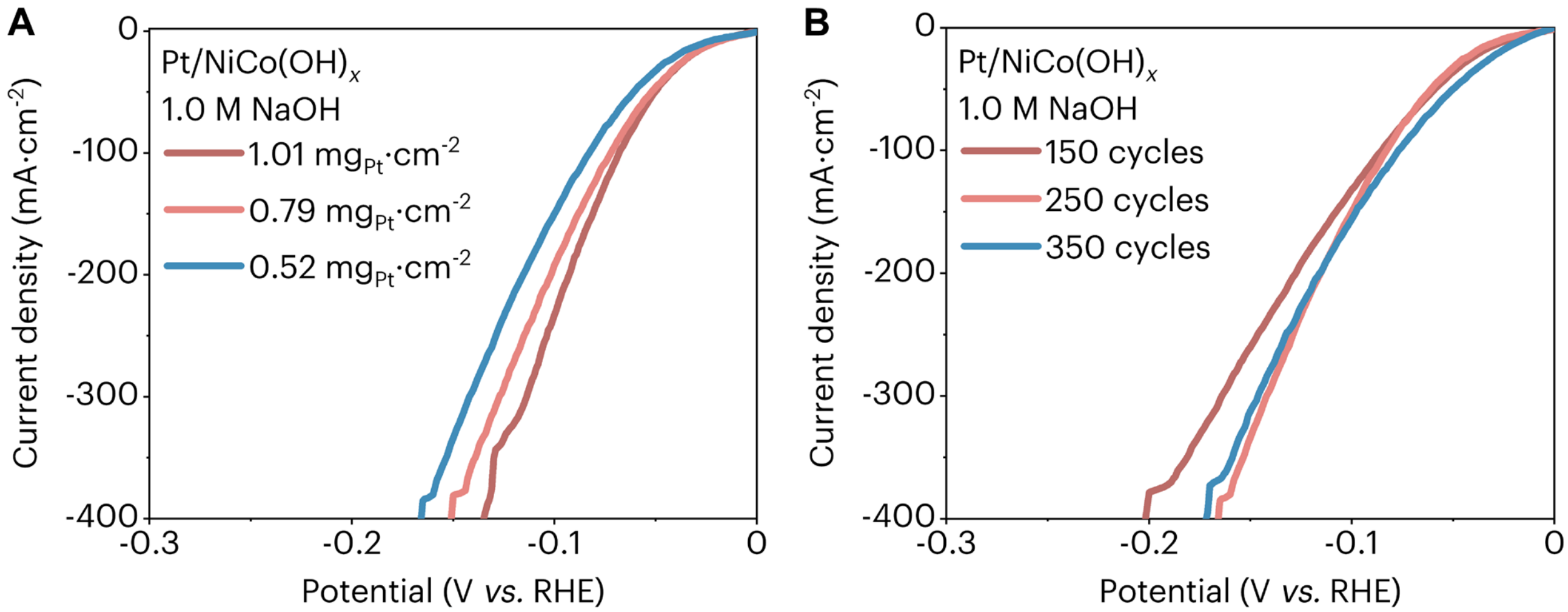

Fig. S46. Electrochemical performance. (**A**) HER LSV curves of Pt/NiCo(OH)$_x$ with different Pt mass loadings. (**B**) HER LSV curves of Pt/NiCo(OH)$_x$ after different electrodeposition cycles.

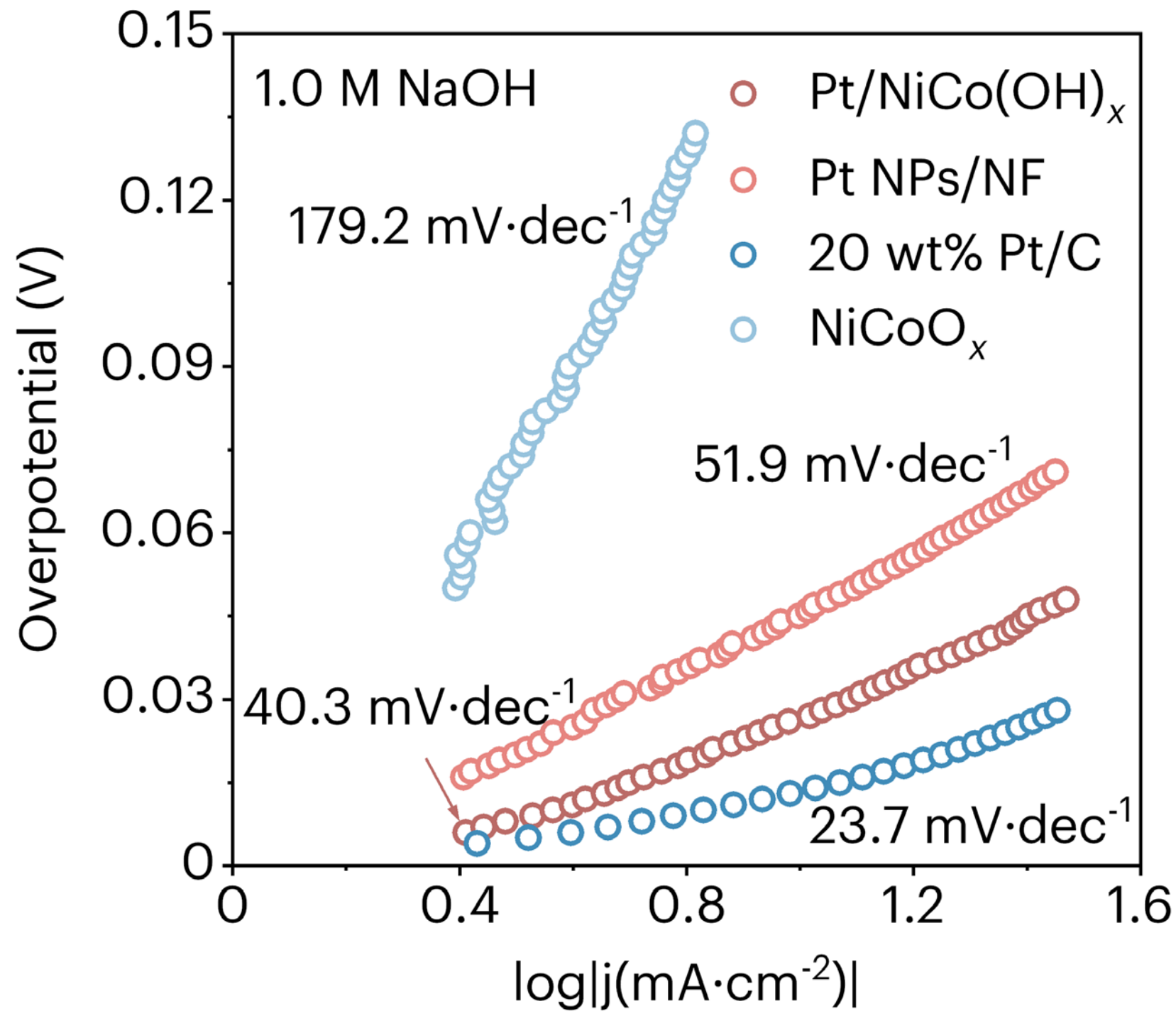

Fig. S47. Electrochemical performance. Tafel plots of Pt/NiCo(OH)$_x$, NiCoO$_x$, Pt NPs/NF and 20 wt% Pt/C for HER in 1.0 M NaOH.

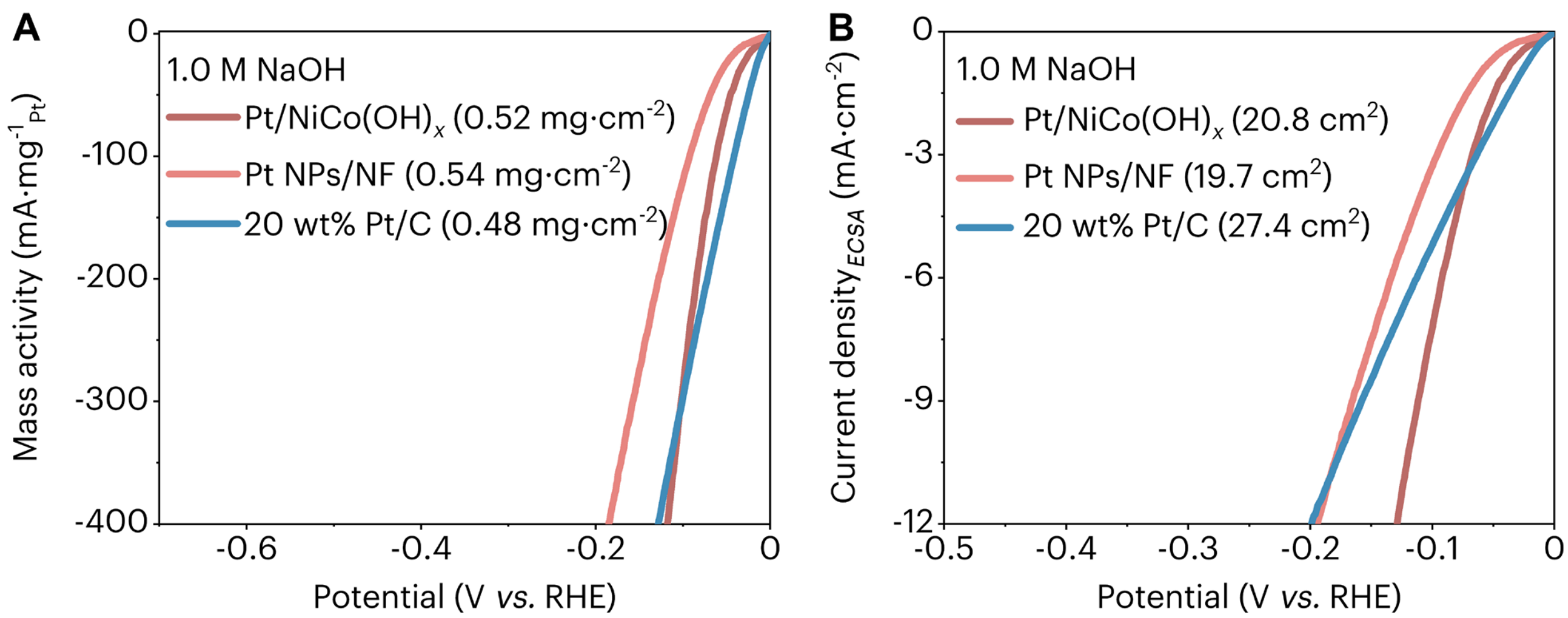

Fig. S48. Electrochemical active site analysis. (**A**) Pt mass loading corrected HER LSV curves of Pt/NiCo(OH)$_x$, Pt NPs/NF and 20 wt% Pt/C. (**B**) ECSA corrected HER LSV curves of Pt/NiCo(OH)$_x$, Pt NPs/NF and 20 wt% Pt/C.

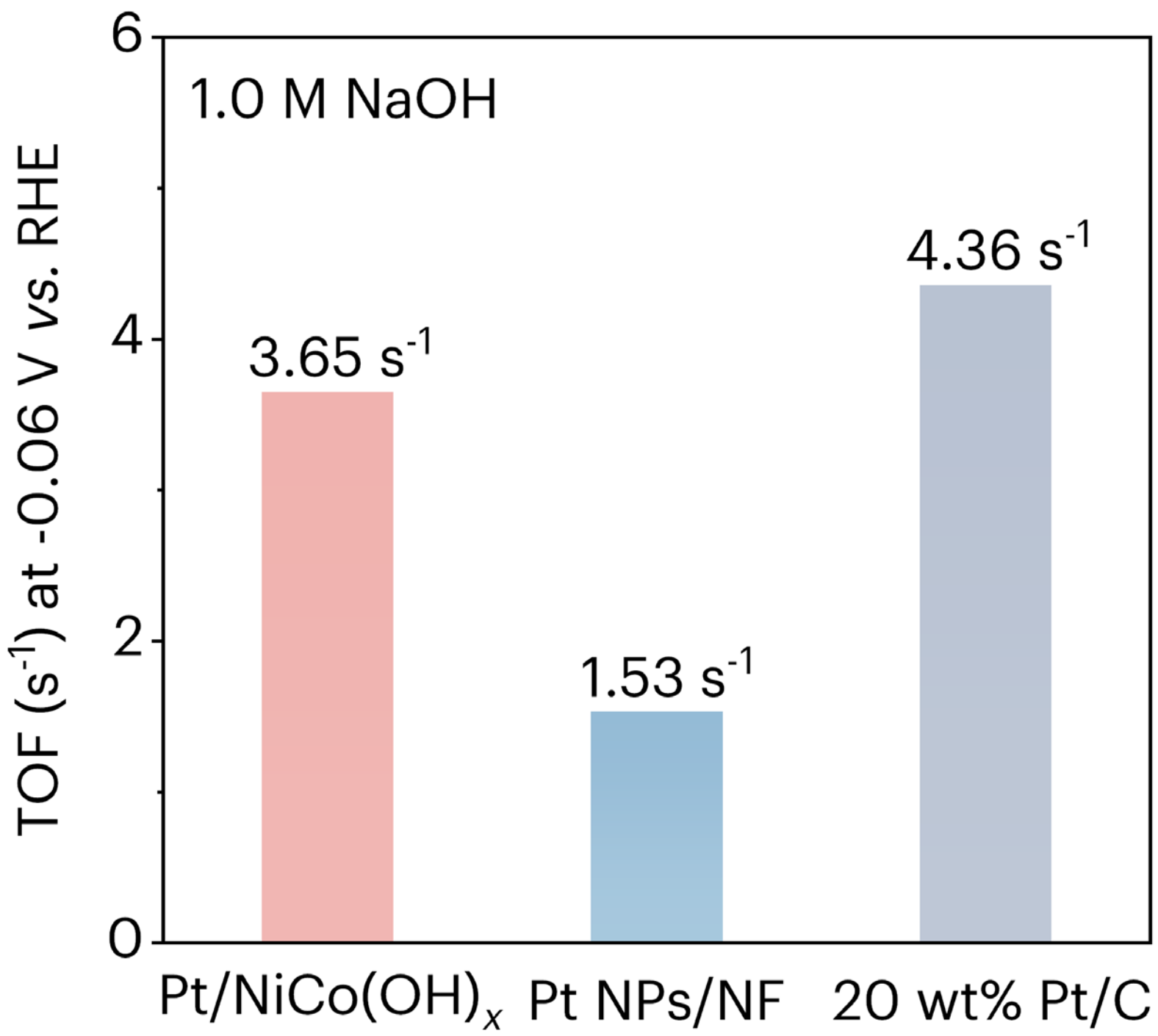

Fig. S49. Electrochemical active site analysis. HER TOF values of Pt sites for Pt/NiCo(OH)$_x$, Pt NPs/NF and 20 wt% Pt/C at -0.06 V *vs.* RHE.

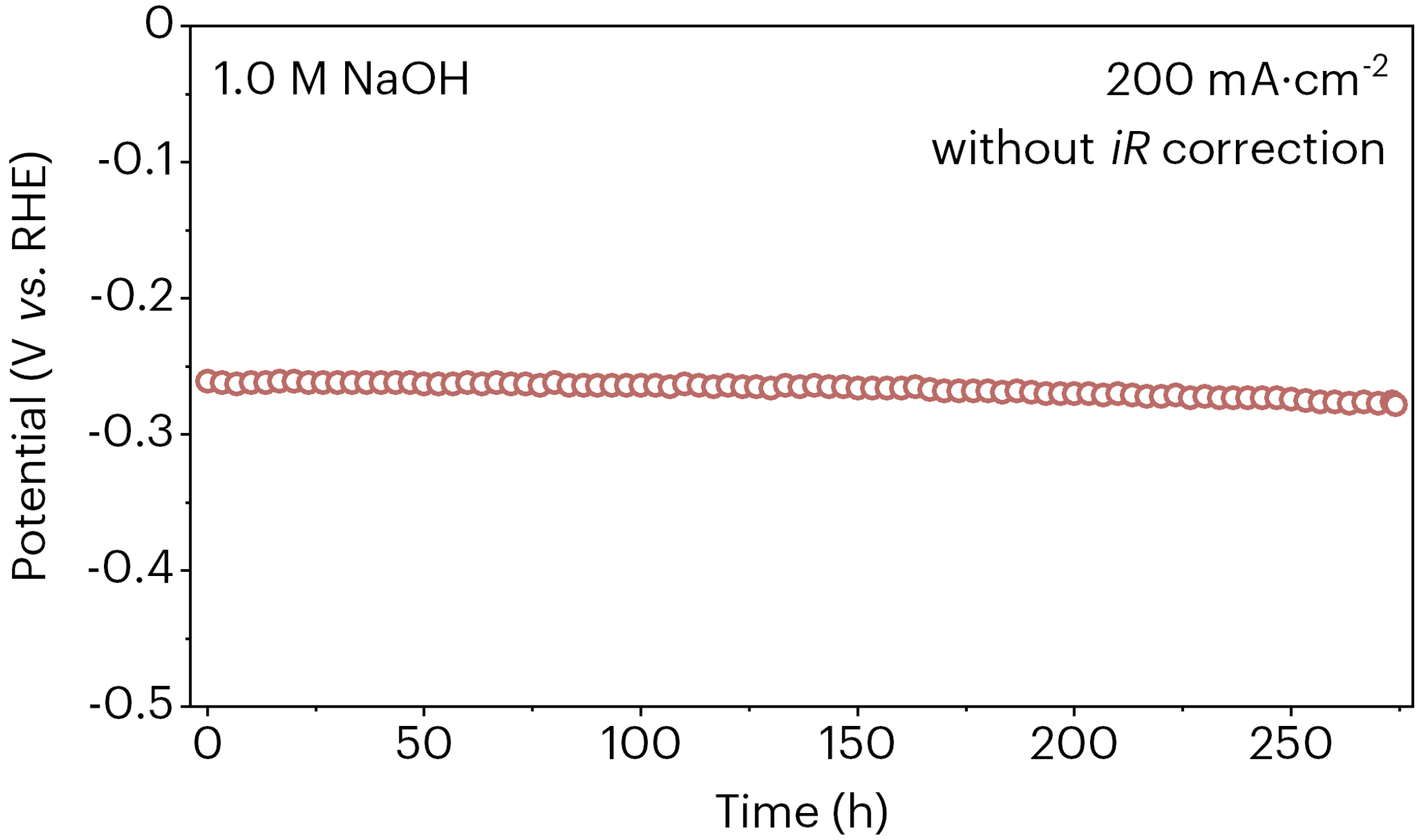

Fig. S50. Stability analysis. HER stability test of Pt/NiCo(OH)$_x$ under current density of 200 mA·cm^{-2} in 1.0 M NaOH.

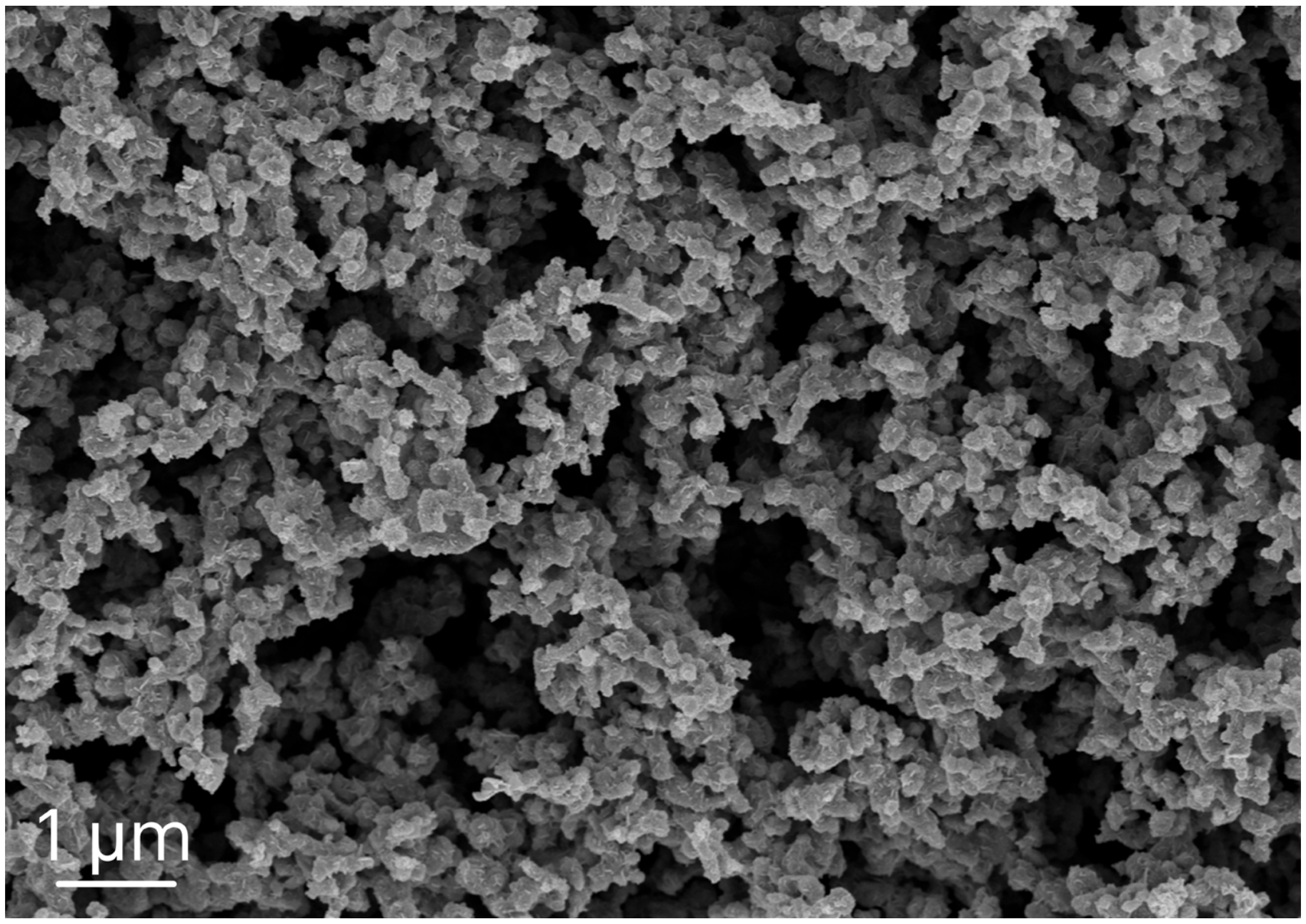

Fig. S51. Structural analysis. SEM image of $Pt/NiCo(OH)_x$ after HER stability test.

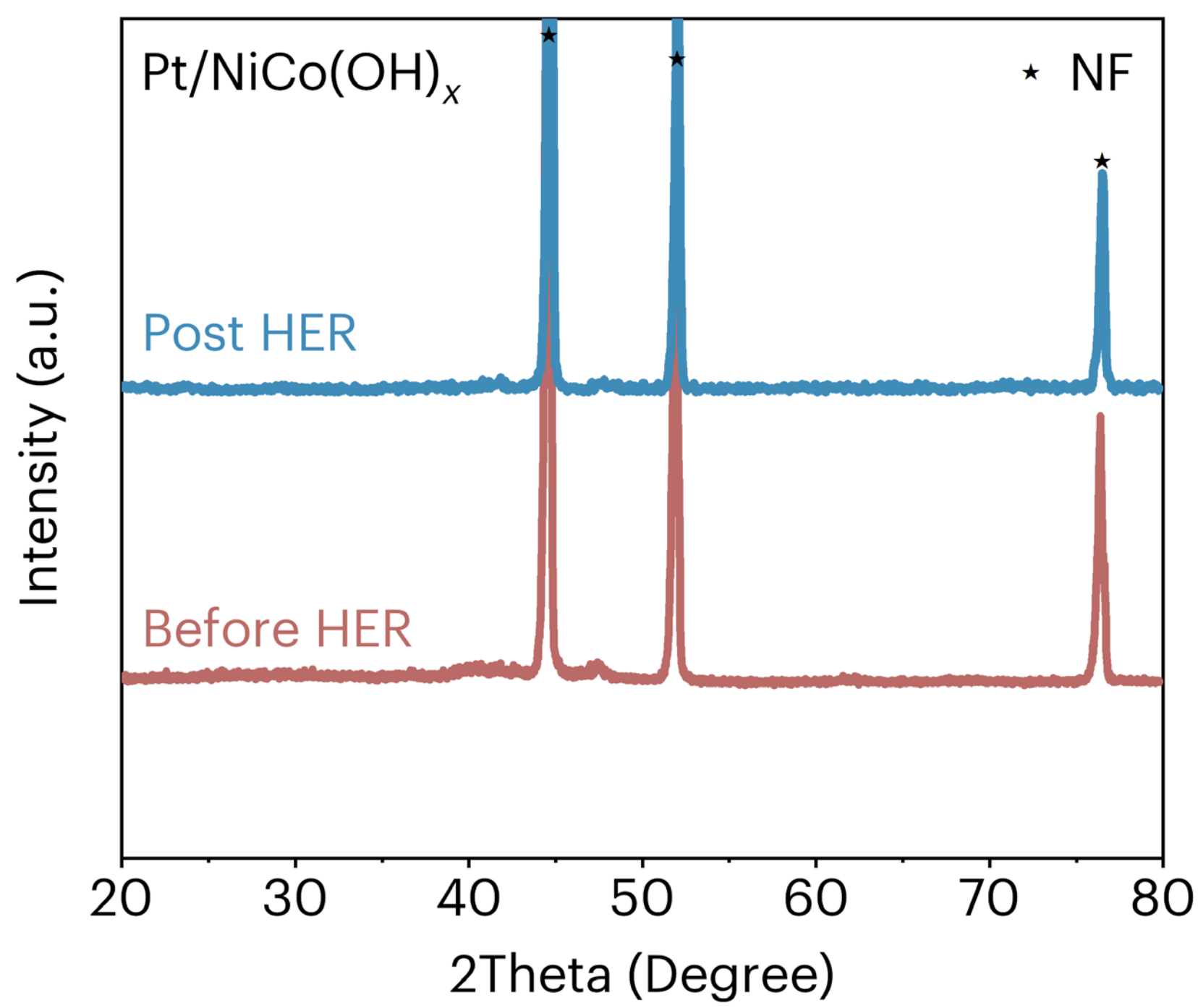

Fig. S52. Structural analysis. XRD patterns of Pt/NiCo(OH)$_x$ before and after HER stability test.

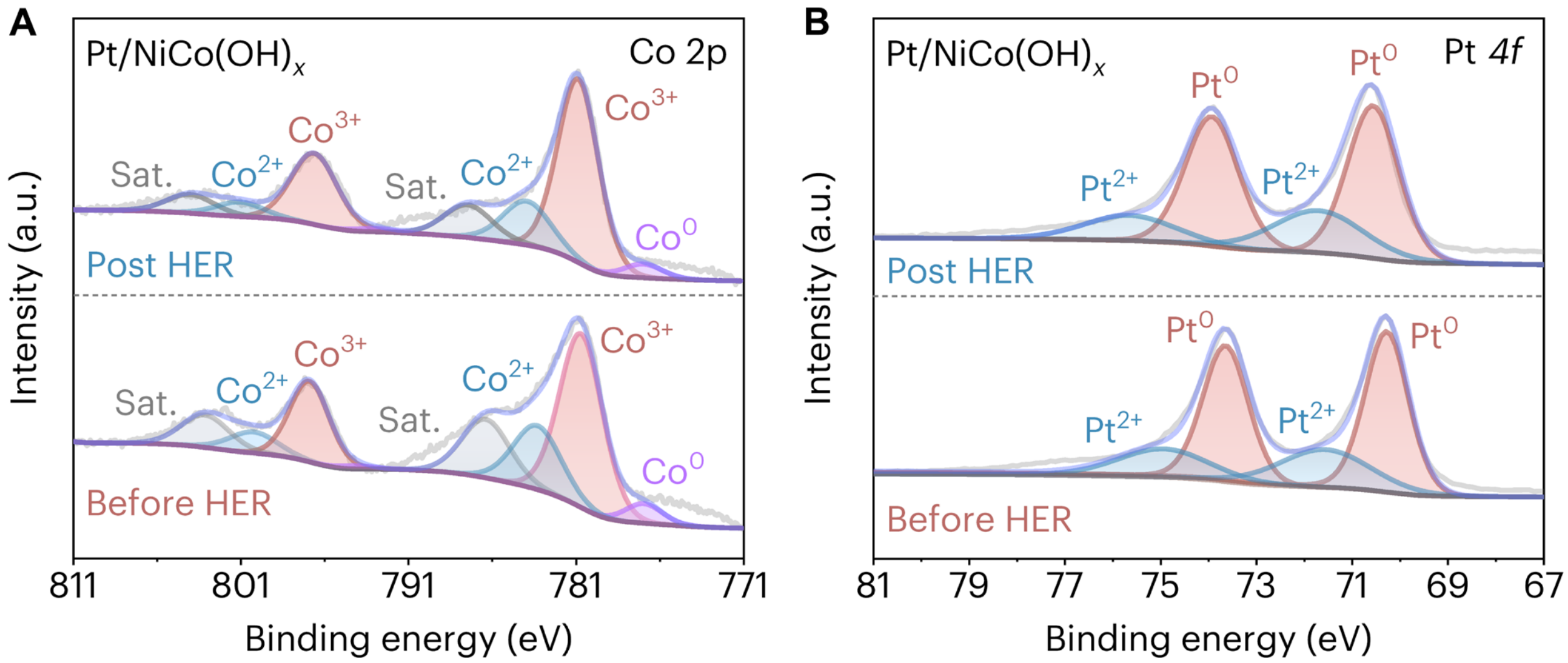

Fig. S53. Valence analysis. High-resolution (**A**) Co 2p and (**B**) Pt 4*f* XPS spectra of Pt/NiCo$(OH)_x$ before and after HER stability test.

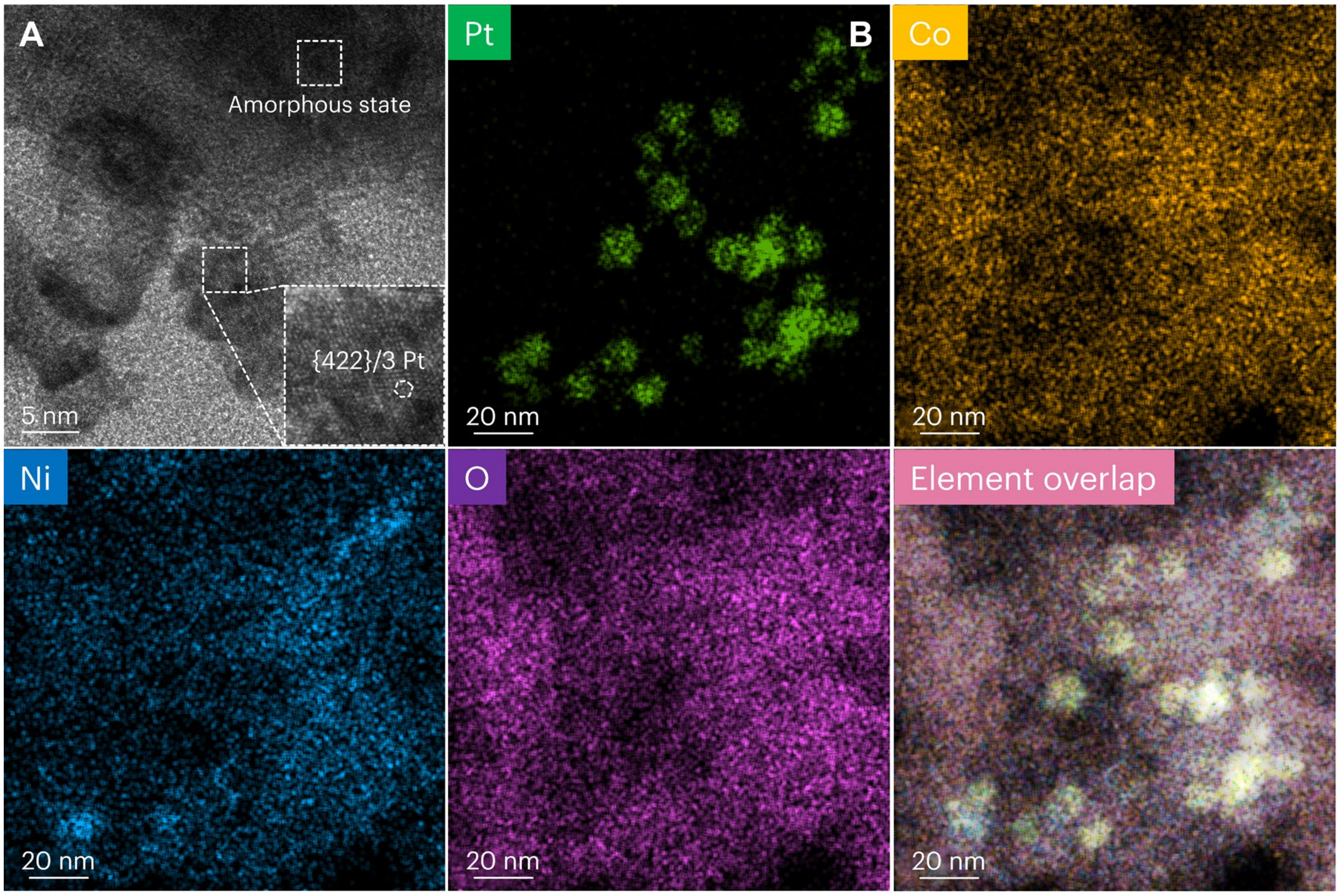

Fig. S54. Structural analysis. (**A**) High-resolution TEM image and (**B**) EDS elemental mapping images for Pt, Co, Ni and O of Pt/NiCo(OH)$_x$ after HER stability test.

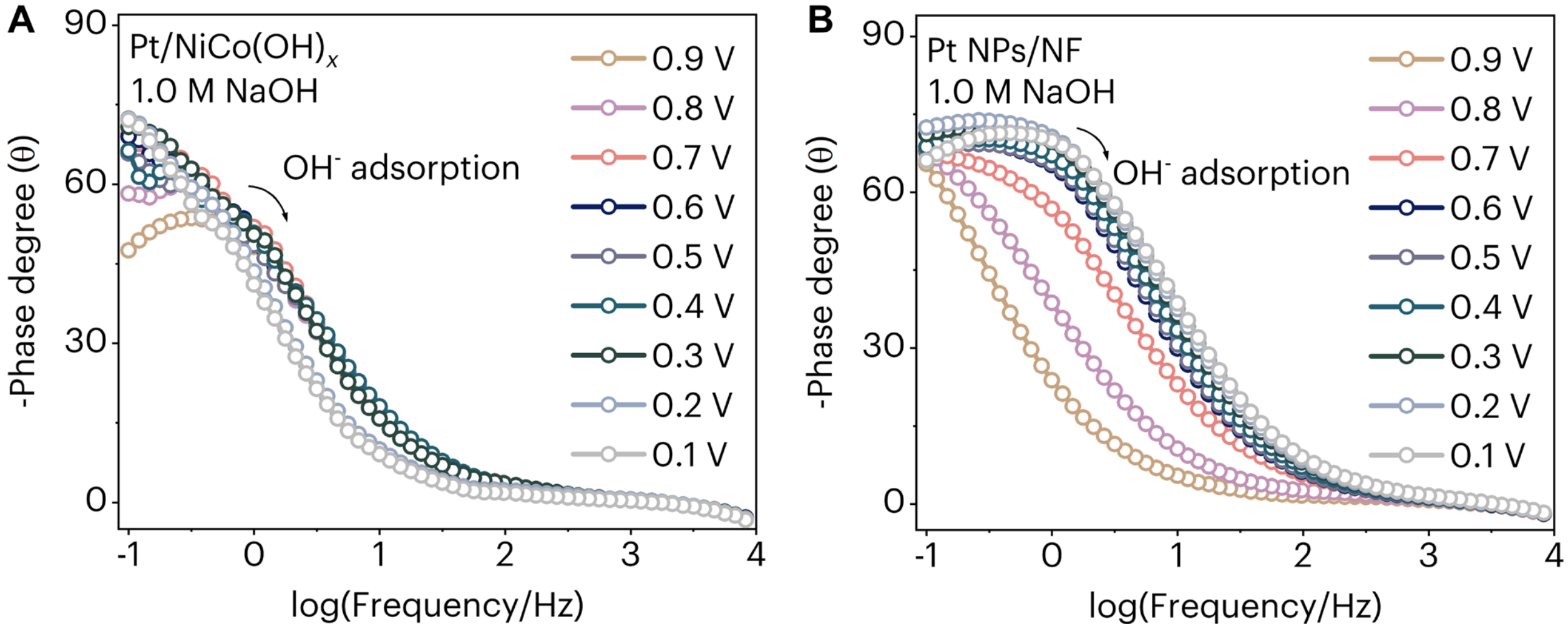

Fig. S55. Impedance analysis. *In-situ* Bode plots of (**A**) Pt/NiCo(OH)$_x$ and (**B**) Pt NPs/NF in 1.0 M NaOH.

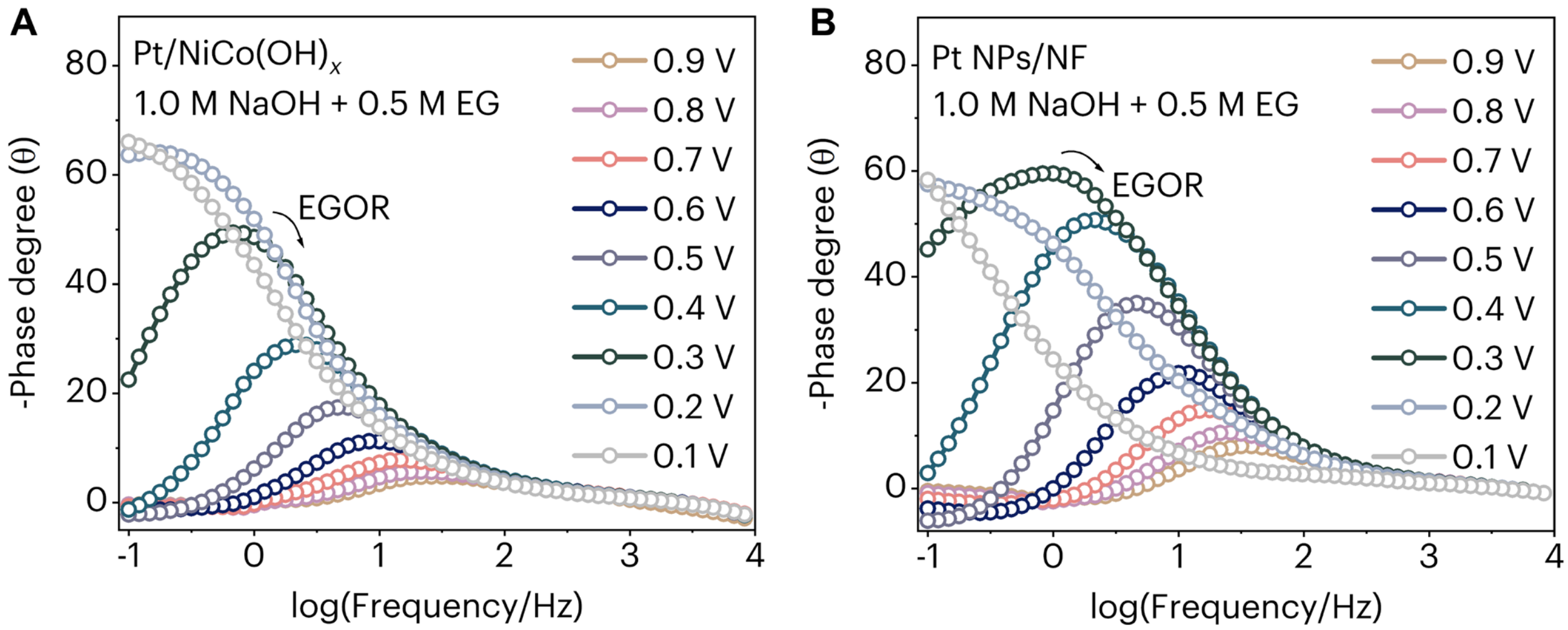

Fig. S56. Impedance analysis. *In-situ* Bode plots of (**A**) Pt/NiCo(OH)$_x$ and (**B**) Pt NPs/NF in 1.0 M NaOH with 0.5 M EG.

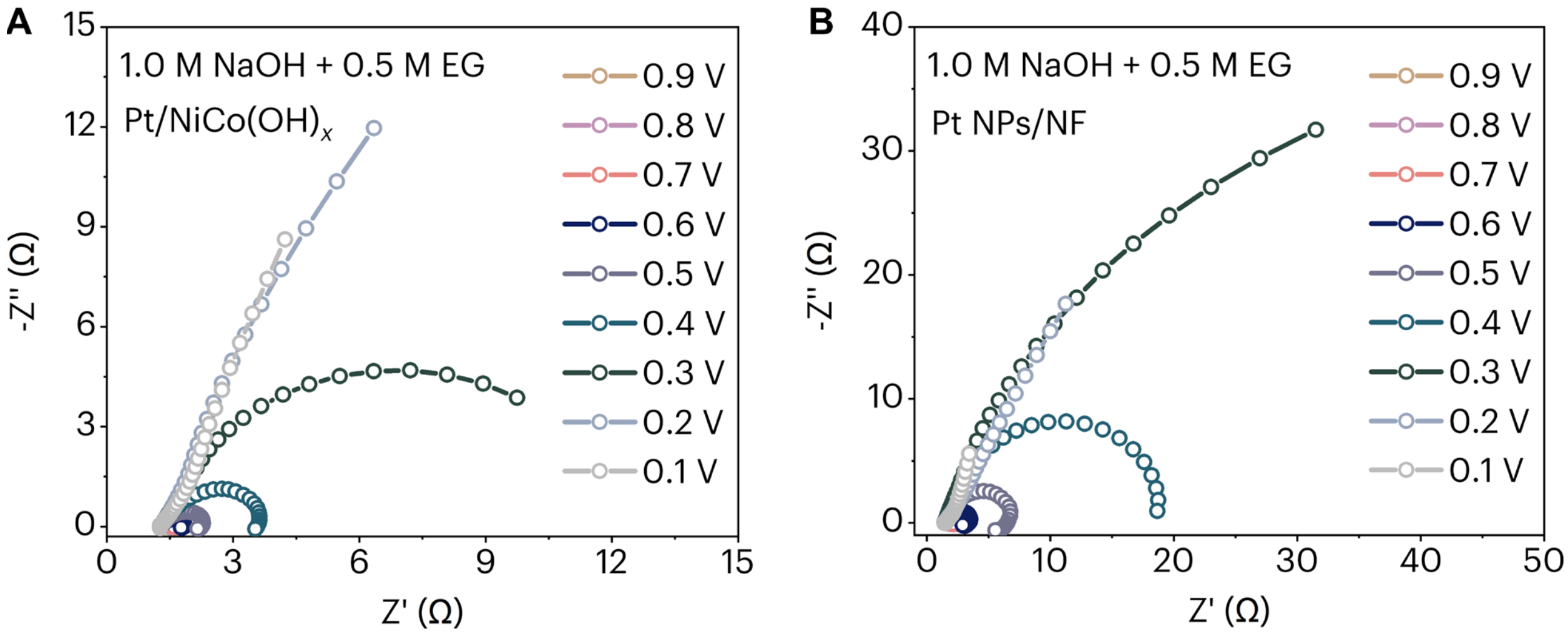

Fig. S57. Impedance analysis. *In-situ* Nyquist plots (without fitting on equivalent circuit) of (**A**) Pt/NiCo(OH)$_x$ and (**B**) Pt NPs/NF in 1.0 M NaOH with 0.5 M EG.

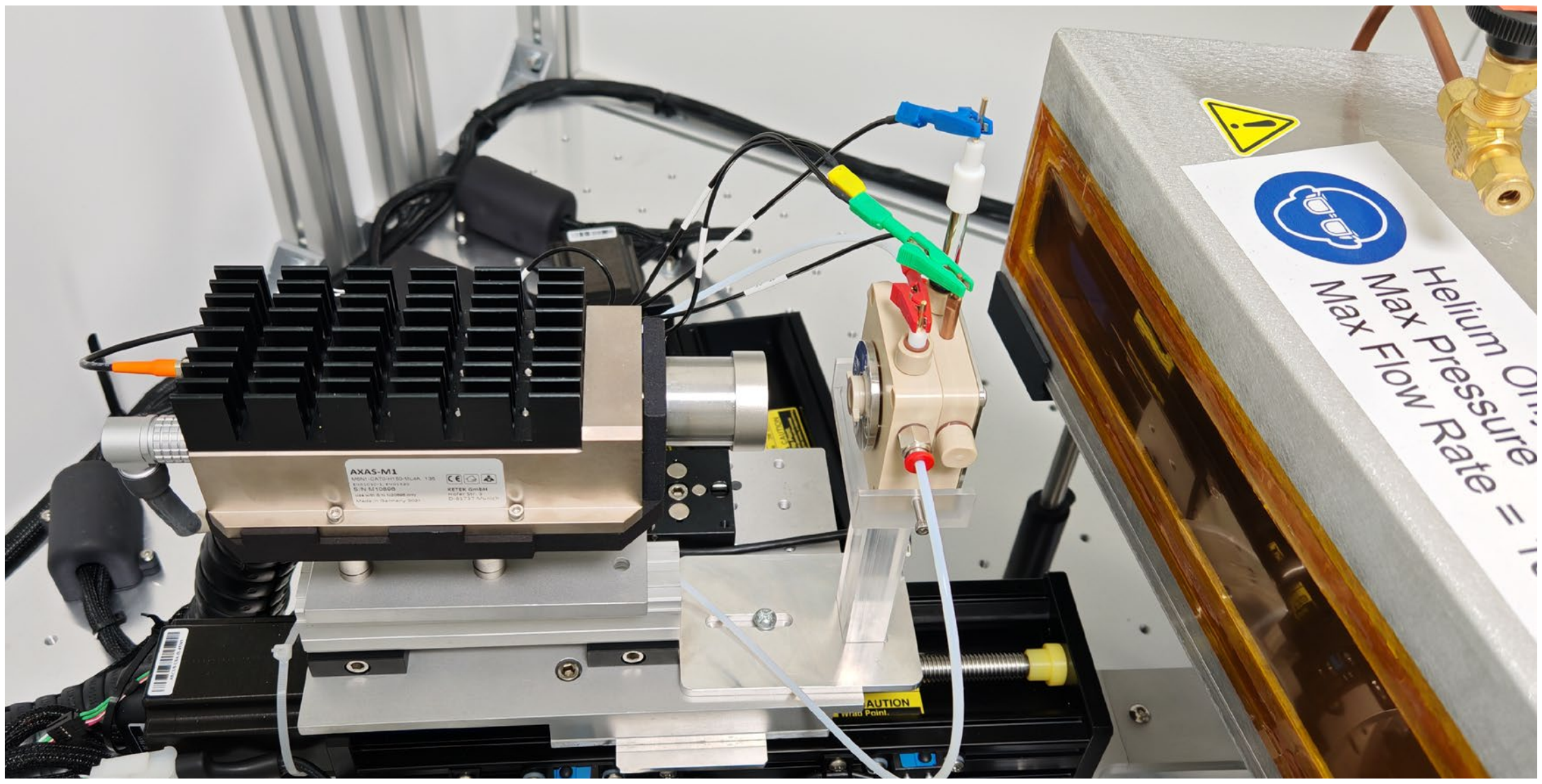

Fig. S58. Valence and coordination analysis. Schematic diagram of the flow electrolyzer for the *in-situ* EXAFS test.

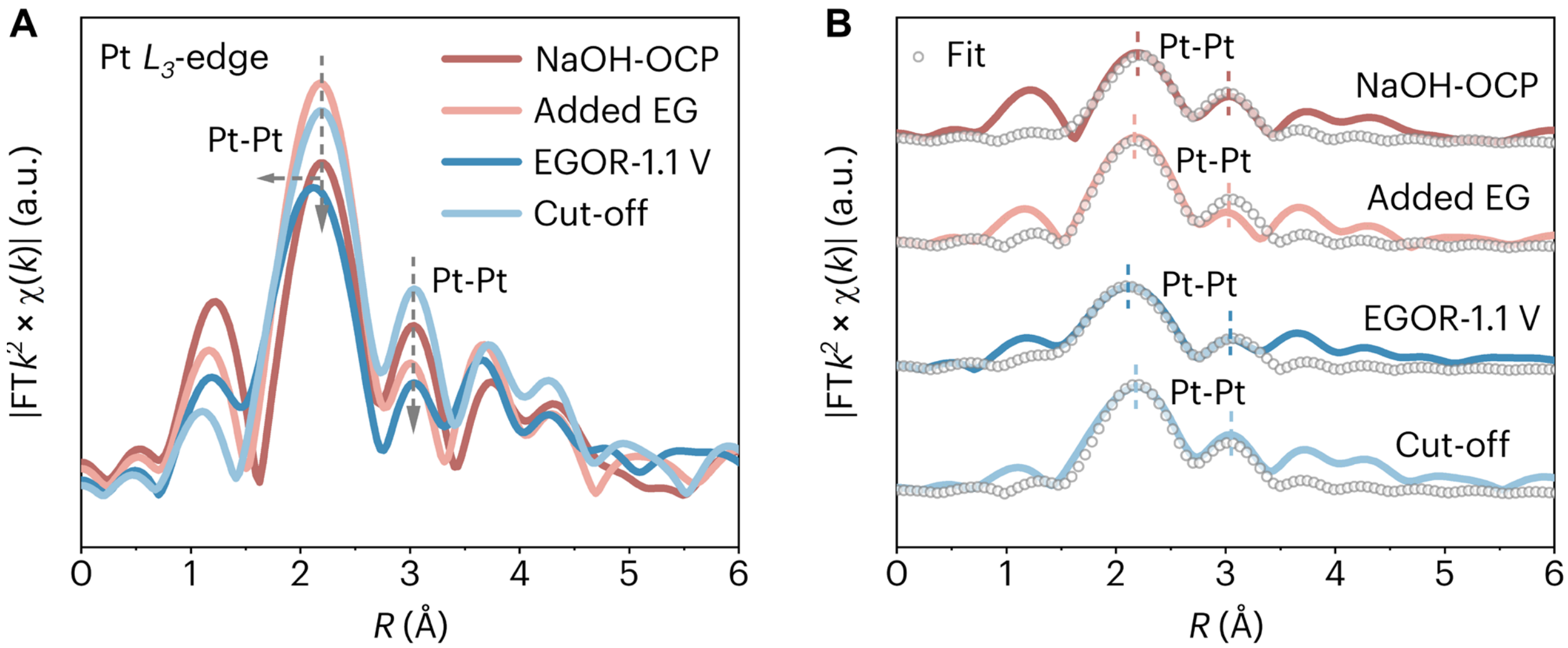

Fig. S59. Coordination analysis. (**A**) Fourier-transform Pt L_3-edge with (**B**) corresponding fitting *in-situ* EXAFS spectra under different conditions.

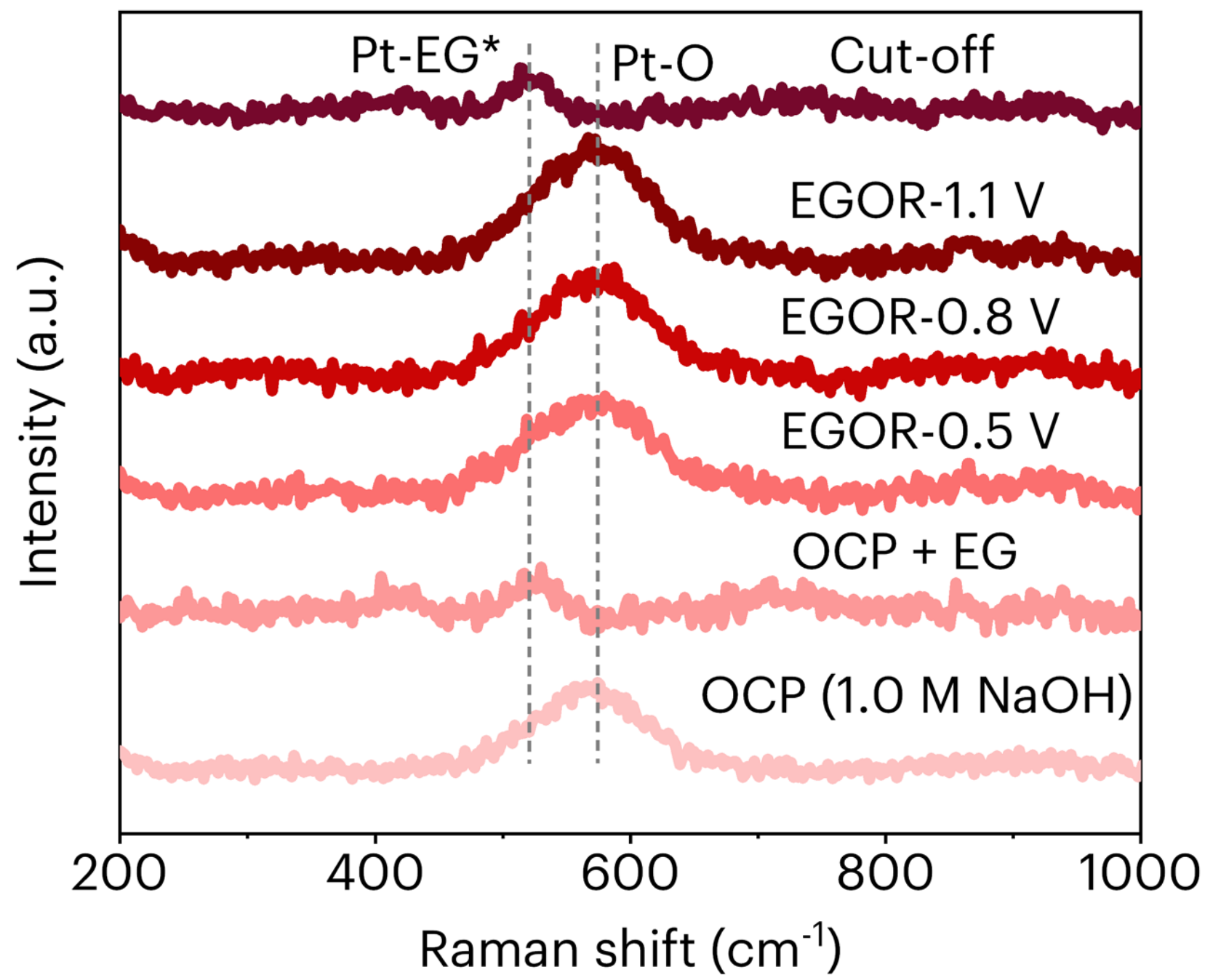

Fig. S60. Reaction intermediate analysis. *In-situ* Raman spectra of Pt on the glassy carbon electrode under different conditions.

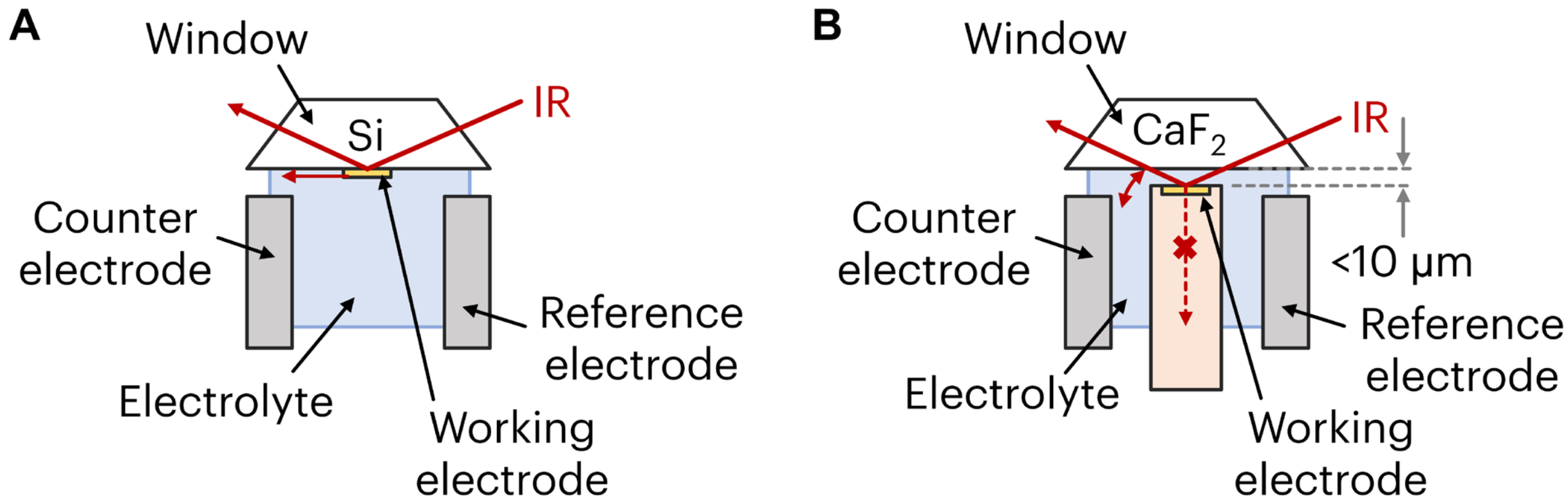

Fig. S61. Reaction intermediate analysis. Schematic diagrams of the mechanism for (**A**) *in-situ* FTIR spectra (ATR-SEIRAS model) and (**B**) *in-situ* FTIR spectra (IRRAS model).

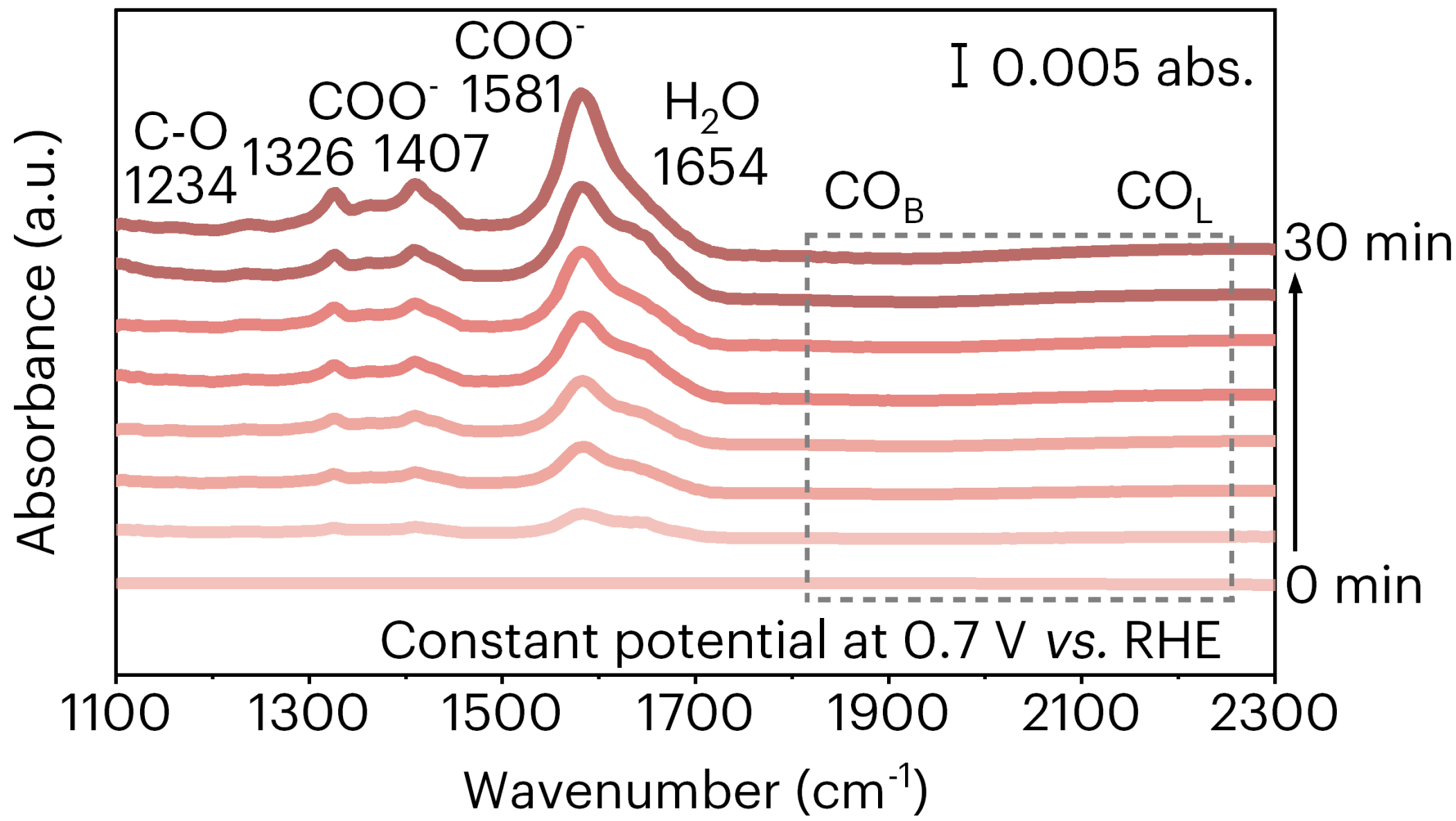

Fig. S62. Reaction intermediate analysis. *In-situ* FTIR spectra (IRRAS model) of Pt/NiCo(OH)$_x$ for EGOR through CP method in 1.0 M NaOH with 0.5 M EG.

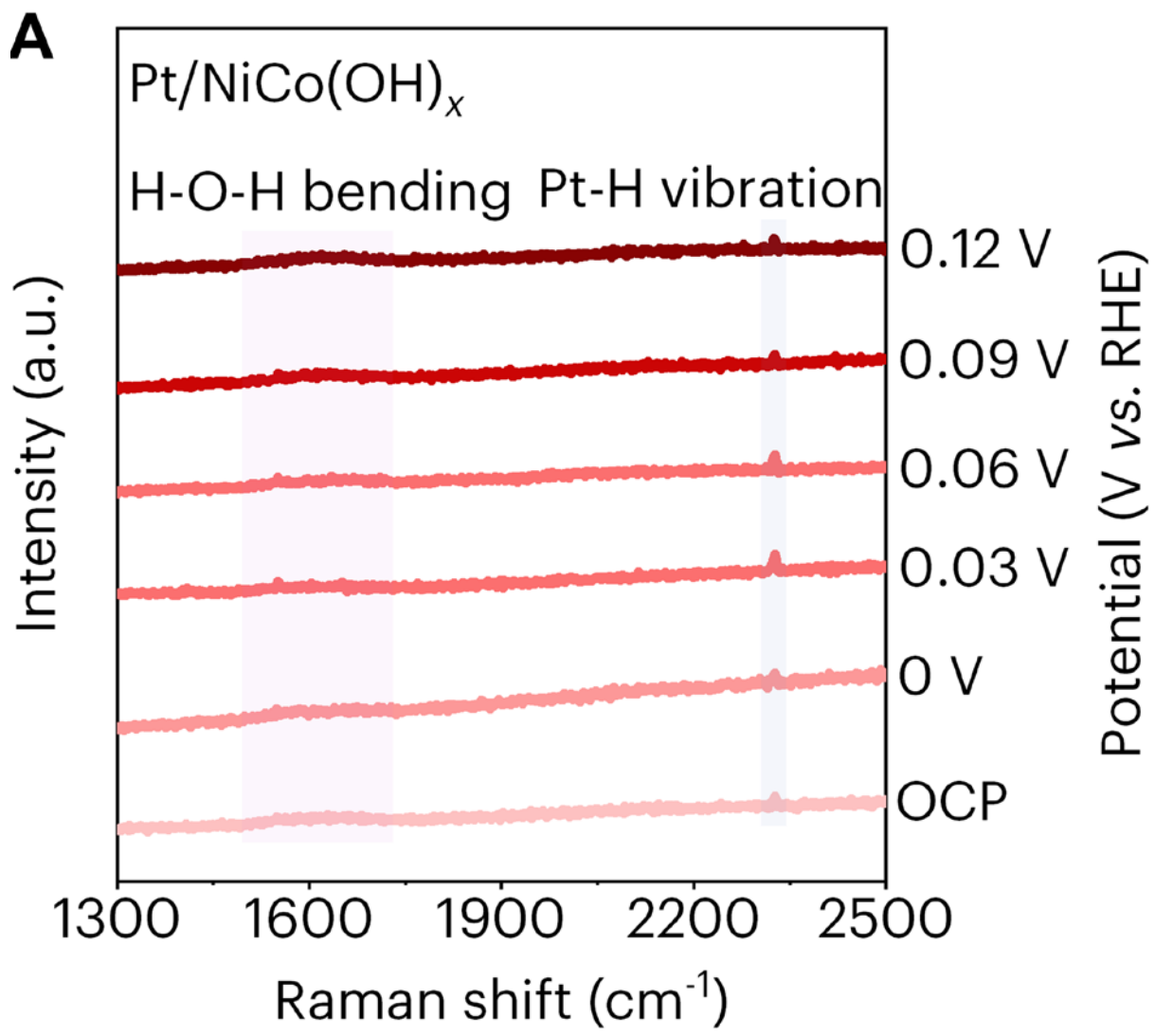

Fig. S63. Reaction intermediate analysis. *In-situ* Raman spectra of (**A**) Pt/NiCo(OH)$_x$ and (**B**) Pt NPs/NF for HER in 1.0 M NaOH.

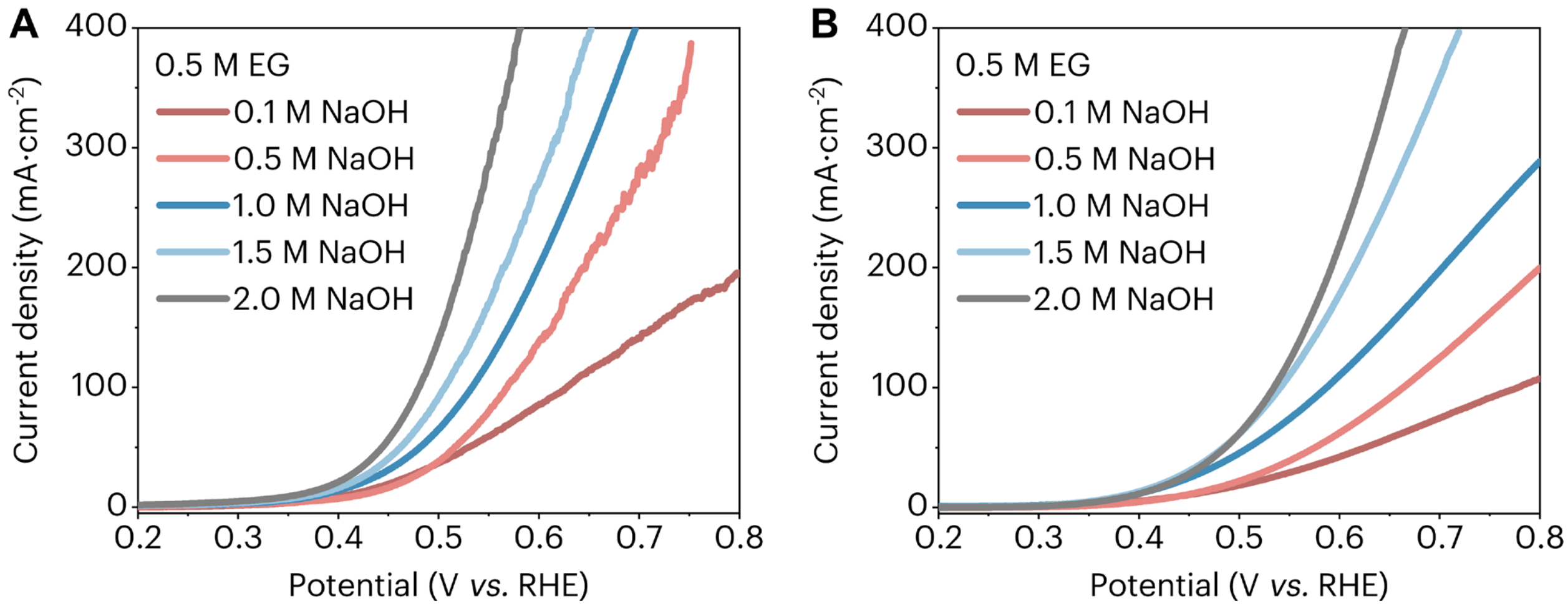

Fig. S64. Reaction order analysis. LSV curves for (**A**) Pt/NiCo(OH)$_x$ and (**B**) Pt NPs/NF in different concentrations of NaOH with 0.5 M EG.

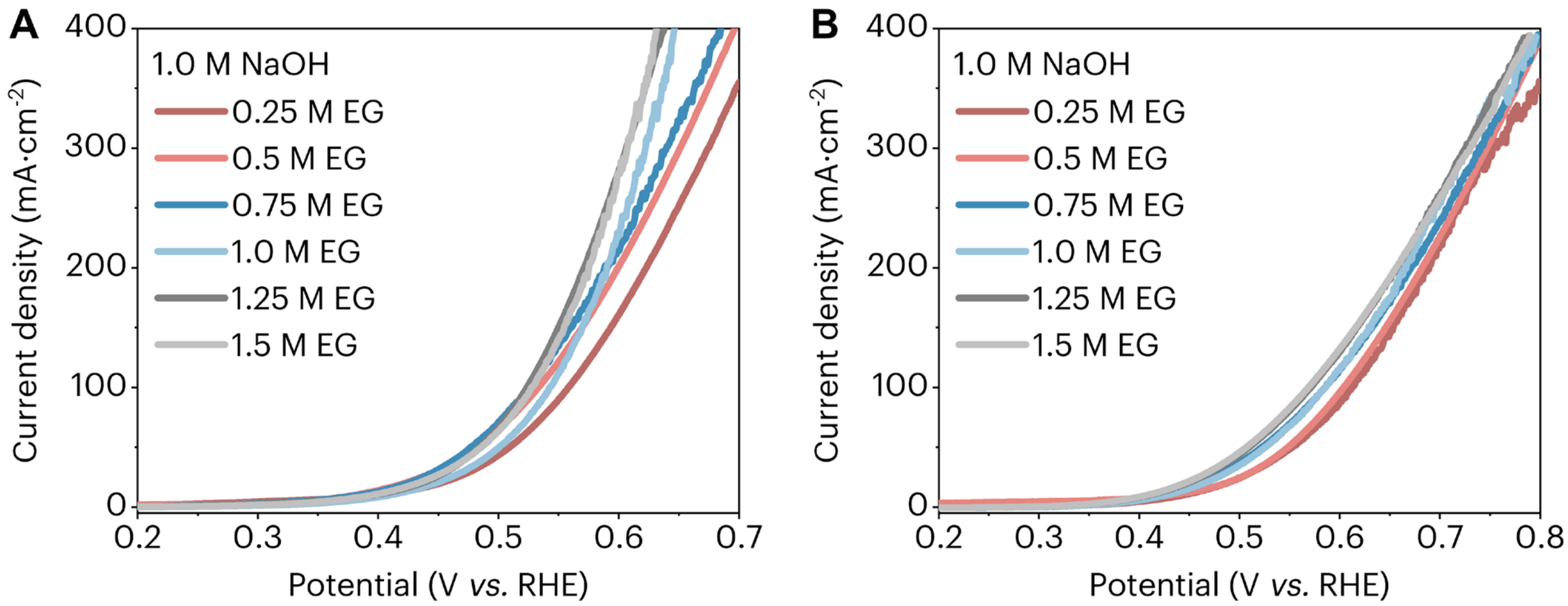

Fig. S65. Reaction order analysis. LSV curves for (**A**) Pt/NiCo(OH)$_x$ and (**B**) Pt NPs/NF in different concentrations of EG with 1.0 M NaOH.

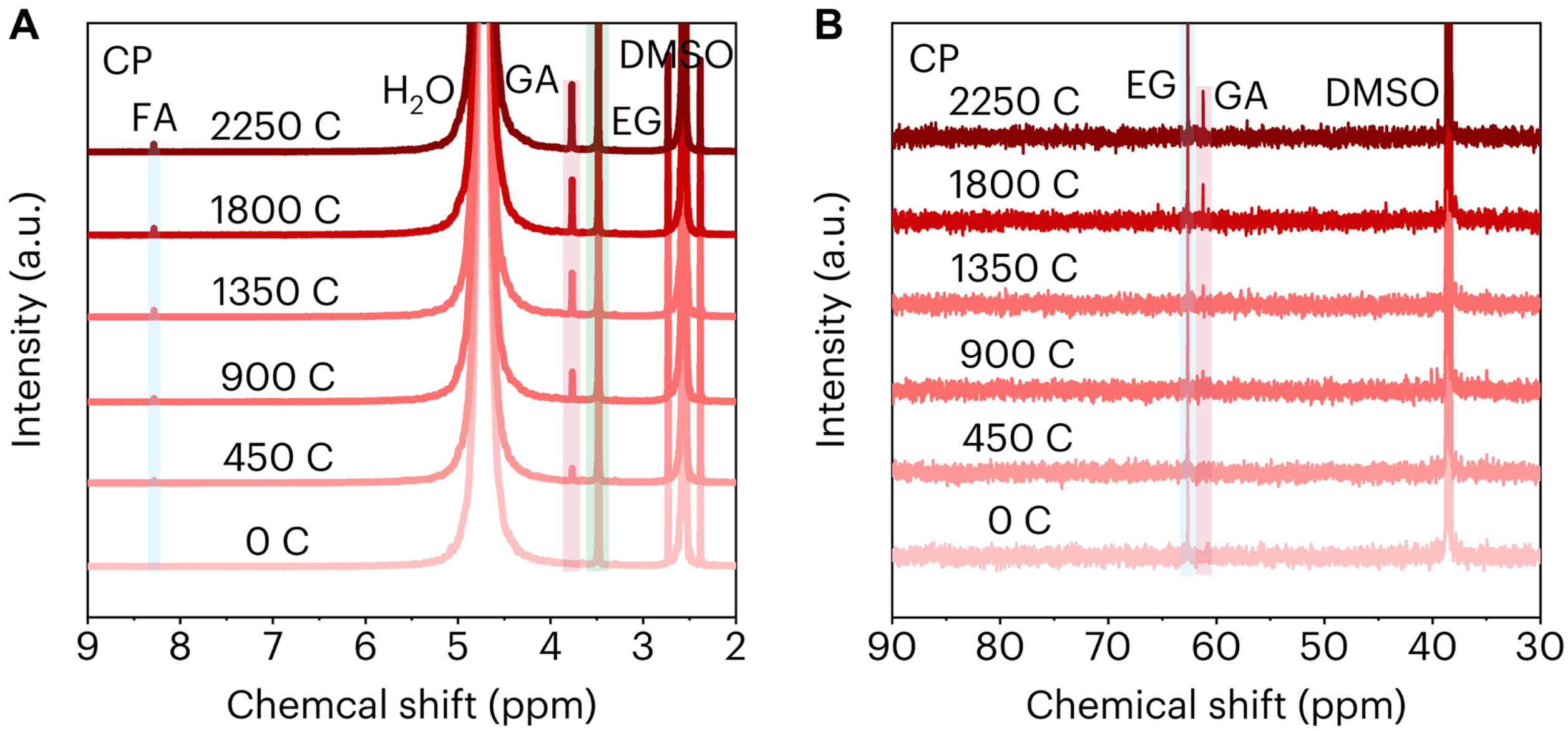

Fig. S66. Product analysis. (**A**) ^{1}H and (**B**) ^{13}C nuclear magnetic resonance (NMR) spectra of Pt/NiCo(OH)$_x$ for EGOR in 1.0 M NaOH with 0.1 M EG (0.1 L) after various electrolysis charges by CP method.

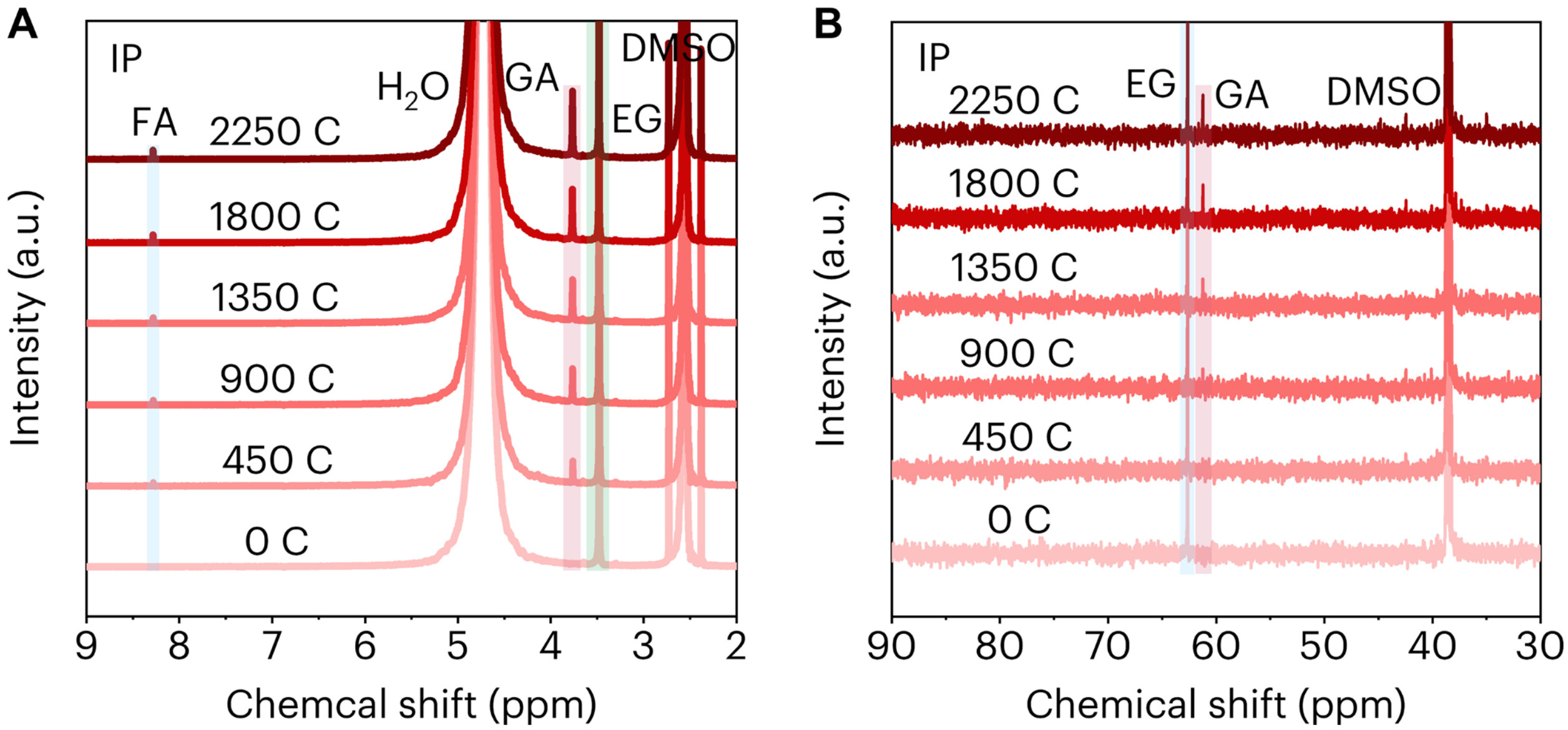

Fig. S67. Product analysis. (**A**) ^{1}H and (**B**) ^{13}C NMR spectra of Pt/NiCo(OH)$_x$ in 1.0 M NaOH with 0.1 M EG (0.1 L) for EGOR after various electrolysis charges by IP method.

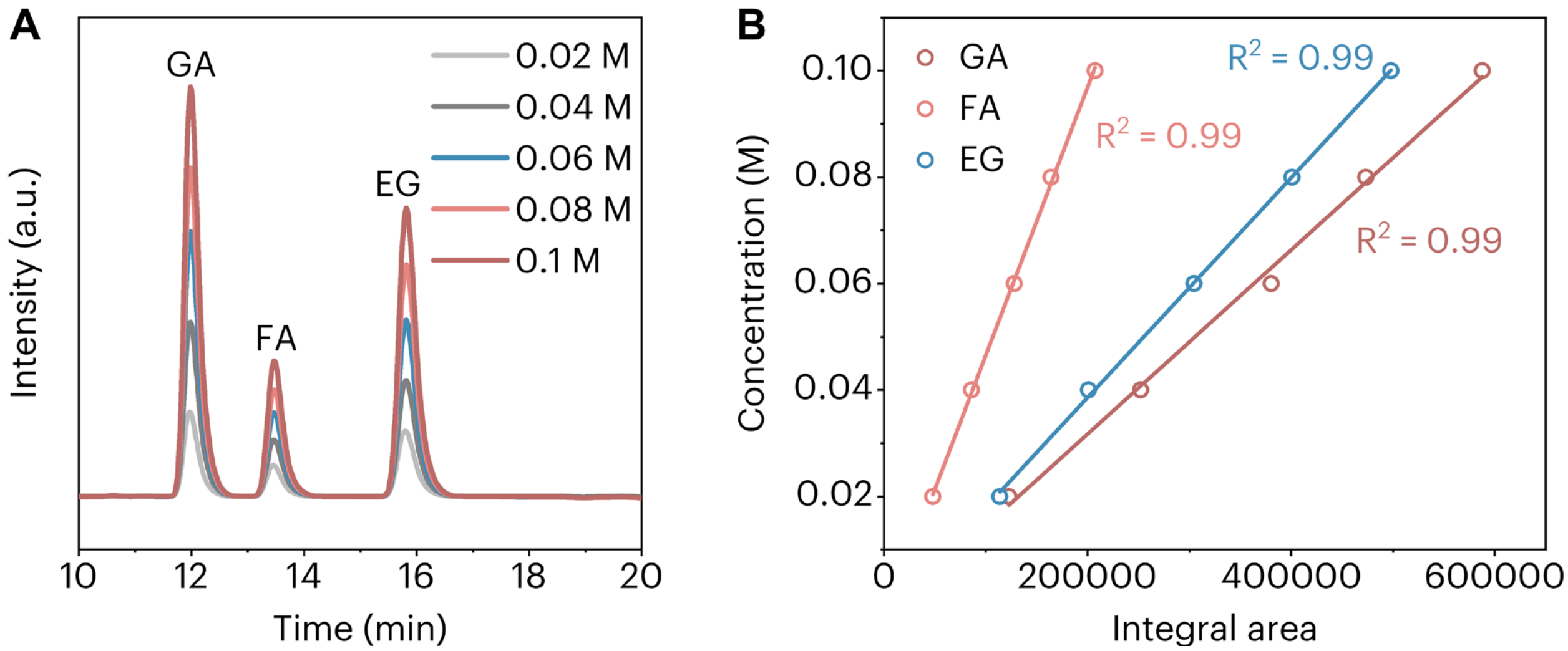

Fig. S68. Product analysis. (**A**) High-performance liquid chromatography (HPLC) acquired in different concentrations of GA, FA and EG. (**B**) Standard curves of GA, FA and EG under different concentrations.

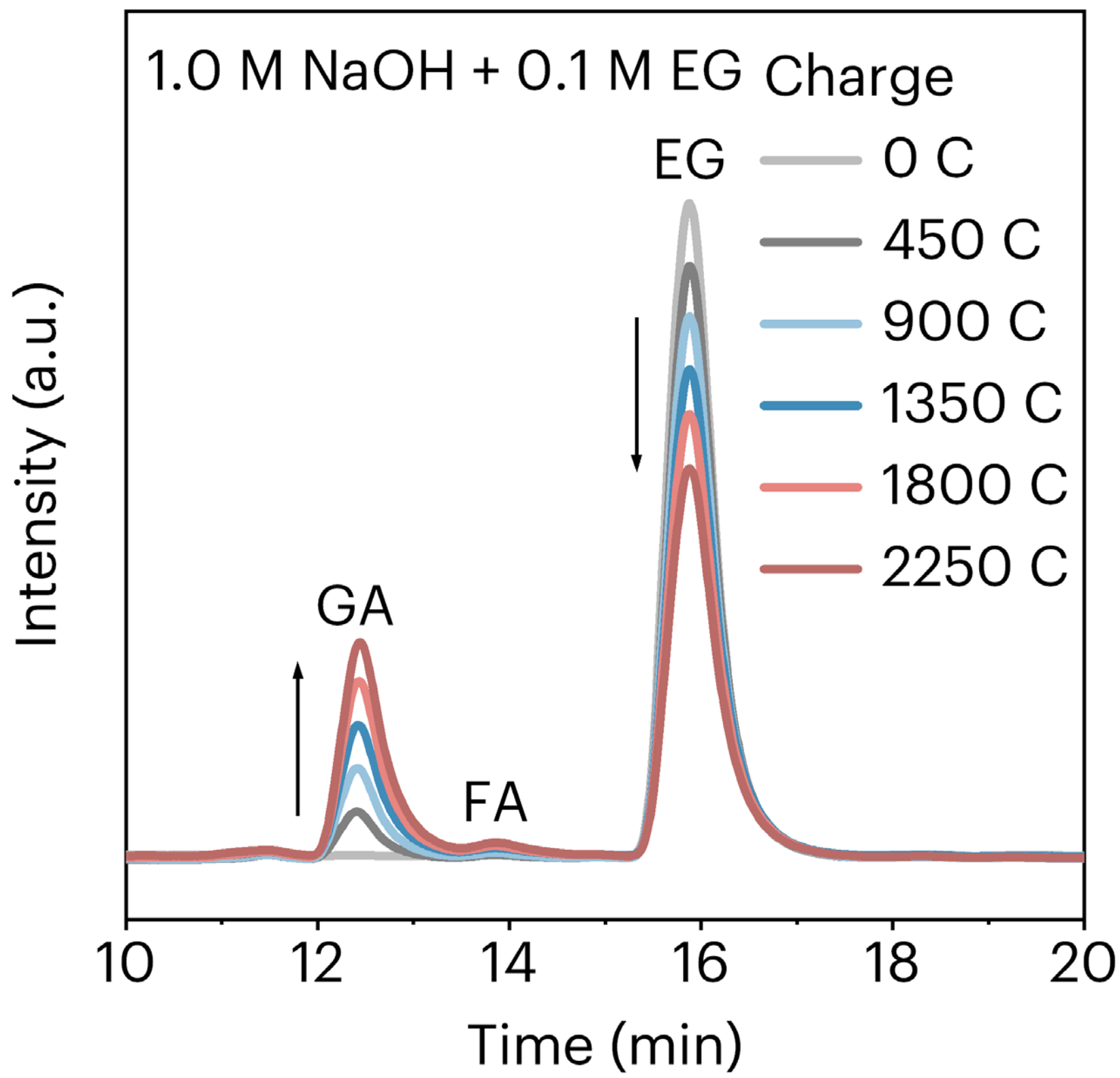

Fig. S69. Product analysis. HPLC chromatograms for Pt/NiCo(OH)$_x$ in 1.0 M NaOH with 0.1 M EG (0.1 L) after various electrolysis charges during EGOR.

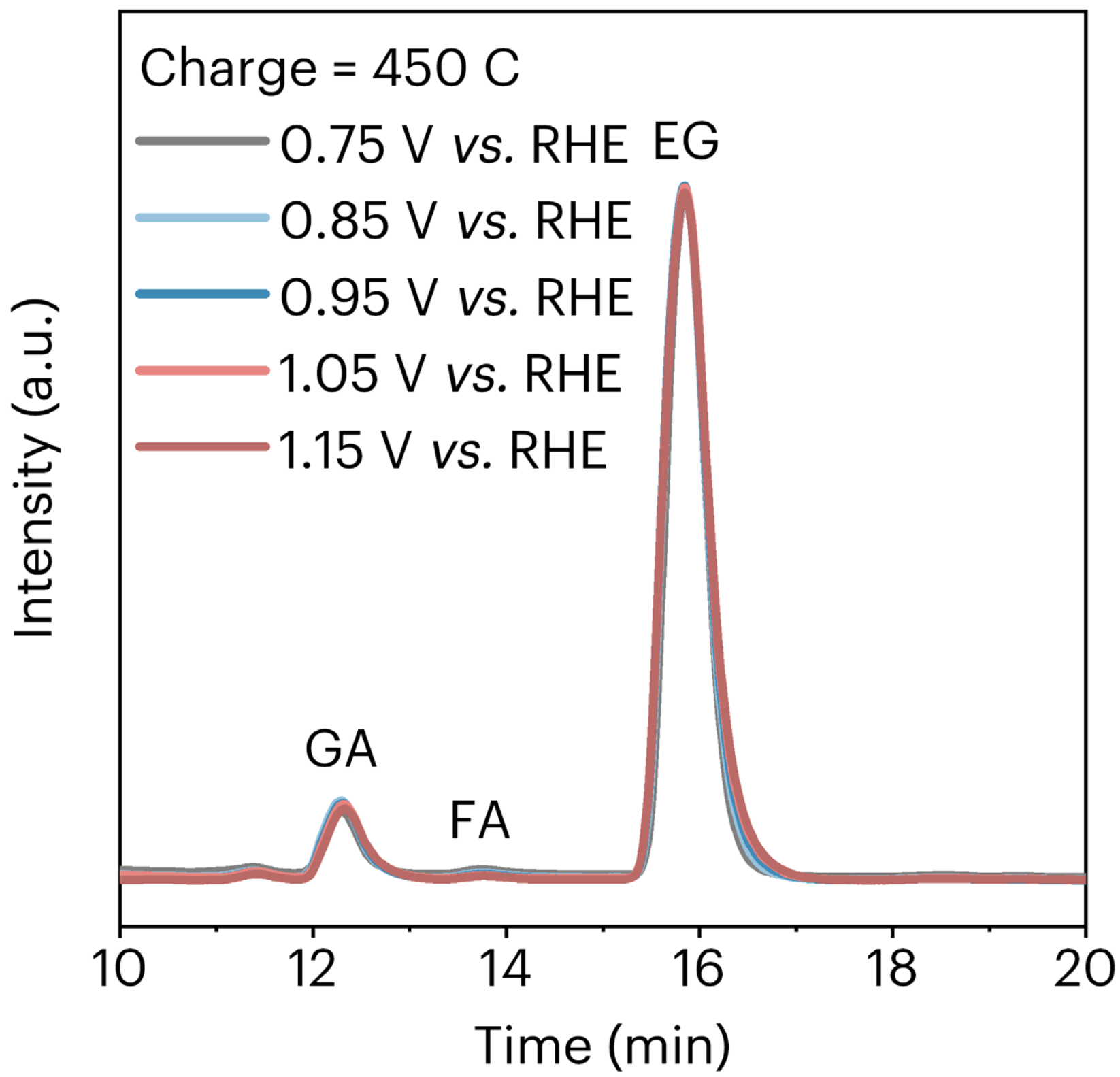

Fig. S70. Product analysis. HPLC chromatograms for Pt/NiCo(OH)$_x$ in 1.0 M NaOH with 0.1 M EG (0.1 L) after electrolysis charge of 450 C under different potentials.

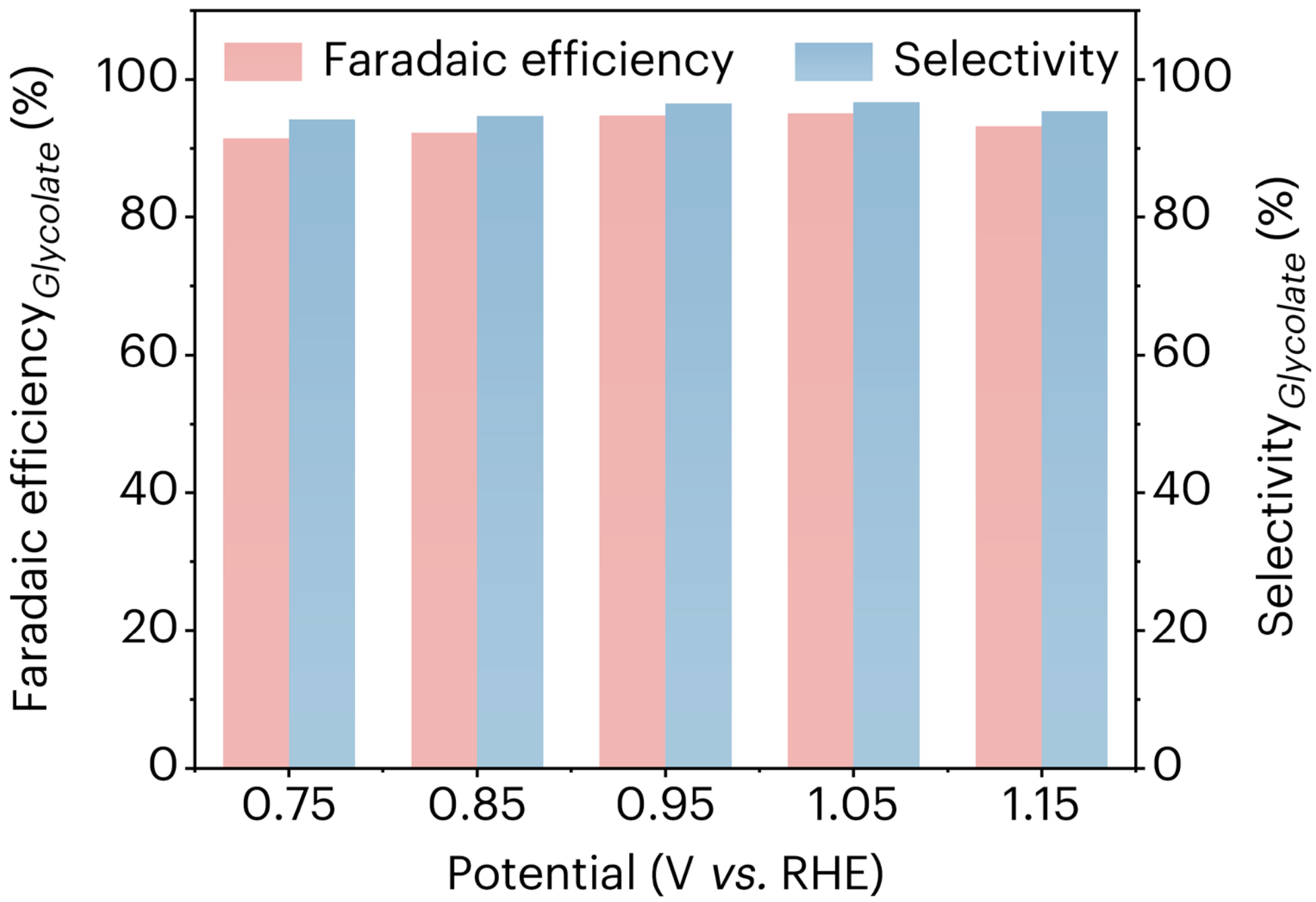

Fig. S71. Product analysis. Faradaic efficiency with selectivity of the Pt/NiCo(OH)$_x$ catalyst for glycolate under different potentials.

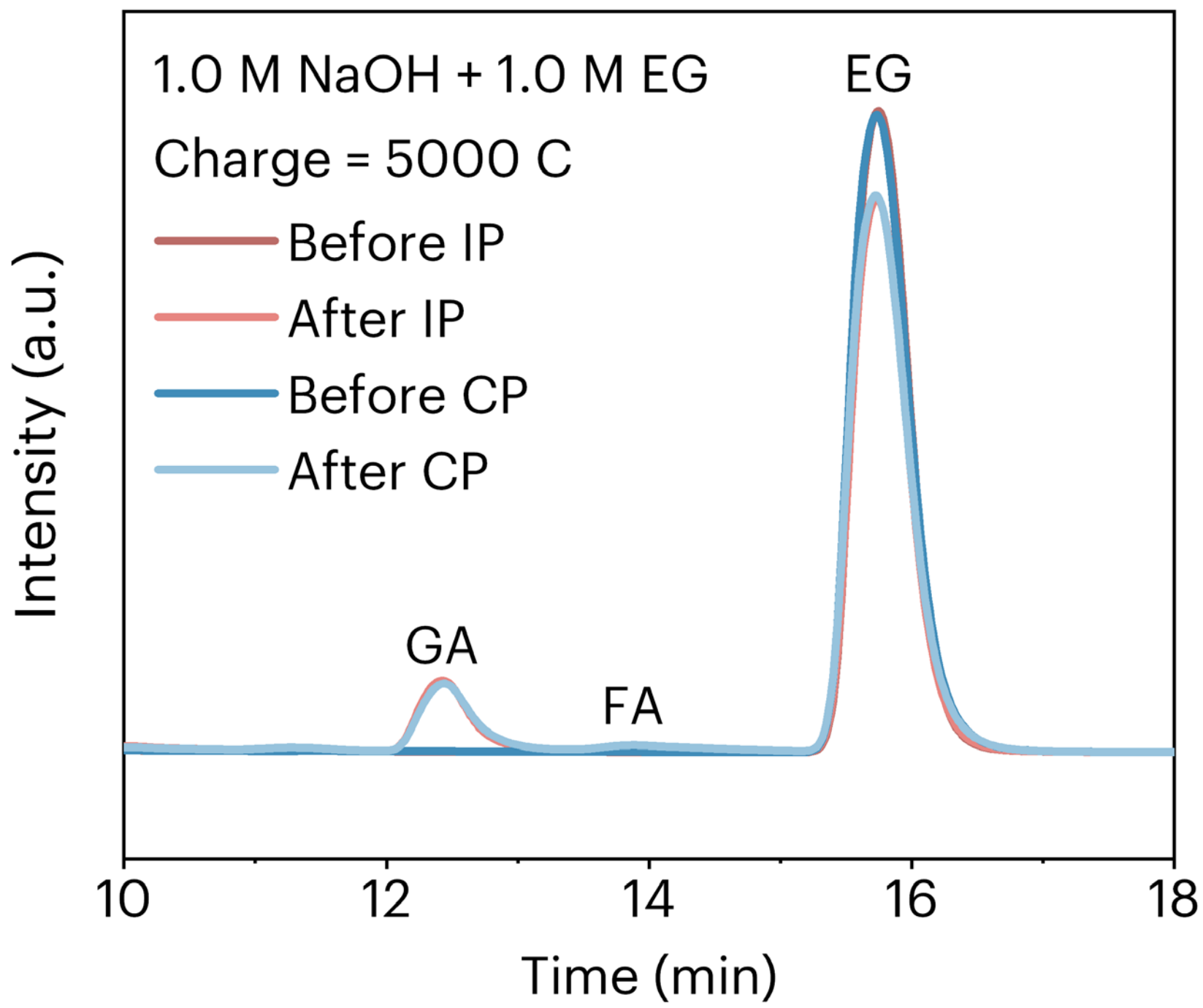

Fig. S72. Product analysis. HPLC chromatograms for Pt/NiCo(OH)$_x$ in 1.0 M NaOH with 1.0 M EG (0.1 L) after electrolysis charge of 5000 C under IP (1.05 V *vs.* RHE, 1200 s; 0.1 V *vs.* RHE, 2 s) and CP (1.05 V *vs.* RHE) modes.

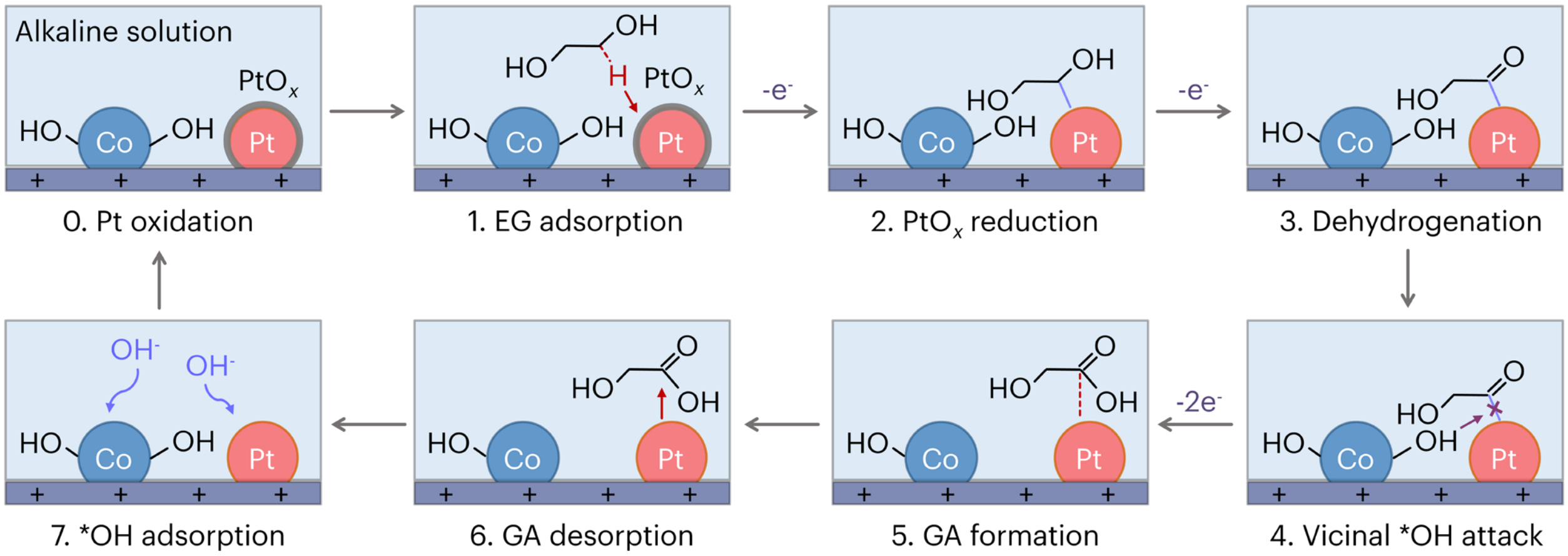

Fig. S73. EGOR mechanism diagram. Schematic representation of the EGOR mechanism for Pt/NiCo(OH)*x*.

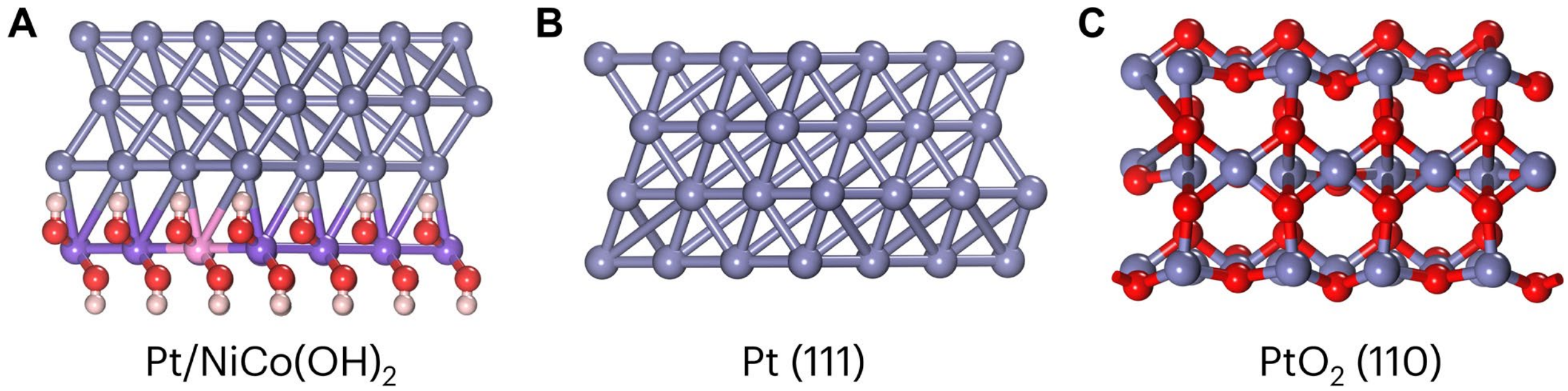

Fig. S74. Theoretical calculation. DFT-optimized configurations of (**A**) Pt/NiCo(OH)$_2$, (**B**) Pt (111) and (**C**) PtO$_2$ (110).

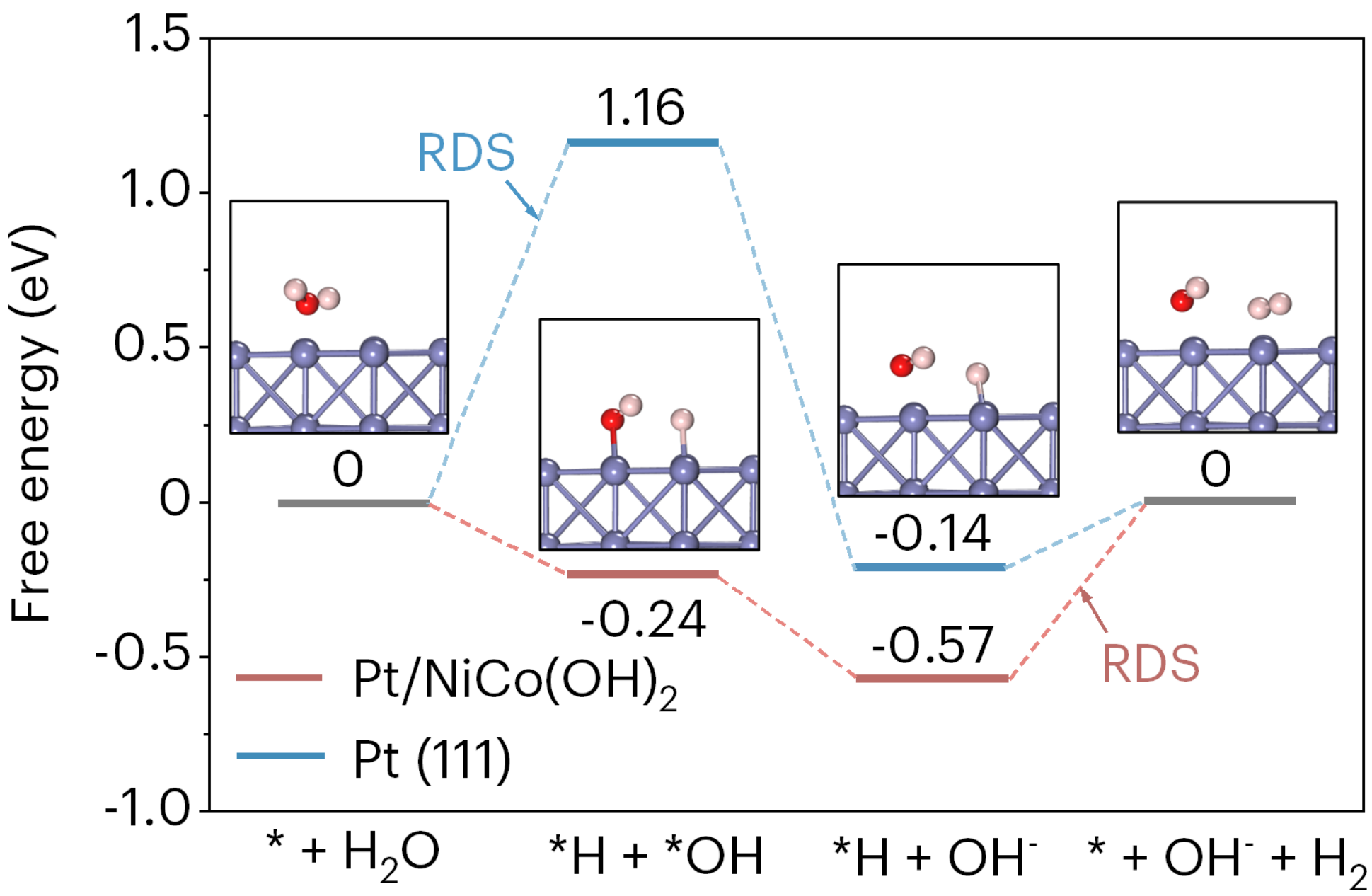

Fig. S75. Theoretical calculation. Gibbs free energy of HER stepwise pathway for Pt (111) and $Pt/NiCo(OH)_2$.

Note: The specific reaction steps of HER are described below:

1) $H_2O + cat \rightarrow H^*\text{-}cat + OH^*\text{-}cat$

2) $H^*\text{-}cat + OH^*\text{-}cat + e^- \rightarrow H^*\text{-}cat + OH^-$

3) $H^*\text{-}cat + H_2O + e^- \rightarrow cat + OH^- + H_2$

Four stages of the free energy calculations for HER include initial state, activated water adsorption, H* intermediate formation, H_2 desorption. At the applied potential of $U_0 = 0$ V *ys.* RHE, the free energies for HER are calculated as following:

1) $G_0 = G_{cat} + G_{H_2O}$

2) $G_1 = G_{H*cat} + G_{OH*cat}$

3) $G_2 = G_{H*\text{-}cat} + G_{OH-}$

4) $G_3 = G_{cat} + G_{OH-} + 1/2G_{H_2} = G_0$

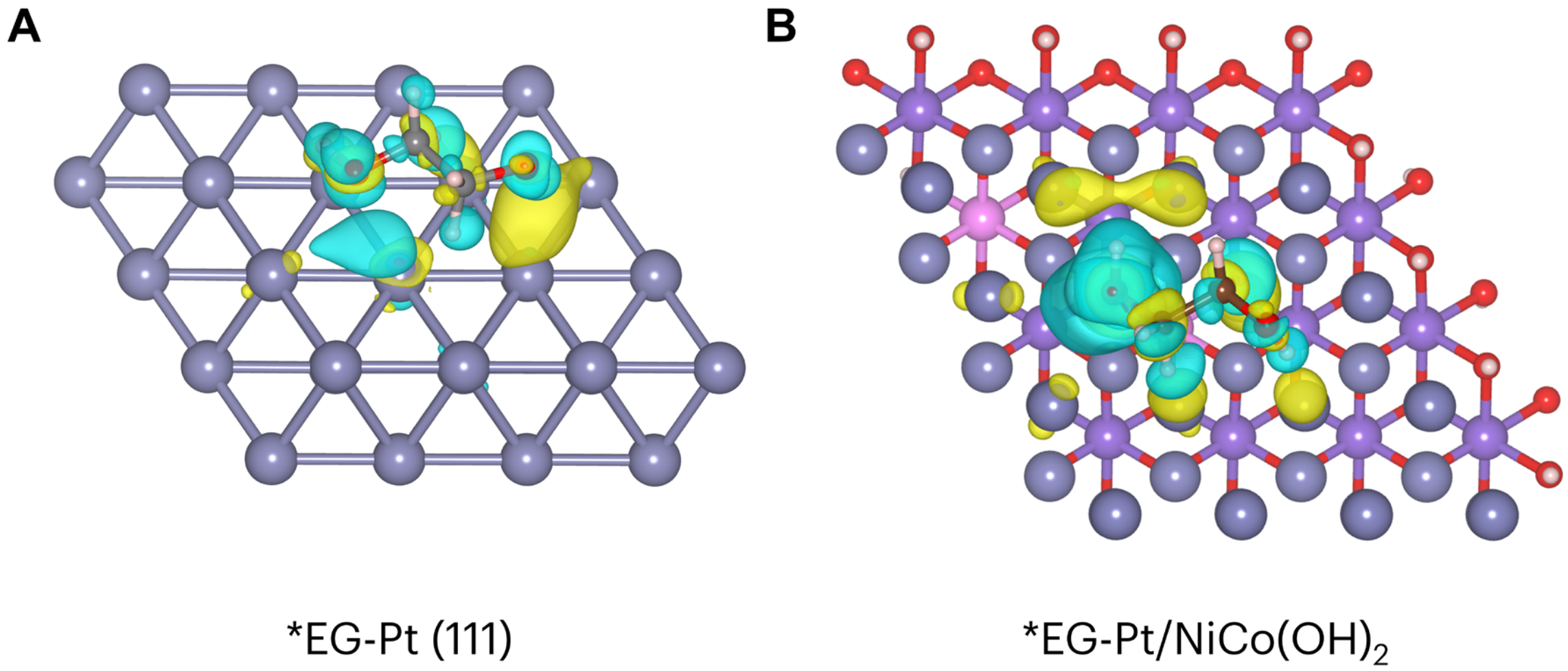

Fig. S76. Theoretical calculation. Charge density difference analysis of (**A**) EG-adsorbed Pt (111) and (**B**) EG-adsorbed Pt/$NiCo(OH)_2$. Blue and yellow regions represent electron accumulation and depletion.

Note: values of average Bader charges for Pt sites are -0.02 $e \cdot atom^{-1}$ (EG-adsorbed Pt (111)) and -0.07 $e \cdot atom^{-1}$ (EG-adsorbed Pt/$NiCo(OH)_2$).

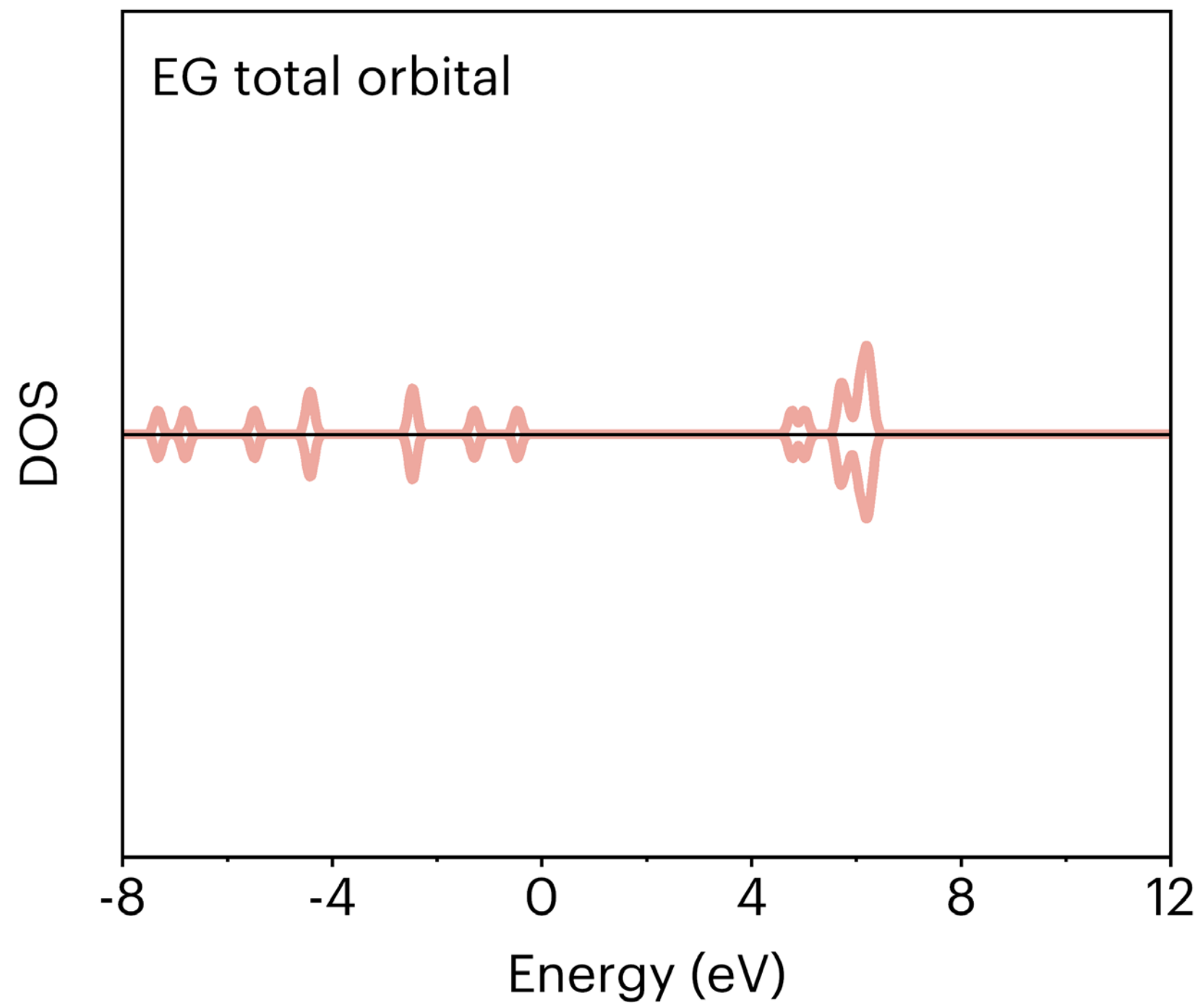

Fig. S77. Theoretical calculation. Density of states (DOS) analysis of EG. Isosurface value is set to be 0.005 eV·Å^{-3}.

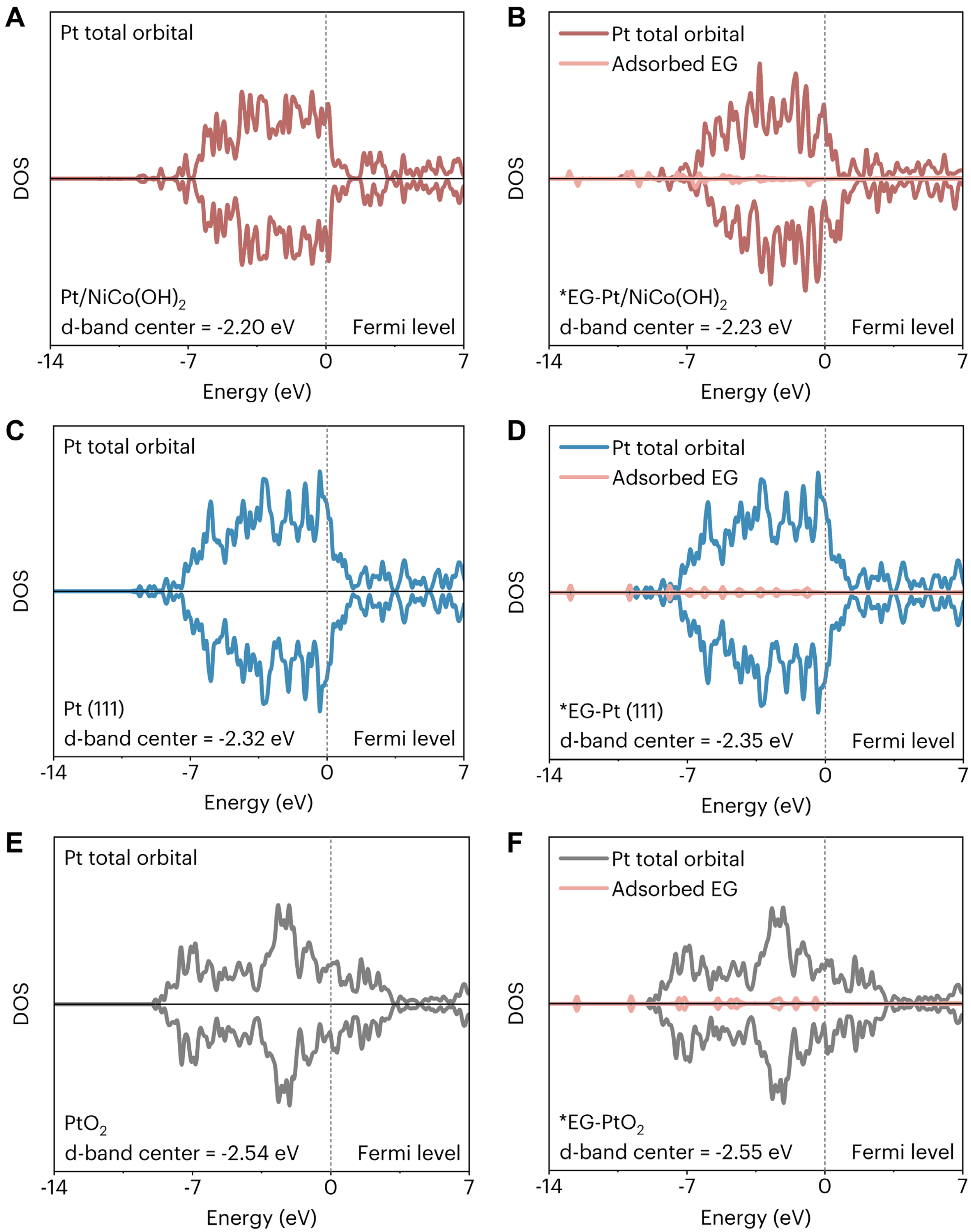

Fig. S78. Theoretical calculation. DOS analysis of Pt total d-orbitals for (**A**) Pt/NiCo$(OH)_2$, (**B**) EG-adsorbed Pt/NiCo$(OH)_2$, (**C**) Pt (111), (**D**) EG-adsorbed Pt (111), (**E**) PtO_2 (110) and (**F**) EG-adsorbed PtO_2 (110). Isosurface value is set to be 0.005 eV·Å^{-3}.

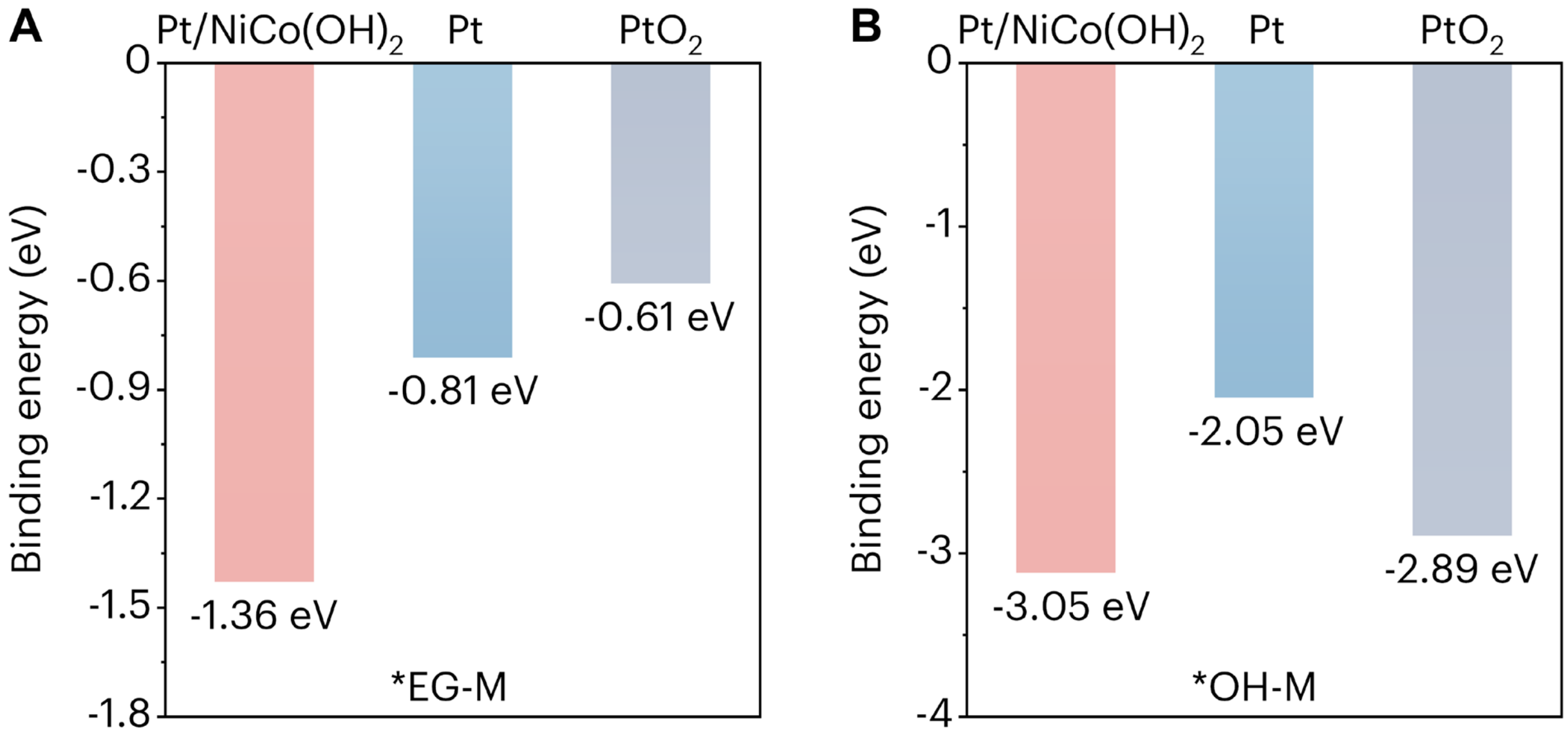

Fig. S79. Theoretical calculation. Binding energies of (**A**) EG and (**B**) hydroxyl for $Pt/NiCo(OH)_2$, Pt (111) and PtO_2 (110).

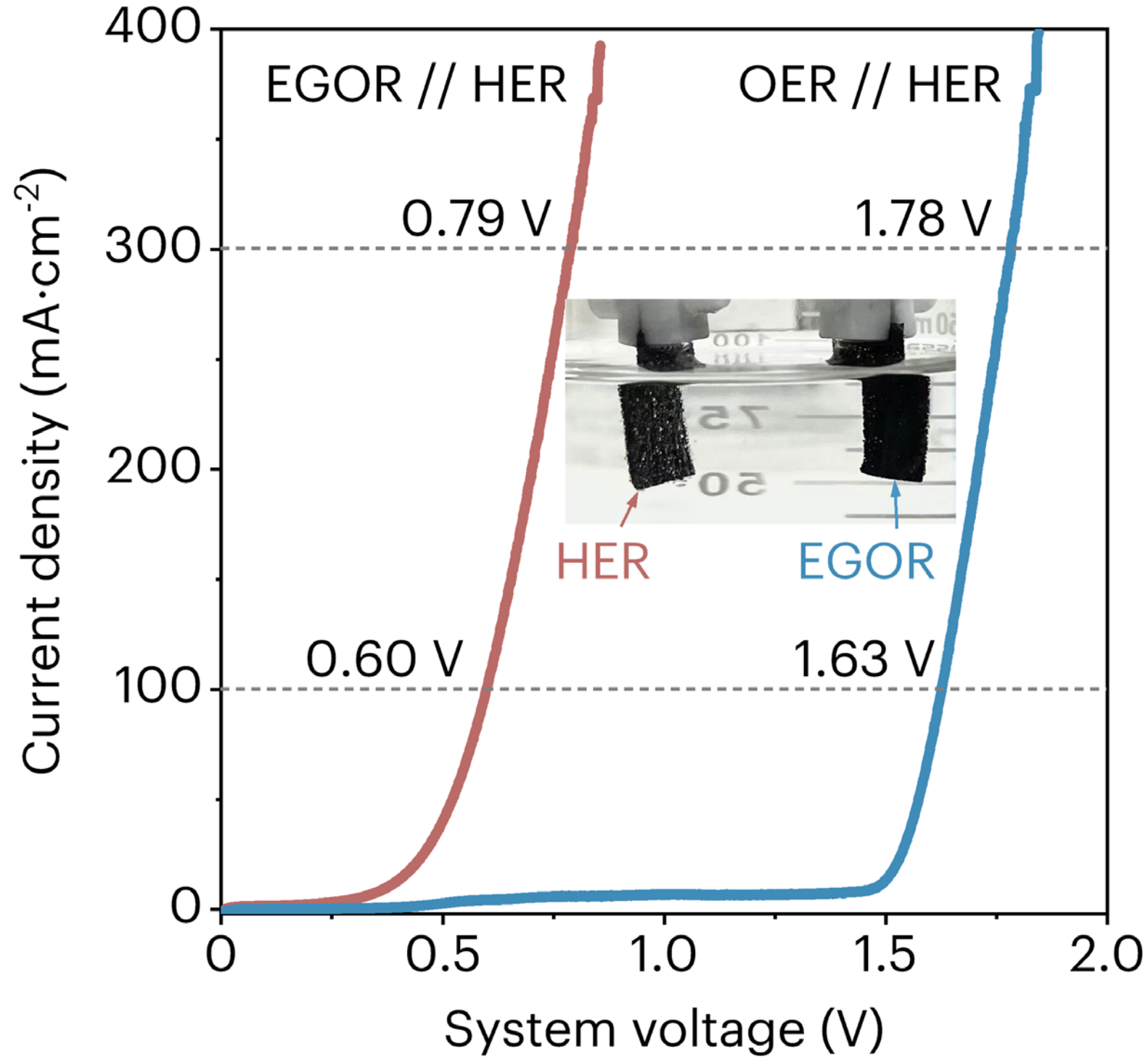

Fig. S80. Bifunctional electrochemical activity. LSV curves for EGOR // HER and OER // HER using the Pt/NiCo(OH)$_x$ catalyst. *Inset* shows the digital image of the two-electrode system (with 85% *iR* correction).

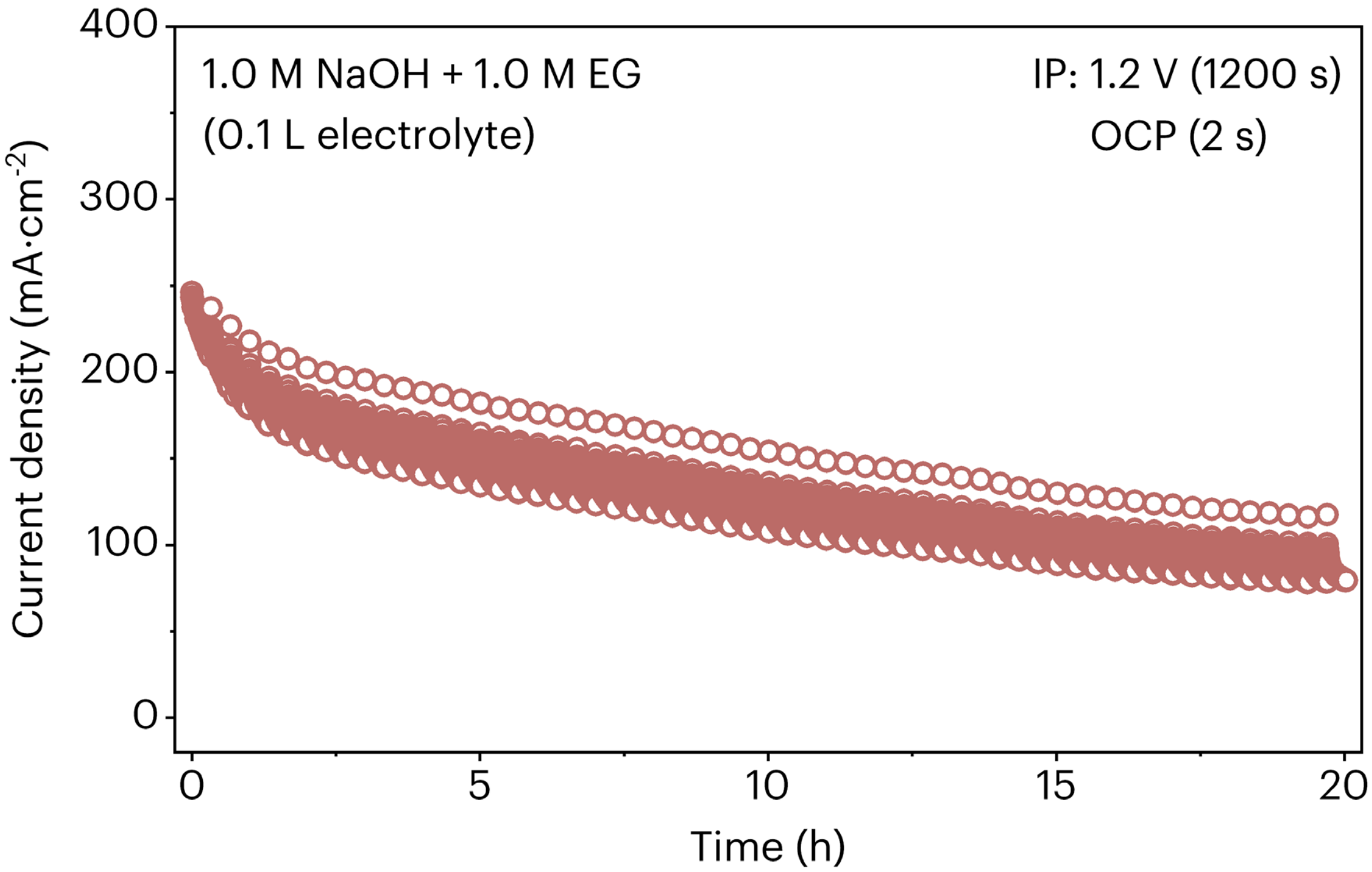

Fig. S81. Bifunctional electrochemical activity. Stability test of the Pt/NiCo(OH)$_x$ assembled two-electrode system by IP strategy.

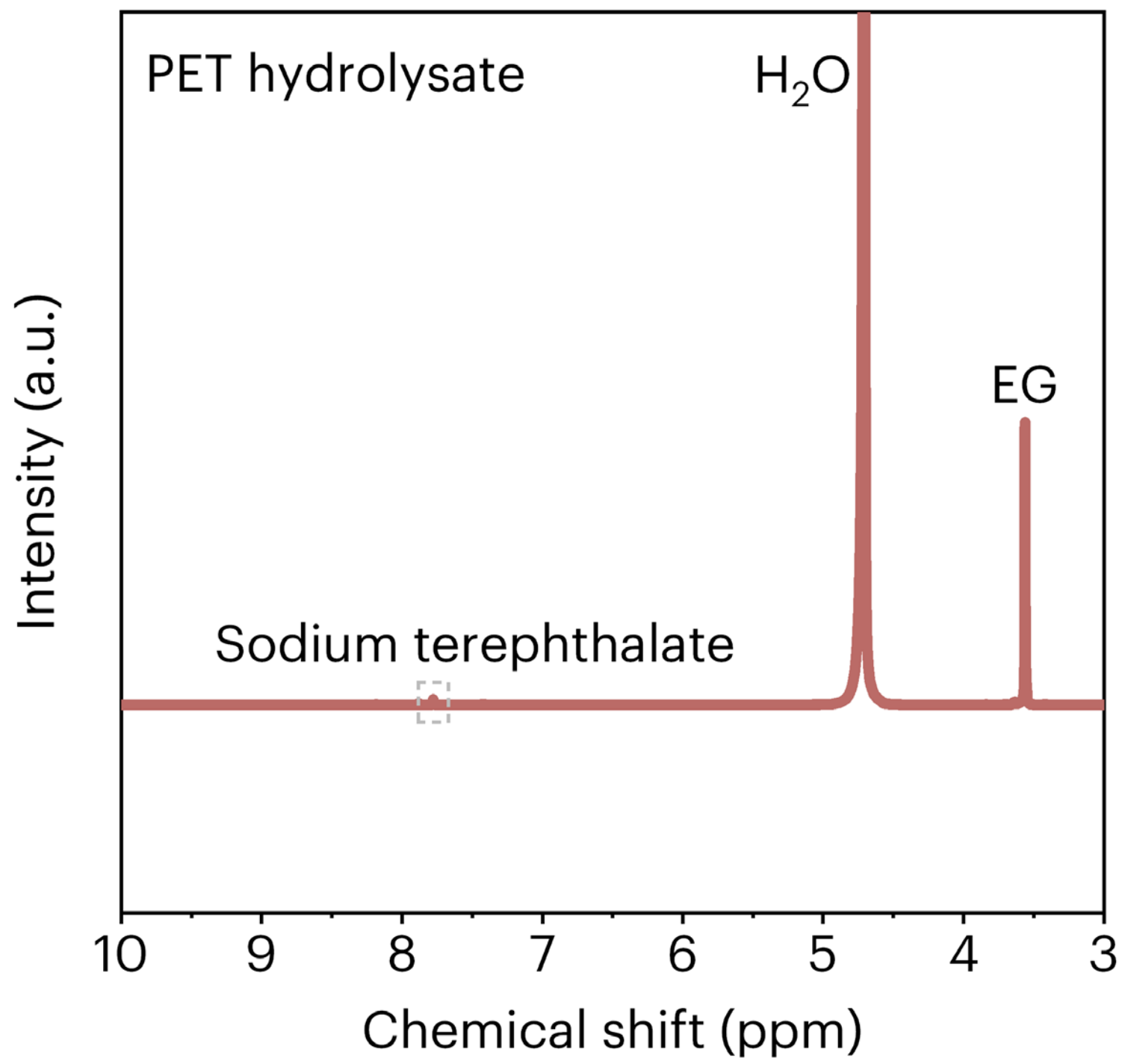

Fig. S82. Product analysis. ^{1}H NMR spectra of the PET hydrolysate.

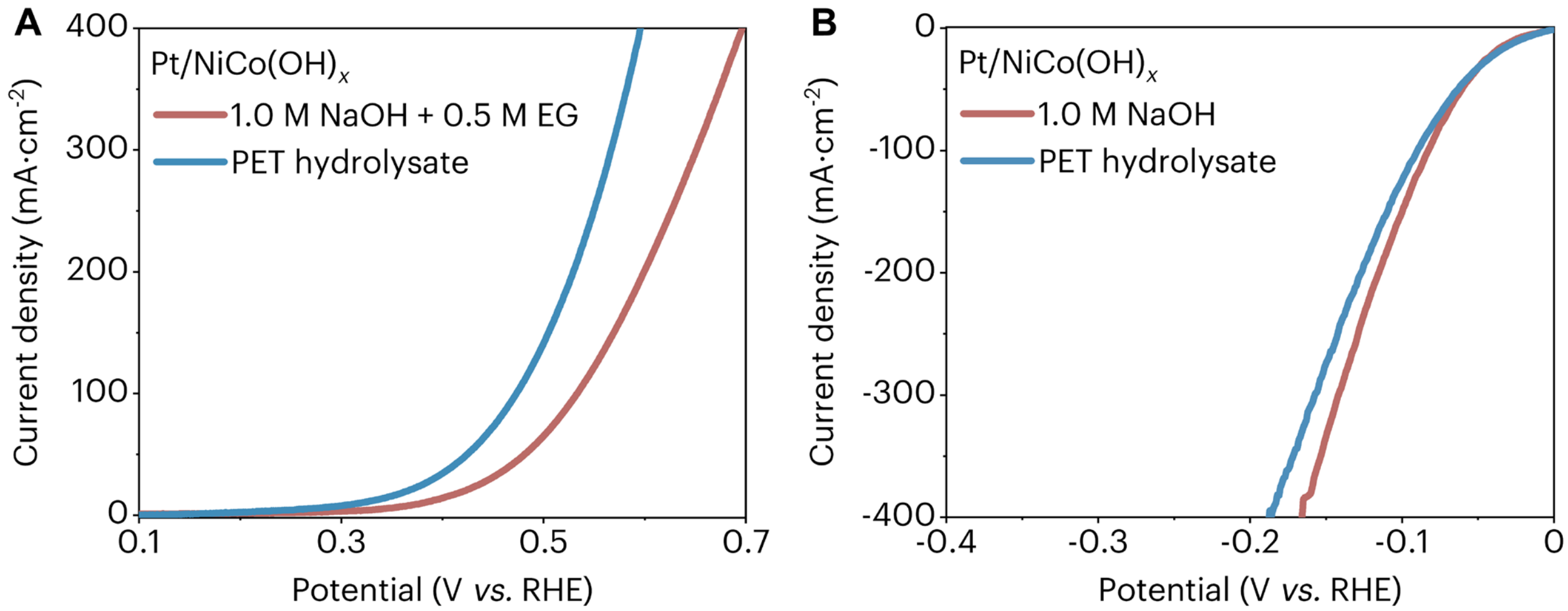

Fig. S83. Bifunctional electrochemical activity. (**A**) EGOR and (**B**) HER LSV curves of Pt/NiCo(OH)*x* in 1.0 M NaOH with 0.5 M EG and PET hydrolysate.

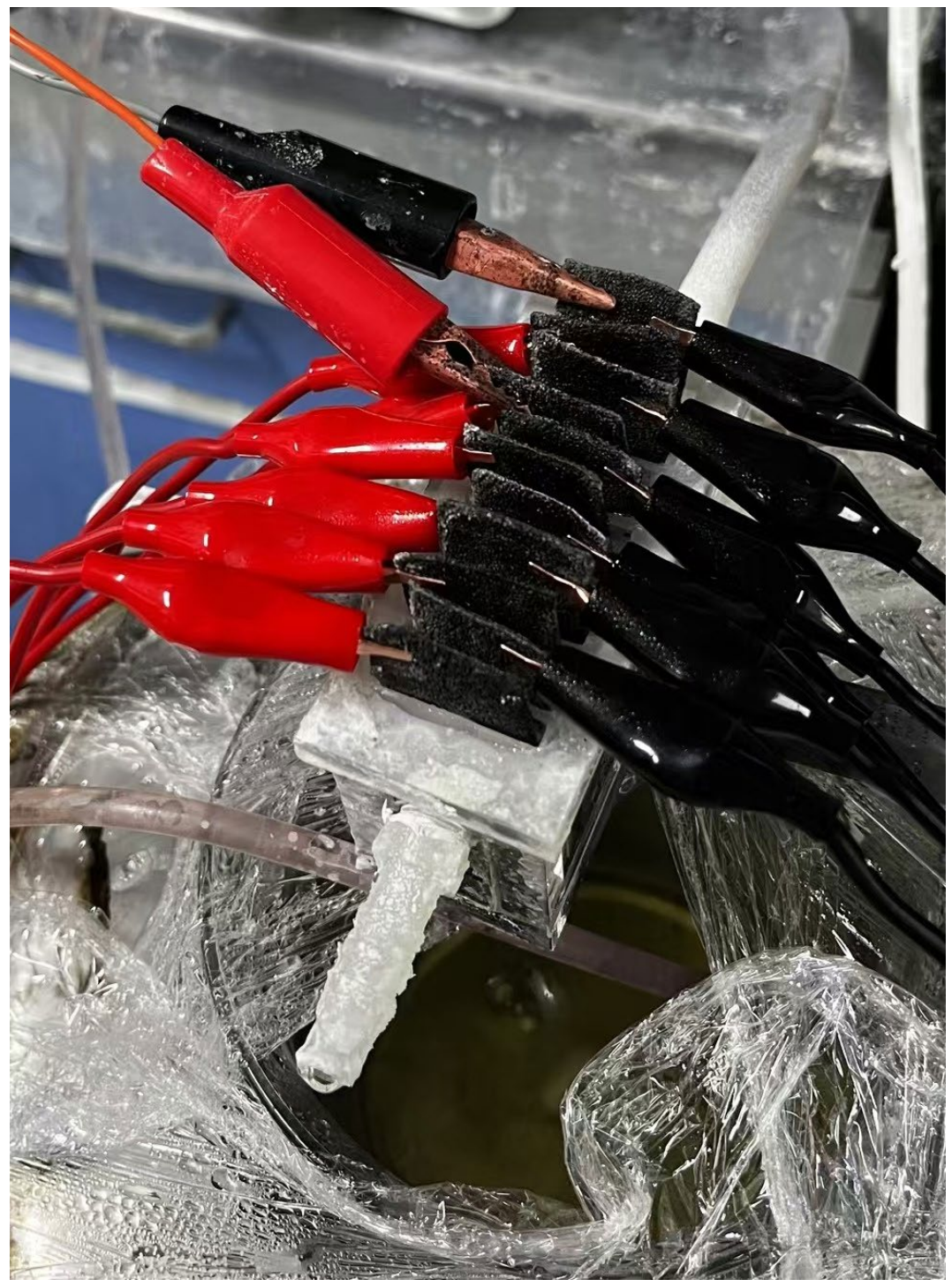

Fig. S84. Scale-up reaction device diagram. Digital image of the six-level slot type flow electrolyzer.

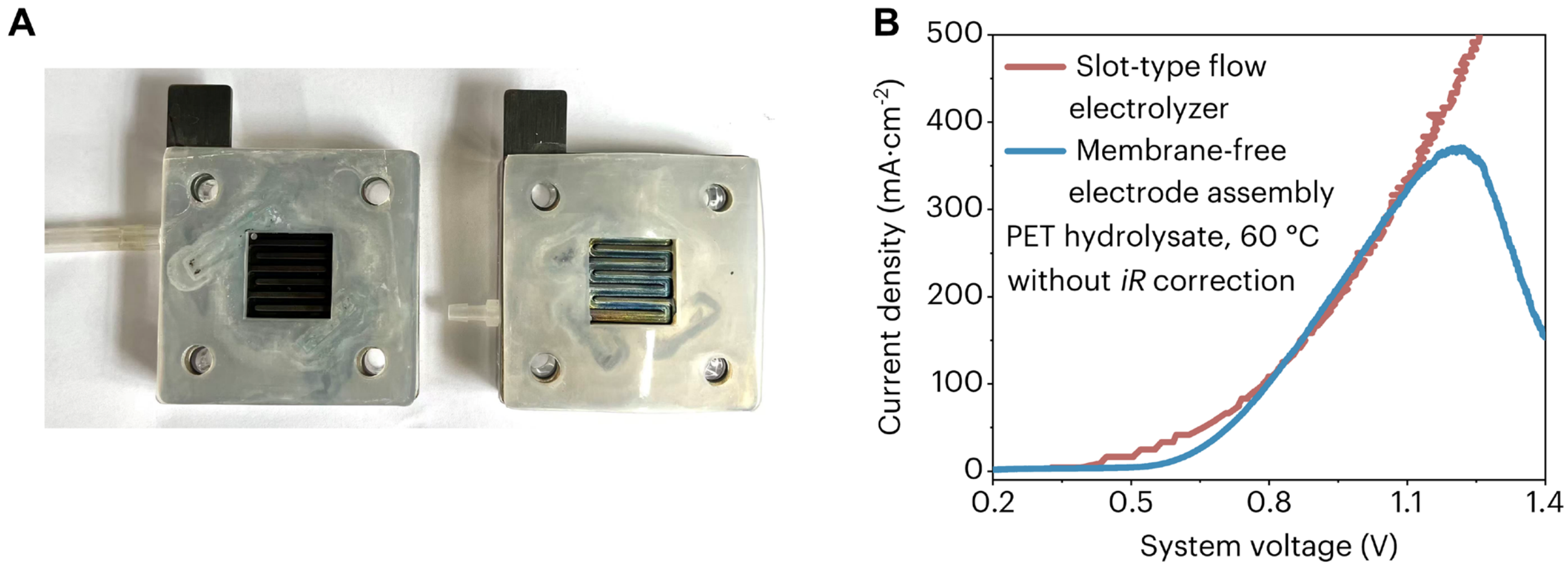

Fig. S85. Scale-up reaction device diagram. (**A**) Digital image of the membrane-free electrode assembly electrolyzer. (**B**) LSV curves for EGOR // HER using the Pt/NiCo(OH)$_x$ catalyst in the different devices.

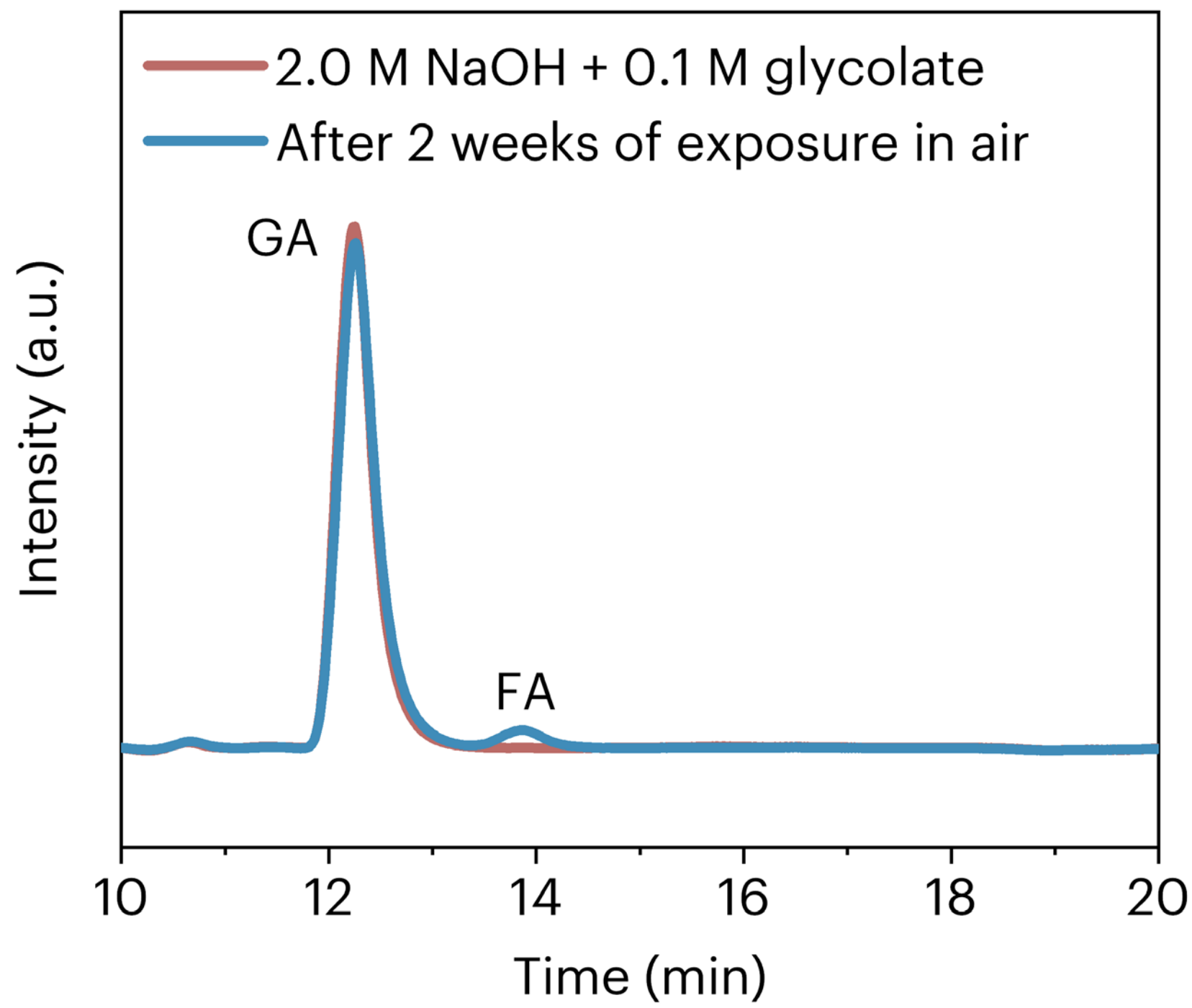

Fig. S86. Product analysis. HPLC diagrams of 2.0 M NaOH + 0.1 M glycolate before and after 2 weeks exposure in air.

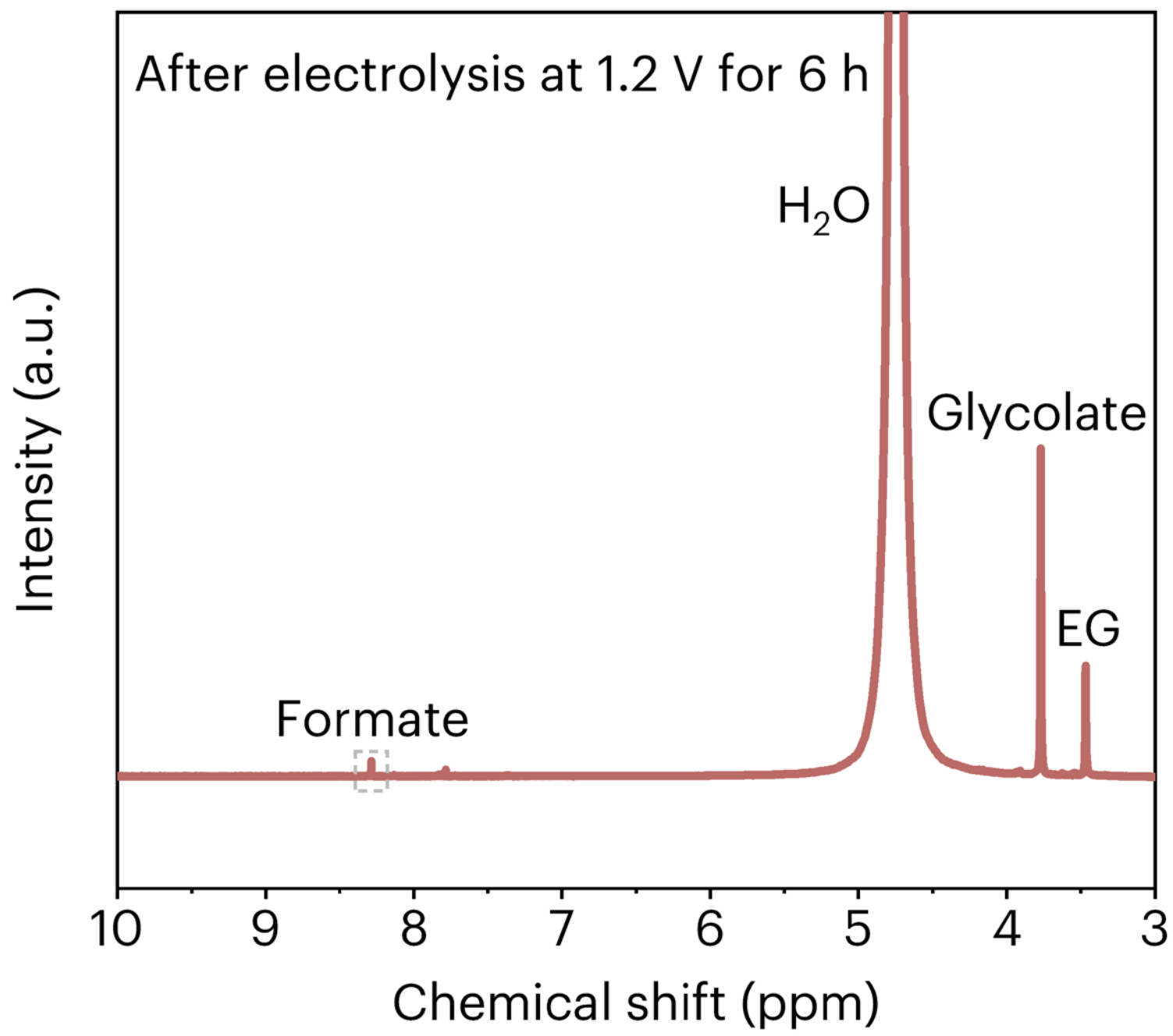

Fig. S87. Product analysis. ^{1}H NMR spectra of the PET-hydrolysate after electrolysis at 1.2 V for 6 h.

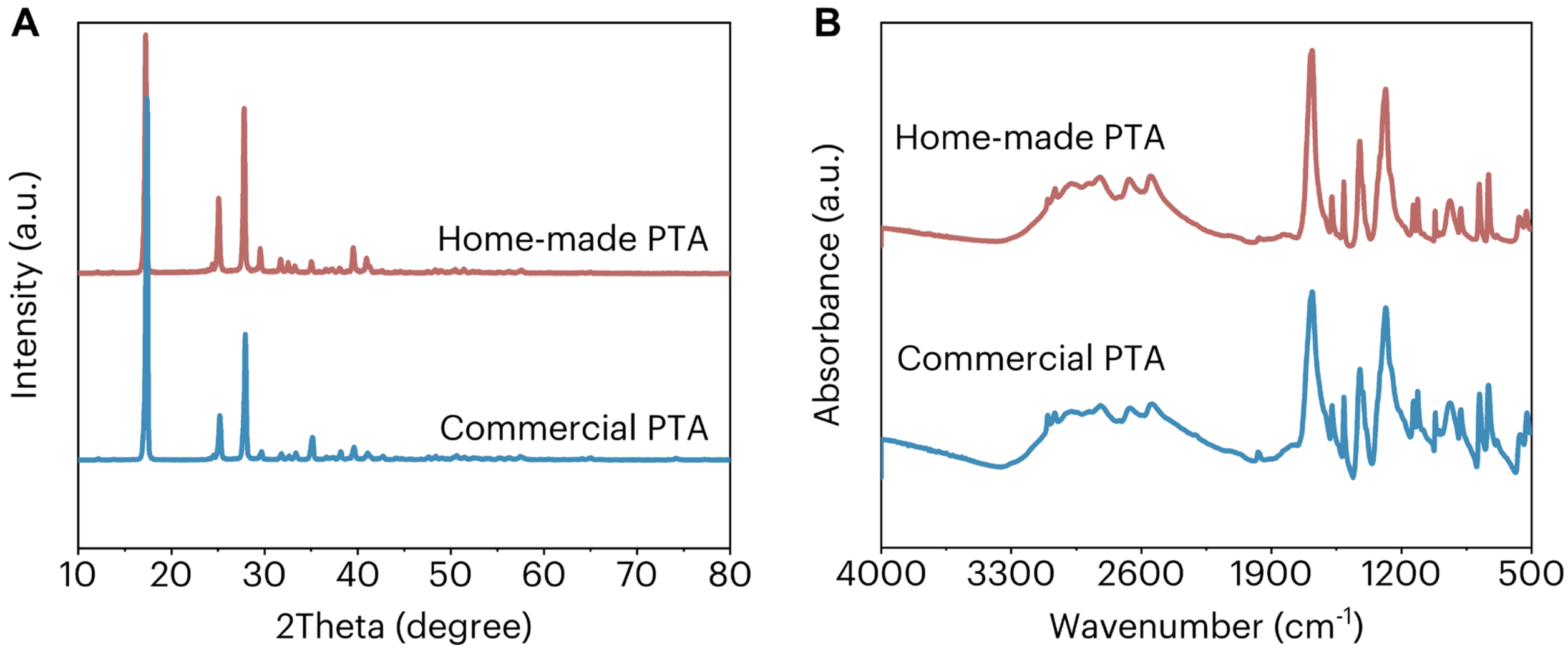

Fig. S88. Product analysis. (**A**) XRD patterns and (**B**) FTIR spectra of home-made PTA and commercial PTA.

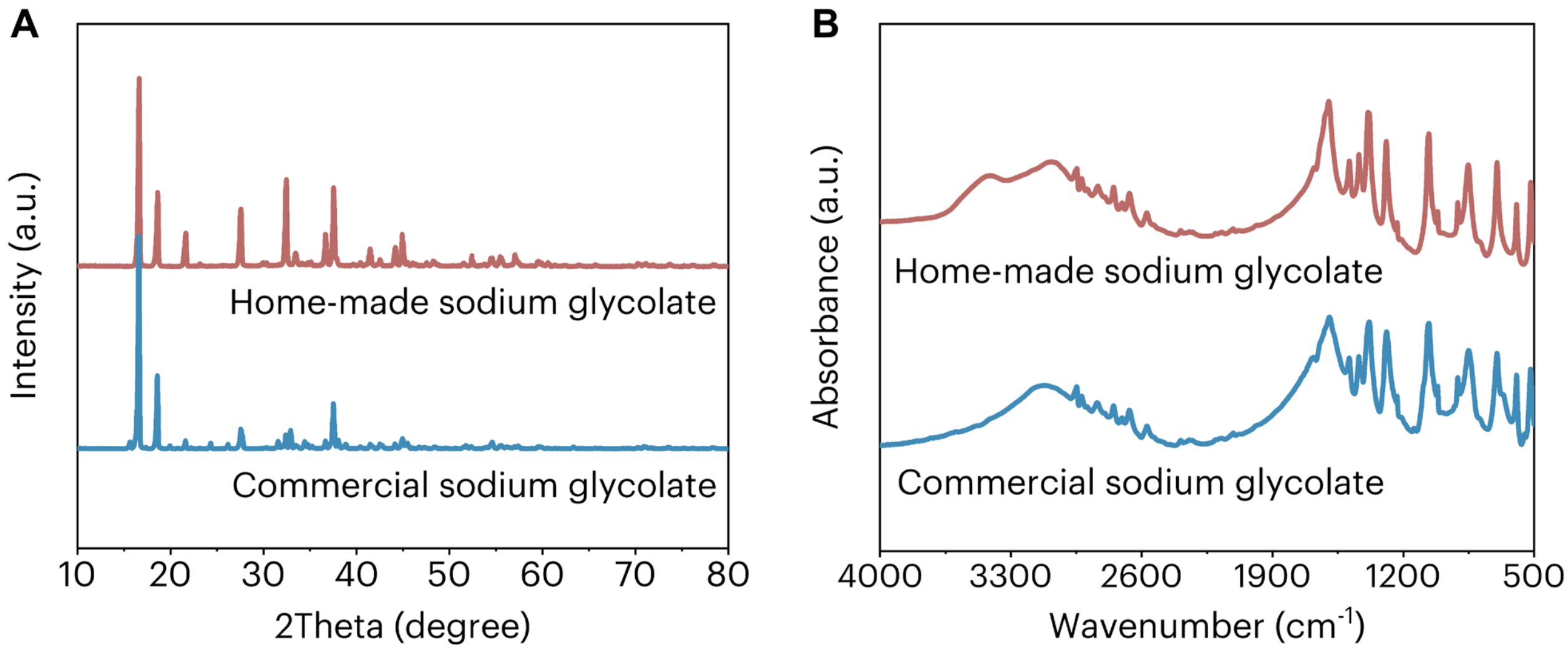

Fig. S89. Product analysis. (**A**) XRD patterns and (**B**) FTIR spectra of home-made sodium glycolate and commercial sodium glycolate.

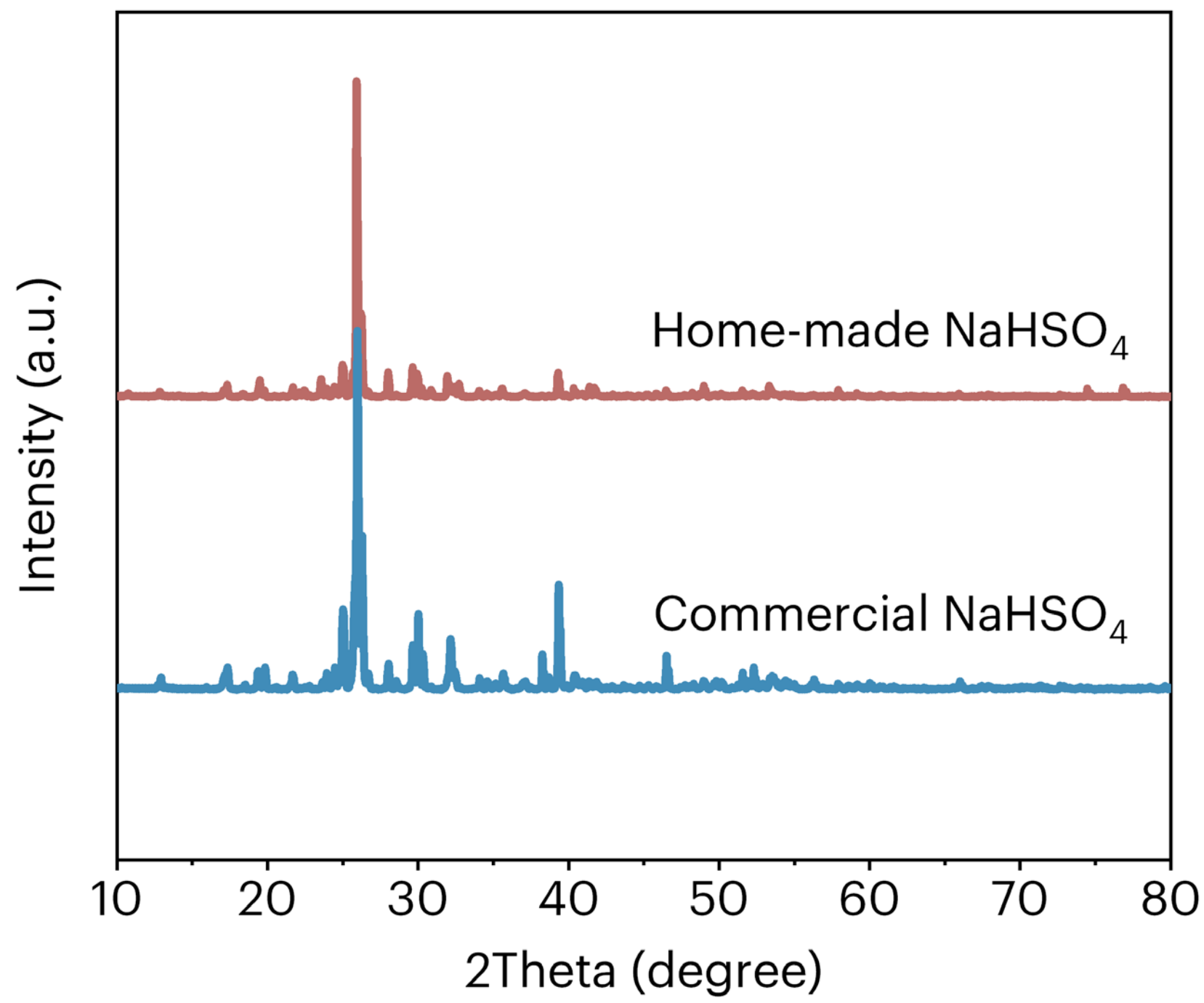

Fig. S90. Product analysis. XRD patterns of home-made $NaHSO_4$ and commercial $NaHSO_4$.

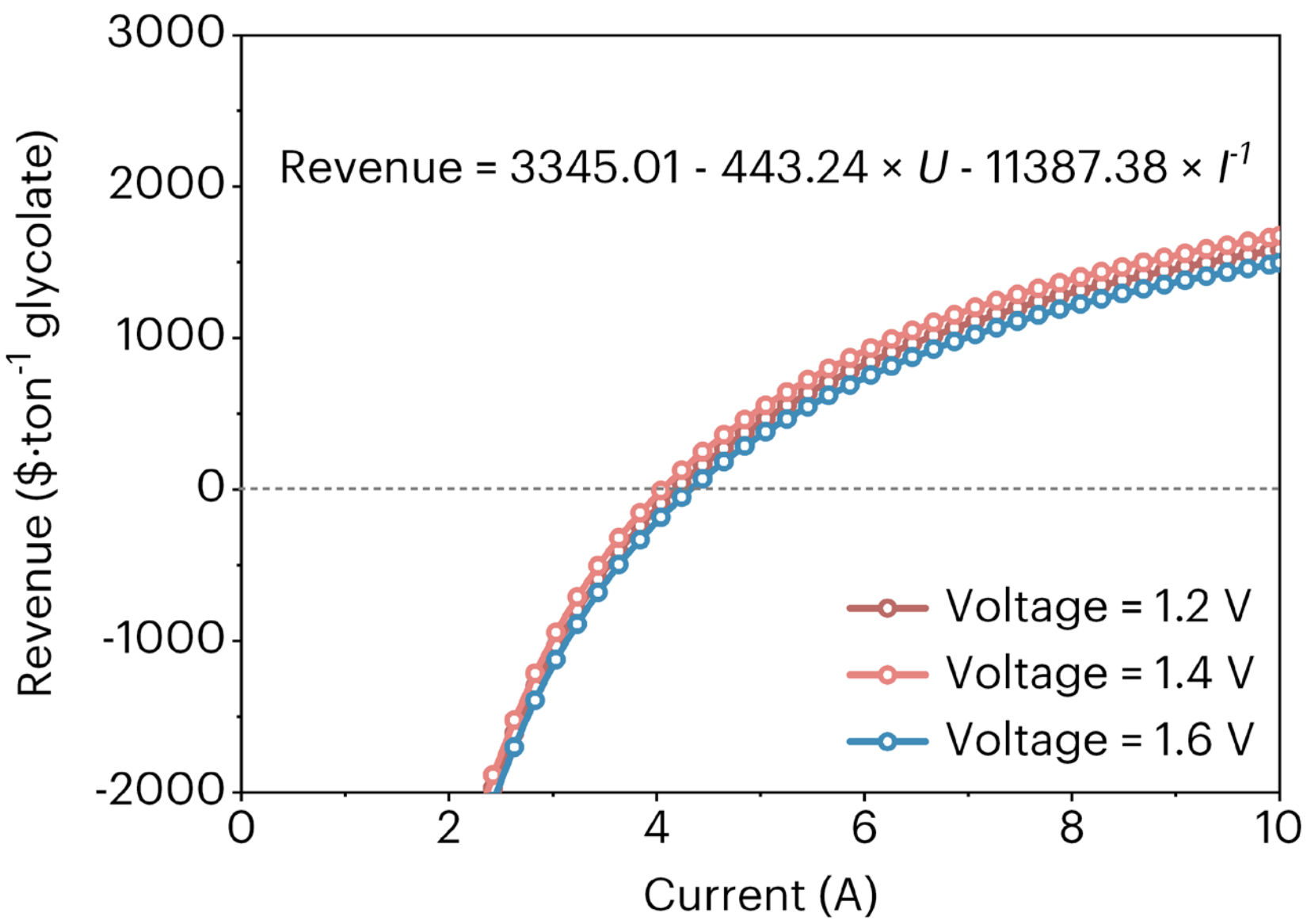

Fig. S91. Techno-economic analysis. Techno-economic analysis of the operating condition for the synthesis of glycolates through electrochemical PET upgrading.

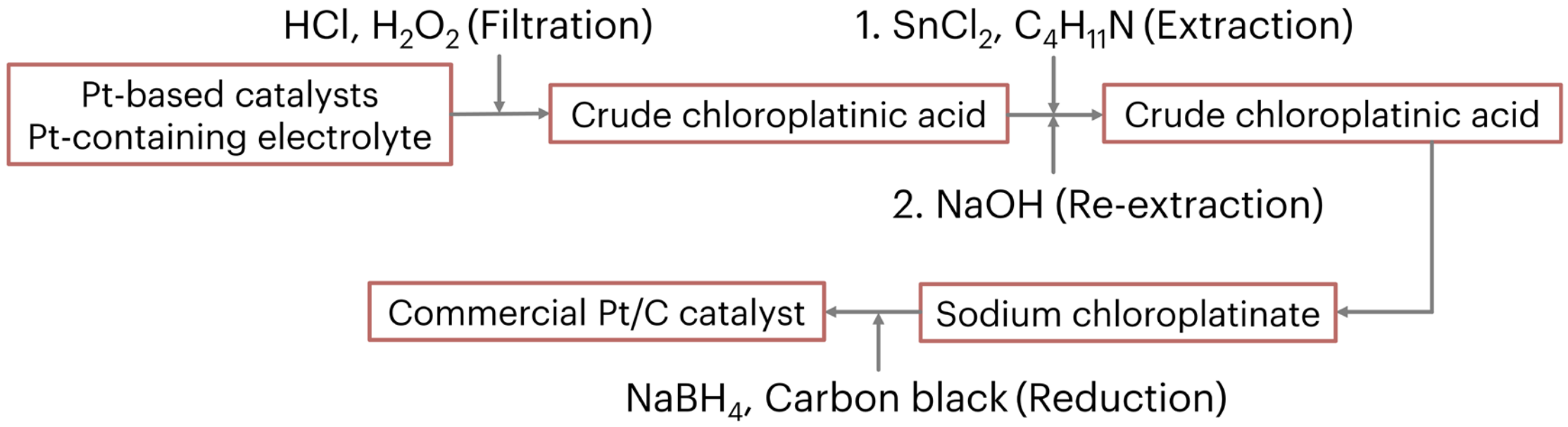

Fig. S92. Technical schematic diagram. The extra technical route for recycling Pt-based catalyst and remaining Pt-containing electrolyte.

Supplementary Tables

Table S1. The valence ratio calculations of Pt and Co in $NiCoO_x$, $Pt/NiCo(OH)_x$, Pt NPs/NF, $Pt/NiCo(OH)_x$ (Post EGOR stability test) and $Pt/NiCo(OH)_x$ (Post HER stability test) based on the XPS peak area.

Sample	Co^0 (%)	Co^{2+} (%)	Co^{3+} (%)	Pt^0 (%)	Pt^{2+} (%)
$NiCoO_x$	11.6	19.9	68.5	/	/
$Pt/NiCo(OH)_x$	7.2	27.0	65.8	69.9	30.1
Pt NPs/NF	/	/	/	67.1	32.9
$Pt/NiCo(OH)_x$ (Post EGOR)	/	25.4	74.6	66.7	33.3
$Pt/NiCo(OH)_x$ (Post HER)	5.2	20.7	78.1	67.6	32.4

Table S2. EXAFS fitting parameters of different samples at the Pt L_3-edge (S_0^2 = 0.74).

Sample	Path	CN	R (Å)	$\sigma^2 \times 10^{-3}$ (Å^2)	ΔE_0 (eV)	R-factor
Pt foil	Pt-Pt	12	2.76	4.25	7.48	0.001
$Pt/NiCo(OH)_x$	$Pt-Pt_1$	1.92	2.37	3.15	12.32	0.0089
	Pt-Pt	7.26	2.77	4.83		
Pt NPs/NF	Pt-Pt	10.91	2.75	4.86	8.49	0.003
NaOH-OCP	Pt-O	0.70	2.60	5.13	7.34	0.009
	Pt-Pt	8.24	2.10	3.00		
Added EG	$Pt-Pt_1$	1.14	2.47	3.05	10.89	0.02
	Pt-Pt	7.18	2.70	5.75		
EGOR-1.1 V	Pt-O	0.66	2.06	3.00	10.95	0.013
	$Pt-Pt_1$	1.07	2.38	7.06		
	Pt-Pt	6.57	2.78	7.85		
Cut-off	$Pt-Pt_1$	0.62	2.51	3.33	8.45	0.011
	Pt-Pt	6.46	2.76	5.60		

Note: The obtained S_0^2 was fixed to 0.74 by fitting Pt foil in the Pt L_3-edge of different samples. The suitable range for fitting EXAFS data was selected in the K-range of 2.7 - 8.7 Å^{-1} and R range of 1.45 - 3.34 Å.

Table S3. Comparison of the EGOR performance (without *iR* correction) for Pt/NiCo(OH)$_x$ with previously reported state-of-art noble-metal based catalysts.

Catalyst	Stability time (h)	Current density at 0.7 V *vs.* RHE (mA·cm^{-2})	Faradaic efficiency (%)	Reference
Pt/NiCo(OH)$_x$	3000	127.9	95.1	This work
LM-PdCu	2	8	96.8	*Han et al., 2025 (51)*
Pd-Ni(OH)$_2$	200	105	94.1	*Liu et al., 2023 (40)*
Au-Ni(OH)$_2$	1	20	91.0	*Yan et al., 2023 (20)*
PdAg/NF	2	30	92.0	*Si et al., 2021 (19)*
(PtIr)(FeMoBi)	30	50	95.0	*Hao et al., 2025 (38)*
PdCu	2.78	70	/	*Hu et al., 2018 (52)*
PdMn SANFs	24	110	94.3	*Liu et al., 2025 (33)*
Pd$_x$Ag$_y$	500	70	97.0	*Wang et al., 2023 (53)*
Pd/NiMoO$_4$/NF	1500	65	98.9	*Shi et al., 2024 (1)*
Pt-Ni(OH)$_2$	5	70	93.0	*Liu et al., 2024 (54)*
Pd$_{67}$Ag$_{33}$	2	30	92.7	*Chen et al., 2024 (55)*
PdCuNiCoFe	300	80	98.6	*Huang et al., 2026 (56)*
Pt$_1$/RuO$_2$	500	90	95.3	*Liu et al., 2025 (57)*

m-HEA/NF	125	60	99.0	*Zhou et al., 2026 (58)*
I-Pd	204	50	98.3	*Wang et al., 2026 (59)*
PtPdRuOs/NF	8	75	95.2	*Wang et al., 2026 (60)*

Table S4. Comparison of the ampere-level performance with recently reported alcohol-oxidation assisted system.

System	Stability time (h)	System voltage (V)	Current (A)	Reference
Pt/NiCo(OH)$_x$ // Pt/NiCo(OH)$_x$	250	1.18	10	This work
Au/CoOOH // NF	1.7	2	4.8	*Li et al., 2022 (61)*
Au/Ni(OH)$_2$ // NF	16	1.4	4.7	*Yan et al., 2023 (20)*
Pd-CuCo$_2$O$_4$ // CoP	110	1.4	2	*Liu et al., 2024 (50)*
Cu$_x$Ni$_{2-x}$P // NF	8	2	2.1	*Ma et al., 2024 (62)*
Mo$_1$-CoSe/NF // D-SA-C	30	0.94	0.1	*Qi et al., 2025 (63)*
Cr^{3+}-Ni(OH)$_2$ // PdCu	120	2.2	1	*Li et al., 2023 (64)*
Pd-Ni(OH)$_2$ // Ni$_2$P	50	1.2	0.9	*Liu et al., 2023 (40)*
Pd/NF // NF	24	1.2	0.6	*Xia et al., 2025 (29)*

Table S5. Calculating data of the techno-economic analysis for glycolate production through electrochemical PET upgrading (average current of 6.4 A at 1.18 V) and GA production through formaldehyde carbonylation process.

Target	Price of cost/revenue for electrochemical PET upgrading ($\$\cdot ton^{-1}$ glycolate)	Price of cost/revenue for formaldehyde carbonylation process ($\$\cdot ton^{-1}$ GA)
Labor cost	2393.3	886.6
Electricity cost	435.1	26.6
Separation cost	2365.4	413.3
Capital cost	595.4	122.9
Maintenance cost	119.1	21.9
Raw material cost	4998.3	397.2
H_2 revenue	152.5	/
PTA revenue	2323.3	/
Glycolate revenue	4772.5	/
$NaHSO_4$ revenue	4730.6	/
Crude EG revenue	/	86.4
GA (70% liquid) revenue	/	1980.0

REFERENCES

1. K. Shi, D. Si, X. Teng, L. Chen, J. Shi, Pd/$NiMoO_4$/NF electrocatalysts for the efficient and ultra-stable synthesis and electrolyte-assisted extraction of glycolate. *Nat. Commun.* **15**, 2899 (2024).

2. J. Chen, M. Jiang, F. Zhang, L. Wang, J. Yang, Interstitial boron atoms in pd aerogel selectively switch the pathway for glycolic acid synthesis from waste plastics. *Adv. Mater.* **36**, 2401867 (2024).

3. X. Zhou, M. Zha, J. Cao, H. Yan, X. Feng, D. Chen, C. Yang, Glycolic acid production from ethylene glycol via sustainable biomass energy: Integrated conceptual process design and comparative techno-economic-society-environment analysis. *ACS Sustain. Chem. Eng.* **9**, 10948–10962 (2021).

4. Research and Markets, Glycolic Acid Market by Grade, Application - Global Forecast 2025-2030 (Report), ID: 5674271 (2024); https://researchandmarkets.com/report/glycolic-acid#rela0-5005230.

5. Y. Chen, X. Ren, Q. Wei, Conversion of *Undaria pinnatifida* residue to glycolic acid with recyclable methylamine in low temperature hydrothermal liquefaction. *Bioresour. Technol.* **228**, 47–55 (2017).

6. S. Yunhai, S. Houyong, C. Haiyong, L. Deming, L. Qinghua, Synergistic extraction of glycolic acid from glycolonitrile hydrolysate. *Ind. Eng. Chem. Res.* **50**, 8216–8224 (2011).

7. S. K. Bhattacharyya, D. Vir, High-pressure synthesis of glycolic acid from formaldehyde, carbon monoxide, and water in presence of nickel, cobalt, and iron catalysts. *Adv. Catal.* **9**, 625–635 (1957).

8. P. K. Samantaray, A. Little, D. M. Haddleton, T. McNally, B. Tan, Z. Sun, W. Huang, Y. Ji, C. Wan, Poly(glycolic acid) (PGA): A versatile building block expanding high performance and sustainable bioplastic applications. *Green Chem.* **22**, 4055–4081 (2020).

9. T. Wang, X. Cao, L. Jiao, Progress in hydrogen production coupled with electrochemical oxidation of small molecules. *Angew. Chem. Int. Ed. Engl.* **61**, e202213328 (2022).

10. J.-T. Ren, L. Chen, H.-Y. Wang, W.-W. Tian, Z.-Y. Yuan, Water electrolysis for hydrogen production: From hybrid systems to self-powered/catalyzed devices. *Energ. Environ. Sci.* **17**, 49–113 (2024).

11. H. Zhou, Y. Ren, B. Yao, Z. Li, M. Xu, L. Ma, X. Kong, L. Zheng, M. Shao, H. Duan, Scalable electrosynthesis of commodity chemicals from biomass by suppressing non-Faradaic transformations. *Nat. Commun.* **14**, 5621 (2023).

12. M. Macleod, H. P. H. Arp, M. B. Tekman, A. Jahnke, The global threat from plastic pollution. *Science* **373**, 61–65 (2021).

13. A. Rahimi, J. M. García, Chemical recycling of waste plastics for new materials production. *Nat. Rev. Chem.* **1**, 0046 (2017).

14. J. Chen, L. Zhang, L. Wang, M. Kuang, S. Wang, J. Yang, Toward carbon neutrality: Selective conversion of waste plastics into value-added chemicals. *Matter* **6**, 3322–3347 (2023).

15. J. Wang, X. Li, M. Wang, T. Zhang, X. Chai, J. Lu, T. Wang, Y. Zhao, D. Ma, Electrocatalytic valorization of poly(ethylene terephthalate) plastic and CO_2 for simultaneous production of formic acid. *ACS Catal.* **12**, 6722–6728 (2022).

16. H. Zhou, Y. Ren, Z. Li, M. Xu, Y. Wang, R. Ge, X. Kong, L. Zheng, H. Duan, Electrocatalytic upcycling of polyethylene terephthalate to commodity chemicals and H_2 fuel. *Nat. Commun.* **12**, 4679 (2021).

17. M. Song, Y. Wu, Z. Zhao, M. Zheng, C. Wang, J. Lu, Corrosion engineering of part-per-million single atom $Pt_1/Ni(OH)_2$ electrocatalyst for pet upcycling at ampere-level current density. *Adv. Mater.* **36**, e2403234 (2024).

18. Y. X. Leiu, K. M. Lim, Z.-J. Chiah, E. S.-Z. Mah, W.-J. Ong, Plastic-to-treasure: Innovative advances in photo/electro-catalytic upcycling technologies for commodity chemicals and fuels. *EcoEnergy* **3**, 217–253 (2025).

19. D. Si, B. Xiong, L. Chen, J. Shi, Highly selective and efficient electrocatalytic synthesis of glycolic acid in coupling with hydrogen evolution. *Chem. Catal.* **1**, 941–955 (2021).

20. Y. Yan, H. Zhou, S.-M. Xu, J. Yang, P. Hao, X. Cai, Y. Ren, M. Xu, X. Kong, M. Shao, Z. Li, H. Duan, Electrocatalytic ities. *J. Am. Chem. Soc.* **145**, 6144–6155 (2023).

21. K. Miyazaki, T. Matsumiya, T. Abe, H. Kurata, T. Fukutsuka, K. Kojima, Z. Ogumi, Electrochemical oxidation of ethylene glycol on Pt-based catalysts in alkaline solutions and quantitative analysis of intermediate products. *Electrochim. Acta* **56**, 7610–7614 (2011).

22. M. Du, Y. Zhang, S. Kang, C. Xu, Y. Ma, L. Cai, Y. Zhu, Y. Chai, B. Qiu, Electrochemical production of glycolate fuelled by polyethylene terephthalate plastics with improved techno-economics. *Small* **19**, e2303693 (2023).

23. P. Gao, Y. Zeng, P. Tang, Z. Wang, J. Yang, A. Hu, J. Liu, Understanding the synergistic effects and structural evolution of $Co(OH)_2$ and Co_3O_4 toward boosting electrochemical charge storage. *Adv. Funct. Mater.* **32**, 2108644 (2022).

24. S. Ye, F. Luo, Q. Zhang, P. Zhang, T. Xu, Q. Wang, D. He, L. Guo, Y. Zhang, C. He, X. Ouyang, M. Gu, J. Liu, X. Sun, Highly stable single Pt atomic sites anchored on aniline-stacked graphene for hydrogen evolution reaction. *Energ. Environ. Sci.* **12**, 1000–1007 (2019).

25. G. Luo, M. Song, L. An, X. Huang, Q. Zhang, C. Zhang, T. Shen, S. Wang, D. Wang, Synergistically enhanced ORR and HER performance on Co-N-C coupled in-situ generated PtCo intermetallic. *J. Energy Chem.* **100**, 721–729 (2025).

26. K. L. Zhou, Z. Wang, C. B. Han, X. Ke, C. Wang, Y. Jin, Q. Zhang, J. Liu, H. Wang, H. Yan, Platinum single-atom catalyst coupled with transition metal/metal oxide heterostructure for accelerating alkaline hydrogen evolution reaction. *Nat. Commun.* **12**, 3783 (2021).

27. D. Long, Y. Liu, X. Ping, F. Chen, X. Tao, Z. Xie, M. Wang, M. Wang, L. Li, L. Guo, S. Chen, Z. Wei, Constructing CO-immune water dissociation sites around Pt to achieve stable operation in high CO concentration environment. *Nat. Commun.* **15**, 8105 (2024).

28. Y. Hao, S.-F. Hung, W.-J. Zeng, Y. Wang, C. Zhang, C.-H. Kuo, L. Wang, S. Zhao, Y. Zhang, H.-Y. Chen, S. Peng, Switching the oxygen evolution mechanism on atomically dispersed Ru for enhanced acidic reaction kinetics. *J. Am. Chem. Soc.* **145**, 23659–23669 (2023).

29. T. Xia, J. Yang, Q. Ren, Y. Fu, Z. Zhang, Z. Li, M. Shao, X. Duan, Promoting alcohols electrooxidation coupled with hydrogen production via asymmetric pulse potential strategy. *Angew. Chem. Int. Ed. Engl.* **64**, e202420992 (2025).

30. P. Zhou, X. Lv, S. Tao, J. Wu, H. Wang, X. Wei, T. Wang, B. Zhou, Y. Lu, T. Frauenheim, X. Fu, S. Wang, Y. Zou, Heterogeneous-interface-enhanced adsorption of organic and hydroxyl for biomass electrooxidation. *Adv. Mater.* **34**, e2204089 (2022).

31. L. Chen, Z.-H. Yin, J.-Y. Cui, C.-Q. Li, K. Song, H. Liu, J.-J. Wang, Unlocking lattice oxygen on selenide-derived NiCoOOH for amine electrooxidation and efficient hydrogen production. *J. Am. Chem. Soc.* **146**, 27090–27099 (2024).

32. Y. Wang, Y.-Q. Zhu, Z. Xie, S.-M. Xu, M. Xu, Z. Li, L. Ma, R. Ge, H. Zhou, Z. Li, X. Kong, L. Zheng, J. Zhou, H. Duan, Efficient electrocatalytic oxidation of glycerol via promoted OH* generation over single-atom-bismuth-doped spinel Co_3O_4. *ACS Catal.* **12**, 12432–12443 (2022).

33. S. Liu, K. Ma, H. Teng, W. Miao, X. Zhou, X. Cui, X. Zhou, L. Jiang, S. Guo, Self-assembled gap-rich PdMn nanofibers with high mass/electron transport highways for electrocatalytic reforming of waste plastics. *Adv. Mater.* **37**, e2411148 (2025).

34. L. W. H. Leung, M. J. Weaver, Real-time FTIR spectroscopy as a quantitative kinetic probe of competing electrooxidation pathways of small organic molecules. *J. Phys. Chem.* **92**, 4019–4022 (1988).

35. H. Shi, T. Wang, J. Liu, W. Chen, S. Li, J. Liang, S. Liu, X. Liu, Z. Cai, C. Wang, D. Su, Y. Huang, L. Elbaz, Q. Li, A sodium-ion-conducted asymmetric electrolyzer to lower the operation voltage for direct seawater electrolysis. *Nat. Commun.* **14**, 3934 (2023).

36. X. Zheng, X. Zheng, M. Gao, Y. Liu, H. Pan, W. Sun, Platinum-nickel oxide cluster-cluster heterostructure enabling fast hydrogen evolution for anion exchange membrane water electrolyzers. *Angew. Chem. Int. Ed. Engl.* **64**, e202422062 (2025).

37. M. Fleischmann, K. Korinek, D. Pletcher, The oxidation of organic compounds at a nickel anode in alkaline solution. *J. Electroanal. Chem. Interfacial Electrochem.* **31**, 39–49 (1971).

38. J. Hao, T. Wang, J. Cai, G. Gao, Z. Zhuang, R. Yu, J. Wu, G. Wu, S. Lu, X. Wang, M. Du, D. Wang, H. Zhu, Suppression of structural heterogeneity in high-entropy intermetallics for electrocatalytic upgrading of waste plastics. *Angew. Chem. Int. Ed. Engl.* **64**, e202419369 (2025).

39. G. Zhao, J. Lin, M. Lu, L. Li, P. Xu, X. Liu, L. Chen, Potential cycling boosts the electrochemical conversion of polyethylene terephthalate-derived alcohol into valuable chemicals. *Nat. Commun.* **15**, 8463 (2024).

40. F. Liu, X. Gao, R. Shi, Z. Guo, E. C. M. Tse, Y. Chen, Concerted and selective electrooxidation of polyethylene-terephthalate-derived alcohol to glycolic acid at an industry-level current density over a Pd-$Ni(OH)_2$ catalyst. *Angew. Chem. Int. Ed. Engl.* **62**, e202300094 (2023).

41. X. Zhang, L. Xia, G. Zhao, B. Zhang, Y. Chen, J. Chen, M. Gao, Y. Jiang, Y. Liu, H. Pan, W. Sun, Fast and durable alkaline hydrogen oxidation reaction at the electron-deficient ruthenium-ruthenium oxide interface. *Adv. Mater.* **35**, e2208821 (2023).

42. C. Wan, Z. Zhang, J. Dong, M. Xu, H. Pu, D. Baumann, Z. Lin, S. Wang, J. Huang, A. H. Shah, X. Pan, T. Hu, A. N. Alexandrova, Y. Huang, X. Duan, Amorphous nickel hydroxide shell tailors local chemical environment on platinum surface for alkaline hydrogen evolution reaction. *Nat. Mater.* **22**, 1022–1029 (2023).

43. L. Yu, L. Wu, S. Song, B. McElhenny, F. Zhang, S. Chen, Z. Ren, Hydrogen generation from seawater electrolysis over a sandwich-like NiCoN|Ni_xP|NiCoN microsheet array catalyst. *ACS Energy Lett.* **5**, 2681–2689 (2020).

44. H. Zhou, F. Yu, Y. Huang, J. Sun, Z. Zhu, R. J. Nielsen, R. He, J. Bao, W. A. Goddard Iii, S. Chen, Z. Ren, Efficient hydrogen evolution by ternary molybdenum sulfoselenide particles on self-standing porous nickel diselenide foam. *Nat. Commun.* **7**, 12765 (2016).

45. G. Kresse, J. Furthmüller, Efficient iterative schemes for ab initio total-energy calculations using a plane-wave basis set. *Phys. Rev. B* **54**, 11169–11186 (1996).

46. G. Kresse, D. Joubert, From ultrasoft pseudopotentials to the projector augmented-wave method. *Phys. Rev. B* **59**, 1758–1775 (1999).

47. J. P. Perdew, K. Burke, M. Ernzerhof, Generalized gradient approximation made simple. *Phys. Rev. Lett.* **77**, 3865–3868 (1996).

48. S. Grimme, J. Antony, S. Ehrlich, H. Krieg, A consistent and accurate ab initio parametrization of density functional dispersion correction (DFT-D) for the 94 elements H-Pu. *J. Chem. Phys.* **132**, 154104–154100 (2010).

49. H. Shin, K. U. Hansen, F. Jiao, Techno-economic assessment of low-temperature carbon dioxide electrolysis. *Nat. Sustain.* **4**, 911–919 (2021).

50. K. Liu, X. Gao, C.-X. Liu, R. Shi, E. C. M. Tse, F. Liu, Y. Chen, Energy-saving hydrogen production by seawater splitting coupled with PET plastic upcycling. *Adv. Energy Mater.* **14**, 2304065 (2024).

51. S. Han, L. Sun, D. Fan, B. Liu, Pulsed electrosynthesis of glycolic acid through polyethylene terephthalate upcycling over a mesoporous PdCu catalyst. *Nat. Commun.* **16**, 3426 (2025).

52. T. Hu, Y. Wang, H. Xiao, W. Chen, M. Zhao, J. Jia, Shape-control of super-branched Pd-Cu alloys with enhanced electrocatalytic performance for ethylene glycol oxidation. *Chem. Commun.* **54**, 13363–13366 (2018).

53. Y. Wang, K. Liu, F. Liu, C. Liu, R. Shi, Y. Chen, Selective electro-reforming of waste polyethylene terephthalate-derived ethylene glycol into C_2 chemicals with long-term stability. *Green Chem.* **25**, 5872–5877 (2023).

54. X. Liu, X. He, D. Xiong, G. Wang, Z. Tu, D. Wu, J. Wang, J. Gu, Z. Chen, Electro-reforming of PET plastic to C_2 chemicals with concurrent generation of hydrogen and electric energy. *ACS Catal.* **14**, 5366–5376 (2024).

55. J. Chen, F. Zhang, M. Kuang, L. Wang, H. Wang, W. Li, J. Yang, Unveiling synergy of strain and ligand effects in metallic aerogel for electrocatalytic polyethylene terephthalate upcycling. *Proc. Natl. Acad. Sci. U.S.A.* **121**, e2318853121 (2024).

56. B. Huang, X. Chen, J. Luo, J. Cao, D. Fan, M. Bi, F. Feng, L. Huang, C. Y. En, Y. Zhang, Z. Lyu, M. Zhao, Simultaneously engineering the amorphous phase and branch morphology of high-entropy alloy nanomaterials for enhanced ethylene glycol oxidation. *Adv. Mater.* **38**, e73384 (2026).

57. F. Liu, J. Zhou, X. Gao, R. Shi, Z. Guo, E. C. M. Tse, Y. Chen, Modulating adsorption behavior by single-site Pt on RuO_2 for efficient electrosynthesis of glycolic acid from plastic wastes. *Angew. Chem. Int. Ed. Engl.* **64**, e202422183 (2025).

58. Y. Zhou, T. Tian, S. Jiang, R. Shen, H. Yu, Z. Wang, H. Wang, L. Wang, K. Deng, Alternating pulse driven periodic reactivation of high-entropy mesoporous film boosts continuous membrane-free PET waste upcycling coupled with H_2 production. *Adv. Funct. Mater.* **36**, e11835 (2026).

59. H. Wang, X. Dong, F. Liu, X. Chang, T. Yu, J. Chang, M. Liu, R. Zou, Y. Yang, C.-C. Hou, Lattice-distortion-driven electron delocalization enables efficient electrosynthesis of glycolic acid and terephthalic acid from plastic wastes. *J. Am. Chem. Soc.* **148**, 1406–1418 (2026).

60. Z. Wang, J. Song, J. Geng, Y. Xu, H. Wang, S. Yin, K. Deng, H. Yu, L. Wang, Revealing electronic effect in mesoporous medium-entropy alloy for promoting electrocatalytic upcycling of polyethylene terephthalate plastic. *ACS Nano* **20**, 16501–16511 (2026).

61. Z. Li, Y. Yan, S.-M. Xu, H. Zhou, M. Xu, L. Ma, M. Shao, X. Kong, B. Wang, L. Zheng, H. Duan, Alcohols electrooxidation coupled with H_2 production at high current densities promoted by a cooperative catalyst. *Nat. Commun.* **13**, 147 (2022).

62. L. Ma, Y. Miao, J. Yang, Y. Fu, Y. Yan, Z. Zhang, Z. Li, M. Shao, Promoting electrocatalytic glycerol C-C bond cleavage to formate coupled with H_2 production over a $Cu_xNi_{2-x}P$ catalyst. *Adv. Energy Mater.* **14**, 2401061 (2024).

63. J. Qi, Y. Xia, X. Meng, J. Li, S. Yang, H. Zou, Y. Ma, Y. Zhang, Y. Du, L. Zhang, Z. Lin, J. Qiu, Cation-vacancy engineering in cobalt selenide boosts electrocatalytic upcycling of polyester thermoplastics at industrial-level current density. *Adv. Mater.* **37**, 2419058 (2025).

64. S. Li, S. Wang, J. He, K. Li, Y. Xu, M. Wang, S. Zhao, Y. Wang, X. Li, X. Zhong, J. Wang, Chromium-doped nickel oxide and nickel nitride mediate selective electrocatalytic oxidation of sterol intermediates coupled with H_2 evolution. *Angew. Chem. Int. Ed. Engl.* **62**, e202306553 (2023).
